# Electronic Transport in Porous Nanocrystals Enables Ultrasensitive Consistent Detection of Sulfur Dioxide under Variable Humidity

**DOI:** 10.1021/jacs.5c19328

**Published:** 2026-06-02

**Authors:** Elissa O. Shehayeb, Joseph Y. M. Chan, Giovanni Barcaro, Susanna Monti, Katherine A. Mirica

**Affiliations:** † Department of Chemistry, Burke Laboratory, 3728 Dartmouth College, Hanover, New Hampshire 03755, United States; ‡ CNR-IPCF, Institute of Chemical and Physical Processes, Area della Ricerca, Pisa I56124, Italy; § CNR-ICCOM, Institute of Chemistry of Organometallic Compounds, Area della Ricerca, Pisa I56124, Italy

## Abstract

The growing release of gaseous pollutants, such as sulfur dioxide (SO_2_), underscores the need for widely distributed monitoring and reliable electronic sensing platforms capable of detecting trace concentrations of such contaminants under real-world conditions. However, achieving high sensitivity and selectivity, while maintaining stable, reproducible sensing performance with conductive materials, particularly under fluctuating humidity, remains a significant challenge. This work reports a highly crystalline, conductive tetrapyrazinoporphyrazine (TPz)-based metal–organic framework (MOF), DC-103, featuring cobalt-centered TPz ligands and copper bis­(dioxolene) linkages. DC-103 exhibits rapid, robust, and humidity-resistant chemiresistive responses toward SO_2_ across diverse environments. Notably, DC-103 outperforms previously reported MOF-based SO_2_ sensors, achieving a limit of detection as low as 2.2 parts-per-billion (ppb) in air and maintaining consistent, reversible responses across 0–98% relative humidity levels. Furthermore, the sensor demonstrates excellent reusability in humid air, retaining consistent responses upon repeated exposures. Spectroscopic and computational analyses revealed distinct material–analyte interactions and redox changes of MOF components and sulfurous species under dry or humid nitrogen or air atmospheres. Collectively, these findings establish DC-103 as a robust chemiresistive sensor for SO_2_, with superior performance compared to structurally analogous or control structures, demonstrating applicability across diverse atmospheric conditions, and offering significant potential in occupational safety and environmental monitoring.

## Graphical abstract

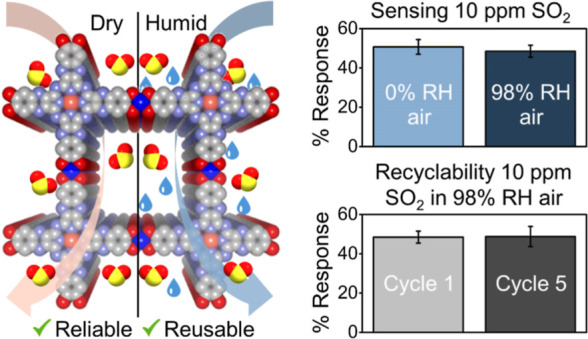

## 1 Introduction

The emission of sulfur dioxide (SO_2_) into the atmosphere poses significant environmental and public health risks due to its widespread and harmful effects on ecosystems and human populations.^1,2^ As a major atmospheric contaminant, SO_2_ not only contributes directly to air quality deterioration,^1,3^ but also indirectly to water and soil pollution through the formation of acid rain.^4,5^ Even at parts-per-million (ppm) levels, SO_2_ is associated with adverse pulmonary, cardiovascular, and neurological effects.^6,7^ These risks have prompted regulatory agencies, such as the Occupational Safety and Health Administration (OSHA), to set an SO_2_ permissible exposure limit (PEL) of 5 ppm.^8^ Although natural processes, such as volcanic eruptions^9,10^ and microbial activity in wetlands and oceans^11,12^ contribute to global SO_2_ emissions, anthropogenic activities account for approximately 65–75% of the released SO_2_.^13,14^ These processes include fossil fuel combustion for power generation and industrial activities,^15^ natural gas production,^16^ petroleum refining,^17,18^ or biomass burning.^19^ The ubiquity of these emissions underscores the urgent need for selective, sensitive, and reliable sensors capable of detecting SO_2_ under realistic environmental conditions.

Although satellite-based differential optical absorbance spectroscopy remote sensing from outer space has enabled large-scale monitoring of SO_2_ emissions in otherwise inaccessible regions,^20,21^ real-time, widely distributed, ground-level detection at concentrations relevant to human health remains underdeveloped.^1^ Accurate monitoring, particularly in areas near emission sources, is further complicated by fluctuating atmospheric humidity, with relative humidity (RH) levels ranging from 0 to 100%.^22^ Existing detection technologies include gas chromatography,^23^ fluorescence,^24^ infrared,^25,26^ and optical methods,^27^ quartz crystal microbalance,^28^ electrochemical sensors,^29^ and chemiresistive approaches.^30^ While chemiresistive sensors offer significant advantages for on-site and portable gas monitoring, many of the conventional techniques are limited by bulky instrumentation,^31^ high operational costs,^29,31^ compromised device longevity,^29^ high power consumption,^29^ slow response times,^32^ and the need for trained personnel.^31^ Materials previously explored for the chemiresistive detection of SO_2_, such as metal oxides,^33^ reduced graphene oxide composites,^34,35^ layered double hydroxides,^36^ carbon nanotubes,^37^ N-doped graphene,^38^ conductive polymers,^39^ perovskite oxides,^40^ and covalent–organic frameworks (COFs)^41,42^ are often impaired by poor sensitivity and stability, limited selectivity, irreversibility, complex synthetic procedures, and in some instances, high power requirements of resulting devices.^31,34,43–45^ Moreover, to mitigate interference from ambient humidity, many of these materials require additional surface functionalization, such as doping or coating with hydrophobic membranes,^46,47^ which often frequently hinder accessibility to active sites and reduce sensor efficiency.^48^ Thus, the development of humidity-tolerant, sensitive, and reusable chemiresistive sensors that function without external modifications remains a compelling need.^48^

Two-dimensional conductive metal–organic frameworks (2D cMOFs) have emerged as promising candidates to overcome these limitations.^49^ Their structural tunability, high surface area, abundant active sites, and intrinsic conductivity make them particularly suited for chemiresistive sensing applications.^50^ Although MOFs and COFs have been utilized for SO_2_ detection through luminescent,^51^ electrochemical,^52^ gravimetric,^53^ or chemiresistive techniques,^54^ their performance under conditions with variable humidity can be compromised compared to laboratory-controlled conditions due to interference of oxygen (O_2_) or water (H_2_O).^54^ For example, Feng and co-workers reported a 2D COF with ultrasensitive SO_2_ detection, but the response showed at least a 6-fold change in magnitude across nitrogen and air environments.^41^ Recently, our group reported the use of a textile-coated copper-hexahydroxytriphenylene, Cu_3_(HHTP)_2_, cMOF for the detection, capture, and detoxification of SO_2_.^54^ While demonstrating promising utility for dosimetric SO_2_ detection, this system exhibited limited sensing reversibility and substantial variation in sensing performance under ambient and humid conditions with decreased sensitivity and reversal of response direction,^54^ suggesting that further optimization of the molecular design is needed to achieve practically relevant functions. We reasoned that merging the established copper-bis­(dioxolene) nodes of proven utility in SO_2_ detection with a redox-stable ligand,^55,56^ formed of cobalt-centered tetrapyrazinoporphyrazine (TPz), to create a new cMOF, would be a valuable molecular design strategy for achieving rapid, sensitive, humidity-tolerant, and recyclable detection of sulfurous gases under real-life settings.

Here, we report the synthesis and characterization of a novel cMOF, DC-103, for the reliable detection of SO_2_ under realistic conditions. We introduce DC-103 as a new analog of the previously reported DC-100, DC-101, and DC-102 developed in our prior work at Dartmouth College (DC).^55^ DC-103 features cobalt-centered TPz units bridged via bis­(dioxolene) linkages, coordinated to copper ions, yielding highly crystalline long-range-ordered 2D architectures with lateral domains of around 100 nm. This framework demonstrates rapid response times (within 6 s), parts-per-billion (ppb)-level sensitivity with record-breaking low theoretical detection limits (as low as 2.2 ppb in air) with respect to other MOF-based SO_2_ sensors, stable chemiresistive performance at 5 ppm, the OSHA PEL concentration of SO_2_,^8^ and excellent reusability across multiple sensing cycles (at least six cycles with consistent performance). Importantly, the sensor maintains an excellent electrical conductivity of 7.6 × 10^–4^ S cm^–1^, exceeding its proton conductivity (2 × 10^–6^ S cm^–1^ at 303 K and 98% RH) even in saturated humid atmospheres, allowing for its robust performance in challenging environments, including ambient air and a wide range of RH levels. Spectroscopic and computational studies of DC-103 upon SO_2_ exposure under varying conditions revealed distinct material–analyte interactions that not only explain variations in sensing response features, but also highlight redox changes within the framework across different environments. Compared with the control structures used in this study, which exhibit unstable responses across varying atmospheres, DC-103 demonstrates the advantage of leveraging the structural modularity of cMOFs for enhancing applied performance. In particular, this study suggests that a key design principle for functional materials with robust sensing performance in humid and air environments is high electrical conductivity dominated by electronic transport, with minimal contributions from mass transport of charged species, such as protonic carriers that can arise in humid environments. By combining structural robustness, sensitivity, and humidity tolerance, DC-103 establishes a new benchmark for chemiresistive SO_2_ detection, enabling applicability in environments prone to humidity fluctuations and pollutant exposure.

## 2 Results and Discussion

### 2.1 MOF Synthesis and Characterization

We synthesized DC-103 from a novel octahydroxy-functionalized TPz linker with a central cobalt ion, CoTPz­(OH)_8_, coordinated to copper bridging ions through bis­(dioxolene) linkages by following a similar approach to our previous report,^55^ as detailed in Figures S1–S12 and Section S2. Building upon optimization efforts conducted for other TPz-based MOFs^55^ (see Figure S13, Table S1, and Section S3), we showed that a reaction including 0.44 mM concentration of CoTPz­(OH)_8_ monomer in anhydrous dimethyl sulfoxide (DMSO), 2.1 equivalence of copper­(II) nitrate hemipentahydrate as a copper salt precursor, and 400 equivalence of ethylene diamine (EDA) as a base additive heated at 85 °C for 15 h yielded a crystalline framework material of DC-103 (simulated eclipsed packing structure shown in Figure 1a and MOF model dynamics shown in Figure 1b). Powder X-ray diffraction (PXRD) suggested the high crystallinity of DC-103 with sharp diffraction peaks at 2θ = 3.9°, 5.6°, 7.9°, and 27.5°, corresponding to the (100), (110), (200), and {001} facet planes, respectively, which are well-aligned with the simulated eclipsed packing structure of DC-103 (Figures 1c and S14). High-resolution transmission electron microscopy (HR-TEM) coupled with selected area electron diffraction (SAED) further demonstrated the long-range crystalline order of DC-103 crystallites, revealing uniformly distributed square pores spanning the ∼100 nm crystallites (Figures 1d,e and S15). The experimental (100) interplanar spacing of 2.19 nm, extracted from TEM images (Figure S16), is in good agreement with the simulated DC-103 structure, which predicts an average spacing of approximately 2.2 nm (Figure 1a). Scanning electron microscopy (SEM) images of DC-103 corroborated these findings, showing sheet-like crystallites with uniform lateral distributions of around 100 nm (Figure S17).

**Figure 1:**
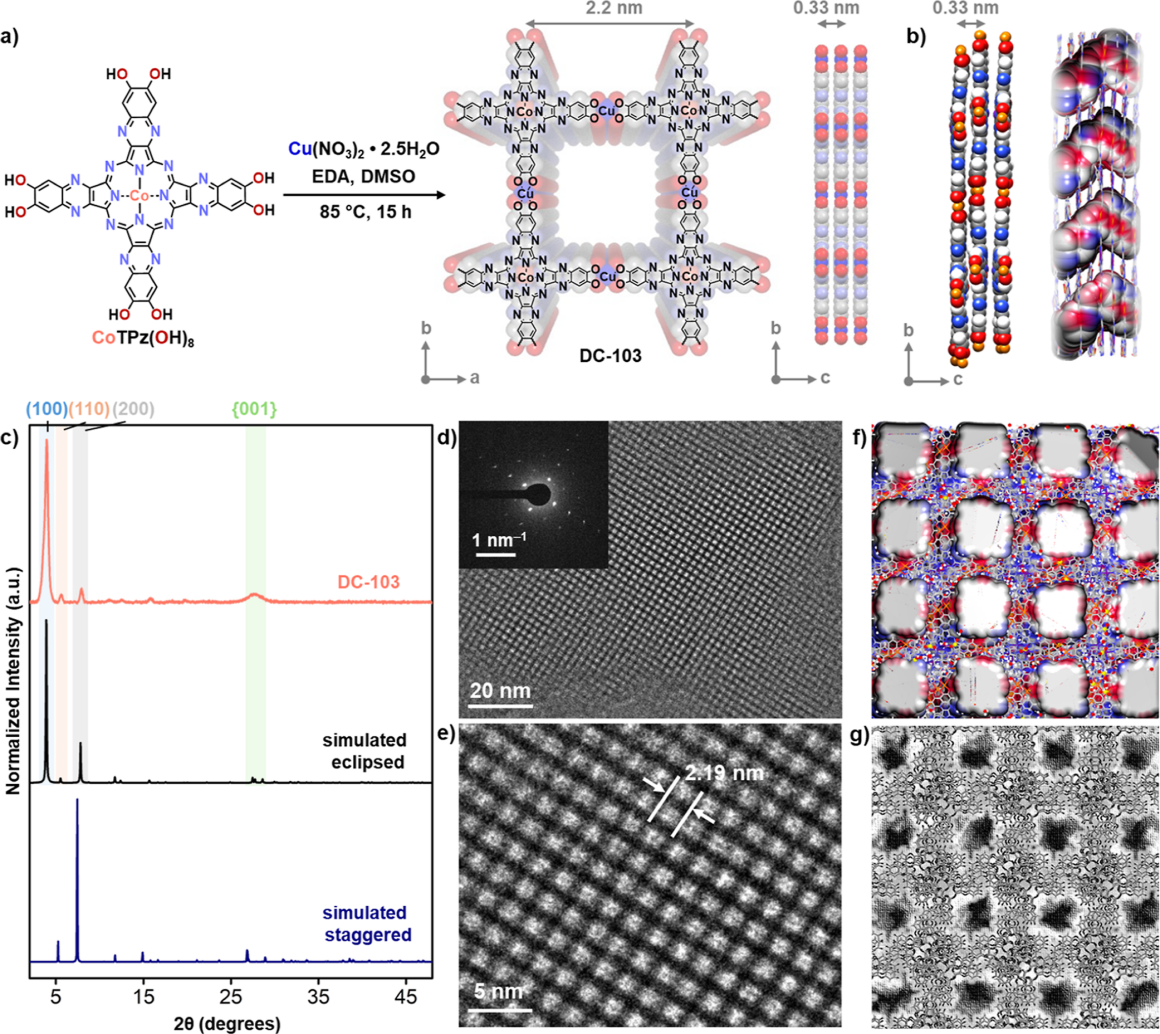
(a) Schematic description of DC-103 synthesis with simulated eclipsed stacking pattern. (b) Side view of the channels from optimized MD structure simulations of DC-103. (c) PXRD diffraction pattern of DC-103 compared to the simulated eclipsed structure. (d) HR-TEM image showing a single crystal of DC-103 with an inset of its corresponding SAED pattern. (e) A zoomed-in snapshot of the HR-TEM image of DC-103 at high magnification. (f) Top view of the MOF channels filled with balls (gray areas) to determine size and shape (CAVER software). (g) Top view of the MOF model (black and white sticks) superimposed on the zoomed microscopy image.

While energy dispersive X-ray (EDX) spectroscopy, inductively coupled plasma–mass spectrometry (ICP–MS), CHN combustion analysis, and thermogravimetric analysis (TGA) supported the expected elemental composition of DC-103 (Figures S18 and S19 and Table S2), attenuated total reflectance Fourier-transform infrared (ATR-FTIR), electron paramagnetic resonance (EPR), and X-ray photoelectron spectroscopy (XPS) revealed the coordination network and oxidation states of the components (Figures S20–S23). In particular, the linker moieties in DC-103 possess semiquinoidal forms, as evidenced by the approximate 50:50 distribution of C–O to C=O binding energies within the O 1s XPS range, consistent with a mixed-valence bridging ion composition of a 46:54 ratio of Cu­(II) to Cu­(I), Figure S22.^54^ The presence of an EPR signal at *g* = 2.068 in Figure S23 further confirmed the incorporation of paramagnetic Cu­(II) centers into the framework.^57^

N_2_ sorption isotherms at 77K gave a BET surface area of 313 m^2^ g^–1^, confirming the permanent porosity of DC-103 (Figure S24). Electrical characterization of pressed pellets demonstrated conductivities of 7.6 × 10^–4^ S cm^–1^ (four-point probe), and minimal variation after exposure to saturated humidity for 2 days, with a two-point probe conductivity of 1.4 × 10^–5^ S cm^–1^ (Section S4.8). Proton conductivity measurements under 98% RH at 303 K yielded a value of 2 × 10^–6^ S cm^–1^ (Figures S25–S26), markedly lower than the electronic conductivity, suggesting that electronic transport may continue to dominate in humid environments. Taken together, these properties suggest that DC-103 may serve as a promising platform for reliable chemiresistive gas sensing under variable humidity conditions.

### 2.2 Computational Characterization of DC-103 Structure

To disclose the structure and evolution of DC-103, we performed classical reactive molecular dynamics (MD) simulations using the ReaxFF force field and compared the results with experimental findings. The geometric features of DC-103 and the oxidation states of the copper ions were confirmed by theoretical calculations on a representative model system (details are reported in Section S5). The simulations were based on an atomistic supramolecular complex, whose size fitted large portions of the experimental material. The used force field, validated in earlier investigations,^54,58^ was ad hoc parametrized to efficiently reproduce the system’s evolution, including the adsorption and migration of the guest species. After equilibration of the empty model structure, which corresponded exactly to the initially stacked, eclipsed-plane configuration (schematic shown in Figure 1a), a remarkable change was observed. The layers shifted in response to steric hindrance, electrostatic repulsion, and intermolecular interactions with neighboring species, and the organic components rearranged their orientations through concerted translations and rotations (Figures S27–S29). This scenario is the most probable because the eclipsed positions of the cobalt metal ions created strong cation–cation repulsions, forcing the organic linkers into high-energy configurations with increasing steric strain within the framework. Even though the eclipsed arrangement could have created, in principle, larger, straight, one-dimensional pore channels, its thermodynamic stability was greatly reduced compared to the more staggered phases. Several snapshots were extracted from the molecular dynamics trajectories and subsequently sampled at the density functional theory (DFT) level of theory, still using representative models (Figures S27–S29). Through local geometry optimizations, we confirmed the dynamics prediction of a stabilized staggered layering (Figure 1b and Scheme S3). The resulting energy landscape was flat, with many configurations lying close in energy, indicative of a highly fluxional layered morphology, as observed in other analogous systems recently explored.^54^ Support for this picture comes from the experimental high-resolution XPS spectra, shown in Figure S22, which lack the component peak at 778 eV, suggesting the absence of direct Co–Co bonds, consistent with these claims. The MOF model dynamics led to the remodeling of the channels into slightly tortuous tunnels, with portions inclined by about 40° relative to the adjacent layer (Figure 1b). A comparison of the optimized staggered model (where the interplane and Co–Co distances are approximately 3.6 and 4.4 Å, respectively) with the high-resolution microscopy images (Figure 1f) indicates good agreement of the model with the experimental data. From the TEM image, it is evident that the mouths of the channels are not perfectly square black holes, but rather variable-sized black spots surrounded by gray-shaded areas, which implies layer shifts, as shown in the superimposed optimized MOF coordinates (Figure 1g). The inspection of partial charges on copper atoms revealed that DC-103 had a mixed composition of Cu­(II) and Cu­(I) with a ratio of about 46:54, consistent with the experimental results (Figure S22).

### 2.3 Chemiresistive Sensing Responses of DC-103 toward SO_2_

To examine the gas sensing performance of DC-103, we prepared chemiresistive devices by dropcasting 25 μL of MOF suspension in water (1 mg mL^–1^) onto gold interdigitated electrodes (IDEs) with 10 μm-wide gaps and drying them in a vacuum chamber for 2 h, for details see Section S6.1, Figures S30–S33, and Table S3. For precise delivery of targeted gas concentrations, we regulated gas flow rates using mass flow controllers directed into a Teflon chamber equipped with gas inlet and outlet ports, within which the electrodes are enclosed; for details, see Section S6.2 and Figure S32. Owing to the good conductivity of DC-103, the resulting devices exhibited Ohmic-contact resistance in the range of 0.1–40 kΩ (Figure S31), allowing for the utilization of a low driving voltage of 0.1 V in the chronoamperometric sensing experiments. We evaluated the response of DC-103 using the negative normalized conductance (−Δ*G*/*G*_0_), which revealed a concentration-dependent increase in response percentages toward SO_2_ across concentrations ranging from 2 to 40 ppm in dry nitrogen (N_2_) and dry air atmospheres (Figures 2a,b and S34–S35). The overall sensing ability of DC-103 was retained in the presence of interferent gaseous species in dry air, with an expected composition^59^ of N_2_ (78.0%), oxygen gas (O_2_, 20.9%), argon (Ar, 0.9%), carbon dioxide (CO_2_, 0.03%), and others (<0.2%), as compared to pure dry N_2_.

**Figure 2:**
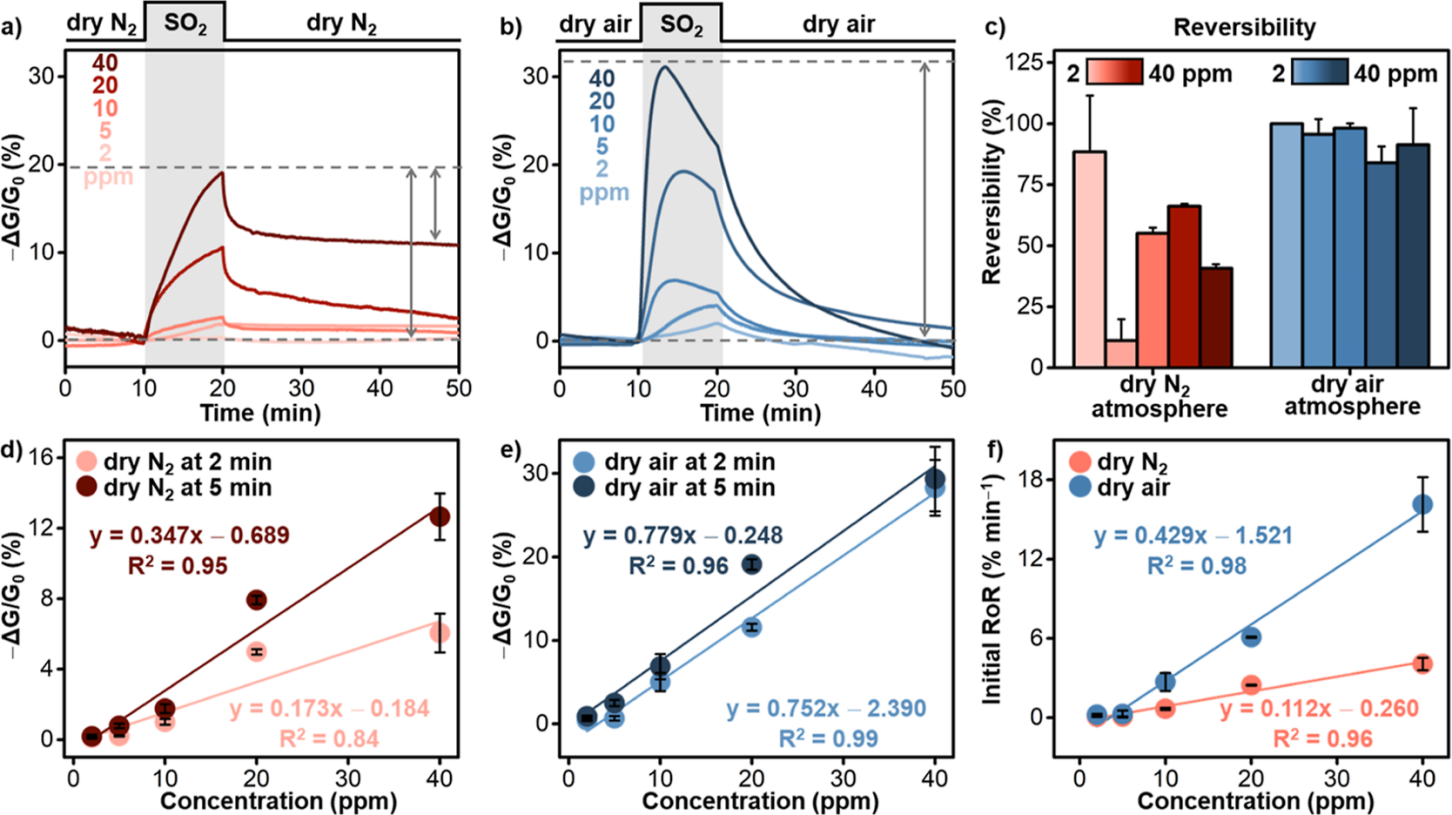
Sensing responses of DC-103 at 2, 5, 10, 20, and 40 ppm of SO_2_ in (a) dry N_2_ and (b) dry air. (c) Bar graph comparing reversibility of responses toward different concentrations of SO_2_ in nitrogen and air atmospheres. Sensing response vs concentration of SO_2_ at 2 and 5 min of exposure in (d) dry nitrogen and (e) dry air. (f) Initial rate of response (RoR) computed as the slope of the first minute of exposure vs concentration of SO_2_ in dry N_2_ and air atmospheres.

We compared the reversibility of response, detection limits (LODs) of DC-103, and initial rates of response (RoR) toward SO_2_ in dry N_2_ and atmospheric air conditions. While the sensing responses were irreversible or only partially reversible across different SO_2_ concentrations in dry N_2_ (Figure 2c), they showed excellent recovery in dry air (averaged around 100% for the 2–40 ppm of SO_2_ concentration range), suggesting a possible role of air in enhancing recovery.^60^ Plotting the concentration-dependent magnitudes of response at 2 and 5 min of exposure to SO_2_ displayed linear relationships (Figure 2d,e), allowing for the calculation of theoretical LODs upon 5 min of exposure with low values of 220 ± 12 ppb in dry N_2_ atmosphere and 57 ± 12 ppb in dry air atmosphere (Figures S36 and S37). More accurately, SO_2_ sensing experiments using DC-103 at 50, 100, and 250 ppb in dry air (Figure S38) yielded theoretical LOD values of 2.2 ± 0.5 ppb and 2.3 ± 0.5 ppb after 10 min of exposure using fifth order polynomial and noise-based 3σ LOD calculation methods, respectively (Figure S39). Notably, DC-103 showed reliable responses after only 6 s of SO_2_ exposure, with linear, concentration-dependent trends at several time points within the first minute of exposure (Figures 2f and S40-S42). The greater initial RoR in air (slope = 0.429% min^–1^ ppm^–1^) compared to N_2_ (slope = 0.112% min^–1^ ppm^–1^) can be attributed to varying electronic transductions upon O_2_ doping^61^ or a higher SO_2_ adsorption rate in the presence of O_2_ from air.^62^ With LOD values lower than the recommended PEL limits and rapid detection capabilities, DC-103 serves as a promising material for the rapid tracking of SO_2_ concentrations in ambient atmosphere before exceeding hazardous levels that may cause serious health and environmental impacts.

Prompted by the stability and enhanced reversibility of DC-103 responses toward SO_2_ in air, we sought to study its sensing response features in humid environments. Based on prior studies,^63–69^ we did not expect oxidation of SO_2_ to sulfur trioxide (SO_3_) or sulfuric acid (H_2_SO_4_) in air or under low-humidity conditions on the time scale of our experiments. At higher humidity levels, however, the possibility of SO_2_ solvation in water vapor and the subsequent formation of sulfurous acid (H_2_SO_3_) increases,^70,71^ which may lead to differences in sensing trends or mechanisms (details in Section S6.2). Remarkably, even at a high RH of 98%, where the concentration of water vapor molecules is as high as ∼26,000 ppm, at least 650 fold greater than the concentration of SO_2_, the sensing performance of DC-103 remained consistent (Figures 3a and S43). We noted at least six distinct features of the sensing response that characterize the outstanding performance of DC-103 as an SO_2_ sensor. First, DC-103 sensor devices maintained a concentration-dependent response in 98% RH humid air (Figure 3b), from which we calculated the theoretical LOD to be as low as 3 ppb upon 5 min of exposure to SO_2_ (Figure S44) with an initial RoR capable of SO_2_ detection within 6 s of exposure (Figures S45–S46). Response and recovery times of DC-103 at different SO_2_ concentrations under different conditions are summarized in Tables S4 and S5, respectively. Second, DC-103 sensing responses showed recyclability upon background gas purge with retention of response magnitude (around 50%) for up to 5 exposure-recovery cycles, even at a low SO_2_ concentration of 10 ppm (Figures 3c and S47). These results highlighted the promising reusability of the same sensor device across multiple exposures toward SO_2_ in humid air, unlike other environments (Figures S48–S49). Third, we noted that while the magnitude of sensing responses of DC-103 across varying humidity levels (0–98% RH) in N_2_ increased gradually from 40 to 80% upon exposure to 10 ppm of SO_2_ across increasing humidity levels, the normalized response remained consistent at around 50% with air as the background gas (Figures 3d and S50 and S51). Fourth, we observed similar enhanced performance metrics in humid air at 5 ppm of SO_2_, the PEL level established by OSHA, even when the concentration of water molecules was 5200 fold greater (with a 26,000 ppm concentration at 98% RH), in which the magnitude of normalized sensing response (around 20%) remained constant across varying RH levels (Figures S52 and S53). Fifth, the long-term stability (upon storage for 4 months at ambient room conditions) and high or low temperature storage stability (upon storage at 50 °C or −20 °C for 24 h) of DC-103 devices produced similar sensing performance toward 40 ppm of SO_2_ in dry air and 98% RH humid air environments (Figures S54–S56). Sixth, though the sensing responses of DC-103 in humid N_2_ environments showed no reversibility after exposure to 10 ppm of SO_2_, responses in humid air demonstrated reversibility percentages reaching up to 90% (Figure 3e). We attributed this difference in reversibility to the possible variation in SO_2_ mechanisms of interaction with DC-103 in air, allowing for weaker and more reversible binding (more details on mechanistic insights are in the section titled “Spectroscopic Insights into Interactions between DC-103 and SO_2_”).

**Figure 3:**
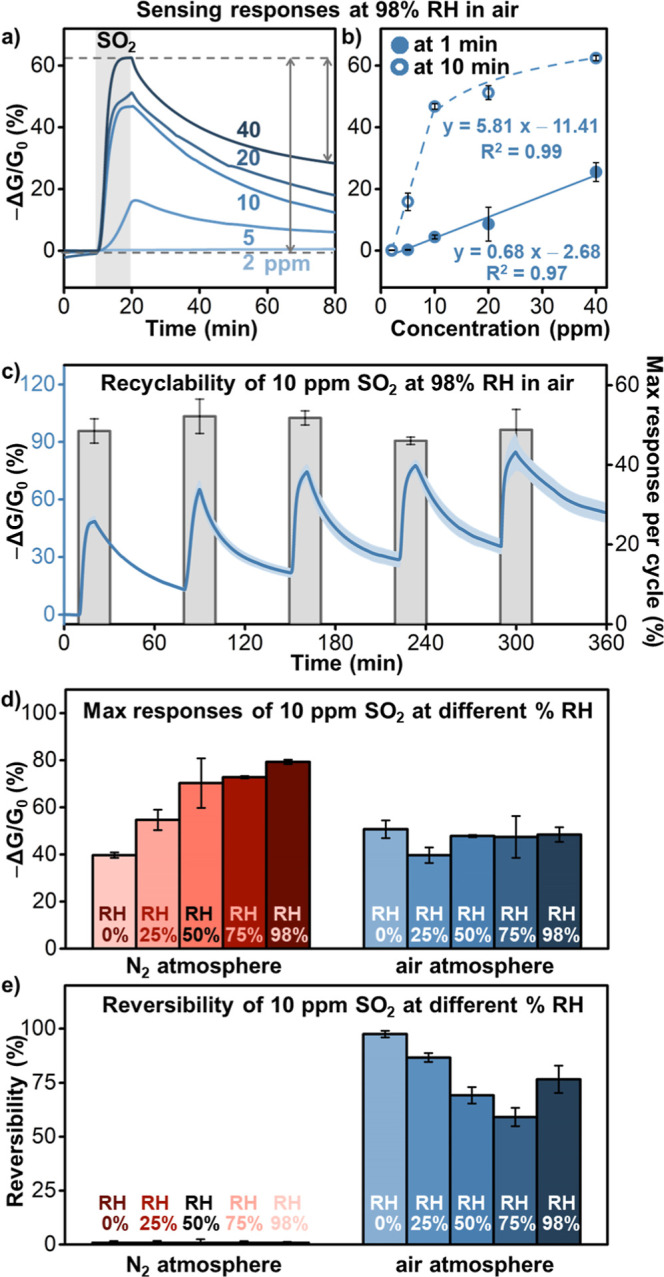
(a) Sensing responses of DC-103 at 2, 5, 10, 20, and 40 ppm of SO_2_ in air at 98% RH and (b) linear concentration-dependent response at 98% RH in air at 1 and 10 min of exposure to SO_2_. (c) Recyclability of response at 10 ppm of SO_2_ for up to 5 times upon exposure for 10 min, followed by recovery for 60 min. The blue shaded region of the sensing curve represents the standard deviation from 3 chemiresistive devices. (d) Maximum values and (e) reversibility of sensing responses toward 10 ppm of SO_2_ at different % RH in nitrogen and air atmospheres. Error bars of bar graphs represent standard deviation of at least 3 devices.

Benchmarking the SO_2_ detection performance of DC-103 against other reported MOFs for SO_2_ sensing in varying conditions (Table S6 and Section S6.3) demonstrated remarkable consistency and stability of response, particularly at saturated humidity in air. In contrast, many other sensors lost sensitivity under high humidity, exhibited higher LODs, or required hydrophobic coatings to maintain performance (Table S6).^52–54,72^ Collectively, these results highlight the superior performance of DC-103 compared to other MOF-based SO_2_ sensors, attributed to its stable response magnitude upon exposure to SO_2_ across varying humidity levels, even at concentrations up to 5200 folds greater than the analyte concentration, in N_2_ and in the presence of interferences in air.

### 2.4 Spectroscopic Insights into Interactions between DC-103 and SO_2_

To gain insight into material–analyte interactions, we performed in situ and ex situ spectroscopic investigations of DC-103 upon exposure to SO_2_ under various conditions. We collected in situ diffuse reflectance infrared Fourier transform spectroscopy (DRIFTS) traces at several time points by exposing a composite of DC-103 with potassium bromide (KBr) to 400 ppm of SO_2_ in both N_2_ and air atmospheres (see Section S7.1, Figures 4a and S57–S60). The hygroscopic nature of KBr rendered DRIFTS analysis under humid conditions inaccessible.^73^ We complemented these spectra with ex situ PXRD and XPS analyses after exposing DC-103 samples to 40 ppm of SO_2_ for 3 h in dry N_2_, dry air, and 98% RH humid air (Figures 4b-d and S61–S64). While PXRD confirmed minimal changes in crystallinity and packing structure after SO_2_ exposure, DRIFTS and XPS analyses revealed key insights into interactions between SO_2_ and DC-103, as well as differences in material–analyte interactions across varying environments.

**Figure 4:**
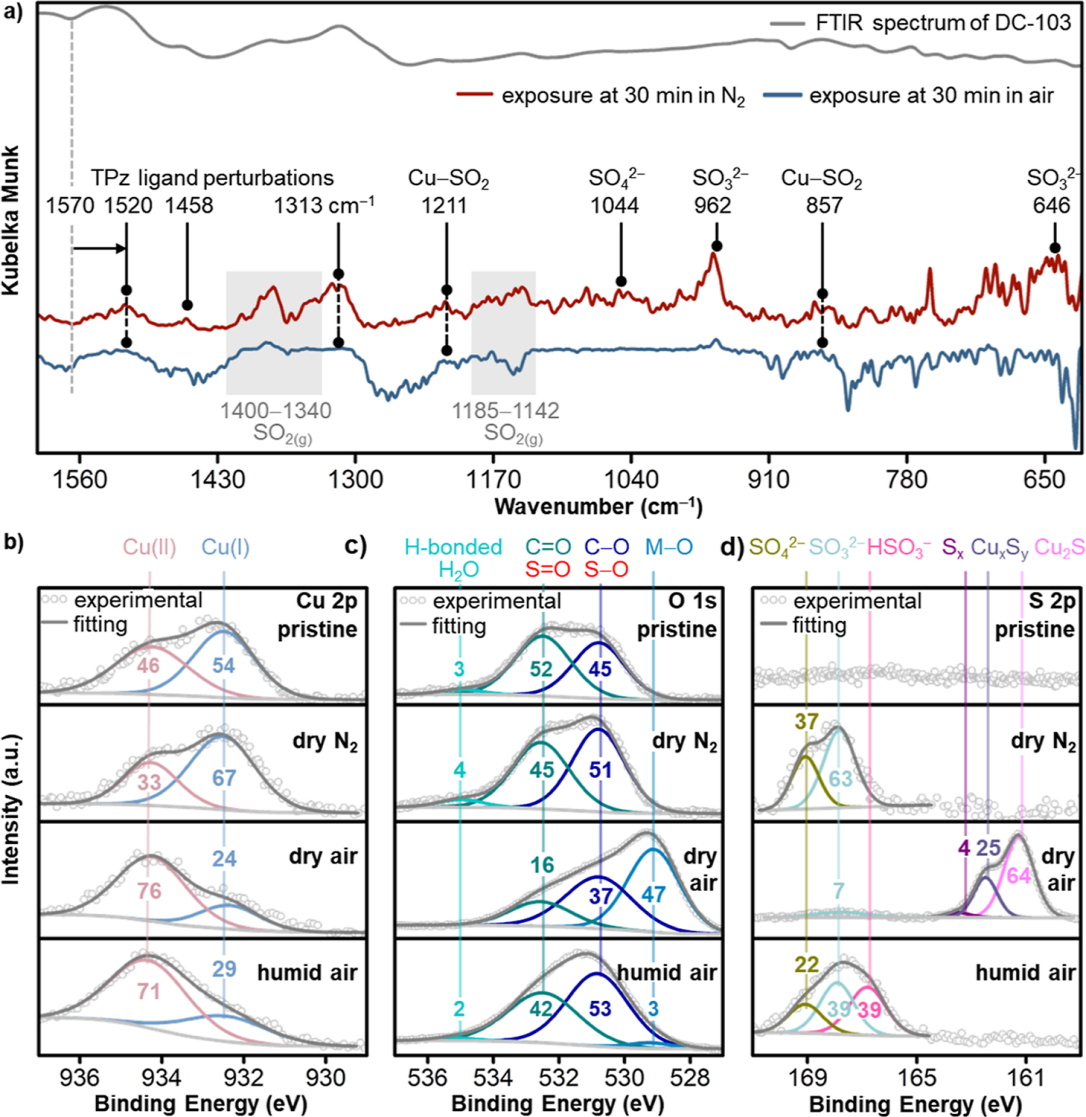
(a) DRIFTS difference spectra of DC-103 upon exposure to 400 ppm of SO_2_ in dry nitrogen and dry air atmospheres. (b) Cu 2p, (c) O 1s, and (d) S 2p high-resolution XPS spectra of DC-103 before and after a 3 h exposure to 40 ppm of SO_2_ in dry N_2_, dry air, and 98% RH humid air.

In N_2_ atmosphere, DRIFTS difference spectra displayed characteristic gaseous SO_2_ peaks at 1400–1340 and 1185–1142 cm^–1^,^74,75^ along with additional bands at 1211 and 857 cm^–1^. We assigned these bands to S–O stretching vibrations of SO_2_ molecules coordinated to copper ions (Cu–SO_2_), with different binding configurations through sulfur and oxygen atoms.^75,76^ Since the gaseous SO_2_ signals were relatively weak at 400 ppm, we also collected spectra under 1% SO_2_ exposure (Figures S57–S60), which revealed more distinct gaseous SO_2_ peaks and confirmed the similarity of the difference spectra. Additionally, we observed a peak shift, manifested as a decrease at 1570 cm^–1^ and a corresponding increase at 1520 cm^–1^, as well as two newly appearing peaks at 1458 and 1313 cm^–1^, which we attributed to perturbations within the TPz ligands of the framework upon the exposure of DC-103 to SO_2_ in N_2_ (Figure 4a).^77^ We further identified the formation of sulfite (SO_3_^2–^) species, evidenced by DRIFTS bands at 962 and 646 cm^–1^ and an S 2p XPS peak at 167.8 eV, as well as sulfate (SO_4_^2–^) species, characterized by a band at 1044 cm^–1^ and an XPS peak at 169.0 eV.^75,78–81^ The oxidation of SO_2_ into these sulfurous species was accompanied by a partial reduction of copper ions, reflected in a decrease in the Cu­(II)/Cu­(I) ratio from 46:54 to 33:67 (Figures 4b and S62).^54^ Although the newly formed S=O and S–O species, which typically appear between 530 and 533 eV,^82,83^ overlap with the C=O and C–O of the O 1s region (Figures 4c and S62), the minimal sulfur content (between 0.2 and 1.0%) determined from XPS (Table S7) renders the effect of S=O and S–O species negligible on the overall C=O/C–O ratio. The results under N_2_ atmosphere, namely the formation of SO_3_^2–^ and SO_4_^2–^ species alongside the reduction of copper ions, are consistent with our previously reported computational simulations of SO_2_ interactions with Cu_3_(HHTP)_2_, which contains structurally analogous copper bis­(dioxolene) active sites.^54^ The presence of water molecules within the DC-103 framework, confirmed by TGA and elemental analysis (Figure S19 and Table S2), facilitates the oxidation of SO_2_. While the interaction of SO_2_ with water can yield H_2_SO_3_, the redox reaction of SO_2_ with water and Cu­(II) produces Cu­(I) and H_2_SO_4_.^54^

In dry air atmosphere, we observed a decrease in the broad electronic absorbance (BEA) in the DRIFTS spectra, resulting in an overall reduction in band intensity (Figures 4a and S58). Nonetheless, spectral features similar to those observed in N_2_ environment emerged, including bands associated with gaseous and adsorbed SO_2_ (at 1400–1340, 1185–1142, 1211, and 857 cm^–1^) and perturbations in the TPz ligands (shift from 1570 to 1520 cm^–1^, 1458, and 1313 cm^–1^). However, in contrast to the N_2_ atmosphere, where SO_2_ oxidation and Cu­(II) reduction predominated, exposure to dry air led to the reduction of SO_2_ into polysulfide species (4% S_*x*_ at 163.4 eV),^84^ copper-deficient nonstoichiometric sulfides (25% Cu_*x*_S_*y*_ at 162.3 eV),^85^ and copper­(I) sulfide (Cu_2_S at 161.2 eV).^86^ These species could not be detected by DRIFTS due to the low signal-to-noise ratio at wavenumbers below 620 cm^–1^,^87^ see Figures S58 and S60. Concomitant with this reduction, we observed the reduction of the ligand with a decrease in C=O to C–O ratio as well as the oxidation of copper bridging ions to a 76:24 Cu­(II)/Cu­(I) ratio (Figures 4b and S63) and the emergence of an additional O 1s peak at 529.0 eV, attributed to lattice oxygen in copper oxide (M–O) species (Figures 4c and S63).^88^ Mechanistically, these results resemble the interaction of cuprous oxide (Cu_2_O) with SO_2_ in air,^62^ where the SO_2_ is adsorbed onto copper centers forming Cu–SO_2_ complexes. Upon the oxidation of Cu­(I) species to Cu­(II), C=O bonds of the ligand and SO_2_ can be reduced to C–O bonds and sulfide species, respectively.

XPS spectra of DC-103 exposed to SO_2_ in 98% RH humid air revealed oxidation of the copper bridging ions, with the Cu­(II)/Cu­(I) ratio increasing from 46:54 to 71:29 (Figures 4b and S64). We also noted the formation of oxidized sulfurous species, including SO_3_^2–^ (39%), SO_4_^2–^ (22%), and HSO_3_^–^ (bisulfite at 166.7 eV, 39%).^89^ We attribute the formation of these species to the initial solvation of SO_2_ in the excess water vapor of the saturated humid atmosphere, leading to the formation of H_2_SO_3_. The dissociation of H_2_SO_3_ to form HSO_3_^–^ and SO_3_^2–^ is consistent with the S 2p XPS peaks at 166.7 eV^89^ and 167.8 eV, respectively (Figures 4c and S64).^89^ The additional peak at 169.0 eV, attributed to SO_4_^2–^ (Figures 4c and S64), can be explained either by the alternative oxidation of SO_2_ in moisture to form H_2_SO_4_, which dissociates as H_2_SO_4_ → 2H^+^ + SO_4_^2–^,^54^ or by the oxidation of SO_3_^2–^ in air (2SO_3_^2–^ + O_2_ → 2SO_4_^2–^).^62,89^ In this proposal, Cu­(I) centers can be oxidized to Cu­(II) in the presence of O_2_ from air and protons generated from the dissociation of H_2_SO_3_ or H_2_SO_4_, according to the following equation: 4Cu^+^ + O_2_ + 4H^+^ → 4Cu^2+^ + 2H_2_O.^90^ However, it is important to note that the above-mentioned spectroscopic measurements were performed at SO_2_ concentrations and exposure times that exceed the experimental sensing conditions. This approach maximized spectroscopic resolution with a high signal-to-noise ratio, but did not necessarily reflect the recovery trends observed at low ppm-level concentrations used in sensing.

To better relate spectroscopic insights to sensing responses, we conducted a strategic study under equivalent 10 min exposure conditions toward 40 and 400 ppm of SO_2_ in dry N_2_ and dry air, followed by a 30 min purge in background gas (Figures S65–S70). Sensing experiments at 400 ppm in both atmospheres showed reduced reversibility upon recovery, compared to exposure at 40 ppm (Figure S65). The limited reversibility of the sensing response at both SO_2_ concentrations in dry N_2_ is confirmed spectroscopically even after 10 min of exposure, consistent with the sensing experiments (Figures S66–S68 and Table S7). Conversely, the reversibility toward 40 ppm of SO_2_ in dry air observed in sensing is consistent with the DRIFTS spectra upon recovery (Figures S66 and S67) and in the absence of distinguishable sulfurous species in its XPS spectra (Figure S69). The reversibility of the sensing response in dry air decreases significantly upon exposure to a higher SO_2_ concentration of 400 ppm, consistent with the irreversible generation of sulfurous species confirmed spectroscopically (Figures S65–S70 and Table S7). Collectively, in situ and ex situ spectroscopic analyses of DC-103 under N_2_, dry air, and humid air reveal distinct mechanistic pathways for SO_2_ interactions that govern differences in reversibility and recyclability while maintaining consistent sensing performance across humidity levels.

### 2.5 Computational Characterization of MOF–Analyte Interactions

The DC-103–SO_2_ interactions identified and characterized by MD under varying conditions confirmed and supported the experimental findings (Figures S71–S80). Several snapshots of portions of the DC-103 model loaded with guest species, namely H_2_O, SO_2_, and N_2_, are shown in Figure 5a. During the system dynamics, we observed not only the strong binding of some molecules to the metal centers, including the adsorption of SO_2_ to the copper site through coordination of the S atom, but also their speciation, diffusion out of the framework, and readsorption on the exposed surfaces of the framework. We identified low levels of intermediate species, namely SO_3_^2–^ (6%), H_2_SO_3_ (at most 11%), and traces of H_2_SO_4_ (at most 2.5%). Instead, the percentage of free SO_2_ inside the MOF was between 25 and 42%, depending on the water content, and the adsorbed SO_2_ molecules were at most 20%. Visual inspection of the trajectory revealed that the intermediate species weakly interacted with the MOF walls (Figures 5b–d) and tended to diffuse toward the surface. The locations of all these species relative to Co and Cu ions were identified by analyzing trends in the radial distribution functions (RDF) over the final portion of the production trajectories (Figures S71–S73). All the Cu–S plots (bulk and surface) show a high, sharp peak at about 2.2 Å, followed by a plateau up to 4 Å, indicating no exchange with neighboring atoms and a stable coordination of the sulfur atoms to Cu. The second, lower-intensity peak corresponding to the Cu–O_SO_2__ RDF at about 3.2 Å confirms that SO_2_ is the adsorbed species. These peaks are absent or very small in the Co–S or Co–O_SO_2__ RDFs, suggesting that the Co centers are inaccessible to SO_2_ and not preferred by the guests. Unlike Cu, which had no water molecules nearby, Co appeared well solvated, as evidenced by the water-oxygen (O_H_2_O_) peak at about 1.8 Å, visible in all graphs. The Co accessibility scenario on the surface is quite different, and the RDF trends clearly show that both SO_2_ and water could be adsorbed on this ion.

**Figure 5:**
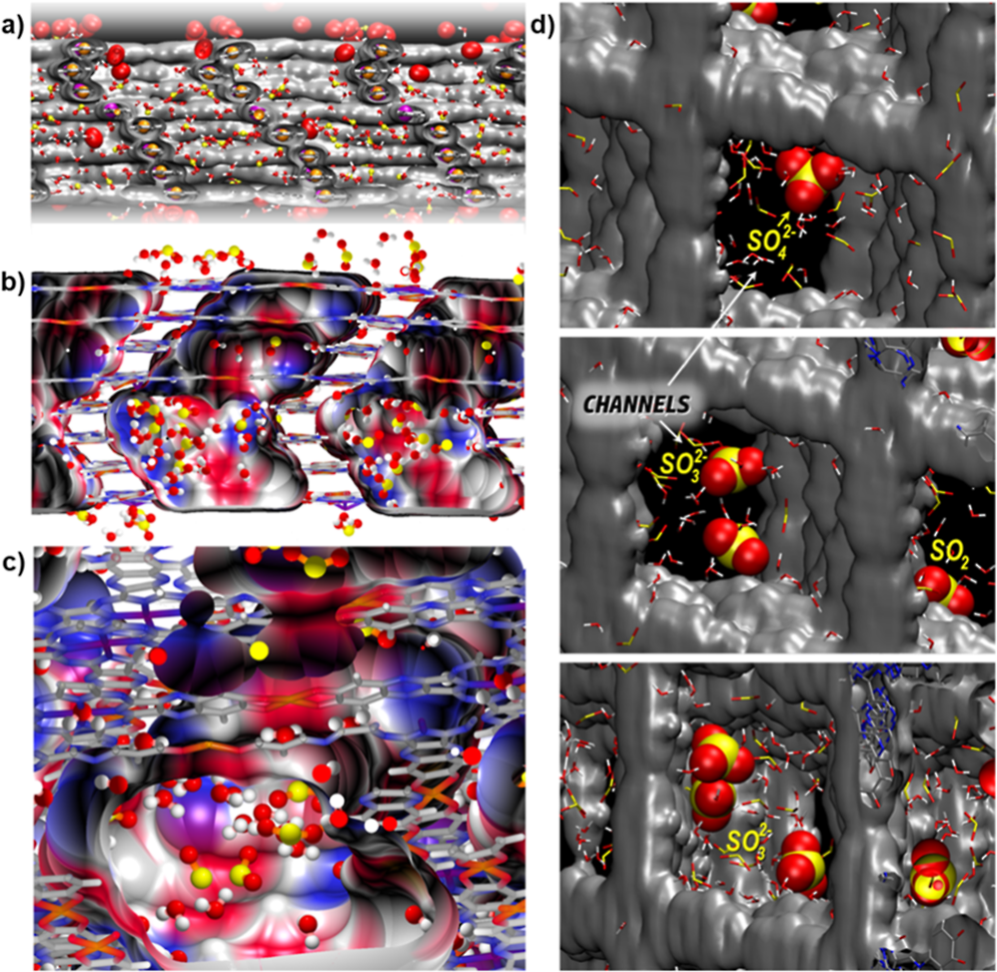
(a) Section of the channels filled with guest species, including O_2_ molecules (red spheres), which are more tendentially located at the surface and a few inside the channels. (b) Side view of the MOF channels filled with SO_2_ and water. Some of the molecules migrated to the surfaces and remained adsorbed there. (c) Zoomed view of one of the channels containing water and SO_2_ species. Color code: C gray, O red, N blue, H white, Co purple, and Cu orange. (d) Representative sections of a few channels (small portions-MOF structure is represented by a gray solvent-accessible surface) filled with water (O red and H white stick model), and SO_2_ (S yellow and O red stick model) molecules. A few reaction products, namely SO_3_^2–^ and SO_4_^2–^ (VDW representation), and free/adsorbed SO_2_ species are shown.

To further confirm these observations, we performed local DFT relaxations on MOF morphologies extracted from MD snapshots and on adsorption configurations inferred from the dynamic properties, considering both inactive and reactive pathways. We confirmed the reduced tendency of SO_2_ to interact with bulk Co sites (Figure S79), which are buried between the layers and, therefore, primarily accessible to smaller molecules such as O_2_ and H_2_O. However, we verified that even interactions between Co sites and the smaller species can induce significant changes in the system morphology (Figure S78) at very low concentrations, confirming the dynamic properties characterized by strong structural rearrangements in response to the guest environment. DFT also confirmed the tendency of SO_2_ to occupy regions near the Cu site, either in the form of undissociated SO_2_ or as oxidized species such as SO_3_^2–^ or SO_4_^2–^. This observation is consistent with previous reports on the closely related Cu_3_(HHTP)_2_ system, which features analogous copper bis­(dioxolene) active sites.^54^ The associated proton transfer to the oxygen atoms of the metal bis­(dioxolene) group leads to pronounced structural rearrangements (Figure S80), including reduction of Cu­(II) to Cu­(I). Such effects may be directly linked to the poor recovery of the material observed under selected conditions. Further details on these aspects are provided in Section S8. In an N_2_ atmosphere, the most noticeable effect in the RDF (Figure S72) was the disappearance of the Co-water peaks at short distances (1.7–3.2 Å), suggesting that the presence of N_2_ restricted the mobility of the internal water, effectively confining and separating the solvent molecules from the Co sites. Additionally, the Cu­(II)/Cu­(I) ratio shifted to 33:67, indicating a decrease in SO_2_ adsorption on these metal sites (by up to 25%) relative to the case in which air substitutes N_2_. The guest confinement effect due to N_2_ within the MOF was further confirmed by analyzing the solvation of the TPz ligands, as reflected in their hydrogen-bonding interactions with water. Indeed, this percentage was higher in N_2_ (38%) than in air (28%), indicating slight retention (Figures S73–S75).

The results of the system dynamics analysis with added O_2_ agree with the experimental scenario. Indeed, O_2_ could be adsorbed on Cu in the bulk at very low humidity (a higher O_2_ percentage of 34% remained in the material, compared with 15% at high humidity), reacted with the internal species producing a variety of water and sulfur intermediates, but tended to diffuse to the surface and outside the MOF. The SO_2_ molecules strongly adsorbed were around 10%, which is half the number observed in the absence of O_2_. The Cu­(II)/Cu­(I) ratio changed to 75:35, which is very close to the experimental estimate (Figure S63). Overall, the computational results suggested that Cu was the main SO_2_ adsorption site on the MOF, with Co playing an important role in binding water under humid conditions. That confinement led to irreversible responses in experiments with N_2_ background gas.

### 2.6 Testing the Response Robustness of DC-103 toward Other Toxic Gases

To evaluate the robust detection and relative signal enhancement of DC-103 toward various gases, we assessed its sensing response toward hydrogen sulfide (H_2_S), nitric oxide (NO), nitrogen dioxide (NO_2_), and ammonia (NH_3_), under varying environmental conditions: dry N_2_ (0% RH), humid N_2_ (98% RH), dry air (0% RH), and humid air (98% RH), Figure 6a. DC-103 exhibited stable, humidity-tolerant, and reusable sensing toward H_2_S over at least 12 cycles, even at 98% RH humid air, though with a larger magnitude of response (40%) upon the first exposure, followed by a diminished, but consistent response of 20% in the following cycles (Figures S81 and S82). The comparable stability in DC-103 responses toward both SO_2_ and H_2_S is likely due to the similarity in underlying redox-type interactions occurring between the reducing sulfurous gases and the framework.^91^ Nonetheless, we focus here on SO_2_ detection given the low LOD achievable with DC-103 relative to prior MOF/COF systems (Table S6), whereas H_2_S detection is already well-established in this class of materials, with reported LODs reaching 5 ppb.^92,93^

**Figure 6:**
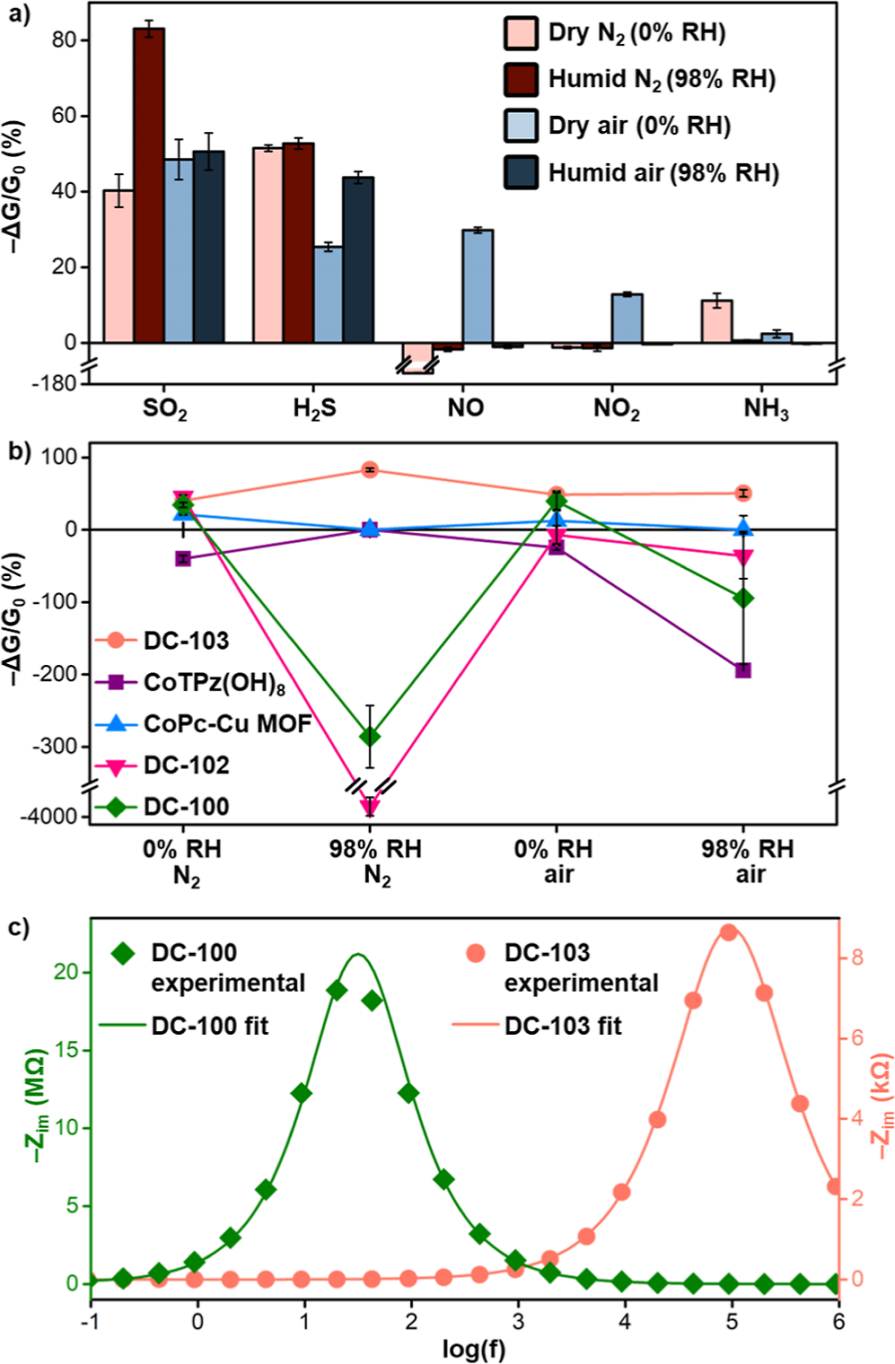
Graphs showing the magnitude of sensing response (a) of DC-103 toward 10 ppm of various analyte gases (SO_2_, H_2_S, NO, NO_2_, NH_3_) and (b) of various materials toward 10 ppm of SO_2_ after 10 min of exposure to the analyte gas in dry N_2_, 98% RH humid N_2_, dry air, and 98% RH humid air environments. Error bars correspond to the standard deviation from three devices. (c) EIS plots of the negative imaginary impedance component (−*Z*_im_) as a function of logarithmic frequency for DC-103 and DC-100 after exposure to 40 ppm of SO_2_ in dry N_2_ for 10 min.

Toward nitrogenous gases, however, DC-103 displayed differences in magnitude and directionality of responses across varying environments. For example, the response of DC-103 toward NO and NO_2_ is almost absent in humid N_2_ and air atmospheres. The response is also inconsistent in complex environments and is greatly impacted by air interference and high humidity (Figures S83 and S84). For NH_3_, though with the same directionality, we observed a large decrease in response magnitude in the presence of air interference and in humid environments (Figure S85). Exposure of DC-103 devices toward 40–50 ppm of other analyte gases, such as carbon monoxide (CO), carbon dioxide (CO_2_), methane (CH_4_), and hydrogen (H_2_) in dry air did not elicit any distinguishable chemiresistive response (Figure S86). Hence, DC-103 shows promise as an interference-tolerant sensor for sulfurous gases, especially over nitrogenous gases. While differentiation of SO_2_ and H_2_S can be challenging to achieve with DC-103 alone due to cross-reactivity, recent reliance on MOF arrays and machine learning algorithms constitutes a promising strategy for enhanced selectivity and differentiation within this class of gases.^94^

### 2.7 Unique Stability of DC-103 Compared to Analogs

To assess the unique stability of DC-103 for reliable SO_2_ and H_2_S detection, we examined the properties and sensing performance of structurally analogous and control structures under varying environments (Figures 6b and S87). We first evaluated the performance of the TPz-based MOF structure by analyzing the sensing responses of the CoTPz­(OH)_8_ monomer as the active material in chemiresistive devices. These experiments suggested that the sensing response of the building block itself was mostly unstable in air and humid conditions, with variable and negative responses (Figures S88 and S89). In contrast, upon incorporation into a copper-based highly crystalline MOF structure, i.e. DC-103, the material exhibited stable and positive responses (Figures 2a,b, and 3a). We also examined a compositionally similar CoPc-Cu-MOF analog, based on phthalocyanine rather than TPz moieties,^95^ which suggested that the MOF combination of a cobalt-centered macrocycle with copper-bridging units is an effective molecular design strategy for SO_2_ detection.^96^ Although CoPc-Cu-MOF showed comparable SO_2_ and H_2_S sensing performance in both dry N_2_ and dry air environments, the devices lost stability under high humidity, leading to short-circuiting even without exposure to any gas (Figures S90–S92). These results confirmed the importance of the TPz-based framework structure of DC-103 in forming sensing response that are functional under humid conditions, owing to the dominance of its electrical conductivity over proton conductivity even at saturated humidity.

To probe the role of metal centers and morphology of TPz-based MOFs, we performed sensing experiments on a TPz-MOF analog DC-102 (H_2_TPz-Cu-MOF), composed of metal-free TPz ligands linked with copper ions.^55^ The absence of the cobalt center from within the TPz framework resulted in unstable currents and inconsistent magnitudes and directionalities of response toward SO_2_ and H_2_S across different environments (Figures S93–S94). Similarly, we tested the sensing capability of DC-100 (NiTPz-Cu-MOF), another cMOF analog with a nickel-centered TPz ligand linked with copper, which is structurally analogous to DC-103 and DC-102.^55^ Though responses to SO_2_ in both dry atmospheres (N_2_ and air) were comparable, we noted poor stability of the chemiresistive devices in humid environments, leading to reversed directionalities and variable response magnitudes (Figure S95). Toward H_2_S, although DC-100 showed a similar humidity-dependent trend in its sensing response, its response under air interference was significantly diminished relative to that in N_2_ (Figure S96). These results further highlight the importance of the enhanced crystallinity and larger crystallite size of the cobalt-centered DC-103 on its robust performance under varying conditions.

To understand the differences that may account for the varying stability of sensing response among the DC-100 MOF series, we considered factors related to the molecular design and material properties, as well as possible differences in the fabricated devices of the different analogs. At the molecular level, we noted that the high electrical conductivity of DC-103, exceeding its proton conductivity (Figure S97), in contrast with sensing properties of DC-100, which possesses an electrical conductivity of 2.6 × 10^–6^ S cm^–1^ and a proton conductivity of 1.1 × 10^–5^ S cm^–1^ (303 K, 98% RH). We hypothesized that under saturated humid environments, proton conductivity in DC-100 likely dominates, yielding large variations in the magnitudes and directionalities of responses in dry (20%) and wet (−300%) environments (Figure S95). This hypothesis was supported by electrochemical impedance spectroscopy (EIS) measurements performed under varying humidity levels and upon SO_2_ exposure (Section S11). The results indicate the dominance of electronic charge transfer under dry conditions and the emergence of Warburg-type diffusion at high humidity, for both DC-103 and DC-100 (Figures S98–S102).^97^ Notably, these changes had minimal impact on the range of real and imaginary impedance (*Z*_re_ and −*Z*_im_, respectively) values for DC-103, whereas DC-100 exhibited variations spanning 4–5 orders of magnitude under AC bias, consistent with trends in the *I*–*V* curves observed under DC bias (Figures S31 and S99–S103). Upon SO_2_ exposure under dry environments, the radii of the Nyquist semicircles increased (Figures S104–S109), indicating an increase in resistance, in agreement with the chemiresistive sensing responses (see Section S11.2.1 for details).^97,98^ A key distinction observed between the two materials was found in the frequency range at which the impedance peaks occur. DC-103 exhibited peaks at higher frequencies, characteristic of charge-transfer processes dominated by electronic effects, whereas DC-100 showed peaks at lower frequencies, indicative of mass transport that may include contributions from ionic diffusion (Figure 6c),^97,99^ further supporting our hypothesis. Under humid conditions (Figures S110 and S111), DC-103 displayed an evolution toward enhanced diffusion or capacitive properties upon SO_2_ exposure, while DC-100 remained dominated by diffusion-controlled processes (see Section S11.2.2 for details).^97^

At the nanoscale, we compared the colloidal stability, crystal size distribution, and zeta potential values of the material suspensions, from which we observed that the DC-103 suspension remained colloidally stable for at least 5 months, while those of DC-102 and DC-100 showed sedimentation or agglomeration of MOF particles (Figures S112–S115). Optical and SEM images of their corresponding dropcasted IDEs (Figures S116–S118) showed the uniformly distributed thin film of DC-103 across the deposited portion of the IDE, unlike the images of DC-102 and DC-100, which showed agglomerated crystals or the presence of cracks in the deposited thin film. Together, these comparisons suggest that, while each control material showed environment-dependent variations in magnitude and directionality of sensing response, DC-103 stood out for its reliable, reproducible responses to 10 ppm of SO_2_ and H_2_S across all tested conditions. We attributed the enhanced stability of DC-103 compared to its analogous counterparts to its enhanced crystallinity and larger crystallite sizes, which enable appreciable electrical conductivity that dominates over its proton conductivity under various conditions. These properties are complemented by the ability of its suspension to form a uniform MOF thin film in the fabricated devices, which is not the case for the other TPz-MOFs shown in this work or other cMOFs reported in the literature.^95,100,101^

## 3 Conclusion

In conclusion, this work demonstrates the synthesis and sensing capabilities of DC-103, a novel, highly crystalline, and intrinsically conductive TPz-based MOF. As a chemiresistive SO_2_ sensor, DC-103 exhibits robust, reversible, and humidity-tolerant responses across a wide range of environmental conditions, addressing a critical challenge in real-world gas sensing settings, where atmospheric humidity fluctuates regularly. Its high sensitivity toward SO_2_, with theoretical LODs as low as 2.2 ppb, rivaling other MOF-based SO_2_ sensors (Table S6), and its consistent signal output across varying relative humidities (0–98% RH) make it a competitive platform for trace-level SO_2_ monitoring. Notably, the sensor exhibits partial to full reversibility depending on the environment, with enhanced reversibility in air compared to nitrogen, and remains reusable for at least five cycles under high humidity conditions—an appealing trait for repeated exposure. Spectroscopic and computational characterizations of the material–analyte interactions reveal the formation of various oxidized or reduced sulfurous species across different environments and suggest the critical role of O_2_ during exposure in oxidizing Cu­(I) to Cu­(II), promoting reversible responses to SO_2_ in air.

Beyond its performance metrics, this work underscores the impact of targeted properties in developing next-generation cMOFs that overcome the limitations of existing materials. The improved humidity tolerance and sensing reversibility in air of DC-103, relative to structurally analogous frameworks such as DC-102, DC-100, and CoPc-Cu-MOF suggest that subtle differences in coordination environment, as well as control over electrical conductivity and crystallinity of the MOF material, may impact charge transport, redox stability, resistance to humidity interferences, as well as host–guest interactions, leading to tailored sensing properties. The computational results show that each unique component of DC-103 plays a key role in its robust SO_2_ detection performance. While the Cu ions and surface Co ions serve as the main active SO_2_ interaction sites, Co ions in the bulk MOF and N atoms within the TPz structure interact with H_2_O molecules (Figures S71–S73). In addition, DC-103 demonstrates a high affinity toward SO_2_, even in the presence of H_2_O (Figure S76). However, the coexistence of H_2_O, potentially in combination with O_2_, can facilitate reactive pathways that lead to the formation of various oxidized sulfur-containing species. These processes may trigger structural rearrangement within the MOF, accompanied by proton migration and changes in the Cu-oxidation state, thereby affecting the reversibility properties (Figures S78–S80). Complementary experimental results from EIS sensing and suspension properties highlight the superior crystallinity, crystallite size, and conductivity, as well as the dominance of electronic charge-transfer mechanisms in DC-103, compared to its analogs that do not demonstrate robust performance (Figure 6c). These key structural interpretations provide strong evidence for the consistent SO_2_ detection even under high humidity.

Future studies aimed at deconvoluting the effects of crystal structure at the molecular level, distinct from those specific to crystal size and morphology, could yield deeper insights into the mechanisms governing signal transduction and gas recognition in the presence of interferences. The consistent and reliable response of DC-103 to both SO_2_ and H_2_S, however, poses a potential limitation for differentiating between these sulfurous gases. This issue can be overcome by adopting an established strategy of incorporating the sensor into a chemiresistive array of multiple sensors, each responding differently toward these gases. This combination enables signal differentiation and the deconvolution of binary mixtures^94^ through machine learning analyses. Collectively, this work not only introduces a promising sensor material for detecting sulfurous gases in realistic conditions, but also contributes to a broader understanding of how the rational design of conductive frameworks can enable next-generation sensing technologies suitable for environmental monitoring, occupational safety, and public health applications.

## Supplementary Material



## Notes

Details and supplementary figures of experimental methods, synthesis optimization, characterization (PXRD, SEM, TEM, ATR-FTIR, conductivity, TGA, BET, EPR), gas sensing experiments, in situ DRIFTS, and post-exposure XPS spectra (PDF)

## References

[1] McLinden C. A., Fioletov V., Shephard M. W., Krotkov N., Li C., Martin R. V., Moran M. D., Joiner J. (2016). Space-based detection
of missing sulfur dioxide sources of global air pollution. Nat. Geosci..

[2] Zhong Q., Shen H., Yun X., Chen Y., Ren Y. a., Xu H., Shen G., Du W., Meng J., Li W. (2020). Global sulfur dioxide emissions and the driving forces. Environ. Sci. Technol..

[3] Perraud V., Horne J. R., Martinez A. S., Kalinowski J., Meinardi S., Dawson M. L., Wingen L. M., Dabdub D., Blake D. R., Gerber R. B. (2015). The future of airborne
sulfur-containing particles in the absence of fossil fuel sulfur dioxide
emissions. Proc. Natl. Acad. Sci. U.S.A..

[4] Ruiz-López M.
F., Martins-Costa M. T., Anglada J. M., Francisco J. S. (2019). A new mechanism
of acid rain generation from HOSO at the air–water interface. J. Am. Chem. Soc..

[5] Cape J. N., Fowler D., Davison A. (2003). Ecological effects of sulfur dioxide,
fluorides, and minor air pollutants: recent trends and research needs. Environ. Int..

[6] Chen Z., Liu N., Tang H., Gao X., Zhang Y., Kan H., Deng F., Zhao B., Zeng X., Sun Y. (2022). Health effects of exposure
to sulfur dioxide, nitrogen dioxide, ozone,
and carbon monoxide between 1980 and 2019: A systematic review and
meta-analysis. Indoor Air.

[7] Khalaf E. M., Mohammadi M. J., Sulistiyani S., Ramírez-Coronel A. A., Kiani F., Jalil A. T., Almulla A. F., Asban P., Farhadi M., Derikondi M. (2024). Effects of sulfur dioxide inhalation
on human health: a review. Rev. Environ. Health.

[8] Singh, J. ; Kaushik, R. ; Chawla, M. Hazardous Gases: Risk Assessment on the Environment and Human Health; Academic Press, 2021.

[9] Zhu Y., Toon O. B., Jensen E. J., Bardeen C. G., Mills M. J., Tolbert M. A., Yu P., Woods S. (2020). Persisting volcanic
ash particles impact stratospheric SO_2_ lifetime and aerosol
optical properties. Nat. Commun..

[10] Ohmoto H. (2020). A seawater-sulfate
origin for early Earth’s volcanic sulfur. Nat. Geosci..

[11] Macdonald B. C., Denmead O. T., White I., Melville M. D. (2004). Natural sulfur dioxide
emissions from sulfuric soils. Atmos. Environ..

[12] Muyzer G., Stams A. J. (2008). The ecology and
biotechnology of sulphate-reducing
bacteria. Nat. Rev. Microbiol..

[13] Smith S. J., Pitcher H., Wigley T. M. (2001). Global
and regional anthropogenic
sulfur dioxide emissions. Glob. Planet. Change.

[14] Fioletov V., McLinden C. A., Griffin D., Abboud I., Krotkov N., Leonard P. J., Li C., Joiner J., Theys N., Carn S. (2022). Version 2 of the global
catalogue of large anthropogenic and volcanic
SO_2_ sources and emissions derived from satellite measurements. Earth Syst. Sci. Data Discuss..

[15] Cullis C., Hirschler M. (1980). Atmospheric sulphur: natural and man-made sources. Atmos. Environ..

[16] Al
Hamadi M., Ibrahim S., Raj A. (2019). Effects of oxygen enrichment
on natural gas consumption and emissions of toxic gases (CO, Aromatics,
and SO_2_) in the Claus process. Ind.
Eng. Chem. Res..

[17] Abdul-Wahab S. A., Al-Alawi S. M., El-Zawahry A. (2002). Patterns of
SO_2_ emissions:
a refinery case study. Environ. Model. Softw..

[18] Salih M., Hamadamin R., Hama J. (2023). Emission and exposure of hydrogen
sulfide in the air from oil refinery: spatiotemporal field monitoring. Int. J. Environ. Sci. Technol..

[19] Jomantas G., Buinevičius K., Šereika J. (2025). Sulfur Emission
Dependence on Various
Factors During Biomass Combustion. Energies.

[20] Martin R. V. (2008). Satellite
remote sensing of surface air quality. Atmos.
Environ..

[21] Streets D. G., Canty T., Carmichael G. R., de Foy B., Dickerson R. R., Duncan B. N., Edwards D. P., Haynes J. A., Henze D. K., Houyoux M. R. (2013). Emissions estimation from satellite retrievals:
A review of current capability. Atmos. Environ..

[22] Yu J., Wang D., Tipparaju V. V., Tsow F., Xian X. (2021). Mitigation
of humidity interference in colorimetric sensing of gases. ACS Sens..

[23] Stevens R., Mulik J., O’Keeffe A., Krost K. (1971). Gas chromatography
of reactive sulfur gases in air at the parts-per-billion level. Anal. Chem..

[24] Wang K., Bi C., Zelenkov L., Liu X., Song M., Wang W., Makarov S., Yin W. (2024). Fluorescent sensing for the detection
and quantification of sulfur-containing gases. ACS Sens..

[25] Oppenheimer C., Francis P., Burton M., Maciejewski A., Boardman L. (1998). Remote measurement of volcanic gases by Fourier transform
infrared spectroscopy. Appl. Phys. B: Lasers
Opt..

[26] Petruci J. F. d. S., Wilk A., Cardoso A. A., Mizaikoff B. (2015). Online analysis
of H_2_S and SO_2_ via advanced mid-infrared gas
sensors. Anal. Chem..

[27] Zhang Y., Wang Y., Liu Y., Dong X., Xia H., Zhang Z., Li J. (2019). Optical H_2_S and SO_2_ sensor based on chemical conversion and
partition differential
optical absorption spectroscopy. Spectrochim.
Acta A: Mol. Biomol. Spectrosc..

[28] Gomes M. T. S., Nogueira P. S. T., Oliveira J. A. (2000). Quantification
of
CO_2_, SO_2_, NH_3_, and H_2_S
with a single coated piezoelectric quartz crystal. Sens. Actuators B: Chem..

[29] Khan M. A. H., Rao M. V., Li Q. (2019). Recent advances
in electrochemical
sensors for detecting toxic gases: NO_2_, SO_2_ and
H_2_S. Sensors.

[30] Najafi P., Ghaemi A. (2024). Chemiresistor gas sensors: Design, Challenges, and
Strategies: A comprehensive review. Chem. Eng.
J..

[31] Rossi A., Spagnoli E., Visonà A., Ahmed D., Marzocchi M., Guidi V., Fabbri B. (2024). SO_2_ Detection over a Wide
Range of Concentrations: An Exploration on MOX-Based Gas Sensors. Chemosensors.

[32] Tang H., Sacco L. N., Vollebregt S., Ye H., Fan X., Zhang G. (2020). Recent advances in 2D/nanostructured
metal sulfide-based gas sensors:
mechanisms, applications, and perspectives. J. Mater. Chem. A.

[33] Jung G., Jeong Y., Hong Y., Wu M., Hong S., Shin W., Park J., Jang D., Lee J.-H. (2020). SO_2_ gas sensing characteristics of FET-and resistor-type gas
sensors having WO_3_ as sensing material. Solid-State Electron..

[34] Khune A. S., Padghan V., Bongane R., Narwade V. N., Dole B., Ingle N. N., Tsai M.-L., Hianik T., Shirsat M. D. (2023). Highly
selective chemiresistive SO_2_ sensor based on a reduced
graphene oxide/porphyrin (rGO/TAPP) composite. J. Electron. Mater..

[35] Kumar R., Avasthi D., Kaur A. (2017). Fabrication of chemiresistive
gas
sensors based on multistep reduced graphene oxide for low parts per
million monitoring of sulfur dioxide at room temperature. Sens. Actuators B: Chem..

[36] Shinde R., Padalkar N., Sadavar S., Patil A., Kale S., Magdum V., Chitare Y., Kulkarni S., Patil U., Parale V. (2022). Lattice engineering
route for self-assembled nanohybrids
of 2D layered double hydroxide with 0D isopolyoxovanadate: Chemiresistive
SO_2_ sensor. Mater. Today Chem..

[37] Chaudhary V., Channegowda M., Ansari S. A., Rajan H. K., Kaushik A., Khanna V., Zhao Z., Furukawa H., Khosla A. (2022). Low-trace
monitoring of airborne sulphur dioxide employing SnO_2_-CNT
hybrids-based energy-efficient chemiresistor. J. Mater. Res. Technol..

[38] Nath U., Sarma M. (2023). Pyridinic dominance n-doped graphene: a potential material for SO_2_ gas detection. J. Phys. Chem. A.

[39] Zhao Z., Ma C., Xu L., Yu Z., Wang D., Jiang L., Jiang X., Gao G. (2023). Conductive
Polyaniline-Based Microwire
Arrays for SO_2_ Gas Detection. ACS
Appl. Mater. Interfaces.

[40] Aranthady C., Jangid T., Gupta K., Mishra A. K., Kaushik S., Siruguri V., Rao G. M., Shanbhag G. V., Sundaram N. G. (2021). Selective
SO_2_ detection at low concentration by Ca substituted LaFeO_3_ chemiresistive gas sensor: a comparative study of LaFeO_3_ pellet vs thin film. Sens. Actuators
B: Chem..

[41] Zhao R., Wang W., Liu Y., Petkov P., Khan A. H., Gao L., Zhang P., Brunner E., Wang H. I., Singh S. (2025). A Donor–Acceptor-Type
Two-Dimensional Poly­(Arylene Vinylene)
for Efficient Electron Transport and Sensitive Chemiresistors. Angew. Chem., Int. Ed..

[42] Wang S., Fu Y., Wang F., Wang X., Yang Y., Wang M., Wang J., Lin E., Ma H., Chen Y. (2024). Scalable Melt Polymerization Synthesis of Covalent Organic Framework
Films for Room Temperature Low-Concentration SO_2_ Detection. J. Am. Chem. Soc..

[43] Zhao W., Obeso J. L., López-Cervantes V. B., Bahri M., Sánchez-González E., Amador-Sánchez Y. A., Ren J., Browning N. D., Peralta R. A., Barcaro G. (2025). Achieving
Sub-ppm Sensitivity in SO_2_ Detection with a Chemically
Stable Covalent Organic Framework. Angew. Chem.
Int. Ed..

[44] Bulemo P. M., Kim D.-H., Shin H., Cho H.-J., Koo W.-T., Choi S.-J., Park C., Ahn J., Güntner A. T., Penner R. M. (2025). Selectivity in Chemiresistive Gas Sensors:
Strategies and Challenges. Chem. Rev..

[45] Sayyad P. W., Khan S. S., Ingle N. N., Bodkhe G. A., Al-Gahouari T., Mahadik M. M., Shirsat S. M., Shirsat M. D. (2020). Chemiresistive SO_2_ sensor: graphene oxide
(GO) anchored poly (3,4-ethylenedioxythiophene):poly
(4-styrenesulfonate) (PEDOT: PSS). Appl. Phys.
A: Mater. Sci. Process..

[46] Zhu X., Chang X., Tang S., Chen X., Gao W., Niu S., Li J., Jiang Y., Sun S. (2022). Humidity-tolerant chemiresistive
gas sensors based on hydrophobic CeO_2_/SnO_2_ heterostructure
films. ACS Appl. Mater. Interfaces.

[47] Kim K., Park J. K., Lee J., Kwon Y. J., Choi H., Yang S.-M., Lee J.-H., Jeong Y. K. (2022). Synergistic approach
to simultaneously improve response and humidity-independence of metal-oxide
gas sensors. J. Hazard. Mater..

[48] Wang Y., Zhou Y. (2022). Recent progress on anti-humidity
strategies of chemiresistive gas
sensors. Materials.

[49] Park C., Baek J. W., Shin E., Kim I.-D. (2023). Two-dimensional
electrically conductive metal–organic frameworks as chemiresistive
sensors. ACS Nanosci. Au.

[50] Benedetto G., Mirica K. A. (2024). Conductive framework
materials for chemiresistive detection
and differentiation of toxic gases. Acc. Chem.
Res..

[51] Obeso J. L., Flores C. V., Peralta R. A., Viniegra M., Martín-Guaregua N., Huxley M. T., Solis-Ibarra D., Ibarra I. A., Janiak C. (2025). Metal–organic
frameworks (MOFs) toward SO_2_ detection. Chem. Soc. Rev..

[52] Chernikova V., Yassine O., Shekhah O., Eddaoudi M., Salama K. N. (2018). Highly
sensitive and selective SO_2_ MOF sensor: the integration
of MFM-300 MOF as a sensitive layer on a capacitive interdigitated
electrode. J. Mater. Chem. A.

[53] Tchalala M., Bhatt P., Chappanda K., Tavares S., Adil K., Belmabkhout Y., Shkurenko A., Cadiau A., Heymans N., De Weireld G. (2019). Fluorinated MOF platform for selective removal
and sensing of SO_2_ from flue gas and air. Nat. Commun..

[54] Zhong Z., Damacet P., Sánchez-González E., Eagleton A. M., Vereshchuk N., Wongratanaphisan R., Anderson J. T., Goncalves S., Peterson G. W., Blount B., Monti S., Barcaro G., Ibarra I. A., Mirica K. A. (2025). Scalable
templated fabrication of Cu-based MOF on textiles for simultaneous
sensing, filtration, and detoxification of SO_2_. Chem.

[55] Chan J. Y. M., Shehayeb E. O., Pennington D. L., Hendon C. H., Mirica K. A. (2025). Molecular
Engineering of a Conductive Metal–Organic Framework for Ultrasensitive,
Rapid, Selective, and Reversible Sensing of Nitric Oxide. J. Am. Chem. Soc..

[56] Shishkin V. N., Kudrik E. V., Shaposhnikov G. P. (2004). Synthesis and some properties of
5,6-(4,4’-dimethylaminophenyl)-2,3-dicyanopyrazine and its
porphyrazine derivative. ChemInform.

[57] Ding J., Wei Z., Li F., Zhang J., Zhang Q., Zhou J., Wang W., Liu Y., Zhang Z., Su X., Yang R., Liu W., Su C., Yang H. B., Huang Y., Zhai Y., Liu B. (2023). Atomic high-spin
cobalt
(II) center for highly selective electrochemical CO reduction to CH_3_OH. Nat. Commun..

[58] Piątek J., Budnyak T. M., Monti S., Barcaro G., Gueret R., Grape E. S., Jaworski A., Inge A. K., Rodrigues B. V. M., Slabon A. (2021). Toward Sustainable Li-Ion Battery
Recycling: Green
Metal–Organic Framework as a Molecular Sieve for the Selective
Separation of Cobalt and Nickel. ACS Sustain.
Chem. Eng..

[59] McNaught, A. D. ; Wilkinson, A. Compendium of Chemical Terminology; Blackwell Science Oxford, 1997; Vol. 1669.

[60] Hung C.-W., Lin K.-W., Liu R.-C., Tsai Y.-Y., Lai P.-H., Fu S.-I., Chen T.-P., Chen H.-I., Liu W.-C. (2007). On the
hydrogen sensing properties of a Pd/GaAs transistor-type gas sensor
in a nitrogen ambiance. Sens. Actuators B: Chem..

[61] de
Lourdes Gonzalez-Juarez M., Morales C., Flege J. I., Flores E., Martin-Gonzalez M., Nandhakumar I., Bradshaw D. (2022). Tunable carrier type of a semiconducting 2D metal–organic
framework Cu_3_(HHTP)_2_. ACS Appl. Mater. Interfaces.

[62] Baxter J., Grunze M., Kong C. (1988). Interaction of SO_2_ with
copper and copper oxide surfaces. J. Vac. Sci.
Technol., A.

[63] Bufalini M. (1971). Oxidation
sulfur dioxide in polluted atmospheres. Review. Environ. Sci. Technol..

[64] Liu T., Chan A. W., Abbatt J. P. (2021). Multiphase oxidation of sulfur dioxide
in aerosol particles: Implications for sulfate formation in polluted
environments. Environ. Sci. Technol..

[65] Flamm D. L., Bacon D., Kinsbron E., English A. T. (1981). Chemical Reaction
of Sulfur Dioxide at High Humidity and Temperature: Implications for
Accelerated Testing. J. Electrochem. Soc..

[66] Urone P., Lutsep H., Noyes C. M., Parcher J. F. (1968). Static studies of
sulfur dioxide reactions in air. Environ. Sci.
Technol..

[67] Tokunaga O., Nishimura K., Washino M. (1978). Radiation treatment of exhaust gases
II. Oxidation of sulfur dioxide in the moist mixture of oxygen and
nitrogen. Int. J. Appl. Radiat. Isot..

[68] Kellogg W., Cadle R., Allen E., Lazrus A., Martell E. (1972). The Sulfur
Cycle: Man’s contributions are compared to natural sources
of sulfur compounds in the atmosphere and oceans. Science.

[69] Smith R. A., Alexander R. B. (1986). Correlations
between stream sulphate and regional SO_2_ emissions. Nature.

[70] Hung H.-M., Hoffmann M. R. (2015). Oxidation of gas-phase SO_2_ on the surfaces
of acidic microdroplets: Implications for sulfate and sulfate radical
anion formation in the atmospheric liquid phase. Environ. Sci. Technol..

[71] Cadle R. (1972). Formation
and chemical reactions of atmospheric particles. J. Colloid Interface Sci..

[72] Zhai Z., Wang J., Sun Y., Hao X., Niu B., Xie H., Li C. (2023). MOFs/nanofiber-based capacitive gas
sensors for the
highly selective and sensitive sensing of trace SO_2_. Appl. Surf. Sci..

[73] Borka L. (1975). Hygroscopic
properties of potassium bromide in infrared spectrophotometry. Anal. Chem..

[74] Shelton R. D., Nielsen A., Fletcher W. (1953). The infrared spectrum and molecular
constants of sulfur dioxide. J. Chem. Phys..

[75] Kent S. A., Katzer J. R., Manogue W. H. (1977). Infrared
Spectroscopic Investigation
of the Adsorption and Reactions of SO_2_ on CuO. Ind. Eng. Chem. Fundam..

[76] Wojcicki A. (1974). Insertion
Reactions of Transition Metal–Carbon σ-Bonded Compounds
II. Sulfur Dioxide and Other Molecules. Adv.
Organomet. Chem..

[77] Kumar S., Kaur N., Sharma A. K., Mahajan A., Bedi R. (2017). Improved Cl_2_ sensing characteristics
of reduced graphene oxide when decorated
with copper phthalocyanine nanoflowers. RSC
Adv..

[78] Persson D., Leygraf C. (1990). Vibrational spectroscopy
and XPS for atmospheric corrosion
studies on copper. J. Electrochem. Soc..

[79] Berger F., Beche E., Berjoan R., Klein D., Chambaudet A. (1996). An XPS and
FTIR study of SO_2_ adsorption on SnO_2_ surfaces. Appl. Surf. Sci..

[80] Guimon C., Gervasini A., Auroux A. (2001). XPS study of the adsorption
of SO_2_ and NH_3_ over supported tin dioxide catalysts
used
in de-NO_x_ catalytic reaction. J.
Phys. Chem. B.

[81] Elder A. C., Bhattacharyya S., Nair S., Orlando T. M. (2018). Reactive adsorption
of humid SO_2_ on metal–organic framework nanosheets. J. Phys. Chem. C.

[82] Chen J., Kisimbiri G. W., Gladich I., Fauré N., Thomson E. S., Temperton R., Kanji Z. A., Kong X. (2025). Surface formation
pathway of nitrogen-and sulfur-containing organic compounds on ammonium
sulfate. J. Phys. Chem. A.

[83] Rameshan R., Nenning A., Raschhofer J., Lindenthal L., Ruh T., Summerer H., Opitz A. K., Martin Huber T., Rameshan C. (2020). Novel sample-stage for combined near
ambient pressure
x-ray photoelectron spectroscopy, catalytic characterization and electrochemical
impedance spectroscopy. Crystals.

[84] Smart R. S. C., Skinner W. M., Gerson A. R. (1999). XPS of sulphide mineral surfaces:
metal-deficient, polysulphides, defects and elemental sulphur. Surf. Interface Anal..

[85] Kundu M., Hasegawa T., Terabe K., Yamamoto K., Aono M. (2008). Structural
studies of copper sulfide films: effect of ambient atmosphere. Sci. Technol. Adv. Mater..

[86] Gong L., Zhao S., Yu J., Li J., Arbiol J., Kallio T., Calcabrini M., Martínez-Alanis P. R., Ibáñez M., Cabot A. (2024). Influence of the catalyst surface
chemistry on the electrochemical self-coupling of biomass-derived
benzaldehyde into hydrobenzoin. Energy Adv..

[87] Elith J. H., H Graham C., P Anderson R., Dudík M., Ferrier S., Guisan A., J Hijmans R., Huettmann F., R Leathwick J., Lehmann A. (2006). Novel
methods improve prediction of species’ distributions from occurrence
data. Ecography.

[88] Xin B., Jing L., Ren Z., Wang B., Fu H. (2005). Effects of
simultaneously doped and deposited Ag on the photocatalytic activity
and surface states of TiO_2_. J. Phys.
Chem. B.

[89] Watanabe M., Ando H., Handa T., Ichino T., Kuwaki N. (2007). Comparative
XPS study of silver and copper surfaces exposed to flowing air containing
low concentration of sulfur dioxide. Zairyo-to-Kankyo.

[90] Carsch K. M., Huang A. J., Dods M. N., Parker S. T., Rohde R. C., Jiang H. Z. H., Yabuuchi Y., Karstens S. L., Kwon H., Chakraborty R., Bustillo K. C., Meihaus K. R., Furukawa H., Minor A. M., Head-Gordon M., Long J. R. (2024). Selective Adsorption
of Oxygen from Humid Air in a Metal–Organic Framework with
Trigonal Pyramidal Copper­(I) Sites. J. Am. Chem.
Soc..

[91] Davydov A. A., Marshneva V. I., Shepotko M. L. (2003). Metal oxides in hydrogen sulfide
oxidation by oxygen and sulfur dioxide: I. The comparison study of
the catalytic activity. Mechanism of the interactions between H_2_S and SO_2_ on some oxides. Appl. Catal. A: Gen..

[92] Wu X., Tian X., Zhang W., Peng X., Zhou S., Buenconsejo P. J. S., Li Y., Xiao S., Tao J., Zhang M., Yuan H. (2024). Solution-Processable MOF-on-MOF System
Constructed via Template-Assisted Growth for Ultratrace H_2_S Detection. Angew. Chem., Int. Ed..

[93] Li Q., Liu X., Zhang Y., Wang X., Wang J., Wang B., Lu G., Liu F. (2026). Passivation-enhanced mixed-potential Cu-MOF sensor
for ppb-level H_2_S detection and real-time food freshness
evaluation. Sens. Actuators B: Chem..

[94] Benedetto G., Damacet P., Shehayeb E. O., Fabusola G., Simon C. M., Mirica K. A. (2025). Metal–organic framework-based
chemiresistive
array paired with machine learning algorithms for the detection and
differentiation of toxic gases. ACS Sens..

[95] Aykanat A., Meng Z., Stolz R. M., Morrell C. T., Mirica K. A. (2022). Bimetallic
Two-Dimensional Metal–Organic Frameworks for the Chemiresistive
Detection of Carbon Monoxide. Angew. Chem.,
Int. Ed..

[96] Martínez M. L., Marín-Rosas P., López-Cervantes V., Guzman-Vargas A., Peralta R. A., Solis-Ibarra D., Ibarra I. A. (2025). Fluorescence spectroscopy:
detection and sensing of SO_2_ and H_2_S using MOFs
and other emerging porous materials. Dalton
Trans..

[97] Lazanas A. C., Prodromidis M. I. (2023). Electrochemical
Impedance Spectroscopy–A Tutorial. ACS
Meas. Sci. Au.

[98] Balasubramani V., Chandraleka S., Rao T. S., Sasikumar R., Kuppusamy M. R., Sridhar T. M. (2020). Review Recent Advances in Electrochemical
Impedance Spectroscopy Based Toxic Gas Sensors Using Semiconducting
Metal Oxides. J. Electrochem. Soc..

[99] Prakasha B. S., Xiao P., José Esplandiu M., Yang J., Navarro-Urrios D., Rodríguez-Viejo J., Sledzinska M. (2025). Impedance-Assisted
Multivariate Analysis Technique for Enhanced Gas Sensing with 2D Dichalcogenides. ACS Sens..

[100] Damacet P., Shehayeb E. O., Mirica K. A. (2025). Controlling the
Spatiotemporal Self-Organization of Stimuli-Responsive Nanocrystals
under Out-of-Equilibrium Conditions. J. Am.
Chem. Soc..

[101] Meng Z., Aykanat A., Mirica K. A. (2019). Welding Metallophthalocyanines
into Bimetallic Molecular Meshes for Ultrasensitive, Low-Power Chemiresistive
Detection of Gases. J. Am. Chem. Soc..

