# Supplementary material for: Electronic Transport in Porous Nanocrystals Enables Ultrasensitive Consistent Detection of Sulfur Dioxide under Variable Humidity
Source: J Am Chem Soc. 2026 Jun 2;148(23):23540–53. doi: 10.1021/jacs.5c19328 (PMC13281522; doi:10.1021/jacs.5c19328)
Supplement: Supplementary file 1 [file ja5c19328_si_002.pdf]

Supporting Information

Electronic Transport in Porous Nanocrystals Enables Ultrasensitive Consistent Detection of Sulfur Dioxide Under Variable Humidity

Elissa O. Shehayeb,[a] Joseph Y. M. Chan,[a] Giovanni Barcaro,[b] Susanna Monti,[c]* and Katherine A. Mirica[a]*

a. Department of Chemistry, Burke Laboratory, Dartmouth College, Hanover, New Hampshire, 03755, United States
b. CNR-IPCF, Institute for Chemical and Physical Processes, Area della Ricerca, Pisa I-56124, Italy
c. CNR-ICCOM, Institute of Chemistry of Organometallic Compounds, Area della Ricerca, Pisa I56124, Italy

Email: sapeptides@gmail.com

Katherine.A.Mirica@dartmouth.edu

Table of Contents

S1. Materials and methods 2
S2. Synthesis of the monomer 4
S3. Synthesis of DC-103 13
S3.1. Optimization trials 13
S3.2. Optimized synthesis procedure of DC-103 18
S4. Characterization of DC-103 20
S4.1. Microscopy images of DC-103 20
S4.2. Elemental analysis of DC-103 22
S4.3. Thermal stability 22
S4.4. ATR-FTIR spectra 23
S4.5. XPS spectra 23
S4.6. EPR plots 24
S4.7. BET isotherms and surface areas 25
S4.8. Conductivity measurements 25
S4.8.1. Electrical conductivity 25
S4.8.2. Proton conductivity 27
S5. Computational details 27
S5.1. Model Building and Reactive Molecular Dynamics Simulations 27
S5.2. Quantum Chemistry calculations: Structural and electronic properties 29
S6. SO_2 Sensing experiments of DC-103 33

S6.1. Sensing experiments and calculations....33
S6.1.1. Preparation of chemiresistive devices 33
S6.1.2. Description of sensing setup 34
S6.1.3. Sensing experimental conditions....37
S6.1.4. Power consumption calculations 38
S6.1.5. Limits of Detection (LOD) calculations....39
S6.1.6. Initial rate calculations 41
S6.1.7. Response/recovery time calculations 41
S6.2. SO_2 sensing of DC-103 41
S6.3. Benchmark against previously reported materials for SO_2 sensing....58
S7. Spectroscopic analyses upon SO_2 exposure....61
S7.1. Diffuse Reflectance Infrared Fourier Transform Spectroscopy (DRIFTS)....61
S7.2. PXRD and XPS characterization after exposure to gases 65
S7.3. Relating spectroscopic analysis to sensing at 40 and 400 ppm 69
S8. Computational characterization of intermolecular interactions....75
S8.1. Reactive Molecular Dynamics Simulations 75
S8.2. Quantum Chemistry calculations....84
S9. Sensing responses of DC-103 towards other gases 91
S10. SO_2 and H_2S Sensing of Control Materials....96
S11. Impedance measurements....103
S11.1. General Experimental Notes....103
S11.2. Results and Discussion 104
S11.2.1. EIS measurements at different humidity levels....104
S11.2.2. EIS measurements upon exposure to SO_2 in dry atmosphere....110
S11.2.3. EIS measurements upon exposure to SO_2 in humid atmosphere....114
S12. Suspension stability 116
S13. References 121

S1. Materials and methods

Unless otherwise specified, all materials were purchased from commercial sources (Millipore Sigma, Thermo Fischer Scientific, Ambeed, or Beantown Chemicals) and used without further purification. NMR spectra were collected on a Bruker 600 MHz NMR spectrometer. Mass Spectra were carried out on a Waters Synapt G2-Si Electron Spray Ionization (ESI) Mass Spectrometer or a Bruker Autoflex Speed LRF Matrix-Assisted Laser Desorption Ionization (MALDI) spectrometer. Powder X-ray Diffraction (PXRD) patterns were recorded on a Rigaku sixth generation MiniFlex X-ray diffractometer with a Cu Kα (600 W, 40 kV, 15 mA, α = 1.54 Å) radiation source. Scanning Electron Microscopy (SEM) images were taken on a Thermo Scientific™

Helios™ 5 CX DualBeam microscope. Energy Dispersive X-ray (EDX) spectra were recorded using an Ultim Extreme 100 Oxford Instrument X-ray detector. Transmission Electron Microscopy (TEM) images were collected on a Thermo Scientific Talos F200i (S)TEM microscope. CHN elemental analysis data were performed using combustion by Atlantic Microlab, Inc. Fourier Transform – Infrared (FTIR) and Diffuse Reflectance Infrared Fourier Transform Spectroscopy (DRIFTS) data were acquired using a Nicolet 6700 FT-IR spectrometer. Nitrogen adsorption experiments for BET isotherms were conducted on an ASAP Plus 2020 3Flex (Micrometrics, Norcross, Georgia) instrument at 77K. Thermogravimetric Analysis (TGA) were recorded on a TGA 55 instrument from 25 to 900 °C with a ramp rate of 5 °C/min under nitrogen or air as purge gases. Bulk conductivity measurements were collected on pressed pellets of the materials using a Signatone tungsten carbide four-point linear probe or using a Gamry Potentiostat Interface 1010E for two-point probe measurements. The latter was also used to collect electrochemical impedance spectroscopy (EIS) measurements. Inductively Coupled Plasma – Mass Spectrometry (ICP-MS) experiments were performed on 8900 Triple Quadrupole ICP-QQQ-MS. X-ray photoelectron spectroscopy (XPS) experiments were performed on a Physical Electronics 5000 VersaProbe II Scanning ESCA Microprobe X-ray/Ultraviolet Photoelectron Spectrometer. Electron Paramagnetic Resonance (EPR) spectra were acquired using a Bruker BioSpin Gmbh spectrometer equipped with a standard mode cavity. Sensing experiments were performed using suspensions of the materials in water dropcasted on interdigitated gold electrodes with 5 or 10 μm gaps purchased from Metrohm (G-IDEAU5 or G-IDEAU10, respectively). Current readings were collected using a PalmSens MUX8-R2 potentiostat on PSTrace 5.8 and 5.9 software. Details of the sensing setup are described in **Section S5**, where analyte gases (sulfur dioxide, hydrogen sulfide, nitric oxide, nitrogen dioxide, ammonia, hydrogen, methane, carbon monoxide, and carbon dioxide) are purchased from Airgas as tanks constituting 10,000 ppm gas analyte in nitrogen or air (except carbon dioxide purchased as 100%). Micro-Trak and Smart-Trak mass flow controllers are acquired from Sierra Instruments, Inc. Sensing under humid conditions was achieved using a KIN-TEK Analytical FlexStream™ Gas Standard Generator System composed of a base module (FlexBase) and humidification module (FlexHG). Dynamic light scattering measurements were performed on a DynaPro NanoStar™, while zeta potential measurements were collected on a Malvern ZetaSizer Nano. Material Studio, VESTA, ImageJ, and OriginLab softwares were used to acquire the molecular structure and analysis images.

S2. Synthesis of the monomer

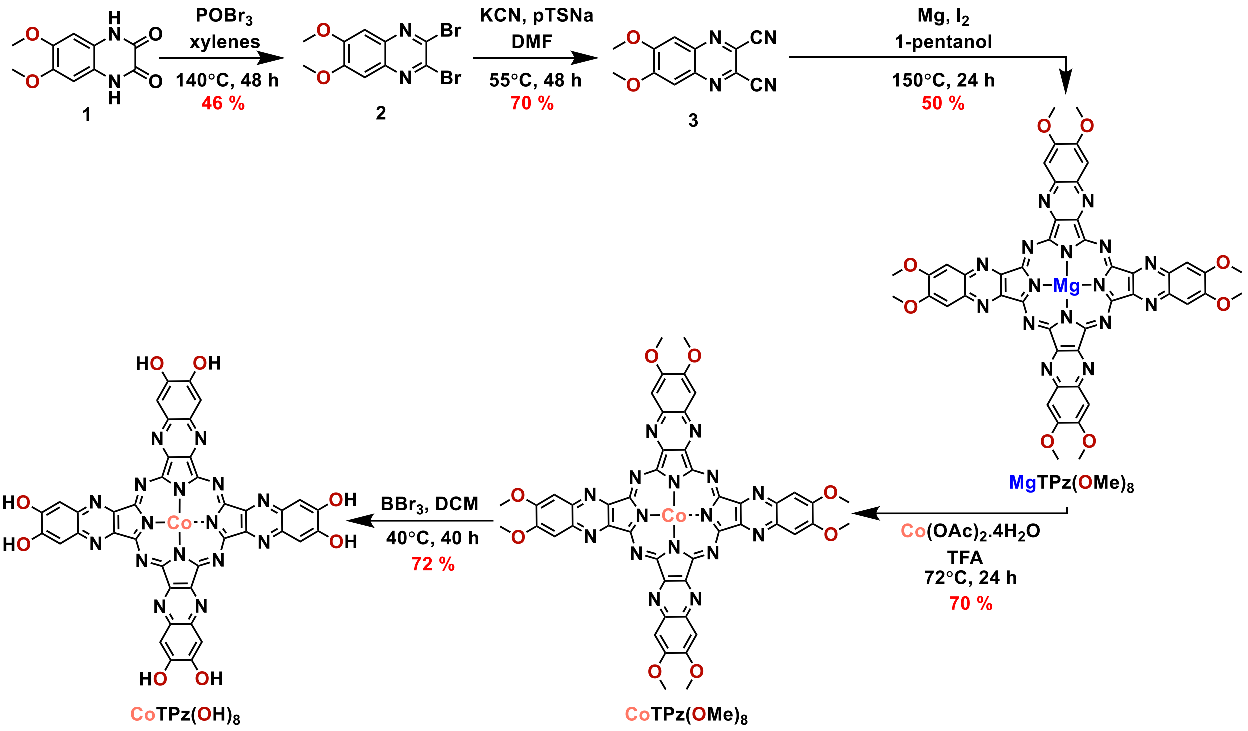

Scheme S1. Synthesis of CoTPz(OH)$_8$ monomer.

6,7-dimethoxy-1,4-dihydroquinoxaline-2,3-dione (1) was synthesized according to previously reported literature procedures.[1]

2,3-dibromo-6,7-dimethoxyquinoxaline (2). A round-bottom flask was charged with **1** (1.11 g, 5.00 mmol, 1 eq), phosphoryl bromide (3.3 g, 11.5 mmol, 2.3 eq), and 25 mL of xylenes mixture. It was assembled into a reflux setup with a nitrogen gas flow input and an outflow of gas into a base bath (to neutralize the exerted $HBr_{(g)}$). The reaction was stirred and refluxed at 140°C for 48 hours. The reaction mixture was then cooled to room temperature and poured over a beaker containing ice. A sodium hydroxide solution was added to make the medium basic. The product was dissolved and extracted from water with dichloromethane (4 × 100 mL). The organic layers were then combined, dried over magnesium sulfate, and evaporated under reduced pressure.

The resulting sticky solid was washed with a minimal amount of hexane and filtered to afford the pure product (0.8 g, 46 %). ^{1}H-NMR (600 MHz, $CDCl_3$, ppm): 7.29 (s, 2H) and 4.04 (s, 2H). ^{13}C-NMR (150 MHz, $CDCl_3$, ppm): 153.82, 138.64, 137.72, 105.79, 56.57. HRMS (ESI): $C_{10}H_9Br_2N_2O_2$, $[M+H]^+$ m/z calculated: 348.9010, found: 348.9012.

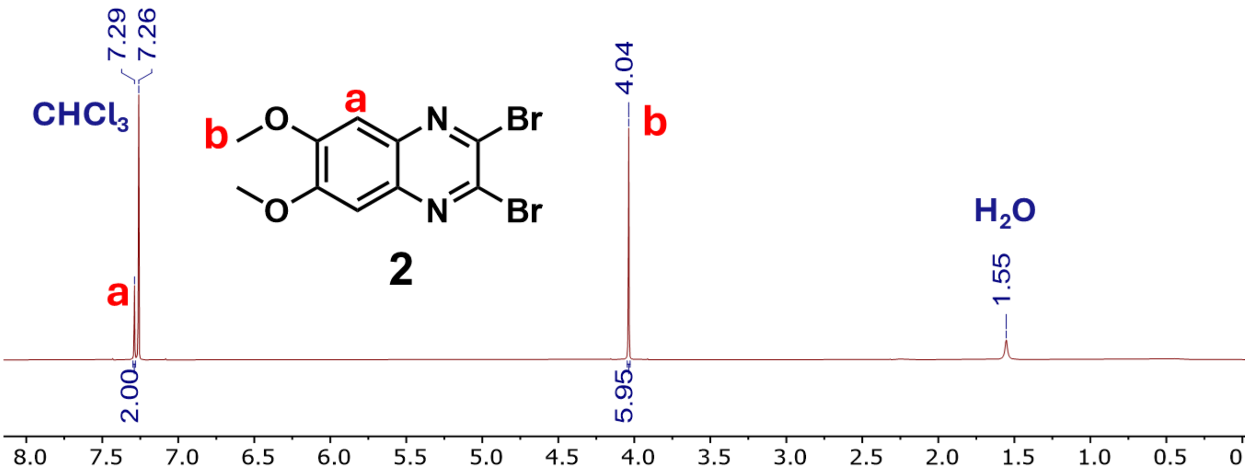

Figure S1. ^{1}H-NMR spectrum of **2** in $CDCl_3$ at 600 MHz.

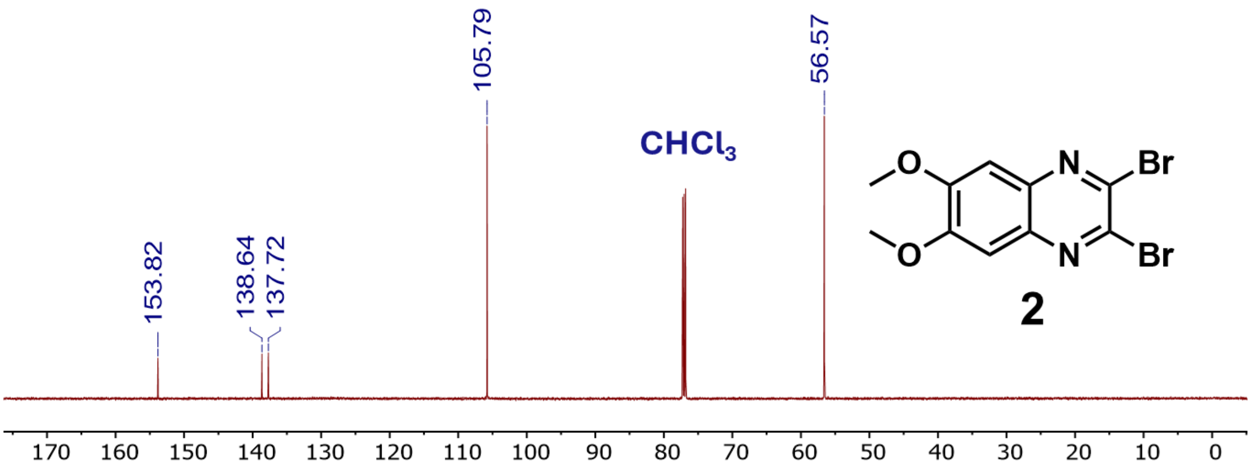

Figure S2. ^{13}C-NMR spectrum of **2** in $CDCl_3$ at 150 MHz.

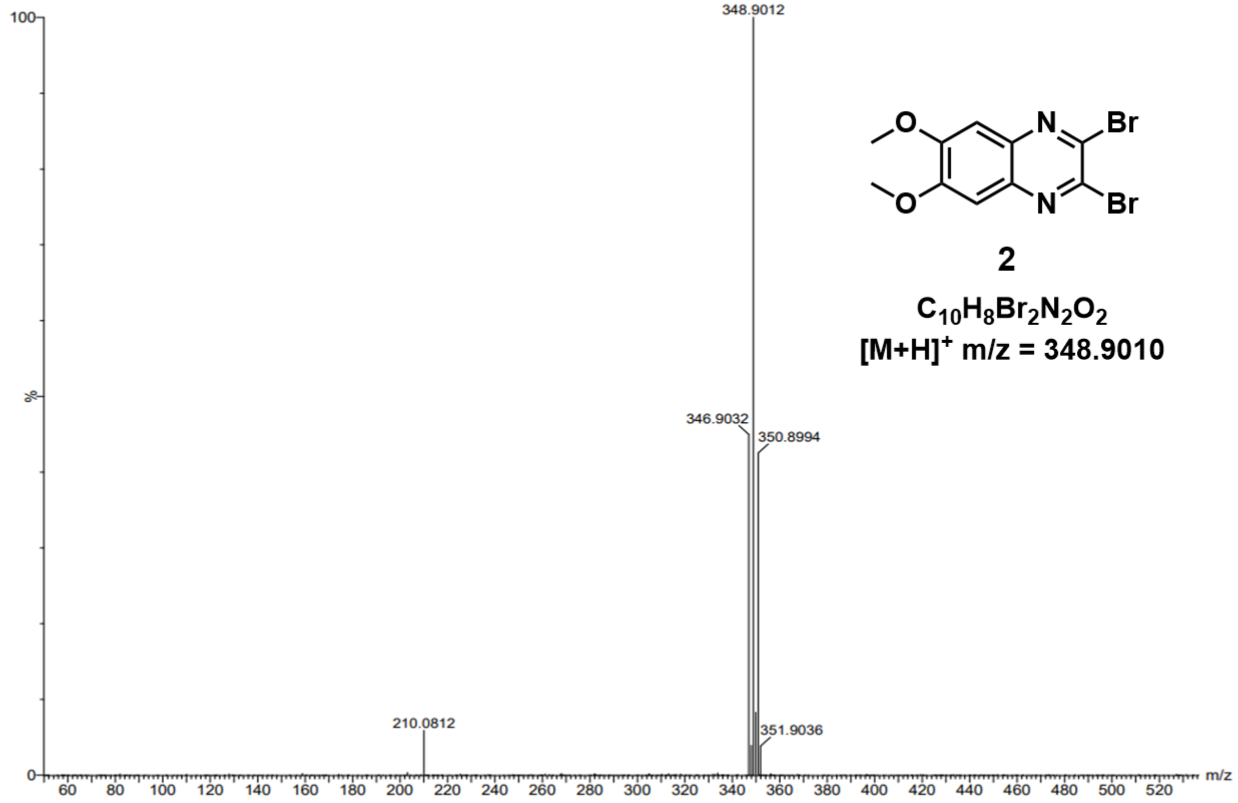

Figure S3. High-resolution mass spectrum (ESI) of **2**.

Synthesis of 6,7-dimethoxyquinoxaline-2,3-dicarbonitrile (3). The solid reactants: **2** (0.5 g, 1.44 mmol, 1 eq), potassium cyanide (0.428 g, 6.58 mmol, 4.6 eq), and *p*-toluene sulfinic acid sodium salt (0.254 g, 1.43 mmol, 1 eq) were added into a Schlenk tube with a stir bar and degassed by three vacuum-nitrogen cycles. Under nitrogen, 6 mL of anhydrous DMF were added using a syringe. The reaction mixture was heated at 55 °C under nitrogen for 20 hours. Then, the reaction was quenched with distilled water, the solid was collected by suction filtration. The pure product was obtained after drying the solid in a vacuum oven overnight (0.30 g, 88 %). ^{1}H-NMR (600 MHz, $CDCl_3$, ppm): 7.41 (s, 2H) and 4.13 (s, 2H). ^{13}C-NMR (150 MHz, $CDCl_3$, ppm): 157.18, 140.48, 128.23, 114.02, 106.36, 57.16. HRMS (ESI): $C_{12}H_9N_4O_2$, $[M+H]^+$ m/z calculated: 241.0681, found: 241.0721.

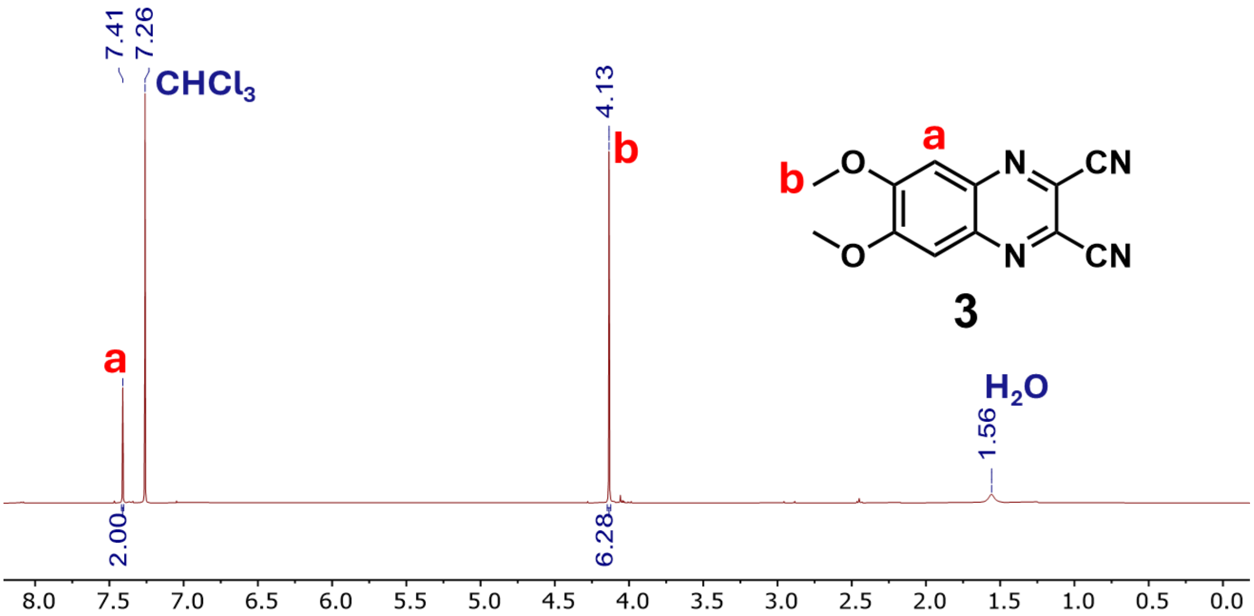

Figure S4. ^{1}H-NMR spectrum of **3** in $CDCl_3$ at 600 MHz.

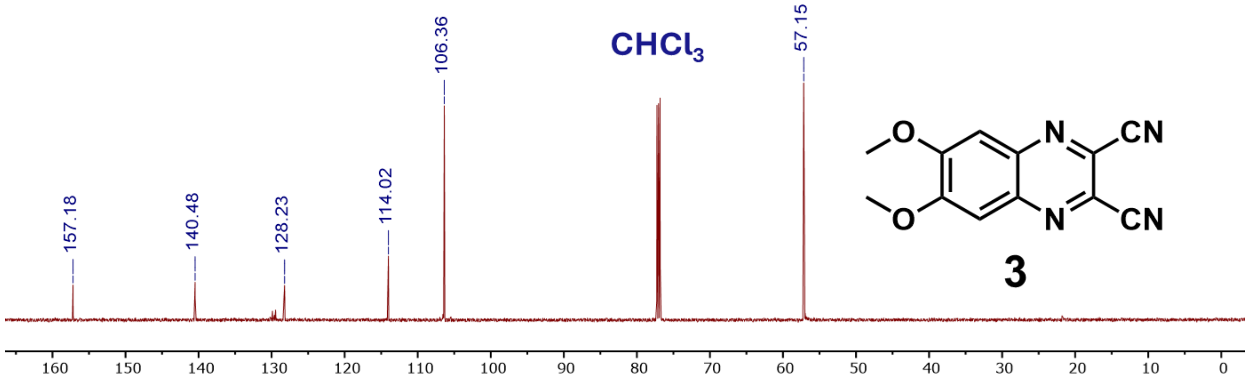

Figure S5. ^{13}C-NMR spectrum of **3** in $CDCl_3$ at 150 MHz.

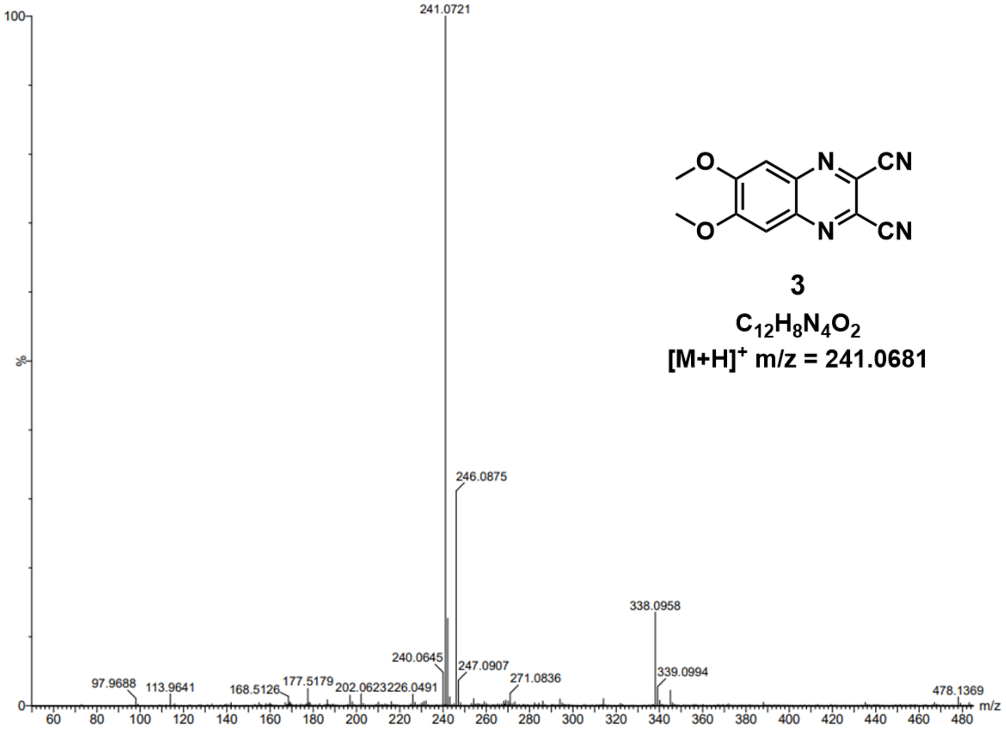

Figure S6. High-resolution mass spectrum (ESI) of **3**.

Synthesis of MgTPz(OMe)$_8$. In a 3-neck round-bottom flask, magnesium turnings (82 mg, 3.37 mmol, 4 eq) previously activated by a dilute hydrochloric acid solution were added with a stir bar. After degassing with three vacuum-nitrogen cycles, 6 mL of 1-pentanol and a crystal of sublimed iodine were added, and the reaction mixture was heated at 130 °C under nitrogen for at least 10 hours until a thick suspension of colorless crystals is observed. Under nitrogen, **5** (0.2 g, 0.832 mmol, 1 eq) was added, and the reaction mixture was further heated to 150 °C for 18 hours. It was then cooled to room temperature and suspended in ethyl acetate, and the solid was washed with methanol and acetone. The remaining precipitate was dried in a vacuum oven overnight to afford the pure product (101 mg, 49 %). ^{1}H-NMR (600 MHz, CF_3COOD, ppm): 8.41 (s, 8H) and 4.48 (s, 24H). HRMS (MALDI): $C_{48}H_{33}MgN_{16}O_8$, $[M+H]^+$ m/z calculated: 985.2518, found: 985.2489.

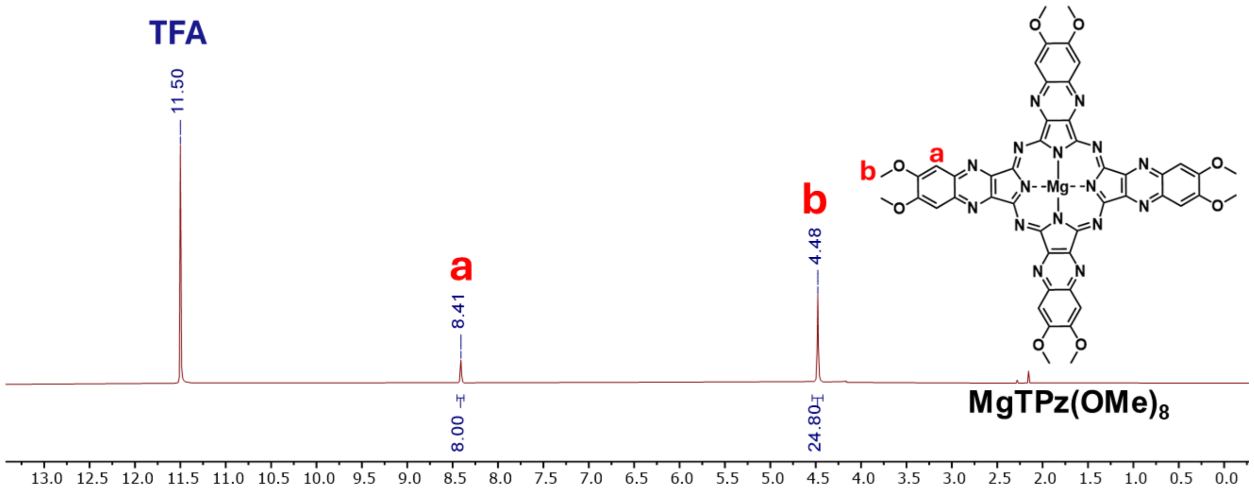

Figure S7. ^{1}H-NMR spectrum of **MgTPz(OMe)$_8$** in CF_3COOD at 600 MHz.

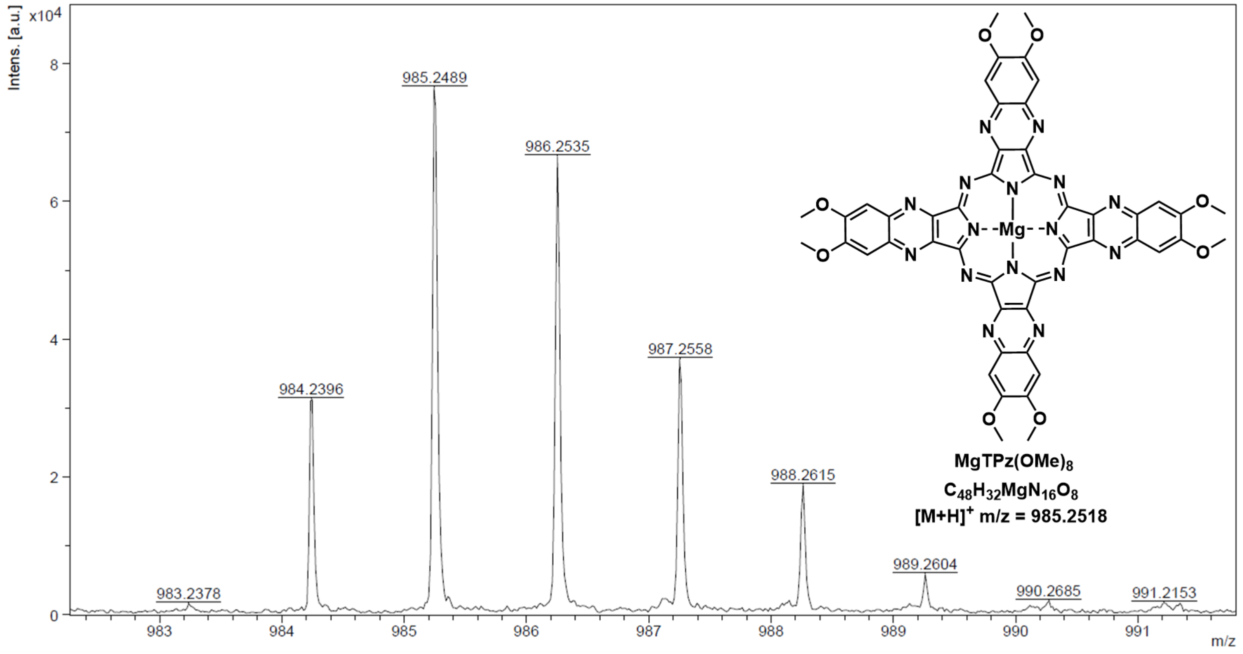

Figure S8. High-resolution mass spectrum (MALDI) of **MgTPz(OMe)$_8$**.

Synthesis of CoTPz(OMe)$_8$. A 3-neck round-bottom flask was charged with **MgTPz(OMe)$_8$** (100 mg, 0.101 mmol, 1 eq), cobalt (II) acetate tetrahydrate (100 mg, 0.40 mmol, 4 eq), and a stir bar. After its assembly in a reflux setup, the solids were degassed by three vacuum-nitrogen cycles. Trifluoroacetic acid (8 mL) was added under nitrogen, and the reaction mixture was refluxed at 72 °C overnight. The resulting solution was evaporated, and the remaining solid was washed with water and acetone in a centrifuge tube. The precipitate was dried in a vacuum oven to afford the pure dark blue product (73 mg, 70 %). ^{1}H-NMR (600 MHz, CF_3COOD, ppm): 8.59 (s, 8H) and 3.92 (s, 24H). ^{13}C-NMR spectrum could not be obtained due to poor solubility in common solvents. HRMS (MALDI): $C_{48}H_{32}CoN_{16}O_8$, $[M]^+$ m/z calculated = 1019.1921, found = 1019.1915.

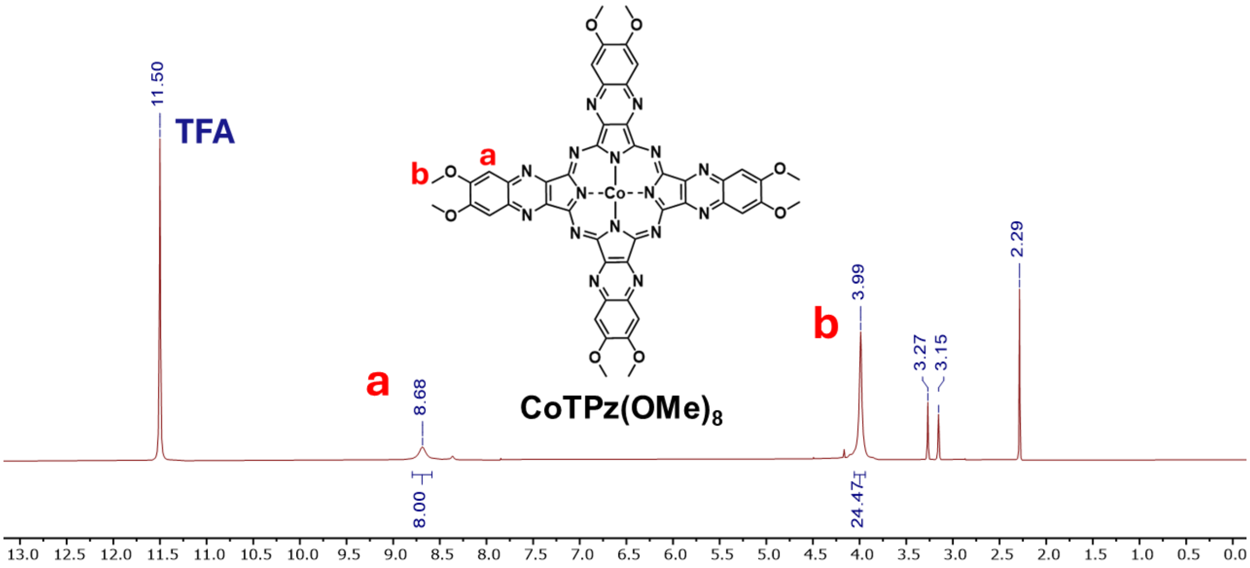

Figure S9. ^{1}H-NMR spectrum of **CoTPz(OMe)$_8$** in CF_3COOD at 600 MHz.

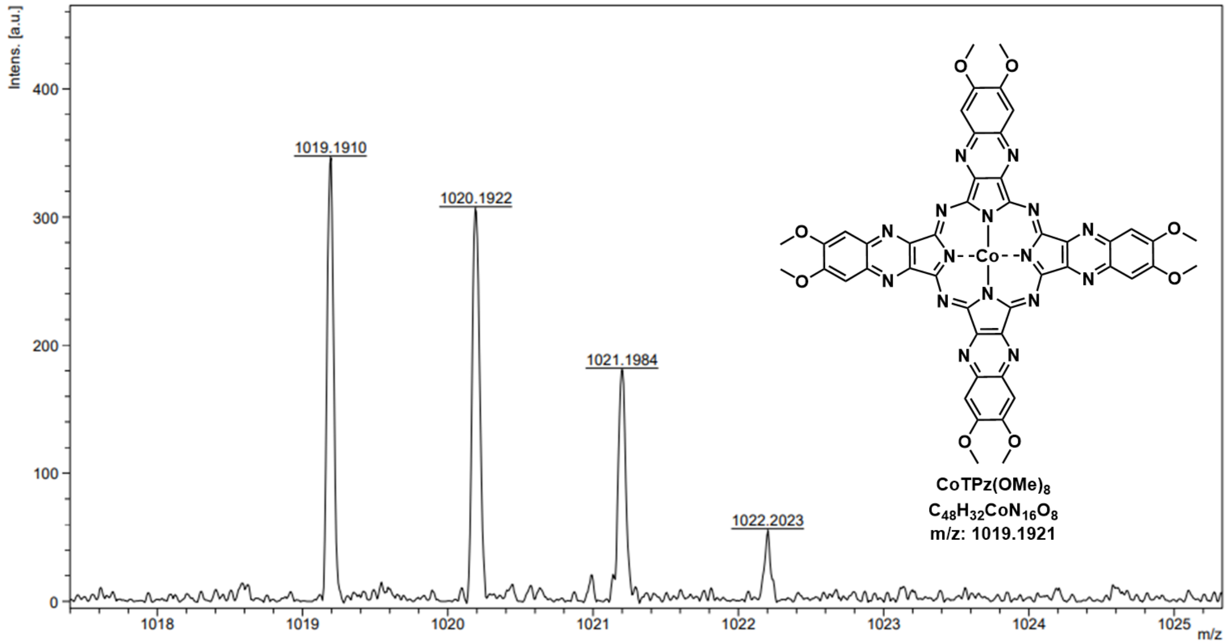

Figure S10. High-resolution mass spectrum (MALDI) of **CoTPz(OMe)$_8$**.

Synthesis of CoTPz(OH)$_8$. In a three-neck round bottom flask fitted into a reflux setup and connected to the Schlenk line, **CoTPz(OMe)$_8$** (220 mg, 0.216 mmol, 1 eq) was added with a magnetic stirrer. The solid was degassed with three vacuum-nitrogen cycles, and under nitrogen, 4.5 mL of anhydrous DCM were added followed by the dropwise addition of boron tribromide (1.03 mL, 10.8 mmol, 50 eq). The reaction mixture was stirred at reflux under nitrogen for 40 hours. After cooling to room temperature, the reaction mixture was poured onto ice with methanol, and the resulting suspension was centrifuged and decanted. The obtained solid was then washed, centrifuged, and decanted successively with methanol and acetone. The solid was then dissolved in around 15 mL of *N*,*N*-dimethylformamide (DMF), followed by its precipitation in 400 mL of water. The precipitate was collected by suction filtration, washed with acetone, and dried under vacuum to obtain the pure dark bluish-green powder (160 mg, 82 %). ^{1}H-NMR (600 MHz, DMSO-d_6, ppm): δ = 11.67 (s, 8H) and 8.61 (s, 8H). HRMS (MALDI): $C_{40}H_{17}CoN_{16}O_8$, $[M+H]^+$ m/z calculated = 908.0703, found = 908.0754.

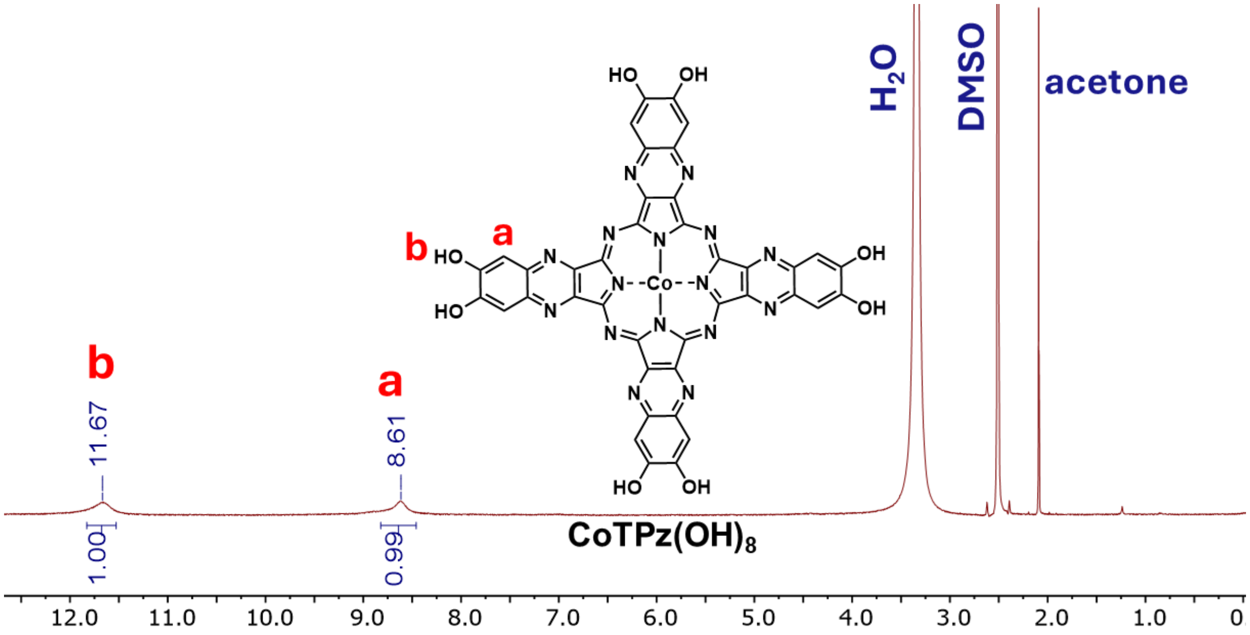

Figure S11. ^{1}H-NMR spectrum of **CoTPz(OH)$_8$** in DMSO-d_6 at 600 MHz.

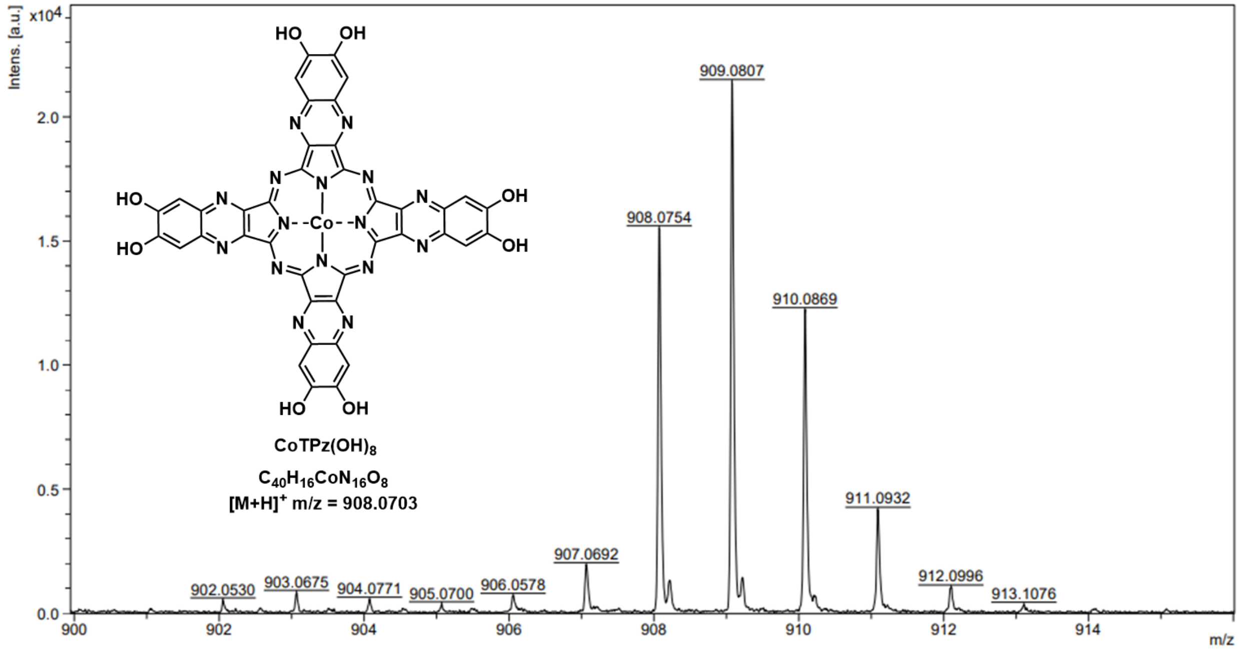

Figure S12. High-resolution mass spectrum (MALDI) of **CoTPz(OH)$_8$**.

S3. Synthesis of DC-103

S3.1. Optimization trials

General procedure followed for the optimization trials of DC-103

Generally, **CoTPz(OH)$_8$** is first introduced into a high-pressure Schlenk tube or a scintillation vial, in case of inert or ambient atmospheres, respectively. If under nitrogen, the solid is initially degassed three times. The solvent in then added, and the mixture is sonicated for around 5-10 minutes until completely soluble. A solution of the metal salt in the solvent is then added, followed by the additive, if any. The reaction vessel is capped and placed in an oven, preheated at 85 °C, for 15 hours.

The conditions followed are tabulated in **Table S1** and their corresponding PXRD patterns are shown in **Figure S13**.

Rationale for the Selection of Optimal Synthesis Conditions

The optimization of MOF synthesis conditions was guided primarily by material crystallinity as assessed by PXRD. Key criteria included peak sharpness, relative (100)/(001) peak intensity ratios, full width at half maximum (FWHM), and minimization of additives where possible.

Across all entries investigated, we maintained several common principles.

i) Choice of solvent: Owing to the poor solubility of the monomer in most conventional solvents, and based on our prior studies on the DC-100 family of MOFs,[2] anhydrous dimethyl sulfoxide (DMSO) was selected as the solvent for all syntheses.

ii) Choice of additive: All entries incorporated a basic additive to promote ligand deprotonation and subsequent partial oxidation of the catecholate moieties, a prerequisite for two-dimensional conductive MOF formation.[3] Ethylenediamine (EDA) was chosen based on its effectiveness in our previous report of DC-100,[2] attributed to its role as a mediator, in which it binds weakly to Cu^{2+}

ions, thus slowing the nucleation process and promoting crystal growth.[4] We employed EDA as an additive in all entries except entry 3, where copper acetate was used as the metal source (as discussed later).

iii) <u>Metal salt equivalence:</u> The equivalence of the Cu^{2+} salt was initially set at a maximum of 2.5 and later reduced to 2.1 to minimize potential coordination of excess Cu^{2+} to the pyrazine nitrogen atoms on the ligand.[5]

iv) <u>Reaction temperature and time:</u> These parameters were held constant at 85 °C and 1 day throughout, based on optimized conditions reported previously.[2]

Initial syntheses of DC-103 were conducted under ambient air in scintillation vials at moderate-to-high monomer concentrations of 5.51 mM (entry 1) and 1.1 mM (entry 2), achieved by varying the volume of DMSO while keeping the metal salt ($Cu(NO_3)_2$•$2.5H_2O$), its equivalence, and the amount and concentration of additive constant. PXRD patterns for both entries confirmed formation of the target MOF structure, with reflections at 3.9°, 7.9°, and 27.5°, corresponding to the (100), (200), and (001) planes, respectively. However, the diffraction peaks were relatively broad, indicating limited crystallinity and motivating further optimization. Substitution of $Cu(NO_3)_2$•$2.5H_2O$ with $Cu(OAc)_2$, wherein acetate counterions may act as an internal base and thus eliminate the need for an external additive, resulted in MOF formation with similarly low crystallinity (entry 3).

Substantial enhancement in crystallinity was observed upon increasing monomer dilution to 0.44 mM under ambient air (entry 4), accompanied by a large excess of EDA (1600 equivalents), conditions previously identified as optimal for DC-100.[2] Under these conditions, PXRD patterns exhibited sharper (100) and (200) reflections and the emergence of the (110) peak at 5.6°. Performing the synthesis under otherwise identical conditions but under an inert N_2

atmosphere (entry 5) yielded further improvement, with noticeably sharper peaks and reduced FWHM values.

Building on the high crystallinity achieved under inert conditions, two parameters were systematically and concurrently varied:

i) EDA equivalence: To reduce the amount of additive potentially retained in the pores or during activation while maintaining high crystallinity, the EDA equivalence was decreased from 1600 to 40, 100, 400, and 1000 in entries 6, 7, 15, and 8, respectively. Entries 6–8 retained the principal diffraction peaks but exhibited broader reflections relative to entry 5, indicating diminished crystallinity. In contrast, entry 15 maintained sharp peaks and high crystallinity despite a 4-fold reduction in EDA equivalence.

ii) Solvent volume: In parallel, we attempted reducing the volume of DMSO while maintaining 1600 equivalents of EDA (entries 9–11). These conditions resulted in broader peaks and, in some cases, weakened or absent (110) reflections, rendering them less optimal.

Additional combinations of monomer concentration and additive equivalence (entries 12–14) similarly produced materials with increased FWHM and reduced peak sharpness.

Based on these observations, entry 15 was identified as the optimal synthesis condition, employing $Cu(NO_3)_2$•$2.5H_2O$ as the metal source, a reduced EDA equivalence of 400, high monomer dilution (0.44 mM), and inert N_2 atmosphere.

Table S1. A summary of the reaction conditions followed for the optimization of DC-103 synthesis

Entry	[monomer] (mM)	Metal salt (eq)	Additive (eq)	Solvent (mL)	Temp (°C)	Time (days)	Air/ N_2
1	5.51	$Cu(NO_3)_2$•2.5H_2O (2.5 eq)	EDA (40 eq)	DMSO (2 mL)	85	1	Air
2	1.1	$Cu(NO_3)_2$•2.5H_2O (2.5 eq)	EDA (40 eq)	DMSO (10 mL)	85	1	Air
3	5.51	$Cu(OAc)_2$ (2.5 eq)	-	DMSO (2 mL)	85	1	Air
4	0.44	$Cu(NO_3)_2$•2.5H_2O (2.5 eq)	EDA (1600 eq)	DMSO (25 mL)	85	1	Air
5	0.44	$Cu(NO_3)_2$•2.5H_2O (2.1 eq)	EDA (1600 eq)	DMSO (25 mL)	85	1	N_2
6	0.44	$Cu(NO_3)_2$•2.5H_2O (2.1 eq)	EDA (40 eq)	DMSO (25 mL)	85	1	N_2
7	0.44	$Cu(NO_3)_2$•2.5H_2O (2.1 eq)	EDA (100 eq)	DMSO (25 mL)	85	1	N_2
8	0.44	$Cu(NO_3)_2$•2.5H_2O (2.1 eq)	EDA (1000 eq)	DMSO (25 mL)	85	1	N_2
9	0.55	$Cu(NO_3)_2$•2.5H_2O (2.1 eq)	EDA (1600 eq)	DMSO (20 mL)	85	1	N_2
10	1.1	$Cu(NO_3)_2$•2.5H_2O (2.1 eq)	EDA (1600 eq)	DMSO (10 mL)	85	1	N_2
11	2.2	$Cu(NO_3)_2$•2.5H_2O (2.1 eq)	EDA (1600 eq)	DMSO (5 mL)	85	1	N_2
12	1.1	$Cu(NO_3)_2$•2.5H_2O (2.1 eq)	EDA (400 eq)	DMSO (10 mL)	85	1	N_2
13	0.55	$Cu(NO_3)_2$•2.5H_2O (2.1 eq)	EDA (40 eq)	DMSO (20 mL)	85	1	N_2
14	0.55	$Cu(NO_3)_2$•2.5H_2O (2.1 eq)	EDA (400 eq)	DMSO (20 mL)	85	1	N_2
15	0.44	$Cu(NO_3)_2$•2.5H_2O (2.1 eq)	EDA (400 eq)	DMSO (25 mL)	85	1	N_2

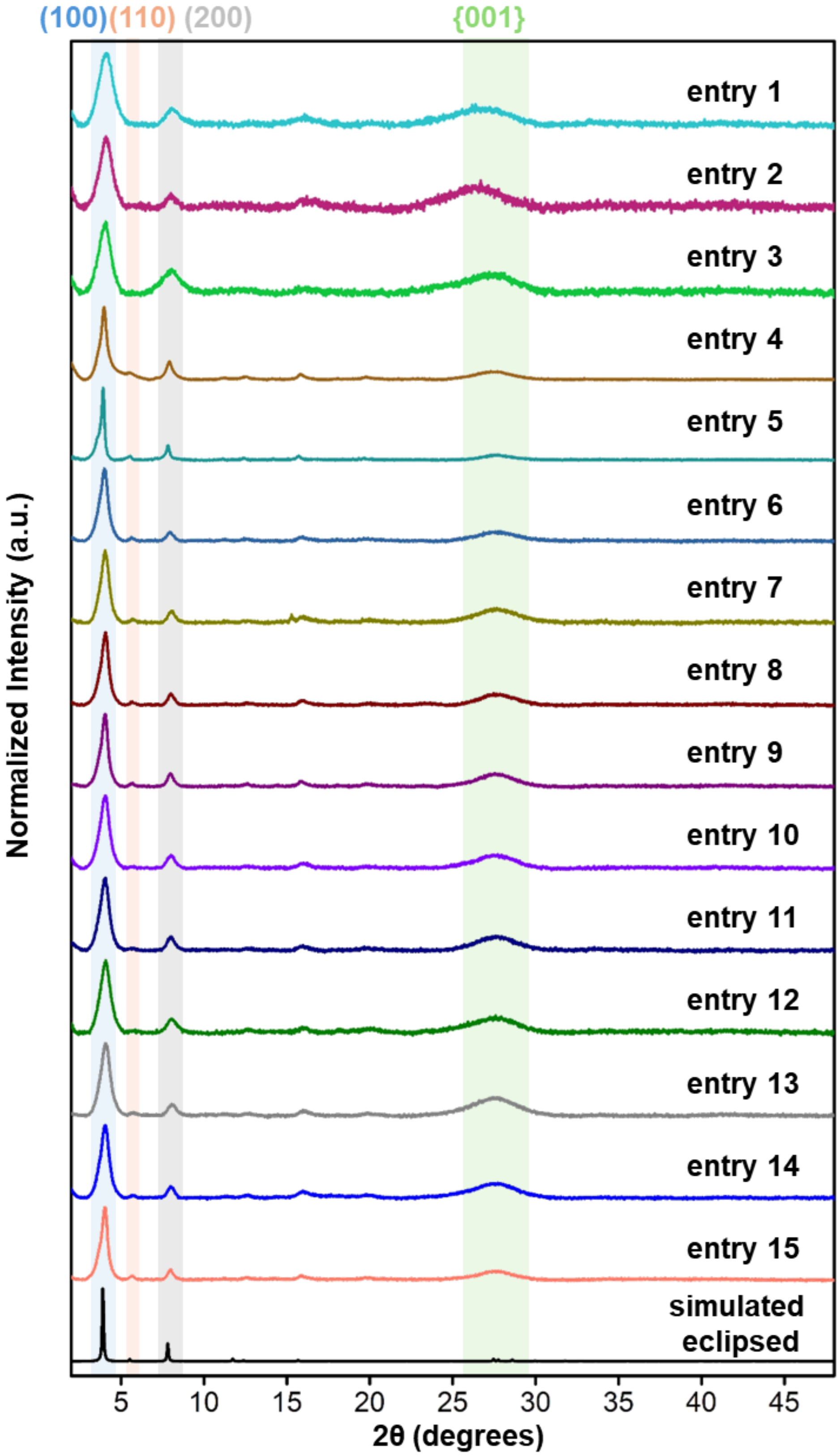

Figure S13. PXRD patterns for the reaction conditions described in **Table S1** for the optimization of DC-103 synthesis, with entry 15 chosen as the optimal condition.

S3.2. Optimized synthesis procedure of DC-103

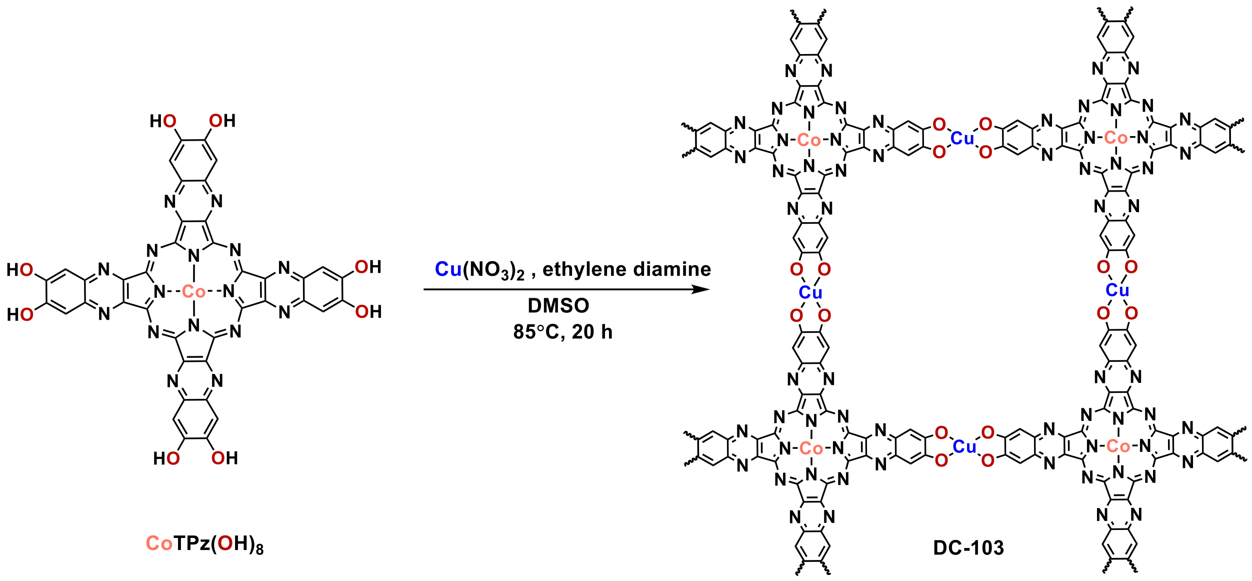

Scheme S2. Synthesis of DC-103.

CoTPz(OH)$_8$ (10 mg, 0.011 mmol, 1 eq) was charged into a high-pressure Schlenk flask and subjected to three vacuum-nitrogen cycles. Under nitrogen, 24 mL of anhydrous DMSO were added, then the tube was sealed and sonicated for 5 minutes. Meanwhile, a 23 mM solution of copper (II) nitrate hemi(pentahydrate), $Cu(NO_3)_2 \cdot 2.5H_2O$, (5.38 mg, 0.023 mmol, 2.1 eq) in 1 mL of anhydrous DMSO was prepared. The reaction vessel was reconnected to argon, and it was opened to add the salt solution followed by ethylene diamine (0.29 mL, 4.4 mmol, 400 eq). The Schlenk tube was sealed and placed in a preheated oven at 85 °C for 15 hours. After cooling to room temperature, the light brown solution with black precipitate was transferred to a centrifuge tube, and the resulting solid was washed and decanted successively with 25 mL of DMF, water, and acetone. The MOF bulk powder was dried under vacuum overnight, and their crystallinities were confirmed by their PXRD diffraction patterns, shown in **Figure 1b**. The MOFs were activated by soaking them for 24 hours in each of DMSO (2 × 20 mL) and THF (1 × 20 mL), followed by drying in a vacuum oven at 60 °C overnight.

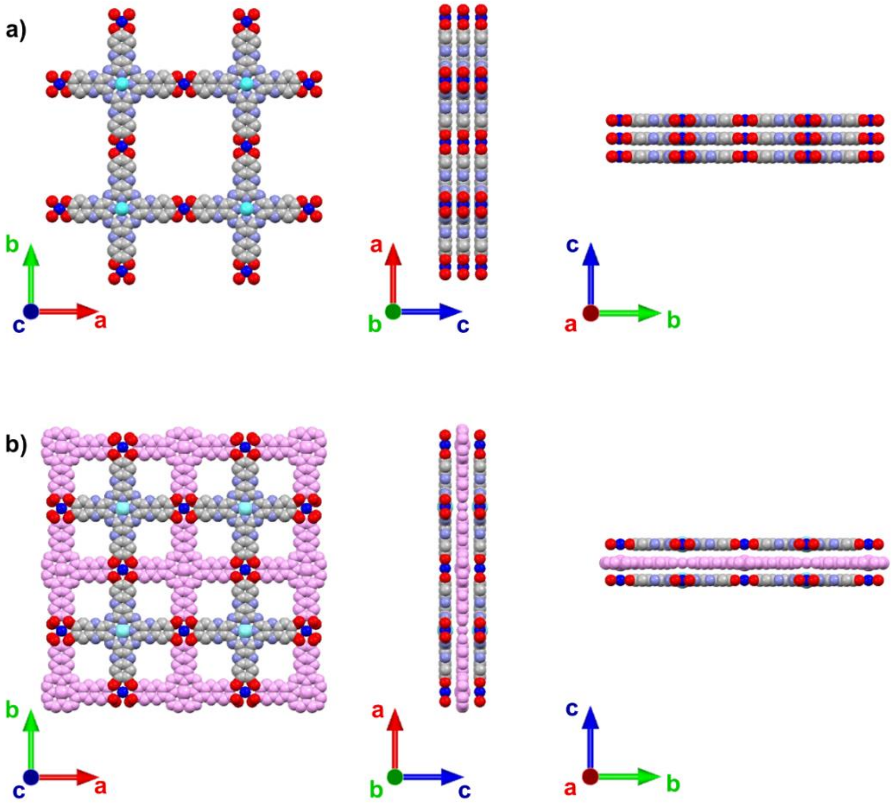

Figure S14. Front and side views of the a) eclipsed and b) staggered packing of DC-103.

S4. Characterization of DC-103

S4.1. Microscopy images of DC-103

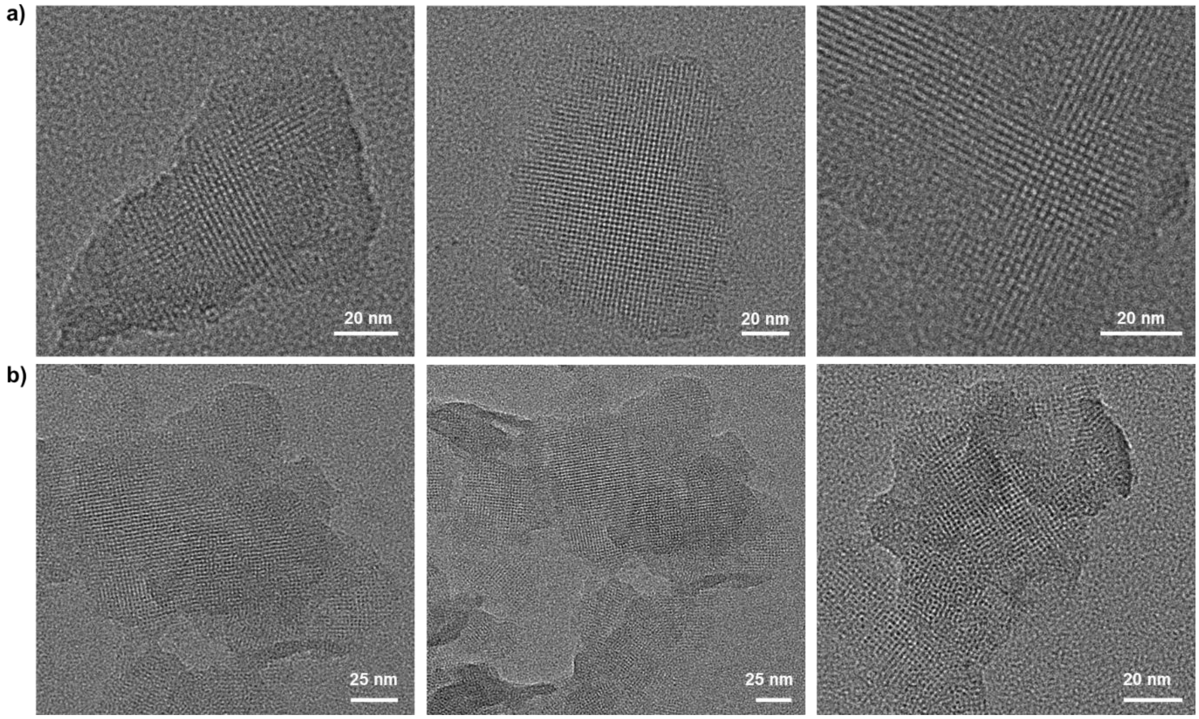

Figure S15. TEM images from different batches of DC-103 taken at different magnifications.

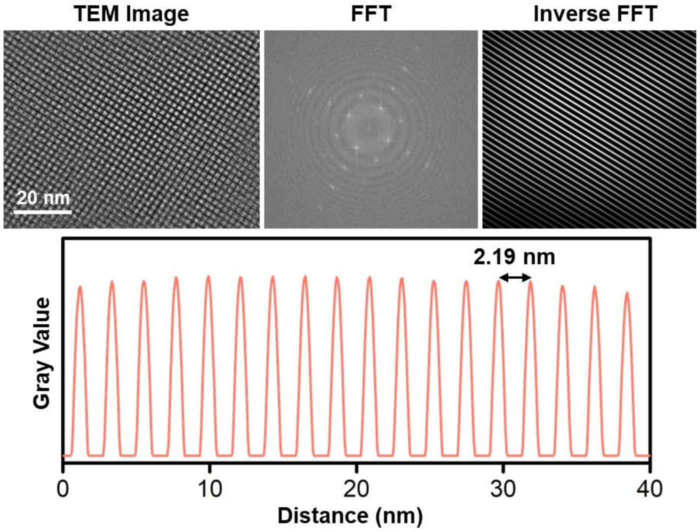

Figure S16. Determination of the interlayer spacing distances of DC-103 from its TEM image with FFT and inverse FFT analyses on ImageJ software. The gray value plots were determined from the inverse FFT for the MOF.

The interplanar spacing extracted experimentally from the TEM images 2.192 ± 0.038 nm, which matches the theoretically expected spacing between (100) planes passing through the TPz metal centers of 2.2 nm.

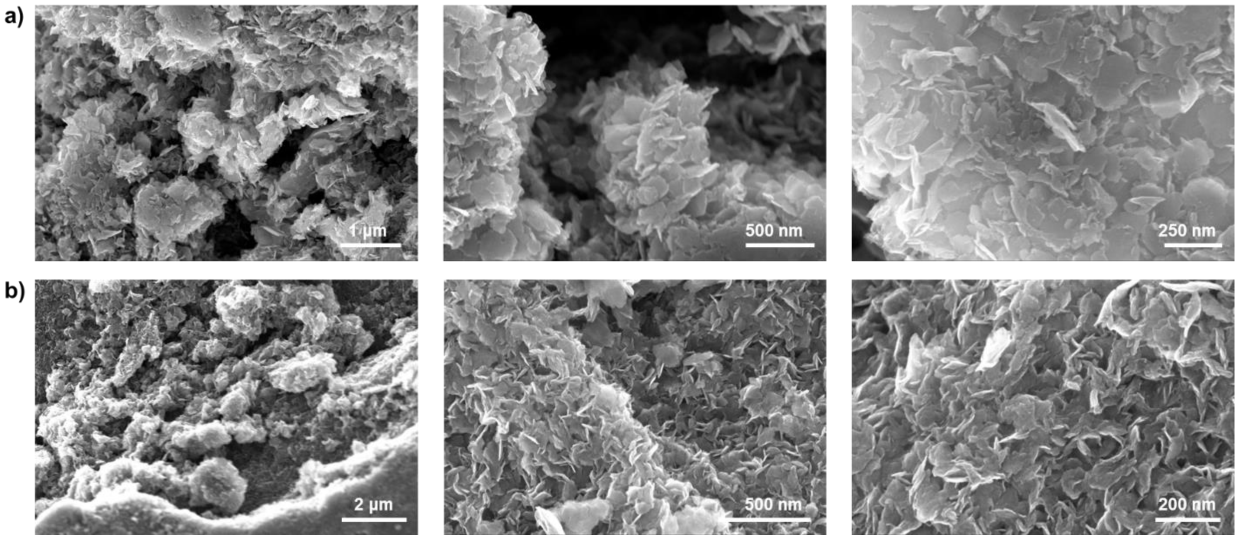

Figure S17. SEM images from different batches of DC-103 (a) and (b) taken at different magnifications.

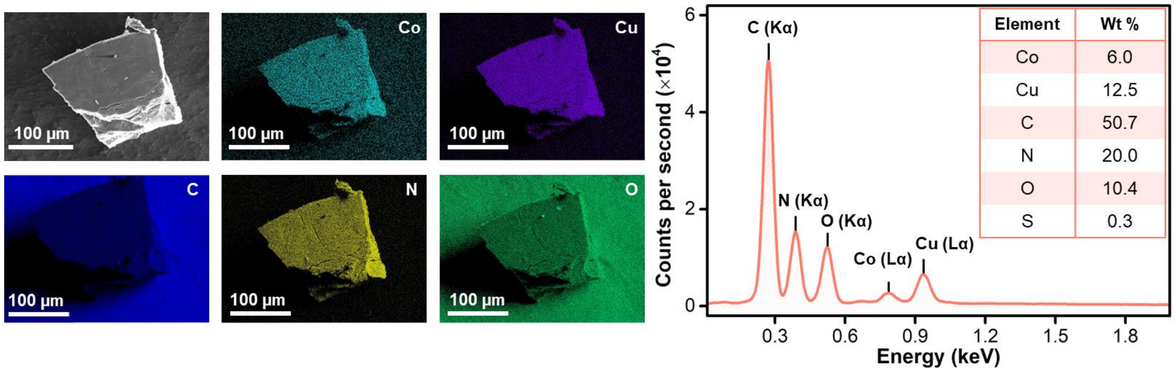

Figure S18. EDX mapping of DC-103.

S4.2. Elemental analysis of DC-103

To quantify the metal content of the MOFs, we performed inductively-coupled plasma mass spectrometry (ICP-MS) on a diluted sample of MOF, digested in a 9:1 solution of concentrated sulfuric acid and hydrogen peroxide (6%).

Table S2. Table summarizing the CHNS combustion and the ICP-MS analysis results of DC-103, as well as its expected molecular formula. (theo % = theoretical percentage based on the simulated unit cell, exp % = average of two experimental percentage values)

		**$CoTPzCu_2$**		**$CoTPzCu_2(H_2O)_3(DMSO)_{0.1}$**	
		theo (%)	exp (%)	theo (%)	exp (%)
**CHNS combustion**	C	46.80	41.13	44.34	41.13
	H	0.79	3.56	1.34	3.56
	N	21.83	18.49	20.59	18.49
	S	0.00	0.35	0.29	0.34
**ICP-MS**	M	(M = Co) 5.74	5.44	(M = Co) 5.41	5.44
	Cu	12.38	13.79	11.68	13.79
	O	12.46	calc. 17.23	16.33	calc. 17.25

S4.3. Thermal stability

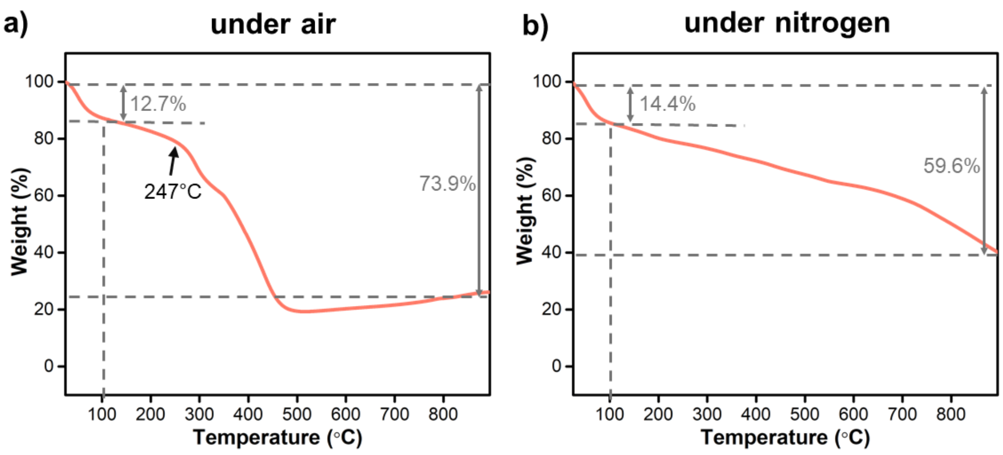

Figure S19. TGA traces of DC-103 a) in air and b) in nitrogen. Plots were collected from 25 to 900 °C at a ramp rate of 5 °C/min.

S4.4. ATR-FTIR spectra

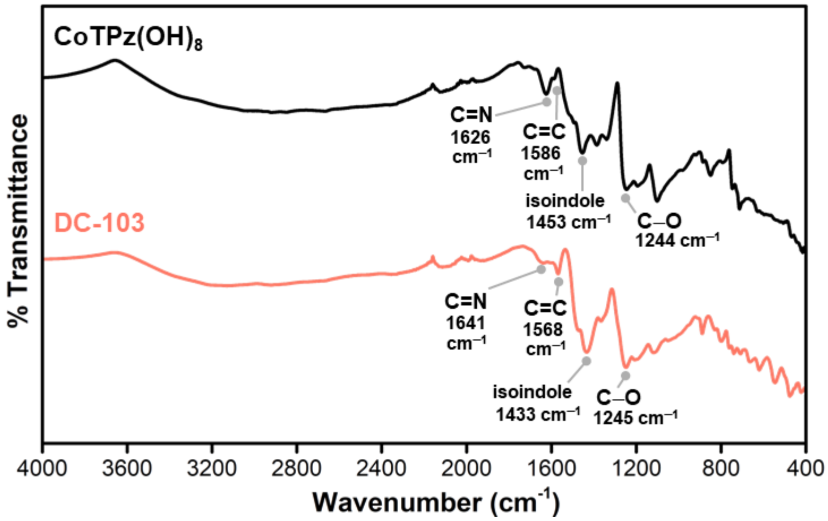

Figure S20. ATR-FTIR spectra of $CoTPz(OH)_8$ monomer and DC-103.

S4.5. XPS spectra

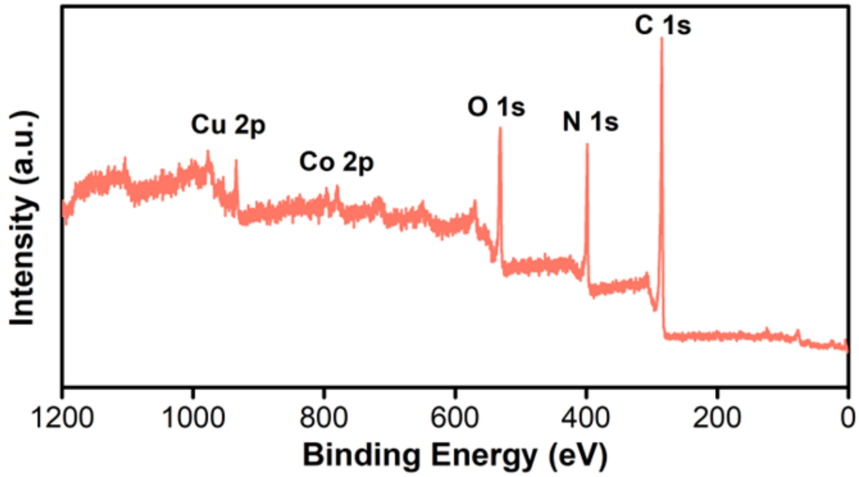

Figure S21. XPS survey spectrum of DC-103.

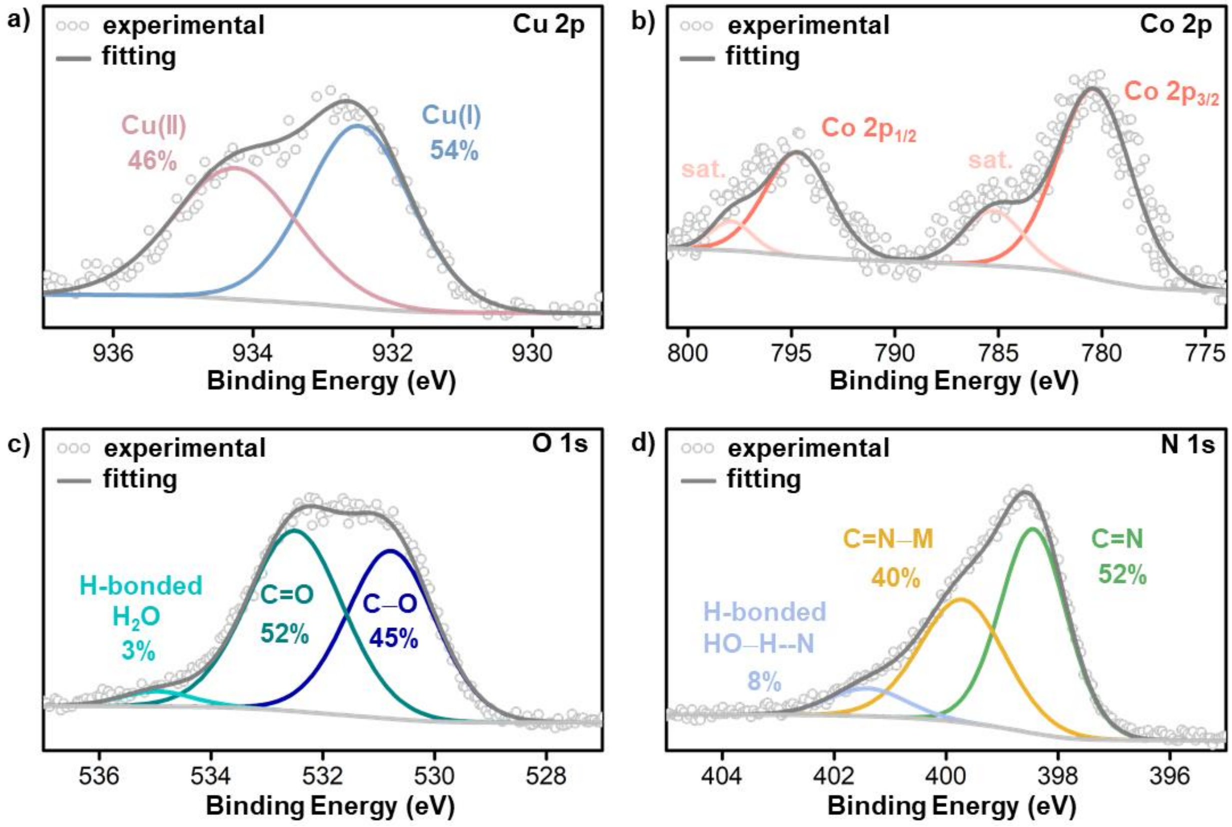

Figure S22. High-resolution XPS spectra of DC-103 at the binding energies of a) Cu 2p, b) Co 2p, c) O 1s, and d) N 1s.

S4.6. EPR plots

2 mg of each sample were placed in quartz EPR tubes and analyzed at room temperature. $CoTPz(OH)_8$ shows a weak signal with g = 2.257, which we attributed to the low-spin d^7 configuration of Co(II) constituting the core of the TPz ligand. Accordingly, we assigned the EPR signal at g = 2.068 in DC-103 to that of paramagnetic Co^{2+} or Cu^{2+} of the MOF, consistent with previous reports.[6]

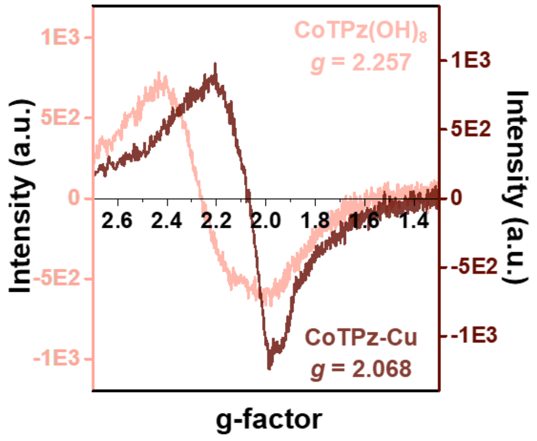

Figure S23. EPR plots taken at room temperature of $CoTPz(OH)_8$ monomer and DC-103.

S4.7. BET isotherms and surface areas

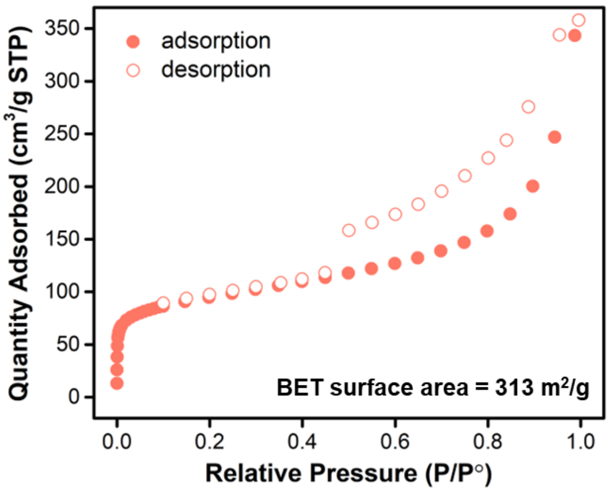

Figure S24. BET N_2 adsorption and desorption isotherms for DC-103 recorded at 77K.

S4.8. Conductivity measurements

S4.8.1. Electrical conductivity

Electrical conductivity measurements were collected on pressed pellets of DC-103 using two methods: 1) by a four-point linear probe under ambient atmosphere, and 2) by a two-point probe under ambient and saturated humid atmosphere.

Four-point linear probe:

The electrical conductivity was determined according to **Equation S1**:[7]

$$\sigma = \frac{I}{V} \times \frac{1}{2\pi sF}$$

where σ is the conductivity (in S/cm), I is the current (in A), V is the voltage (in V), s is the spacing between the probes (here 1.25 mm), and F is a unitless correction factor for the diameter and thickness of the pellet.

The electrical conductivity of DC-103 on a pellet of thickness 0.47 mm and diameter 6 mm, measured using a four-point probe, was determined to be 7.6 ± 2.6 × 10^{-4} S cm^{-1}.

Two-point probe:

Current-Voltage (I-V) curves were collected using a two-point probe on a pressed pellet of DC-103 of thickness 1.1 mm and diameter 6.05 mm (**Figure S25**). The electrical conductivity was then obtained similar to the calculations of **Equation S1** to be 3.6 ± 0.1 × 10^{-5} S cm^{-1} in ambient atmosphere. The pellet was then placed in a home-made saturated humid chamber for around 2 days, in which the electrical conductivity at 98% relative humidity (RH) was measured again with an obtained value of 1.3 ± 0.3 × 10^{-5} S cm^{-1}, mostly unchanged compared to its value at ambient atmosphere.

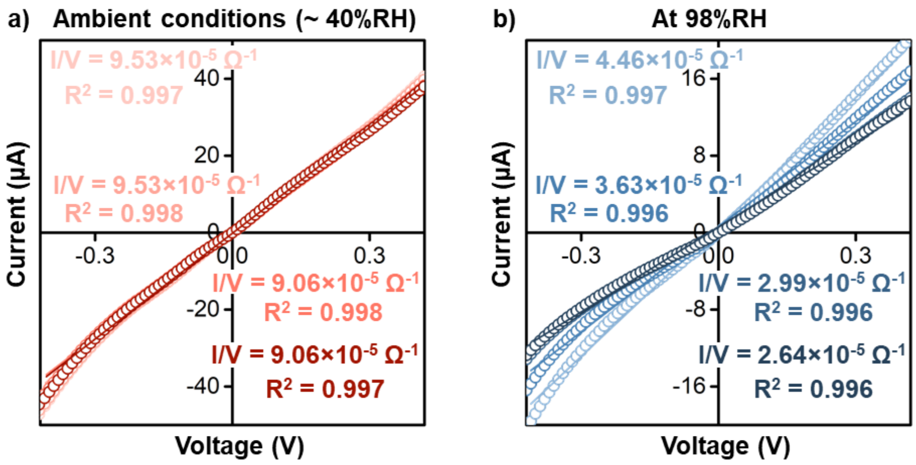

Figure S25. I-V curves of two-point probe measurements performed on DC-103 pellets a) at ambient room temperature conditions and b) at saturated humidity of 98% RH.

S4.8.2. Proton conductivity

Proton conductivity measurements were collected, using a two-point probe in the saturated (98% RH) humidity home-made chamber described above, at different temperatures ranging from 303 to 333 K. **Figure S26** shows the obtained Nyquist plots as well as the temperature-dependence change in proton conductivity, from which the proton conductivity value was determined to be around 2.0 × 10^{-6} S cm^{-1} at ambient temperature, and the activation energy was calculated as 0.61 eV.

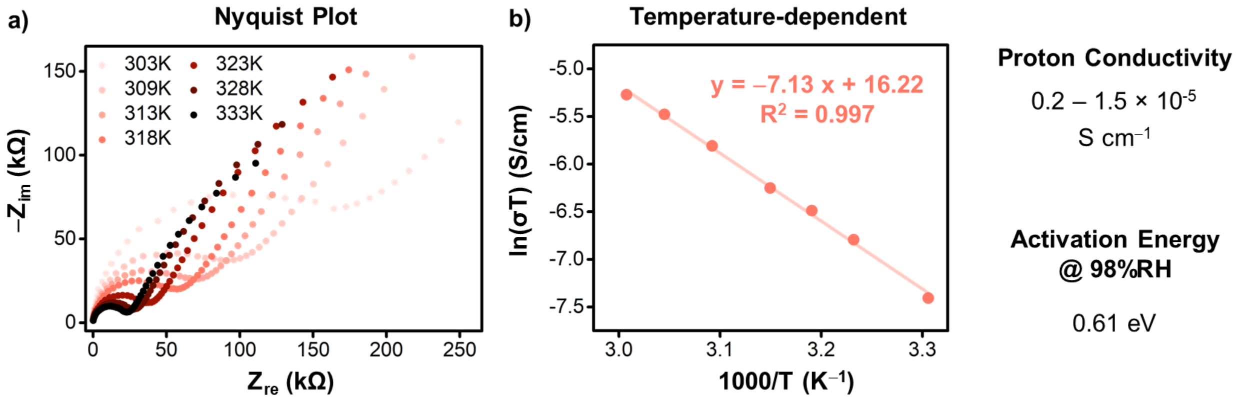

Figure S26. a) Nyquist plot of DC-103 at temperatures ranging between 303 and 333K, and b) temperature-dependent conductivity calculations, allowing for the determination of proton conductivity values ranging between 0.2 and 1.5 × 10^{-5} S cm^{-1} across the temperature ranges and an activation energy of 0.61 eV in a saturated (98% RH) humid environment.

S5. Computational details

S5.1. Model Building and Reactive Molecular Dynamics Simulations

We used classical reactive molecular dynamics (RMD) simulations, based on a previously tuned force field[8, 9] containing all the appropriate parameters for the DC-103 MOF system, to reproduce more realistically (at the sub-100 nanometer scale in a wet environment) structure, dynamics of a MOF, of the loaded species (i.e., solvent molecules, oxygen, nitrogen, and gaseous pollutants) and their interactions/reactions with all the framework sections located in the bulk or at the exposed surfaces.

We started the calculations by optimizing an atomistic model of the CoTPz+Cu subunit at the density functional theory level (DFT) using the PBE functional implemented in the Quantum Espresso (QE) package,[10] and the generalized gradient approximation (GGA) with the Perdew–Burke–Ernzerhof (PBE) functional,[11] Projector Augmented-Wave (PAW) pseudopotentials[12] and Grimme-D3 Dispersion corrections.[13] Wave-function and charge-density cutoffs of 60 and 600 Ry were employed for the plane-wave basis sets. Single-particle wave functions were calculated spin-unrestricted by applying a smearing of the one-particle levels of 0.002 Ry. Periodic boundary conditions were used in all directions. The cell lattice constants were optimized using the variable-cell algorithm.

The force field (FF) representation of the subunit was tested against the DFT-optimized geometry, verifying that that configuration was satisfactorily reproduced (we obtained a maximum root-mean-square deviation of all atoms of about 0.08 Å).

Then, a multilayered (eight-layer) supramolecular model of the DC-103 system was created by replicating the DFT-optimized subunit four times in the x and y directions (resulting in an approximately 10 x 10 nm^2 square system) to remove the bias introduced by periodic boundary conditions. All these choices were based on preliminary tests carried out in earlier investigations.[14, 15]

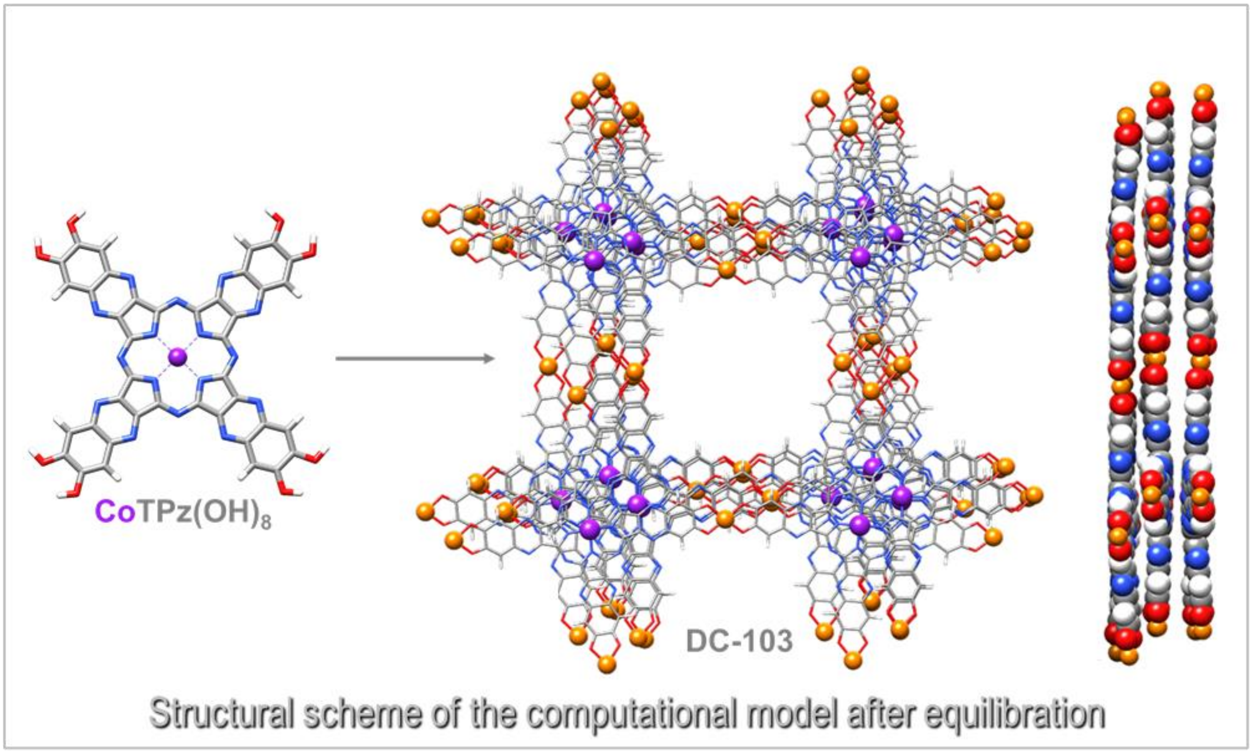

Scheme S3. Structural scheme of the computational model after equilibration

S5.2. Quantum Chemistry calculations: Structural and electronic properties

To investigate different stacking modes at the DFT level of theory, we employed unit cells consisting of a variable number of layers, ranging from two to four, along the *z* direction, with an average interlayer spacing in the 0.3–0.4 nm range. The minimal replicated unit was retained along the *x* and *y* directions (in a range of 2.2–2.3 nm). Reciprocal-space sampling was limited to the Gamma point, as we verified that increasing the number of k points did not significantly affect the band structures of the systems under investigation.

The results obtained for several bulk morphologies, selected on the basis of our earlier experience and extracted from snapshots of the reactive dynamics during the production runs, are summarized in **Figure S27**, which shows two-layer unit cells, and in **Figure S28**, which displays four-layer unit cells. After cell shape and size optimization, we observed the spontaneous stabilization of a tilted structure (**Figure S27a**), with staggered stacking layers, as discussed in

the previous section. Orthorhombic unit cells were also built by imposing alternative stacking arrangements, as illustrated by the geometry shown in **Figure S27b**. In this case, the fully eclipsed stacking (**Figure S27c**) was significantly unstable, whereas the extreme stacking arrangement (**Figure S27d**), corresponding to a partial closure of the MOF channels, had the highest-energy morphology. Analogous stacking modes were also generated for thicker unit cells, as displayed in **Figure S28**. For these structures, the energy differences per layer decreased in both staggered and eclipsed configurations, reflecting slight internal rearrangements that further stabilize the system by allowing greater structural freedom. In particular, the geometries shown in **Figure S28a,b** are the characteristic morphologies of a dynamic MOF model. Indeed, these configurations were sampled during the MD simulations and were therefore selected as prototype models for investigating electronic and sensing properties at the DFT level. Overall, the relatively small energy differences among the explored morphologies highlight the fluxional character of this MOF. This behavior is consistent with previous findings for the Cu-HHTP system[8] and can be attributed to the material nature.

All the structural models considered in this work feature nearly square unit cells in the (x,y) plane with lattice parameters of approximately 2.3 nm, in good agreement with the experimental characterization of the material. In the lowest-energy morphologies, the interlayer distances between metal centers (both Co and Cu) range from about 3.6 to 4.0 Å. At the electronic level, DFT describes the system as magnetic, with one unpaired electron per metal center. This result is consistent with the formal Cu^{2+} and Co^{2+} oxidation states and agrees with the reported XPS characterization. Analysis of the spin-density distribution shows that the magnetization arises mainly from *d*-type electrons localized on the metal centers, with a non-negligible contribution from the *p*-type orbitals of the oxygen atoms coordinated to the Cu centers (**Figure S29**).

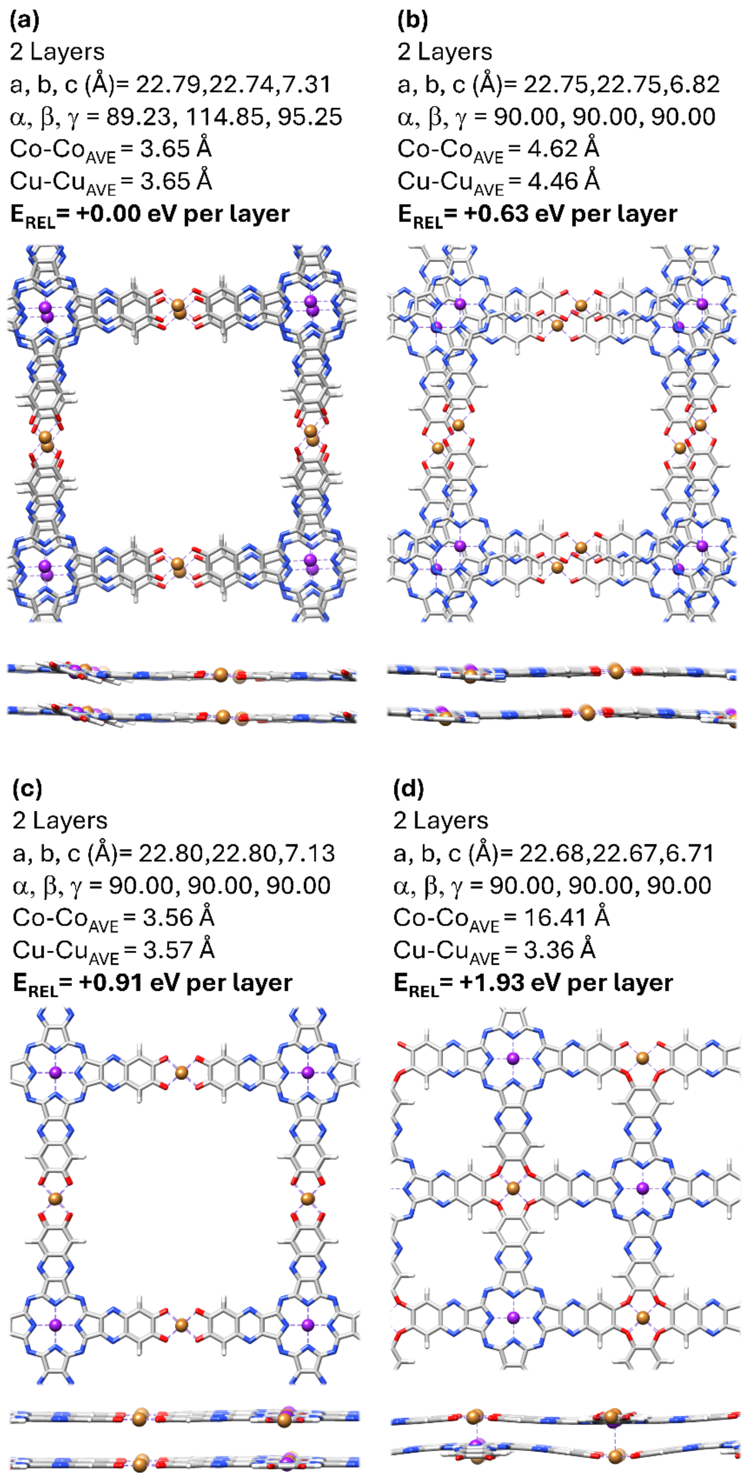

Figure S27. a-d) Selected two-layer morphologies explored for the DC-103 system at the DFT level. For each configuration, the DFT-optimized lattice parameters (cell-axis lengths), unit-cell angles (also DFT-optimized when different from 90 degrees), average interlayer distances between metal centers, and relative stabilities (in eV) are indicated. Color code: C gray, H white, O red, N blue, Cu orange, and Co purple.

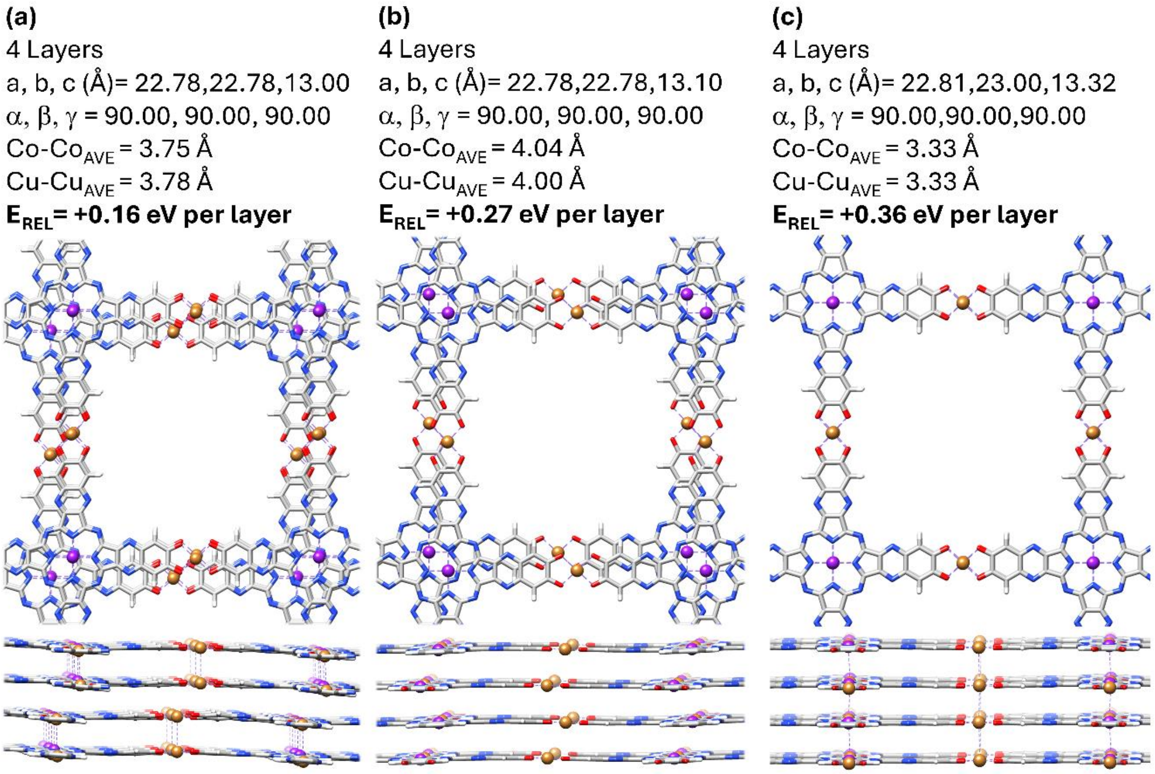

Figure S28. a-c) Selected four-layer morphologies explored for the DC-103 system at the DFT level. For each configuration, the DFT-optimized lattice parameters (cell-axis lengths), unit-cell angles, average interlayer distances between metal centers, and relative stabilities (in eV) are indicated. Color code: C gray, H white, O red, N blue, Cu orange, and Co purple.

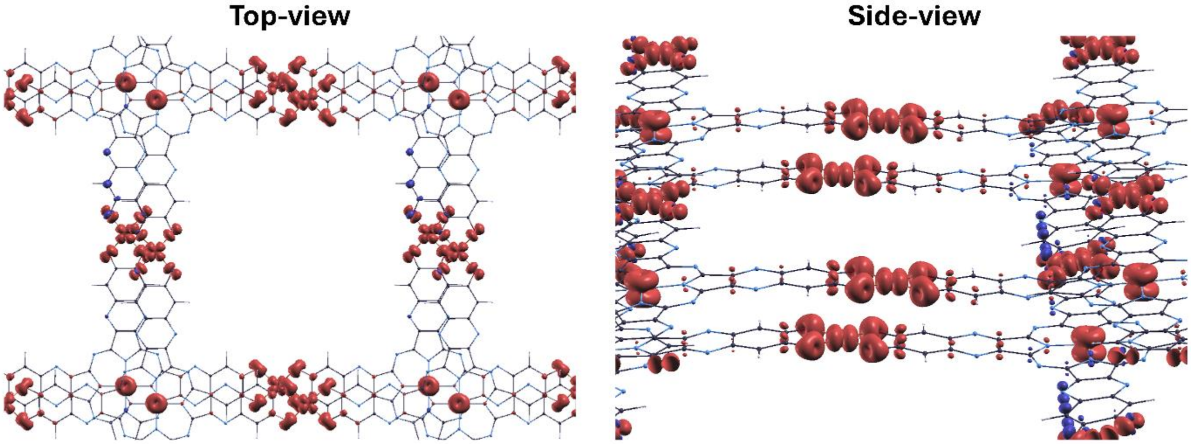

Figure S29. Top and side-view of the spin-density of the DC-103 structure depicted in **Figure S28b**. Magnetization arises mainly from *d*-type electrons localized on the metal centers, with a non-negligible contribution from the *p*-type orbitals of oxygen atoms coordinated to the Cu centers. Color code: C gray, H white, O red, N blue, Cu orange, and Co purple.

S6. SO_2 Sensing experiments of DC-103

S6.1. Sensing experiments and calculations

S6.1.1. Preparation of chemiresistive devices

A suspension of DC-103 was prepared by sonicating 1 mg of the MOF in 1 mL of Milli-Q water for around 1.5 to 2 hours. PXRD spectra of the sonicated MOF were recorded to confirm the retention of its crystallinity, as shown in **Figure S30**. Of each suspension, 25 µL were drop-casted onto 10-µm gold interdigitated electrodes. The electrodes were allowed to dry under vacuum for 2 hours to get resistance values in the range of 0.1-40 kΩ.

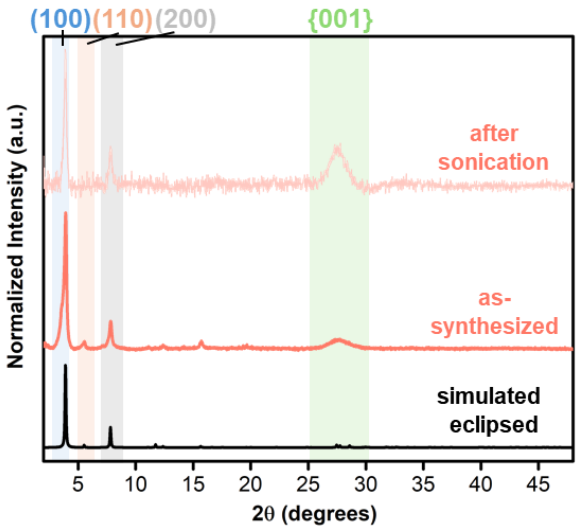

Figure S30. PXRD traces of the as-synthesized DC-103 and after sonication for around 2 hours.

Linear sweep voltammetry (current-voltage, or I-V) measurements of the fabricated DC-103 devices exhibited linear Ohmic characteristics of the deposited material (**Figure S31**), with extracted resistance values ranging from 0.14 to 0.45 kΩ. I-V curves under different background gases were achieved by placing the electrodes in a Teflon chamber in the sensing setup described in **Section S6.1.2**.

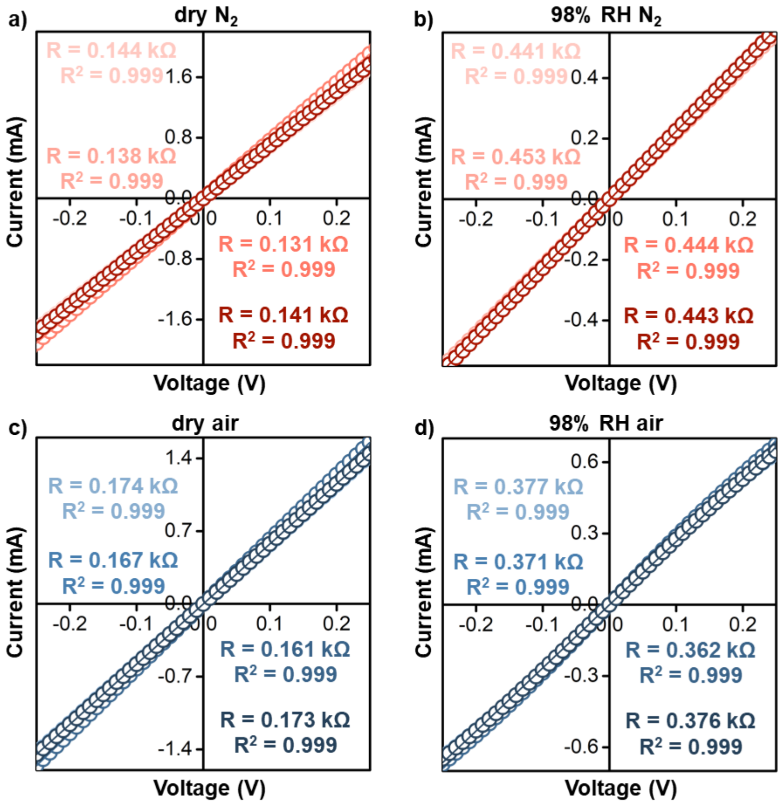

Figure S31. I-V curves of fabricated four replicate DC-103 devices in a) dry N_2, b) 98% RH N_2, c) dry air, d) 98% RH air.

S6.1.2. Description of sensing setup

A home-built setup, illustrated in **Figure S32**, is used for the sensing experiments. Analyte gases were purchased in 10,000 ppm concentrations (1% analyte gas in dry N_2) and diluted in dry nitrogen (N_2) gas, compressed air (termed as dry air), or a certain % RH of N_2 or air, to allow the elution of the desired concentration of gas analyte. Briefly, a low-flow mass flow controller (MFC) connected to the analyte gas tank would deliver the desired amount needed, say 2 mL min^{-1}, and the latter will then get premixed with background gas delivered through a high-flow MFC, say at 0.5 L min^{-1}, before reaching the Teflon chamber, where our electrodes are placed, with a resulting diluted concentration of 40 ppm. The Teflon gas chamber creates a good seal around the electrodes with an inlet of the diluted gas and an outlet when ready for measurement.

For sensing under humid conditions, the desired background gas flow rate was directly humidified through the KIN-TEK Analytical FlexStream™, and the percentage relative humidity (% RH) values were in the error range of ± 3%.

The prepared electrodes are placed within a breadboard, with electrical connections supplied to a potentiostat from which the voltage is supplied (**Figure S32**).

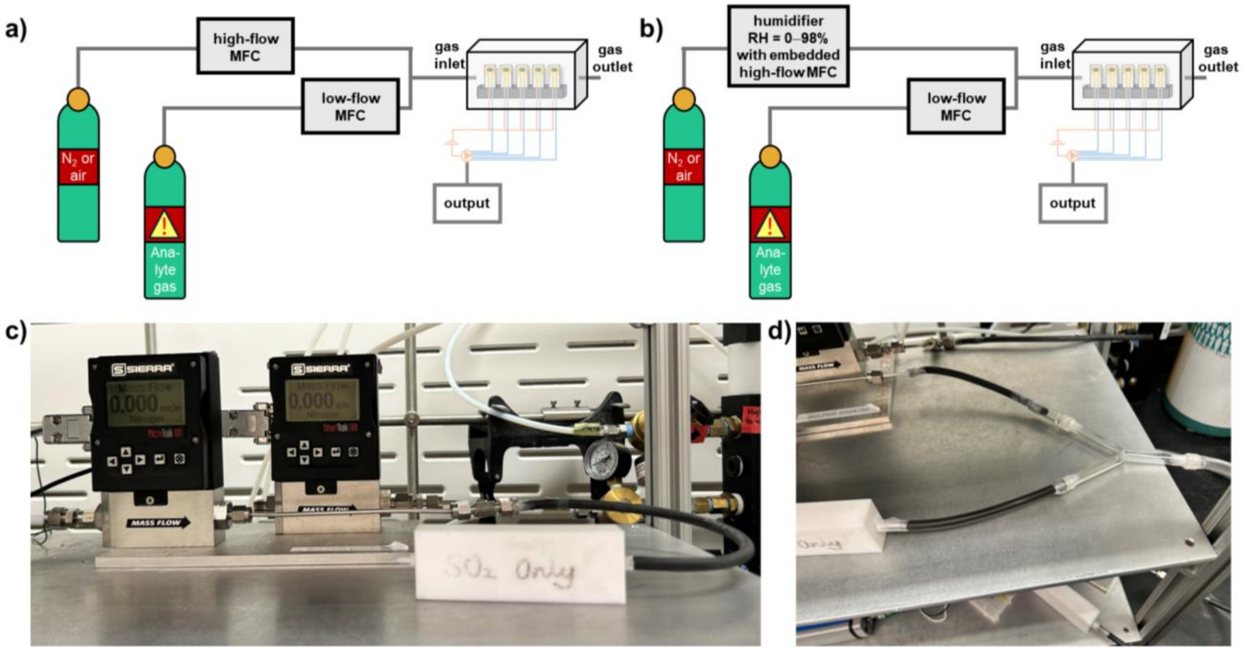

Figure S32. A schematic illustration of the chemiresistive sensing setup for experiments a) under dry conditions and b) under humid conditions. Images of the connections between the mass flow controllers and the Teflon gas sensing chamber a) under dry conditions and b) under humid conditions.

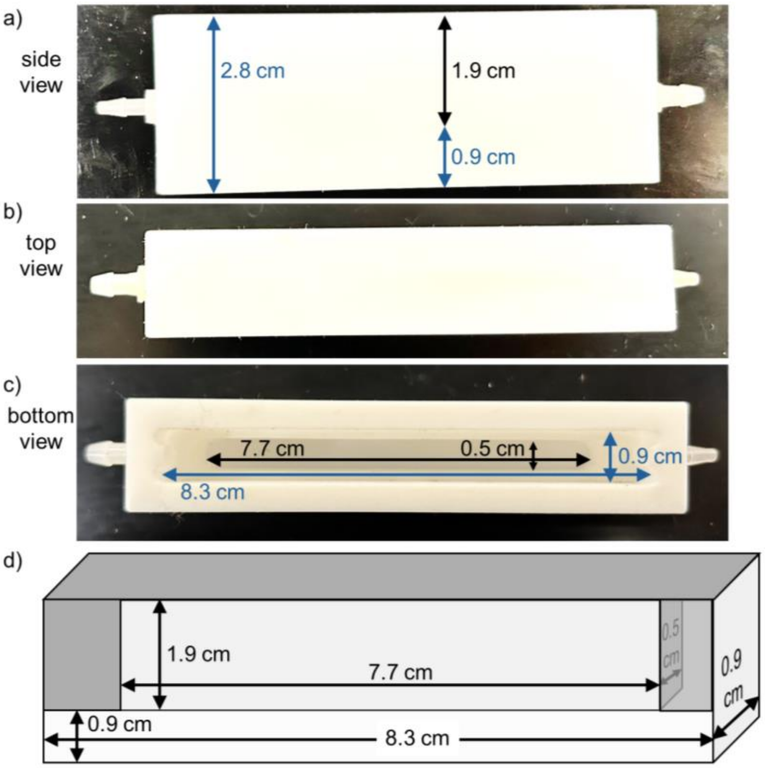

Figure S33. Photographs of different sides: a) side, b) top, and c) bottom views, and d) illustration of the Teflon chamber used for sensing experiments showing its size and distances in its grooves, allowing to calculate the open volume of the chamber as 14.038 cm^3.

Illustrations of the Teflon chamber are shown in **Figure S33**, from which we deduced the volume that surrounds the devices during the sensing experiments.

Total volume of Teflon chamber = volume of inner portion + volume of exposed outer portion

Volume of inner portion = length × width × height = 1.9 × 7.7 × 0.5 = 7.315 cm^3

Volume of exposed outer portion = length × width × height = 0.9 × 8.3 × 0.9 = 6.723 cm^3

Total volume of Teflon chamber = 7.315 + 6.723 = 14.038 cm^3

S6.1.3. Sensing experimental conditions

In the gas sensing experiments, the electrodes are first equilibrated with a stream of background gas for around 15 minutes to ensure a straight baseline prior to recording. Each recorded measurement includes a baseline of background gas flow, followed by 10 minutes exposure to the analyte gas at the desired concentration, and a recovery period where the analyte mass flow controller is switched off, and the electrodes are only subjected to the background gas. All sensing experiments of DC-103, including in dry/humid (0–98% RH) nitrogen/air environments, were performed at a constant driving voltage of 0.1 V and at room temperature (without the use of any external heating appliances). The output obtained from the software is in the form of changes in current with time.

The change in current of different electrodes was normalized according to **Equation S2**, where $-\Delta G/G_0$ (in %) is the normalized change in conductance, also referred to as normalized response, I (in μA) represents the current at a certain time, and I_0 (in μA) is the initial current at 10 min directly before gas exposure.

Equation S2: $$-\Delta G/G_0 = -\frac{I - I_0}{I_0} \times 100$$

Recovery calculations were performed for the MOFs towards the different gases at the tested concentrations according to the following equation:

Equation S3: $$\%\, Reversibility = \frac{I_{max} - I_{after\ recovery}}{I_{after\ recovery}} \times 100$$

where I_{max} is the current at maximum response (mainly at 20 minutes, i.e. after 10 minutes of exposure) and $I_{after\ recovery}$ is the current at the end of the recovery period (at 40 minutes, i.e. after 20 minutes of recovery).

Cyclic sensing experiments were performed by exposing the same electrodes to the specific concentration of gas multiple times, between which the devices were purged with the background

gas. In brief, after equilibrating the chemiresistive devices with the background gas at the target flow rate (high-flow MFC: 0.5 L min^{-1}), they were exposed to 10 ppm of analyte gas (low-flow MFC: 0.5 mL min^{-1}) for 10 minutes. The low-flow MFC is then set back to zero, while the high-flow MFC remains at 0.5 L min^{-1}, during which the devices are allowed to recover for 30 minutes or one hour without removing the Teflon chamber (**Figure S32**). Subsequent cycles are then performed similarly through several exposure-recovery cycles as described above.

S6.1.4. Power consumption calculations

To provide a quantitative description of the power consumption used in a sensing experiment, we performed calculations, according to the following formula: Power (in Watts) = Voltage (in Volts) × Current (in Amperes). Considering the applied driving voltage of 0.1 V and the average current readings of DC-103 devices of 0.72, 0.22, 0.59, and 0.28 mA in dry N_2, 98% RH N_2, dry air, 98% RH air, respectively, at 0.1 V (extracted from the I-V curves shown above), we can calculate an approximate consumption of power as shown in **Table S3**. Previous literature on the power consumption of gas sensors have classified sensors of lower than 100 mW into the following categories: low power (1–100 mW), ultralow power (<1 mW), and self-powered (~0 mW external power consumption).[16] Based on established classification of power consumption by gas sensors in the literature, DC-103 devices (Power ~0.072 mW) exhibit ultralow power efficiency, owing to their room temperature and low driving voltage operation, owing to the high conductivity of DC-103.

Table S3. Power consumption calculations of DC-103 devices in different conditions.

Environment	Driving voltage	Current	Power consumption
Dry N_2	0.1 V	0.7197 mA	0.072 mW
98% RH N_2	0.1 V	0.2242 mA	0.022 mW
Dry air	0.1 V	0.5908 mA	0.059 mW
98% RH air	0.1 V	0.2796 mA	0.028 mW

S6.1.5. Limits of Detection (LOD) calculations

Calculation of Theoretical LODs

LODs were calculated using two methods:

- <u>5th order polynomial fit method:</u>

LODs using this method were determined according to the calculations described by Ammu *et al.*[17] Briefly, we fitted the values of the equilibrated baseline before exposure (normalized conductance values between 0 and 10 minutes) to a fifth order polynomial equation. We then determined the sum of the squared residuals (V_{x^2}) by adding the squared values of the difference between the fitted and measured response values (**Equation S4**).

Equation S4: $V_{x^2} = \sum(y_i - y)^2$, where y_i values correspond to the measured data points and y values correspond to those extracted from the curve-fitting equation.

Subsequently, the root mean square of the noise (rms_{noise}) is calculated using **Equation S5**.

Equation S5: $rms_{noise} = \sqrt{\frac{V_{x2}}{N}}$, where N refers to the number of data points used in the curve fitting.

LOD values are then calculated according to **Equation S6** using the slopes of the linear concentration-dependent responses determined at different exposure times.

Equation S6: $LOD_{polynomial\ fit} = 3 \times \frac{rms_{noise}}{slope}$

- <u>Noise-based 3σ method</u>:

LODs using this method were determined based on the standard deviation of the equilibrated baseline before exposure. Briefly, we computed the standard deviation (σ_{noise}) of experimental values from a 5-minute linear portion of the baseline. We then calculated the LOD values

according to **Equation S7** using the slopes of the linear concentration-dependent responses determined at different exposure times.

Equation S7: $LOD_{noise-based} = 3 \times \frac{\sigma_{noise}}{slope}$

Discussion on LODs

Theoretical LODs calculated using the methods described above assume that the linear relationship between sensor signal and analyte concentration extends from the experimentally-studied concentration range into the calculated ppb-range of LODs. For the ppm-level concentrations investigated in this work, the gap between the experimental sensing range and the extrapolated theoretical LODs spans approximately one to three orders of magnitude. Though this extent of extrapolation is within the two to five orders of magnitude range of literature precedent,[18] this extrapolation assumption may not be accurate due to possible differences in linearity and slopes across ppm-to-ppb level spans.

To improve the accuracy and credibility of the LOD values, we performed additional sensing experiments at ppb-level concentrations and calculated LODs based on ppb-to-ppb extrapolations, rather than ppm-to-ppb extrapolations. The chemiresistive response of DC-103 towards SO_2 in dry air remained robust at ppb-level concentrations, exhibiting a linear concentration-dependent response over the range of 50 to 250 ppb. The corresponding LODs calculated using the two methods described above showed minimal variation.

Several experimental constraints should be noted that may contribute to discrepancies between sensing responses measured in the ppm and the ppb regimes. First, ppm-level concentrations were generated using a 1% (10,000 ppm) SO_2 gas cylinder (balance N_2) diluted with a constant background gas flow of 0.5 L min^{-1}. Owing to the 2L min^{-1} maximum flow rate of the background gas MFC and the 0.1 mL min^{-1} minimum flow rate of the analyte gas MFC, this configuration does not permit access to low-ppb concentrations using the 1% gas tank. Consequently, a 0.1% (1000

ppm) SO_2 gas cylinder was employed to achieve ppb-level concentrations. Differences in cylinder sources and background flow rates may cause discrepancies in the resulting sensing responses. Second, sensing experiments could not be performed at the theoretically calculated LODs of 2–3 ppb due to limitations in MFC flow control. Constrained by the minimum analyte flow rate of 0.1 mL min^{-1} and the maximum background gas flow rate of 2 L min^{-1}, as described above, the lowest attainable analyte concentration was 50 ppb. Notably, such low analyte flow rates fall within the ±1% instrumental error of the MFC (± 0.04 mL min^{-1}), which may introduce additional uncertainty in gas delivery at these limits.

S6.1.6. Initial rate calculations

Initial rates were determined from the slope of a linear fit to the initial response of DC-103 towards SO_2 at each concentration. In most cases, the fit was performed over the first minute of exposure. However, when the response deviated from linearity over this interval, only the initial linear region was used.

S6.1.7. Response/recovery time calculations

Response times were defined as the elapsed time from the onset of gas exposure required to reach 90% of the maximum response[19] of DC-103 at each SO_2 concentration.

Recovery times were defined as the elapsed time after termination of SO_2 exposure required for the response to return to 10% of the maximum response.[19]

S6.2. SO_2 sensing of DC-103

Since SO_2 is prone to oxidation into sulfur trioxide (SO_3) and sulfuric acid (H_2SO_4) under certain environmental conditions,[20, 21] we have carefully considered the stability of SO_2 under the various atmospheric conditions incorporated in this study. Based on previous reports in the literature, in the absence of a photocatalyst and irradiation, SO_2 has been proven relatively stable during the time of exposure (10 minutes) at room temperature and in the absence of sunlight or irradiation

under dry as well as low humid conditions.[22-24] Also, in relation to the sulfur cycle, the conversion of SO_2 into SO_3 in the presence of irradiation and oxygen would require hours or days, which can then be transformed into H_2SO_4,[25, 26] thus posed unlikely under the conditions of our conducted sensing experiments. At higher humidity levels, however, the possibility of SO_2 solvation into water vapor and the subsequent formation of sulfurous acid (H_2SO_3) increases,[27, 28] which may pose some differences in sensing trends or mechanisms. In particular, we considered the following **Equations S8 and S9** with reported Henry's Law Constant (K_H), corresponding equilibrium constant (K_a) and rate constant (k) values of SO_2 at room temperature (298.5 K).[27] In addition, we realize possible oxidation of H_2SO_3 to sulfite (SO_3^{2-}) species, which might be a valuable consideration when considering mechanistic insights (**Equation S10**).

Equation S8: $SO_{2\,(g)} + H_2O \rightleftharpoons H_2O \cdot SO_{2\,(aq)}$ K_H = 1.3 mol kg^{-1} bar^{-1}

Equation S9: $H_2O \cdot SO_{2\,(aq)} \rightleftharpoons HSO_3^- + H^+$ $k_1 = 3.6 \times 10^6$ s^{-1} and $K_{a1} = k_1/k_{-1} = 10^{-1.8}$ M

Equation S10: $HSO_3^- \rightleftharpoons SO_3^{2-} + H^+$ $k_2 \geq 1.9 \times 10^3$ s^{-1} and $K_{a2} = k_2/k_{-2} = 10^{-7.2}$ M

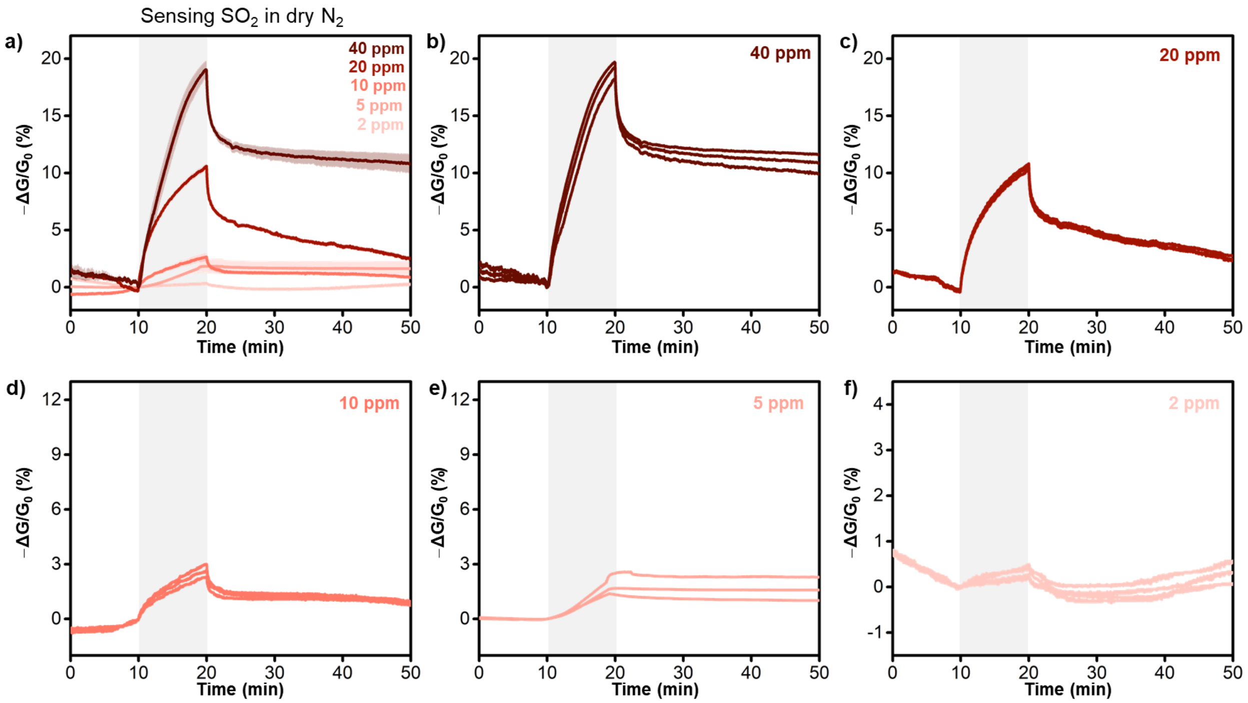

Figure S34. a) Averaged sensing responses of DC-103 towards SO_2 in dry nitrogen at different concentrations. The shaded curves represent the standard deviation of sensing responses from 3 devices and the gray shaded area represents the time of exposure of the devices to SO_2. Sensing responses of at least 3 devices of DC-103 towards b) 40 ppm, c) 20 ppm, d) 10 ppm, e) 5 ppm, and f) 2 ppm of SO_2.

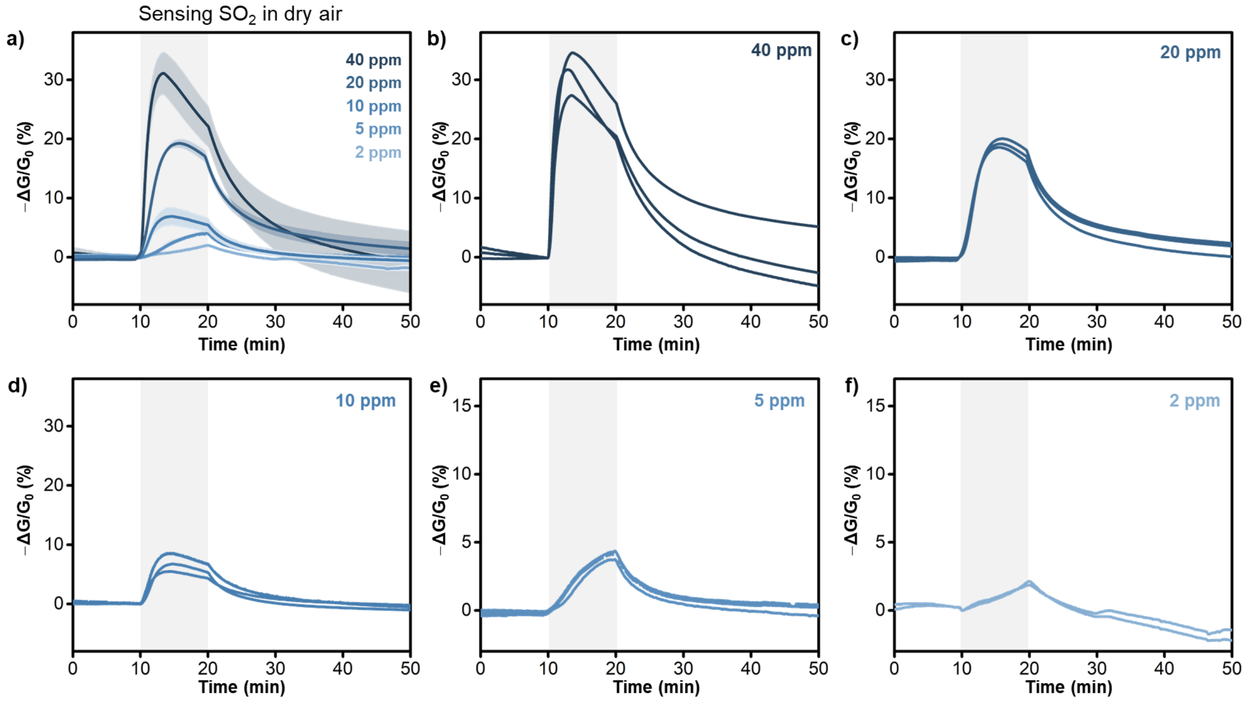

Figure S35. a) Averaged sensing responses of DC-103 towards SO_2 in dry air at different concentrations. The shaded curves represent the standard deviation of sensing responses from 3 devices and the gray shaded area represents the time of exposure of the devices to SO_2. Sensing responses of at least 3 devices of DC-103 towards b) 40 ppm, c) 20 ppm, d) 10 ppm, e) 5 ppm, and f) 2 ppm of SO_2.

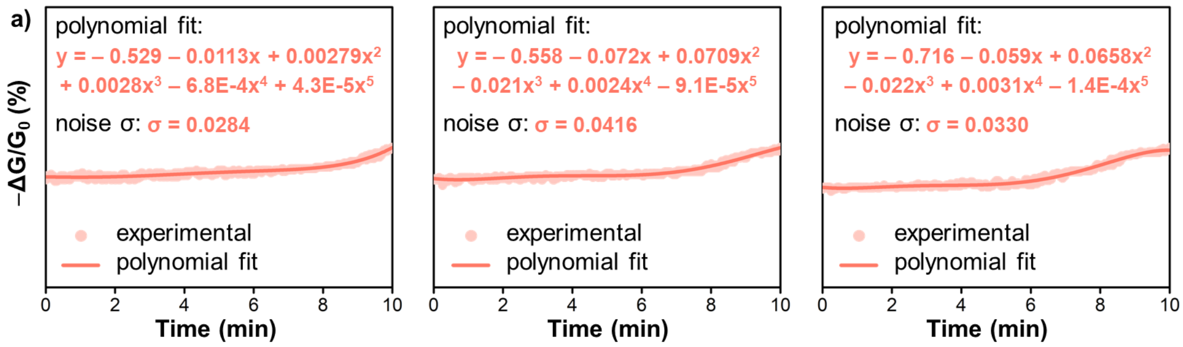

b)

Exposure time (min)	2	5
LOD from polynomial fit method	443 ± 23 ppb	221 ± 12 ppb
LOD from noise-based 3σ method	595 ± 116 ppb	297 ± 58 ppb

Figure S36. a) 5th order polynomial fits and standard deviation (noise σ) of the baseline from three different 2–40 ppm-level sensing responses of DC-103 towards SO_2 in dry N_2 conditions. b) Table summarizing the LOD values of DC-103 towards SO_2 in dry N_2 conditions, calculated using the 5th order polynomial fit (**Equation S6**) and noise-based 3σ (**Equation S7**) methods.

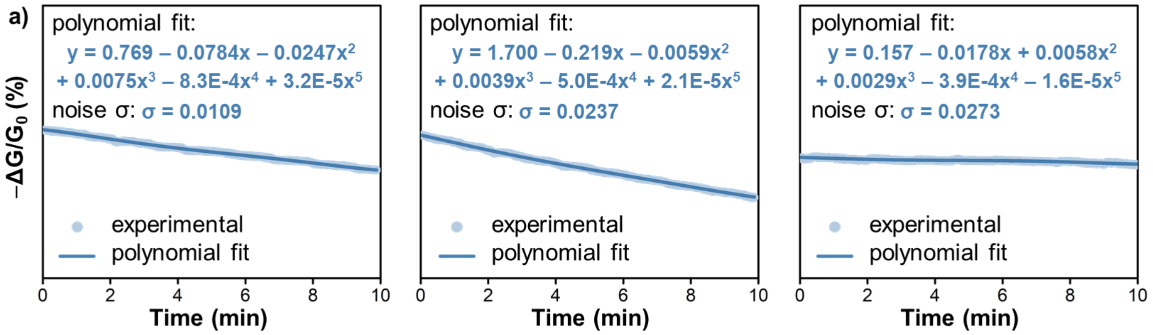

b)

Exposure time (min)	2	5
**LOD from polynomial fit method**	59 ± 12 ppb	57 ± 12 ppb
**LOD from noise-based 3σ method**	82 ± 34 ppb	79 ± 33 ppb

Figure S37. a) 5th order polynomial fits and standard deviation (noise σ) of the baseline from three different 2–40 ppm-level sensing responses of DC-103 towards SO_2 in dry air conditions. b) Table summarizing the LOD values of DC-103 towards SO_2 in dry air conditions, calculated using the 5th order polynomial fit (**Equation S6**) and noise-based 3σ (**Equation S7**) methods.

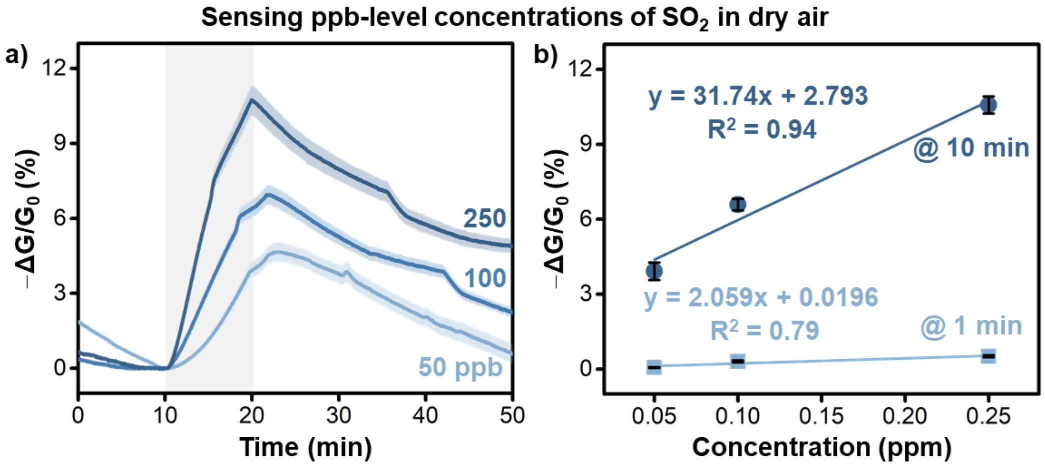

Figure S38. a) Averaged sensing responses of DC-103 towards SO_2 in dry air at 50, 100, and 250 ppb concentrations. The shaded curves represent the standard deviation of sensing responses from 3 devices and the gray shaded area represents the time of exposure of the devices to SO_2. b) Linear concentration-dependent response of DC-103 towards 50–250 ppb of SO_2 in dry air at 1 and 10 minutes of exposure.

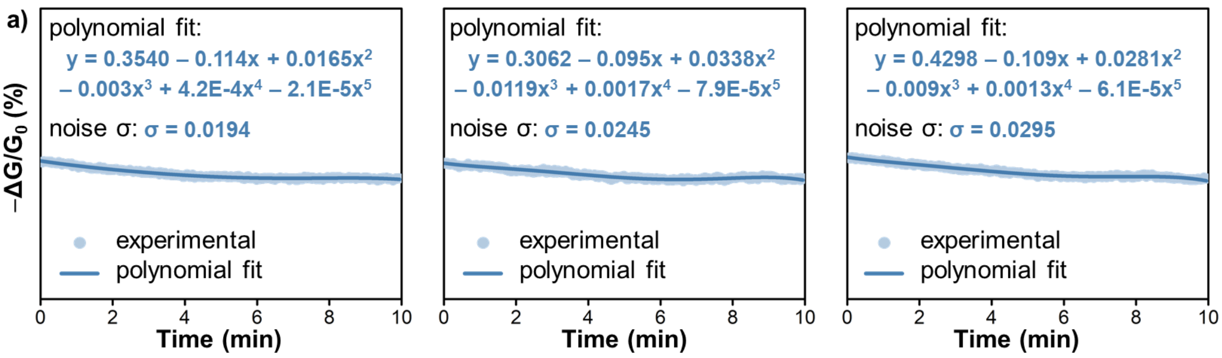

b)

Exposure time (min)	1	10
LOD from polynomial fit method	34 ± 8 ppb	2.2 ± 0.5 ppb
LOD from noise-based 3σ method	36 ± 7 ppb	2.3 ± 0.5 ppb

Figure S39. a) 5th order polynomial fits and standard deviation (noise σ) of the baseline from three different ppb-level sensing responses of DC-103 towards SO_2 in dry air conditions. b) Table summarizing the LOD values of DC-103 towards SO_2 in dry air conditions, calculated using the 5th order polynomial fit (**Equation S6**) and noise-based 3σ (**Equation S7**) methods extracted from ppb-level detection.

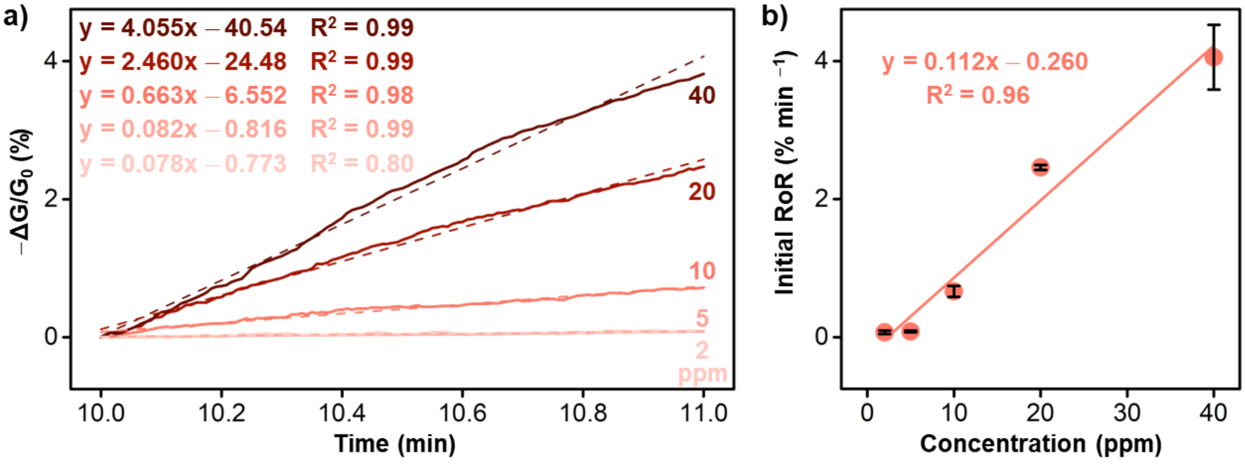

Figure S40. a) Averaged sensing responses of DC-103 across the first minute of exposure to different concentrations of SO_2 in dry nitrogen and their linear fits. b) Initial rates of responses (RoR) determined the slope of the linear fits, at different concentrations of SO_2 in dry nitrogen.

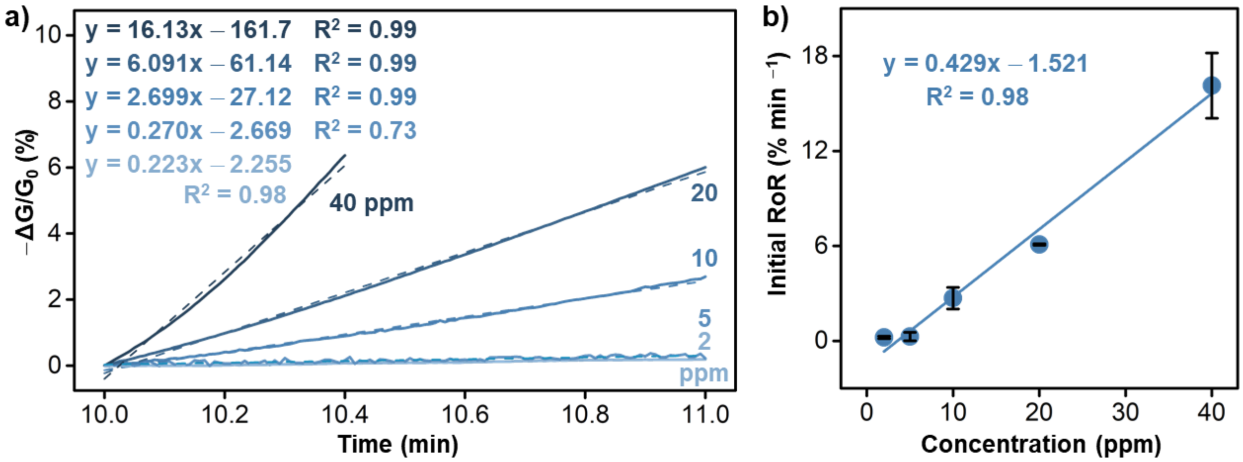

Figure S41. a) Averaged sensing responses of DC-103 across the first minute of exposure to different concentrations of SO_2 in dry air and their linear fits. b) Initial RoR determined the slope of the linear fits, at different concentrations of SO_2 in dry air.

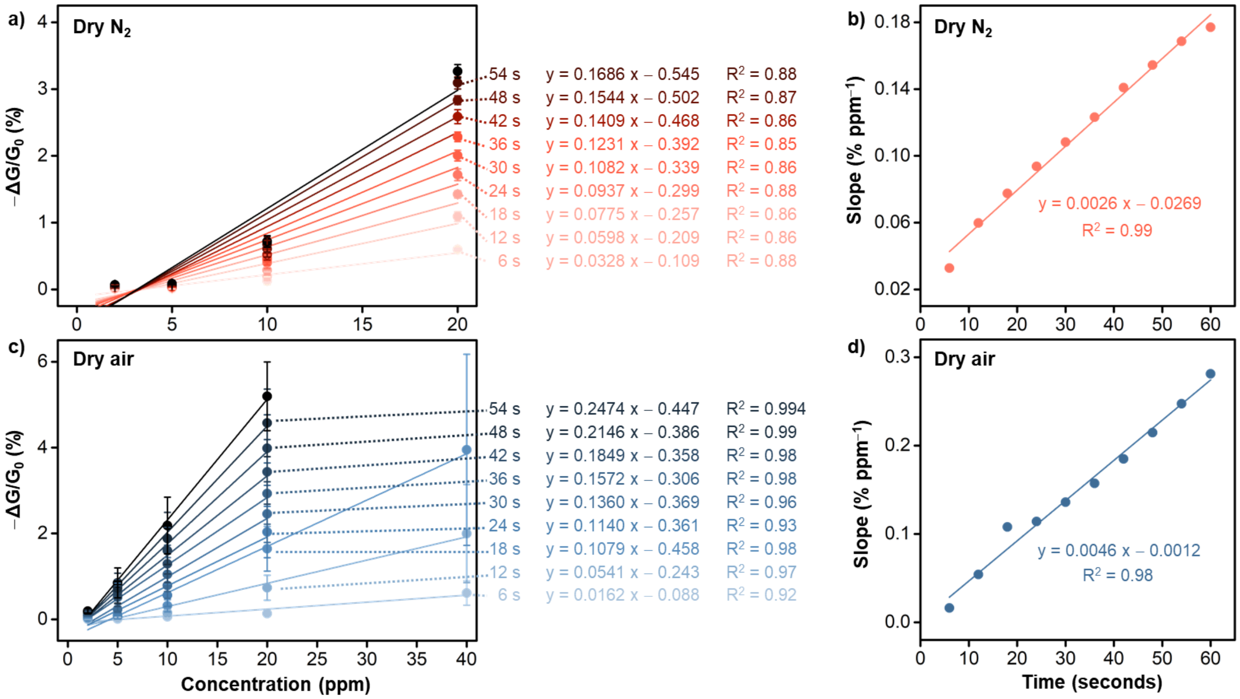

Figure S42. a) Concentration-dependent response of DC-103 towards 2–20 ppm of SO_2 in N_2 at 6, 12, 18, 24, 30, 36, 42, 48, and 54 seconds. b) Linear relationship between the slope of the plots in panel (a) with respect to time. c) Concentration-dependent response towards 2–40 ppm of SO_2 in air at 6, 12, 18, 24, 30, 36, 42, 48, and 54 seconds. d) Linear relationship between the slope of the plots in panel (c) with respect to time.

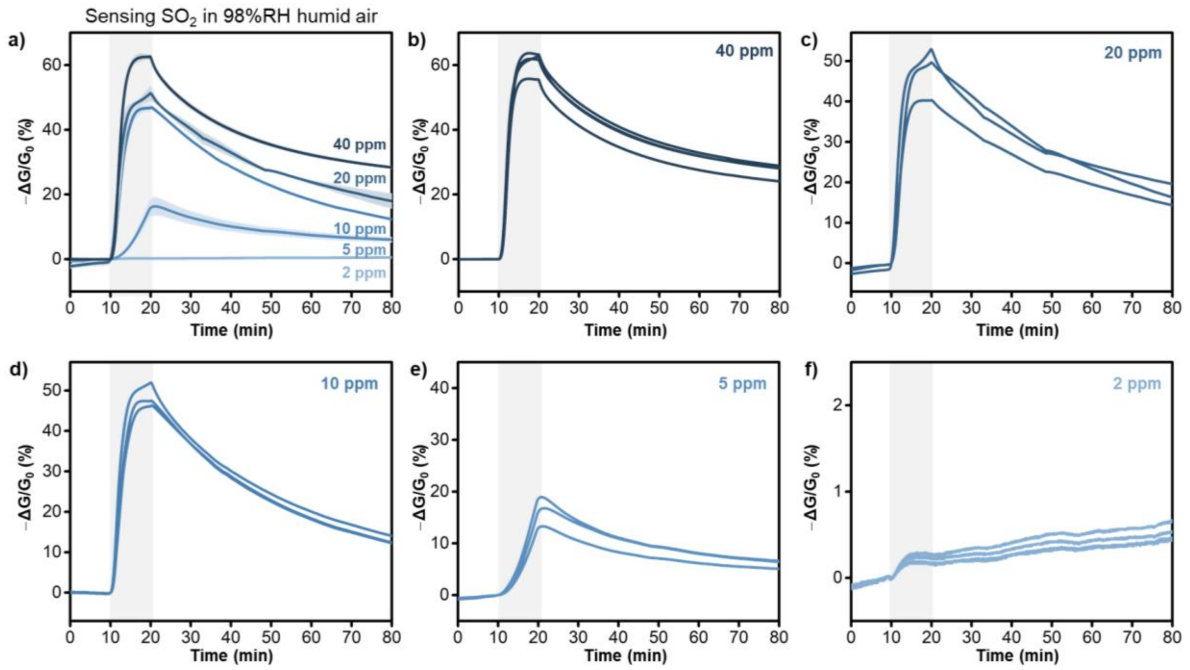

Figure S43. a) Averaged sensing responses of DC-103 towards SO_2 in 98% RH air at different concentrations. The shaded curves represent the standard deviation of sensing responses from 3 devices and the gray shaded area represents the time of exposure of the devices to SO_2. Sensing responses of at least 3 devices of DC-103 towards b) 40 ppm, c) 20 ppm, d) 10 ppm, e) 5 ppm, and f) 2 ppm of SO_2.

a)

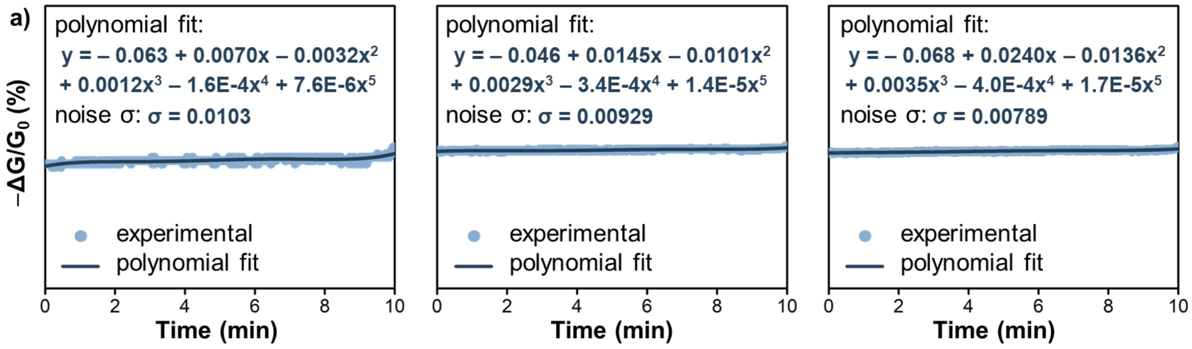

b)

Exposure time (min)	2	5
**LOD from polynomial fit method**	27 ± 3 ppb	3.1 ± 0.3 ppb
**LOD from noise-based 3σ method**	40 ± 5 ppb	4.7 ± 0.6 ppb

Figure S44. a) 5th order polynomial fits and standard deviation (noise σ) of the baseline from three different 2–40 ppm-level sensing responses of DC-103 towards SO_2 in 98% RH humid air conditions. b) Table summarizing the LOD values of DC-103 towards SO_2 in 98% RH humid air conditions, calculated using the 5th order polynomial fit (**Equation S6**) and noise-based 3σ (**Equation S7**) methods.

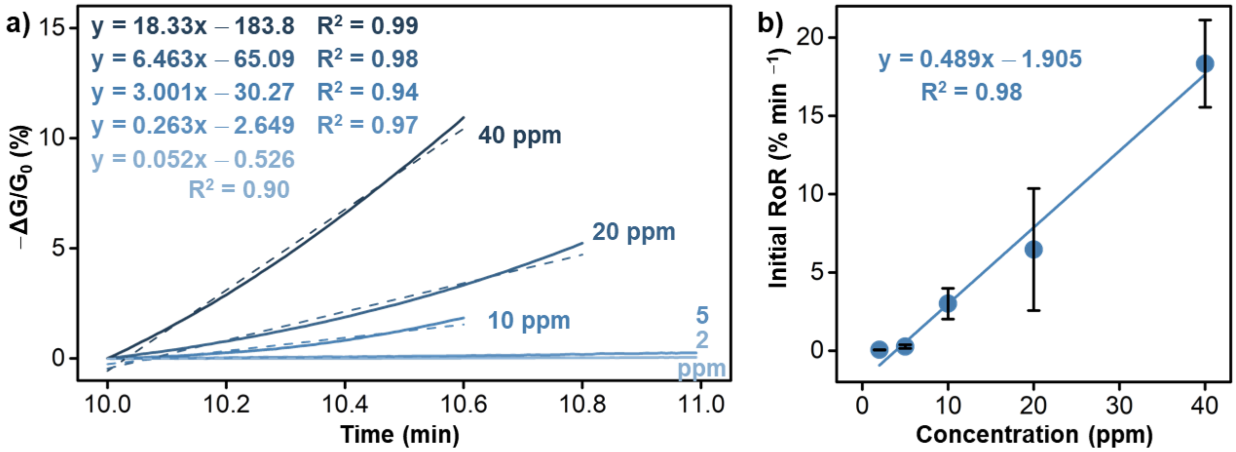

Figure S45. a) Averaged sensing responses of DC-103 across the first minute of exposure to different concentrations of SO_2 in 98% RH humid air and their linear fits. b) Initial RoR determined the slope of the linear fits, at different concentrations of SO_2 in 98% RH humid air.

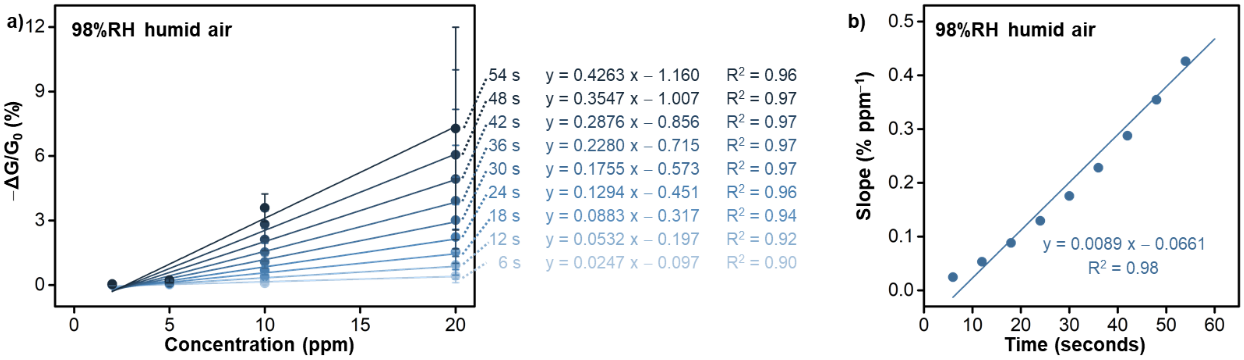

Figure S46. a) Concentration-dependent response of DC-103 towards 2–20 ppm of SO_2 in 98% RH humid air at 6, 12, 18, 24, 30, 36, 42, 48, and 54 seconds. b) Linear relationship between the slope of the plots in panel (a) with respect to time.

Table S4. Table summarizing the response times (in seconds) required to reach 90% of the maximum response at different concentrations of SO_2 in the different atmospheres.

SO_2 concentration	Dry N_2	Dry air	98%RH air
2 ppm	557 s	544 s	207 s
5 ppm	524 s	443 s	555 s
10 ppm	494 s	173 s	305 s
20 ppm	455 s	209 s	293 s
40 ppm	455 s	117 s	251 s

Table S5. Table summarizing the recovery times (in seconds) required to decrease to 10% of the maximum response at different concentrations of SO_2 in the different atmospheres.

SO_2 concentration	Dry N_2	Dry air	98%RH air
2 ppm	145 s	373 s	-
5 ppm	-	838 s	-
10 ppm	-	615 s	-
20 ppm	-	1496 s	-
40 ppm	-	873 s	-

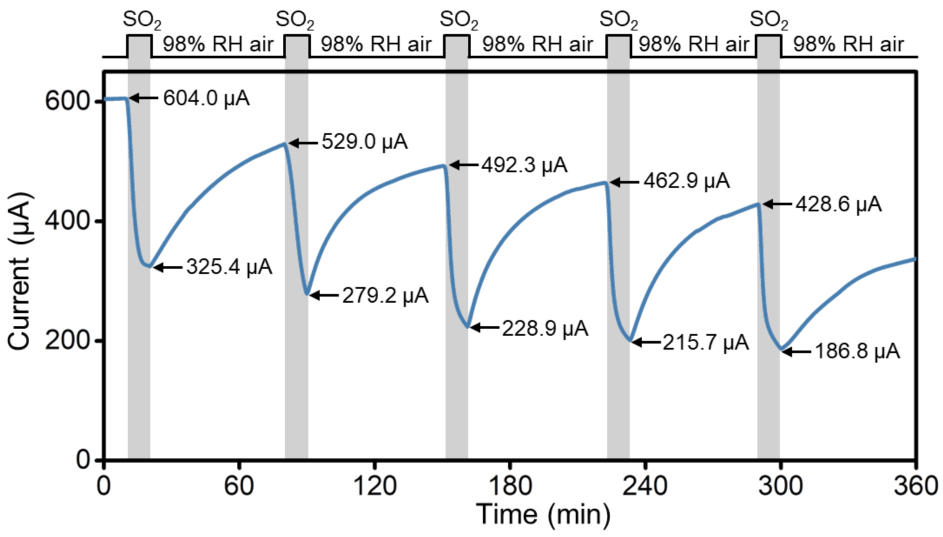

Figure S47. Raw data showing change in current with time upon exposure to 10 ppm of SO_2 in 98%RH humid air over 5 cycles. The data represented here are for one electrode out of the three triplicates whose average is shown in **Figure 3c**.

The absolute response values in **Figure 3c** appear to increase over successive cycles because the sensing response traces (shown in blue) are normalized to the initial baseline current prior to the first exposure. To determine the actual response magnitude for each individual cycle, we instead calculated the change in current relative to the baseline of that specific cycle. Accordingly, for the latter calculations, the value of I_0 in **Equation S2** was considered as the current at the initial point of each cycle rather than the initial current before the first cycle. The raw data and current values for one of the devices is depicted in **Figure S47**.

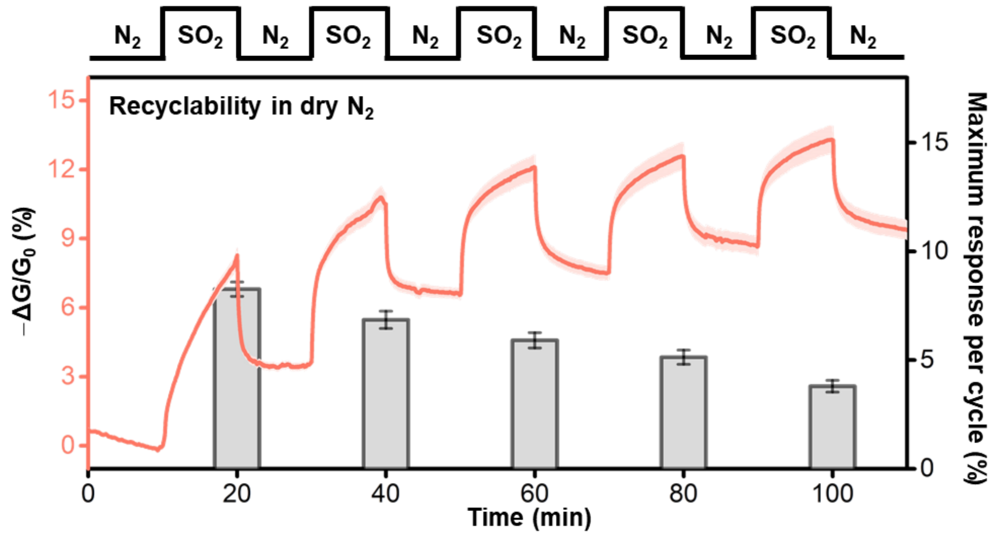

Figure S48. Curves of the averaged sensing responses of DC-103 with the standard deviation of three devices upon multiple exposure-recovery cycles towards 10 ppm of SO_2 in dry nitrogen, and bar graph showing the magnitude of the maximum response value across each cycle.

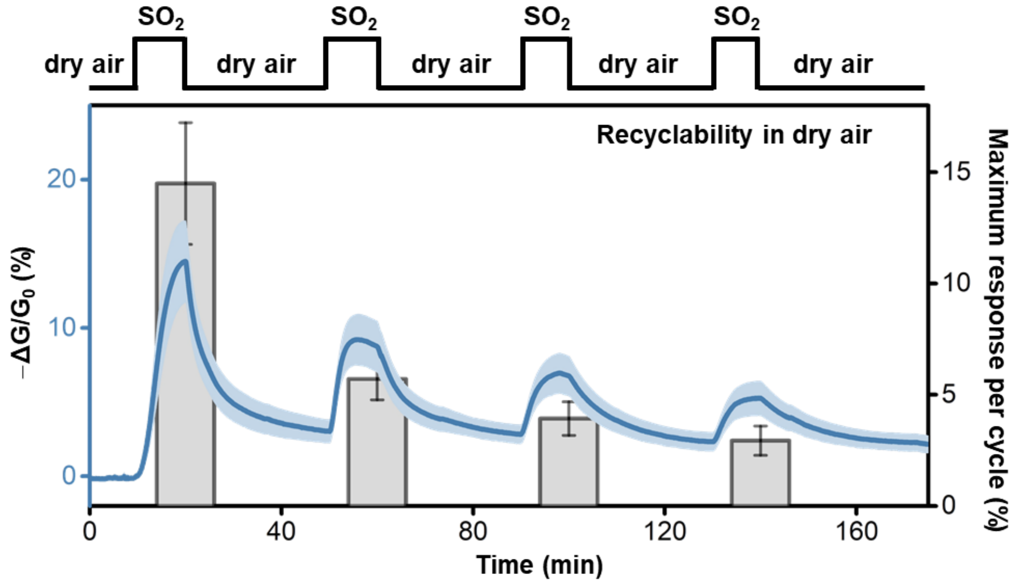

Figure S49. Curves of the averaged sensing responses of DC-103 with the standard deviation of three devices upon multiple exposure-recovery cycles towards 10 ppm of SO_2 in dry air, and bar graph showing the magnitude of the maximum response value across each cycle.

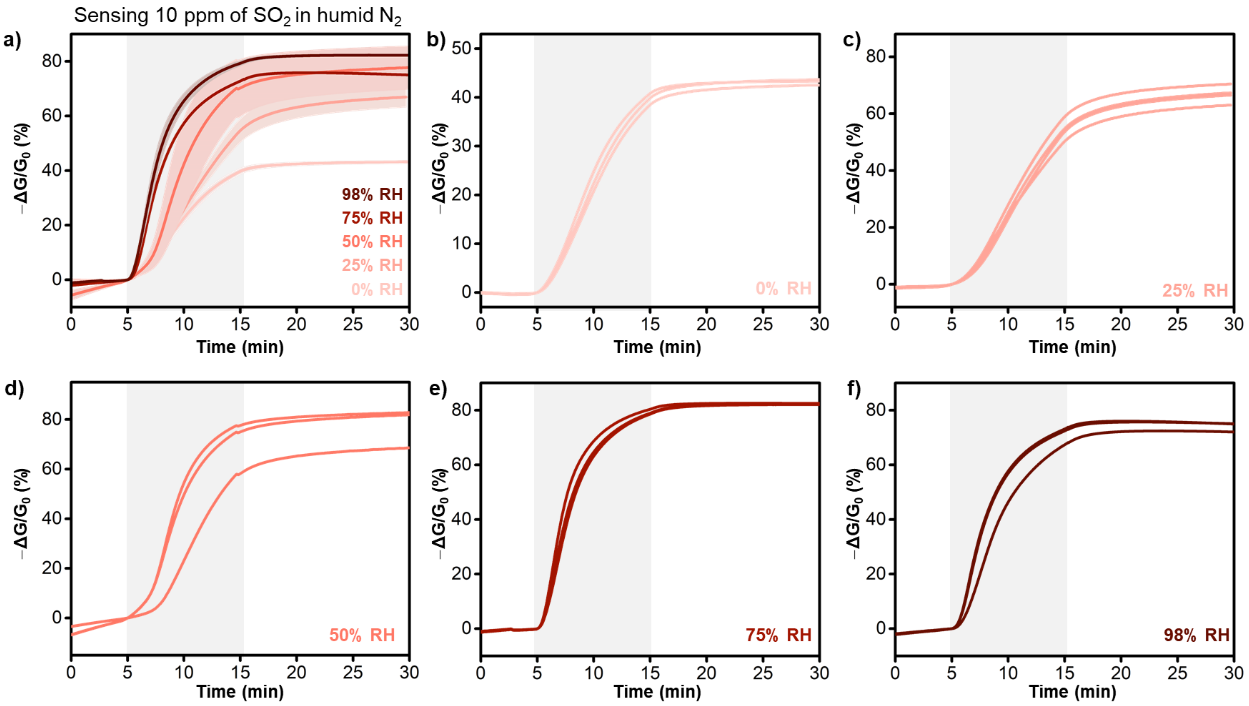

Figure S50. a) Averaged sensing responses of DC-103 towards 10 ppm of SO_2 in nitrogen at different % RH. The shaded curves represent the standard deviation of sensing responses from 3 devices and the gray shaded area represents the time of exposure of the devices to SO_2. Sensing responses of at least 3 devices of DC-103 towards b) 0% RH, c) 25% RH, d) 50% RH, e) 75% RH, and f) 98% RH in nitrogen.

Note: We noticed some discrepancies in the respective magnitudes of response achieved in dry conditions labeled dry N_2 or dry air (**Figure 2a-b**) and those labeled 0% RH N_2 or 0% RH air (**Figure 3d**), attributed to a change in the source of the background gas from direct house N_2 or air flow to an indirect flow where the background gas stream first passes through the humidity vapor generator. The differences between both gas stream connections are shown in **Figure S32**.

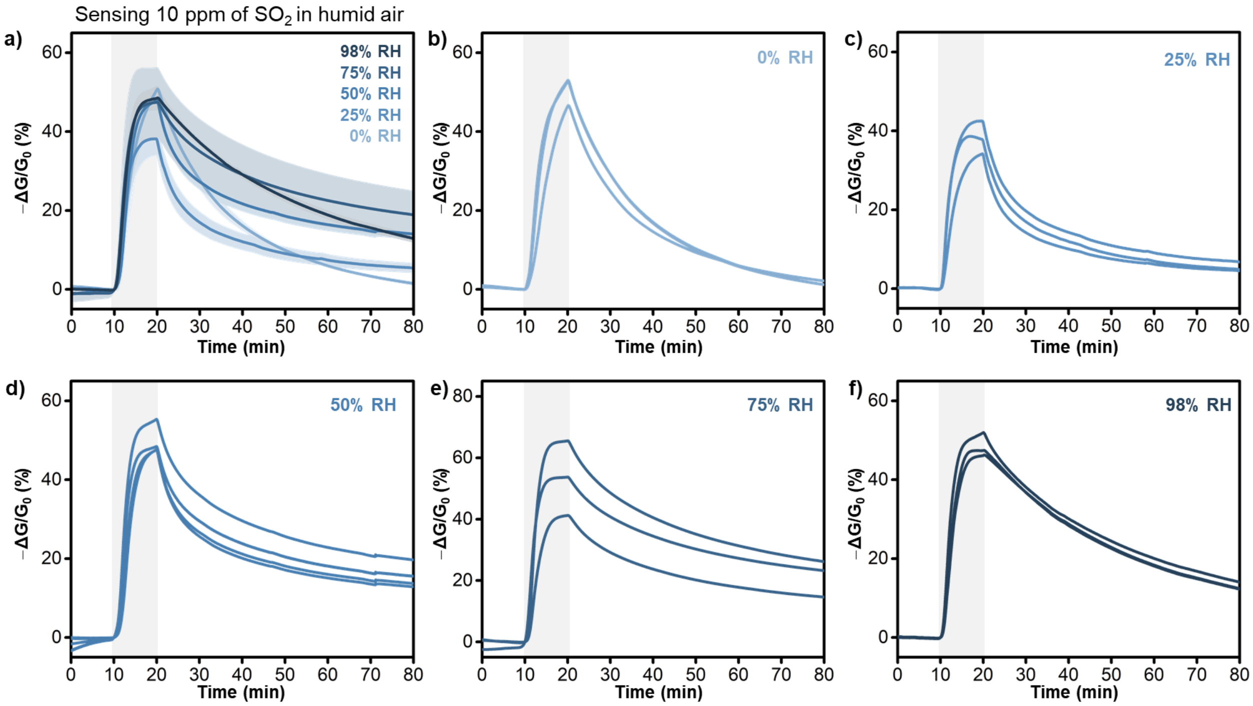

Figure S51. a) Averaged sensing responses of DC-103 towards 10 ppm of SO_2 in air at different % RH. The shaded curves represent the standard deviation of sensing responses from 3 devices and the gray shaded area represents the time of exposure of the devices to SO_2. Sensing responses of at least 3 devices of DC-103 towards b) 0% RH, c) 25% RH, d) 50% RH, e) 75% RH, and f) 98% RH in air.

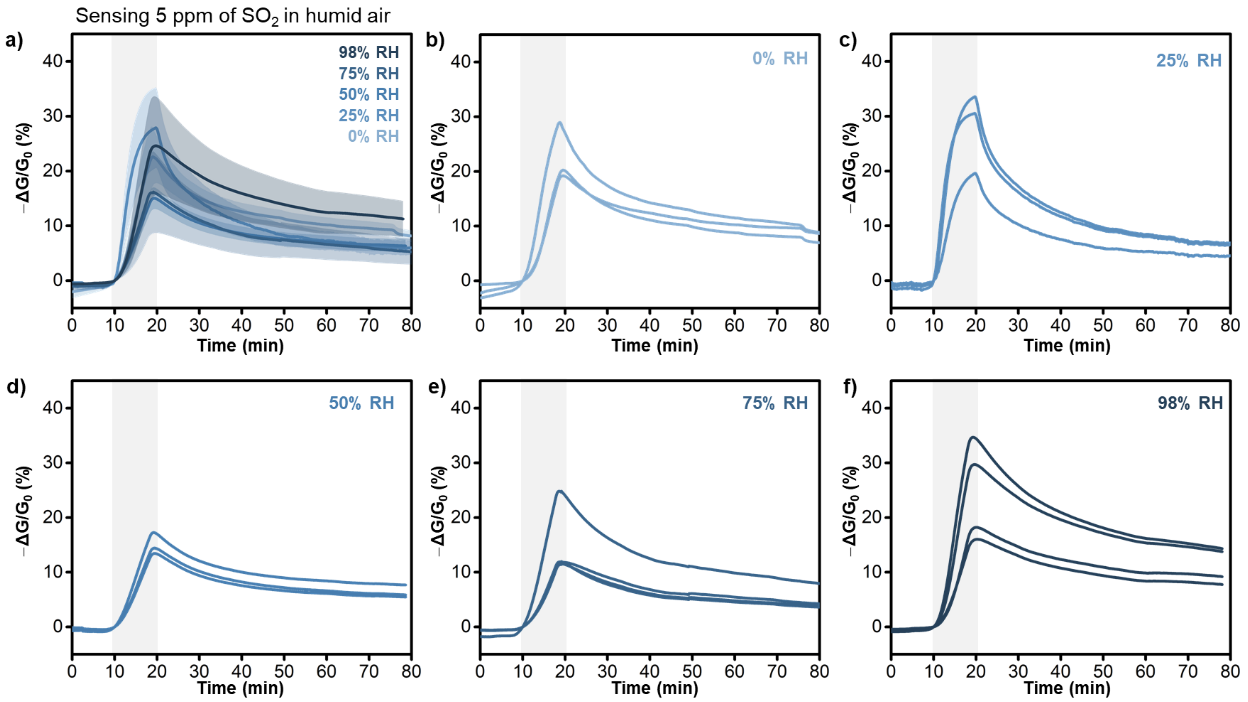

Figure S52. a) Averaged sensing responses of DC-103 towards 5 ppm of SO_2 in air at different % RH. The shaded curves represent the standard deviation of sensing responses from 3 devices and the gray shaded area represents the time of exposure of the devices to SO_2. Sensing responses of at least 3 devices of DC-103 towards b) 0% RH, c) 25% RH, d) 50% RH, e) 75% RH, and f) 98% RH in air.

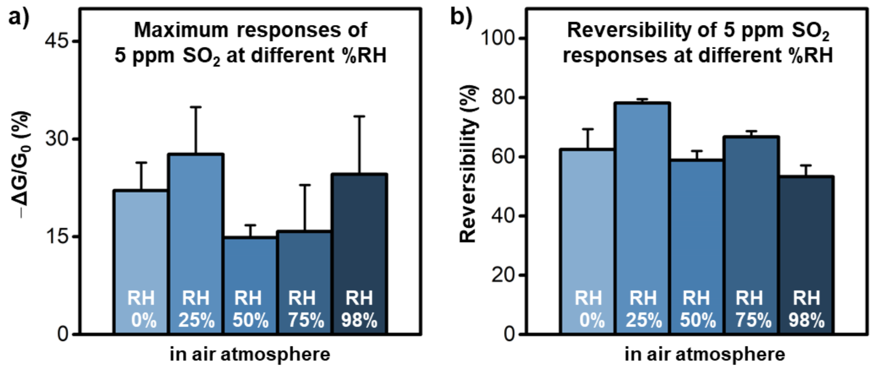

Figure S53. a) Maximum values and b) reversibility of sensing responses of DC-103 towards 5 ppm of SO_2 at different % RH in air.

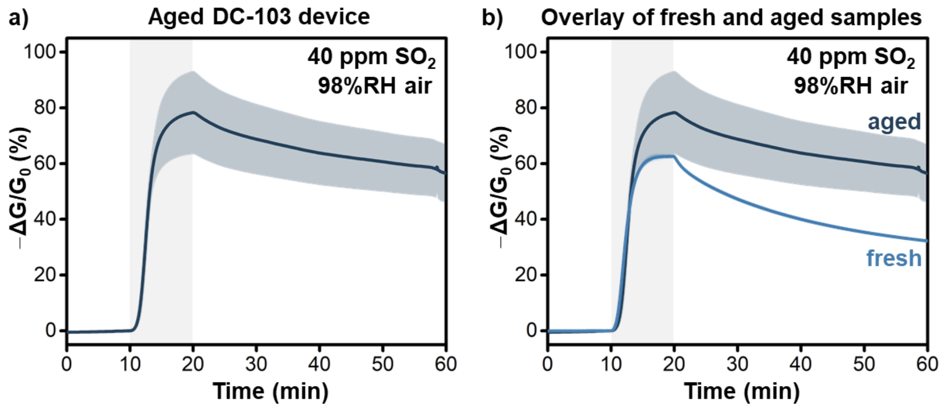

Figure S54. Sensing responses of aged DC-103 devices that were prepared and stored at ambient conditions for 4 months. Averaged sensing responses of a) aged DC-103 devices and b) overlaid responses of aged and fresh DC-103 devices towards 40 ppm of SO_2 in 98% RH air. The shaded curves represent the standard deviation of sensing responses from 3 devices and the gray shaded area represents the time of exposure of the devices to SO_2.

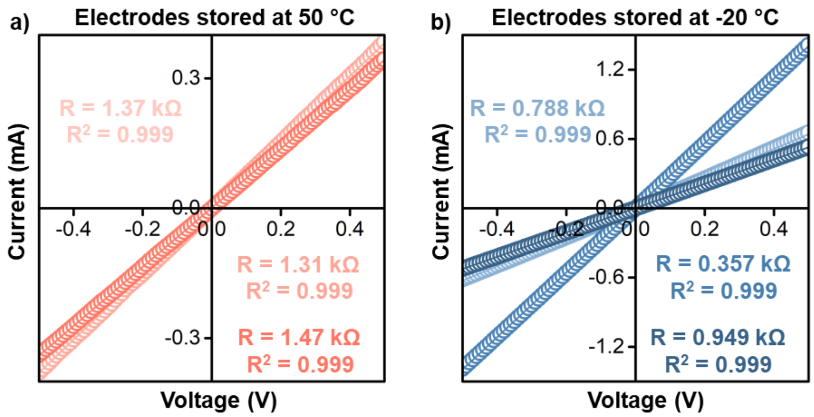

Figure S55. I-V curves of fabricated three replicate DC-103 devices after storage for 24 hours at a) 50 °C and b) -20 °C, then kept at room temperature overnight.

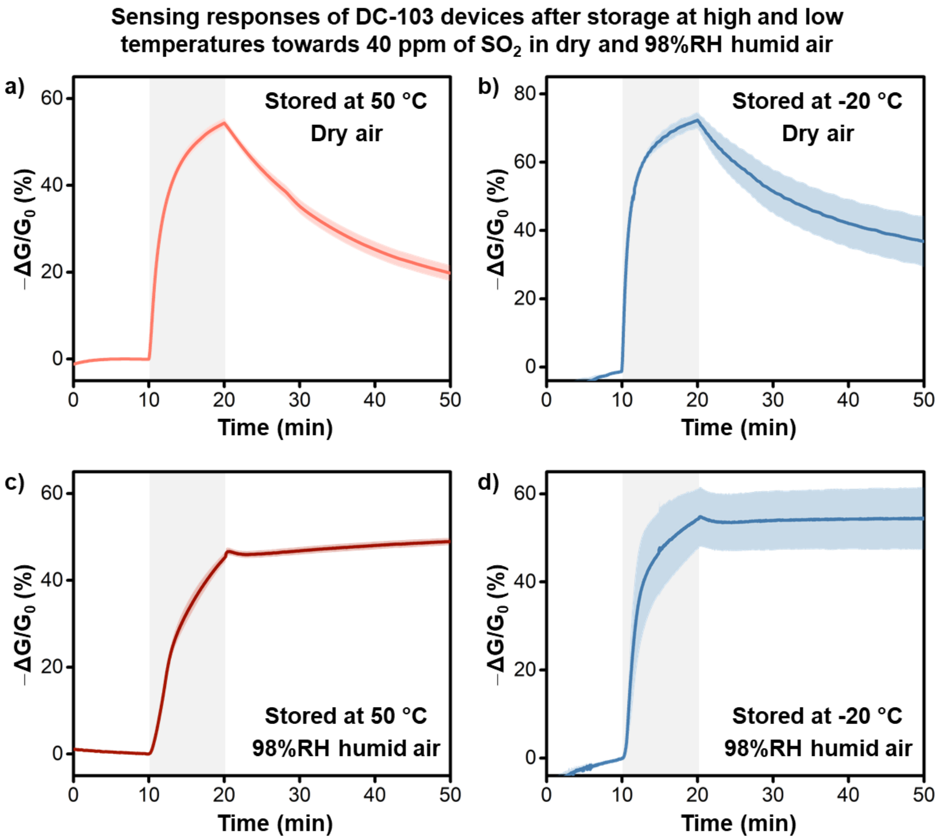

Figure S56. Sensing responses of DC-103 devices that were prepared and stored at 50 °C or -20 °C for 24 hours, then kept at room temperature overnight. Averaged sensing responses of DC-103 devices stored at a) 50 °C and b) -20 °C towards 40 ppm of SO_2 in dry air and at c) 50 C and d) -20 °C towards 40 ppm of SO_2 in 98% RH air. The shaded curves represent the standard deviation of sensing responses from 3 devices, and the gray shaded area represents the time of exposure of the devices to SO_2.

S6.3. Benchmark against previously reported materials for SO_2 sensing

Table S6. Performance of DC-103 and other humidity-tolerant SO_2 MOF/COF sensors.

MOF/COF	Mode of Sensing	response	SO_2 ppm	% RH	Back-ground gas	Reus-ability	Temp °C	LOD (ppb)	ref
DC-103 (MOF)	Chemi-resistive	$-\Delta G/G_0$ = 39.7%	10	0	N_2	0	25	220	This work
		$-\Delta G/G_0$ = 79.3%	10	98	N_2	0	25	-	
		$-\Delta G/G_0$ = 50.7%	10	0	air	-	25	57	
		$-\Delta G/G_0$ = 48.5%	10	98	air	≥ 5 cycles	25	3	
$Cu_3(HHTP)_2$@textile (MOF)	Chemi-resistive	$-\Delta G/G_0$ = 22%	80	0	N_2	0	25	150	8
		$-\Delta G/G_0$ = –34%	80	90	N_2	0	25	-	
		$-\Delta G/G_0$ = 40%	80	0	air	0	25	430	
		$-\Delta G/G_0$ = –63%	80	90	air	0	25	-	
$Cu_3(HHTP)_2$@textile-F[a] (MOF)	Chemi-resistive	$-\Delta G/G_0$ = 21%	80	0	N_2	-	25	-	8
		$-\Delta G/G_0$ = 8%	80	90	N_2	-	25	-	
		$-\Delta G/G_0$ = 54%	80	0	air	-	25	-	
		$-\Delta G/G_0$ = –22%	80	90	air	-	25	-	
KAUST-7 (MOF)	QCM[b]	$\Delta f/f = -4\times10^{-5}$	25	0	N_2	≥ 5 cycles	25	15	29
		$\Delta f/f = -6\times10^{-5}$	25	60	air	-	25	-	
KAUST-8 (MOF)	QCM	$\Delta f/f = -1\times10^{-5}$	25	0	N_2	≥ 5 cycles	25	15	29
		$\Delta f/f = 0$	25	60	air	-	25	-	
MFM-300(In) (MOF)	Capaci-tive	$\Delta C/C\% = 17x10^{-4}$	1	0	N_2	≥ 4 cycles	25	5	30
		$\Delta C/C\% = 22x10^{-4}$	1	85	N_2	-	25	-	
UiO-66-THB/PAN (MOF)	Capaci-tive	$\Delta C/C\% = 200x10^{-4}$	1	0	N_2	≥ 8 cycles	25	100	31
		$\Delta C/C\% = -34.8x10^{-4}$	1	60	N_2	-	25	-	
Eu-BDC-NH_2 (MOF)	Lumine-scent	QE[c] = 0.05	25	0	N_2	-	25	650	32
		QE = 0.19	25	100	N_2	-	25	-	
2DPAV-TBDT-IT (COF)	Chemi-resistive	R% ≈ 15	10	0	N_2	-	100	-	33
		R% = 96	10	0	air	-	100	0.088	
NKCOF-12 (COF)	Capaci-tive	$\Delta C/C_0 \approx 0.2$	1000	0	air	≥ 5 cycles	25	86	34
		$\Delta C/C_0 \approx 0.2$	1000	12	air	≥ 8 cycles	25	-	
		$\Delta C/C_0 \approx 0.2$	1000	32	air	≥ 8 cycles	25	-	

[a] sensing device coated with hydrophobic membrane
[b] quartz crystal microbalance (QCM)
[c] quantum efficiency (QE)

Discussion

It is important to note that gas sensing is not a fully standardized or unified field, and a range of systematic and experimental variables complicate direct quantitative comparisons among the materials listed in **Table S6**. Accordingly, in this section, we acknowledge and discuss the experimental factors and interpretive considerations underlying these variations in sensing conditions and performance metrics. We further describe how the sensing characteristics investigated in this work provide distinct and enhanced performance relative to previously reported materials.

- Comparison with respect to response time

Some previously reported materials for SO_2 detection do not include response time measurements, for several reasons. For example, QCM-based detection using KAUST-7 and KAUST-8 was performed upon full saturation of the MOFs with SO_2,[29] preventing real-time monitoring and precluding calculation of response times. Other studies have not explicitly reported or calculation response times, making direct comparisons inapplicable.[8, 30, 33, 34]

Additional factors that complicate comparisons include discrepancies arising from differences in analyte concentration. Response times vary depending on the analyte concentrations, and those reported at concentrations that differ from what we have studied here cannot be directly compared. For instance, while the authors report a response time of 335 s at 1 ppm and 235 s at 100 ppm for UiO-66-THB/PAN,[31] and of 6 s at 10,000 ppm for Eu-BDC-NH_2,[32] our experiments were not conducted at these concentrations. Instead, DC-103 exhibited a response time of 455 s, 117 s, and 251 s towards 40 ppm of SO_2 in dry N_2, dry air, and 98%RH humid air, respectively (**Table S4**). Thus, any direct comparison between the different reports would be deemed inaccurate.

Another important consideration is the extent of sensor saturation during exposure. Response time is typically defined as the time required to reach 90% of the maximum response (see **Section**

S6.1.7).[19] If the sensor is not fully saturated during the exposure, and if exposure times are shortened and differ across studies (such as the case for 2DPAV-TBDT-IT),[33] the reported response times will depend on these conditions and cannot be systematically compared.

- Comparison with respect to operating temperature

Room-temperature sensing enables a substantial reduction in power consumption.[16] Generally, increasing the operating temperature in chemiresistive gas sensors can enhance sensing performance.[35] This trend is illustrated in the SO_2 detection using 2DPAV-TBDT-IT, where optimal performance was achieved at an operating temperature of 100 °C, exhibiting improved response rate, response time, and sensitivity (up to 90% at 10 ppm), compared to the response observed at room temperature (40% at 10 ppm).[33] Consequently, direct performance comparisons are not straightforward when trade-offs between power consumption and sensitivity are involved.

- Comparison with respect to LODs

Building upon the discussion of LOD credibility when extrapolated from ppm-to-ppb level concentrations in **Section S6.1.5**, and while we have performed sensing experiments at ppb-levels to obtain more reliable LOD estimates, it is important to acknowledge this limitation in the LODs reported in prior studies.[8, 33] For example, sensing experiments conducted at 2–100 ppm for $Cu_3(HHTP)_2$@textile[8] and at 2–10 ppm for 2DPAV-TBDT-IT[33] yielded theoretical LODs of 150 ppb and 0.088 ppb, corresponding to approximately two and five orders of magnitude extrapolation, respectively.[8, 33] Similarly, Zhang and coworkers reported distinct linearity regimes over concentration ranges of 1–100 ppm and 200–1000 ppm; from which the former was used to estimate an LOD of 86 ppb, representing an extrapolation of approximately two orders of magnitude.[34]

It is also important to note that the low LOD of 2 ppb achieved for DC-103 in this work was obtained in a complex environment containing interferences from air. In contrast, similarly low

LODs of 5 ppb reported for MFM-300(In)[30] and 15 ppb reported for KAUST-7 and KAUST-8[29] were achieved under inert N_2 flow. Though the latter study evaluated sensor performance in simulated flue gas environments containing humidity, LOD calculations were not explicitly performed under these conditions and sensor performance in general seemed to be reduced.[29]

- Comparison with respect to robustness of performance in complex environments

Building on the discussion of interference effects from air, it is important to highlight the robust sensing performance of DC-103 reported in this work, which exhibits a consistent and reproducible response in air under saturated water vapor conditions. While some prior studies did not evaluate SO_2 detection in the presence of humidity,[33] or did not examine the influence of moisture on LOD determination and cycling stability,[29, 30, 32] other reports have documented pronounced humidity-induced effects. These include changes in response directionality, as observed for $Cu_3(HHTP)_2$@textile[8] and UiO-66-THB/PAN,[31] as well as complete suppression of sensing response under humid conditions, as reported for KAUST-8.[29] In contrast, this work demonstrates robust sensor performance in terms of signal stability, cycling reproducibility, and sensitivity at relative humidity values as high as 98%, corresponding to approximately 26,000 ppm of water vapor, which is ~650-folds higher than the SO_2 analyte concentration.

S7. Spectroscopic analyses upon SO_2 exposure

S7.1. Diffuse Reflectance Infrared Fourier Transform Spectroscopy (DRIFTS)

For data collection, a homogeneous composite of potassium bromide (KBr) with DC-103 was placed in a sample cup in an air-tight sealed steel chamber with a gas inlet and gas outlet port and with KBr windows to allow IR beam light path. The composite is activated by heating to 100 °C under nitrogen or air flow for around 1 hour while in the sample cup, followed by its respective cooling. The MOF/KBr composite is then subjected to a single beam (unsubtracted), which was used as the initial spectrum, while accounting for a background spectrum of dry KBr. Then,

difference spectrum (Kubelka-Munk) are collected by subtracting spectra collected during exposure from the initial spectrum. Each of these spectra were collected with 32 scans from 400 to 4000 cm^{-1}. Due to low signal of gases at the concentrations at which sensing experiments were performed, we opted to use two higher concentrations of gas (400 ppm and 1% of SO_2 in nitrogen or air) for the DRIFTS experiments. At 400 ppm, we recorded spectra every 5 minutes of exposure to SO_2, followed by recovery spectra every 10 minutes while purging with nitrogen gas. At 1%, we recorded spectra at 0, 1, 2, 5, and 10 min of exposure to SO_2, followed by recovery spectra at 2, 6, 8, and 10 min as well while purging with nitrogen gas.

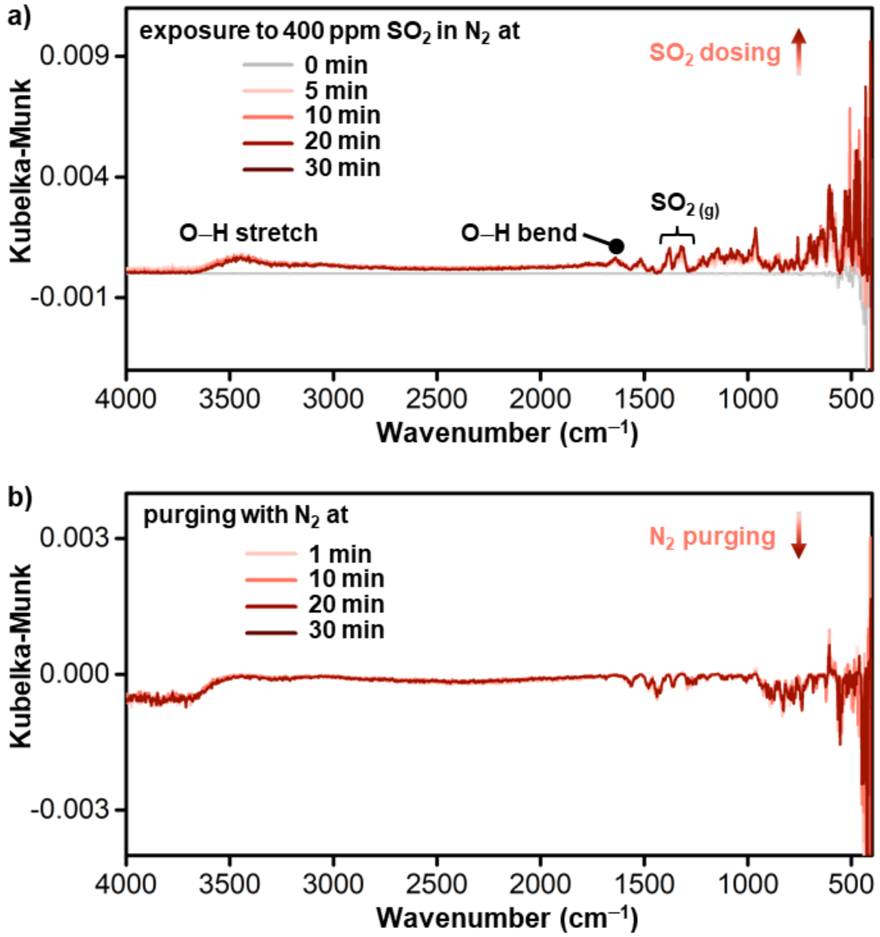

Figure S57. DRIFTS difference spectra of DC-103 a) upon exposure to 400 ppm of SO_2 in nitrogen and b) upon recovery with nitrogen.

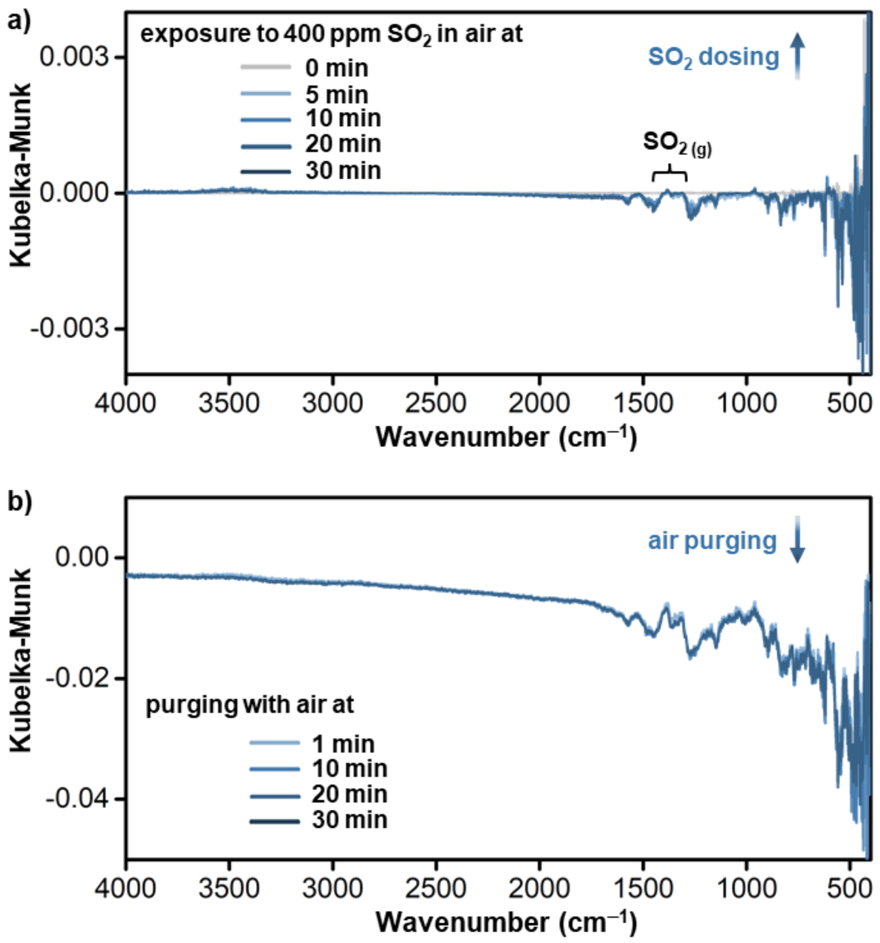

Figure S58. DRIFTS difference spectra of DC-103 a) upon exposure to 400 ppm of SO_2 in air and b) upon recovery with air.

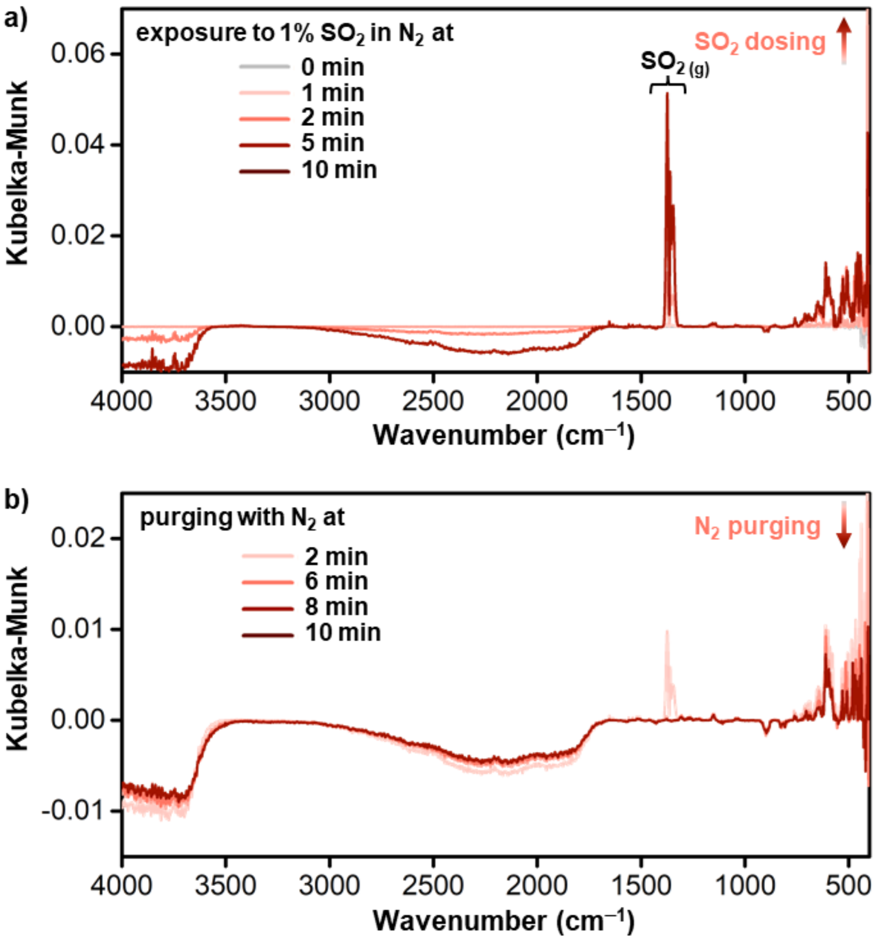

Figure S59. DRIFTS difference spectra of DC-103 a) upon exposure to 1% of SO_2 in nitrogen and b) upon recovery with nitrogen.

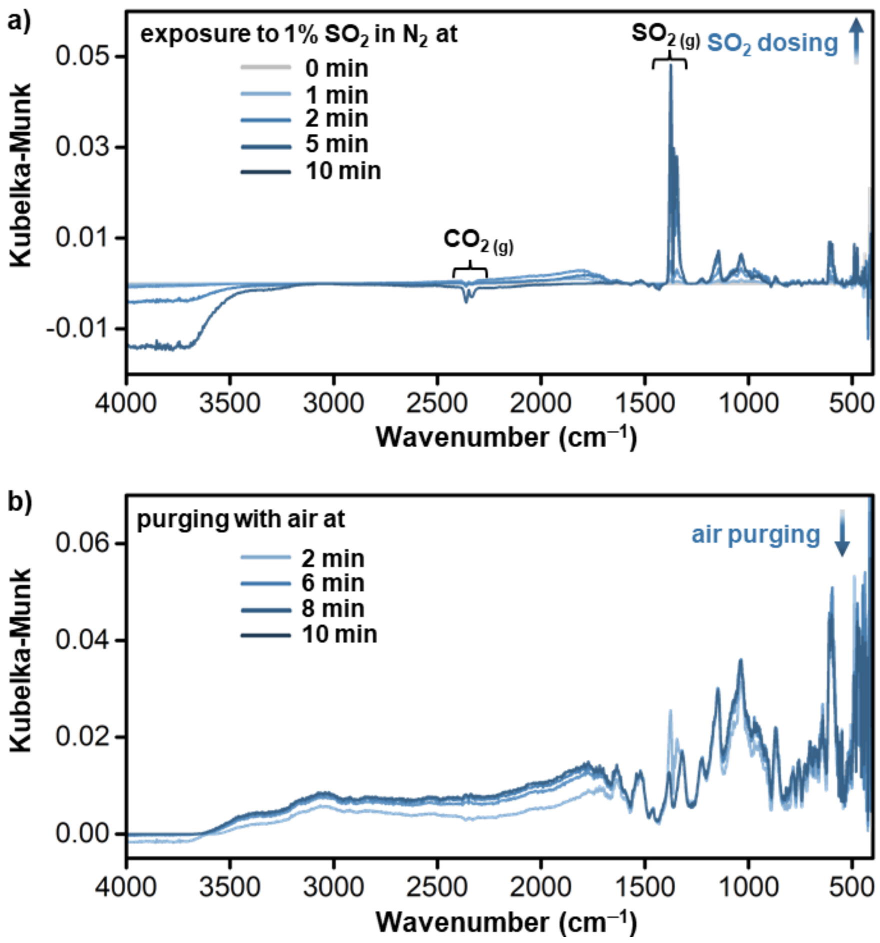

Figure S60. DRIFTS difference spectra of DC-103 a) upon exposure to 1% of SO_2 in nitrogen and b) upon recovery with air.

S7.2. PXRD and XPS characterization after exposure to gases

For PXRD and XPS collected after exposure, the MOF powder samples were placed in a sealed container with an inlet of the gas analyte (at 40 ppm concentration in nitrogen, air, or 98% RH humid air) and an outlet for 3 hours before analysis.

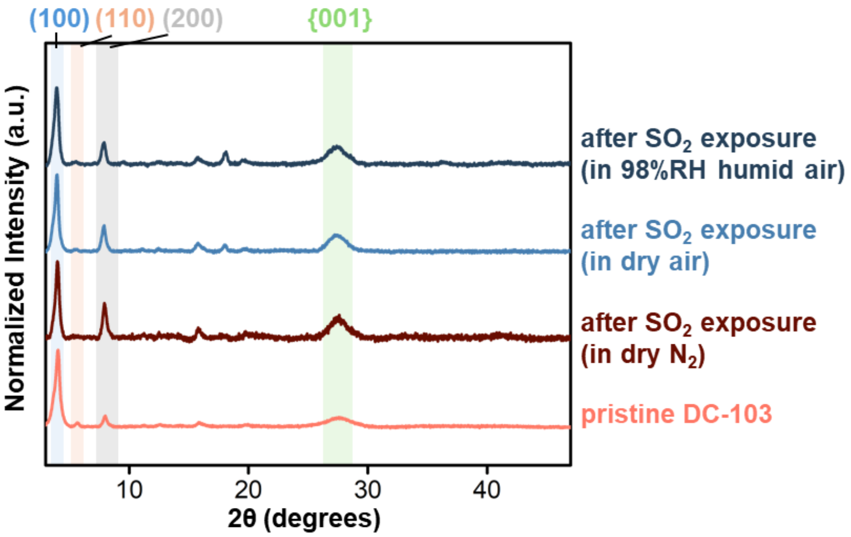

Figure S61. PXRD spectra of DC-103 before and after exposure to 40 ppm of SO_2 in dry N_2, dry air, and 98% RH humid air for 3 hours.

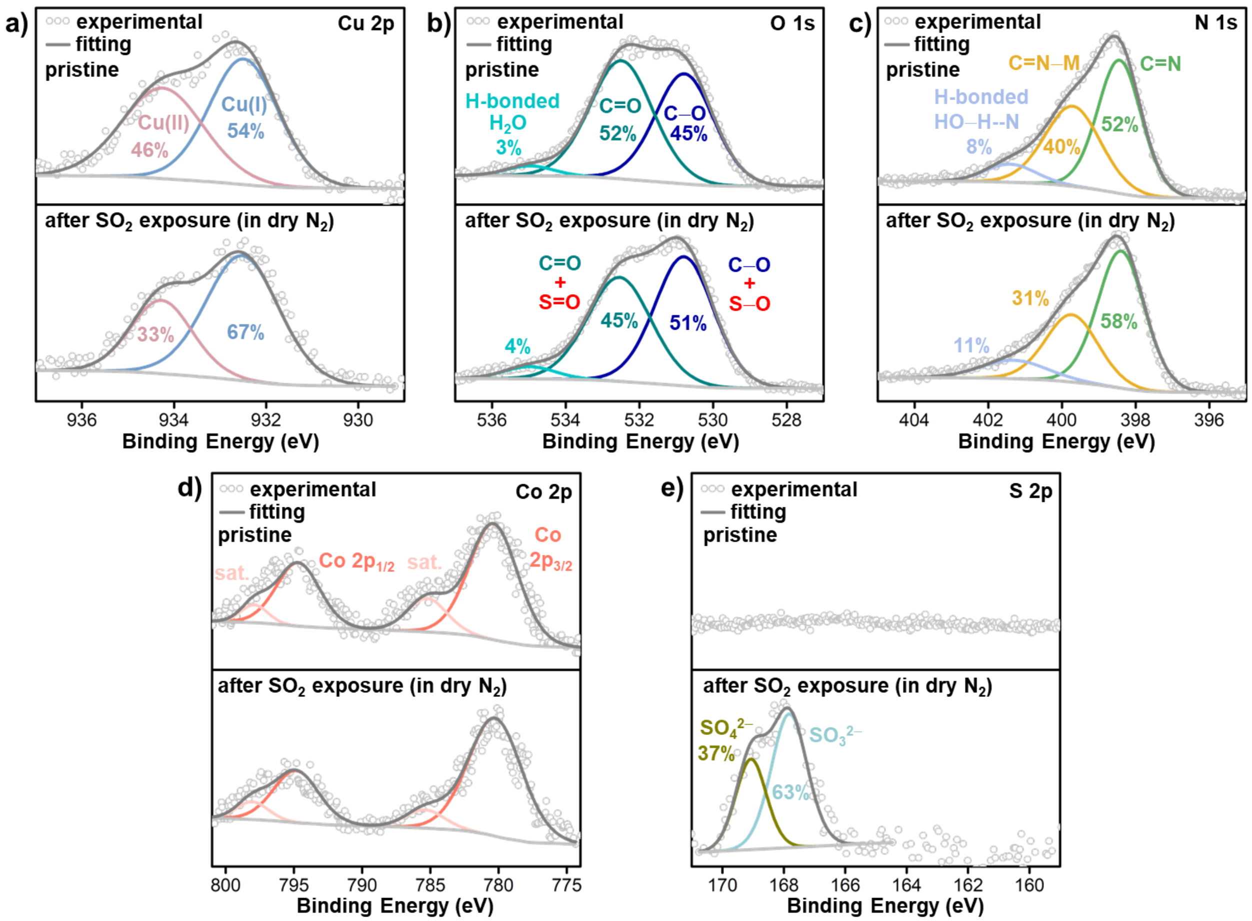

Figure S62. High-resolution XPS spectra of DC-103 after exposure to 40 ppm of SO_2 in dry N_2 for 3 hours with the binding energies of a) Cu 2p, b) O 1s, c) N 1s, d) Co 2p, and e) S 2p.

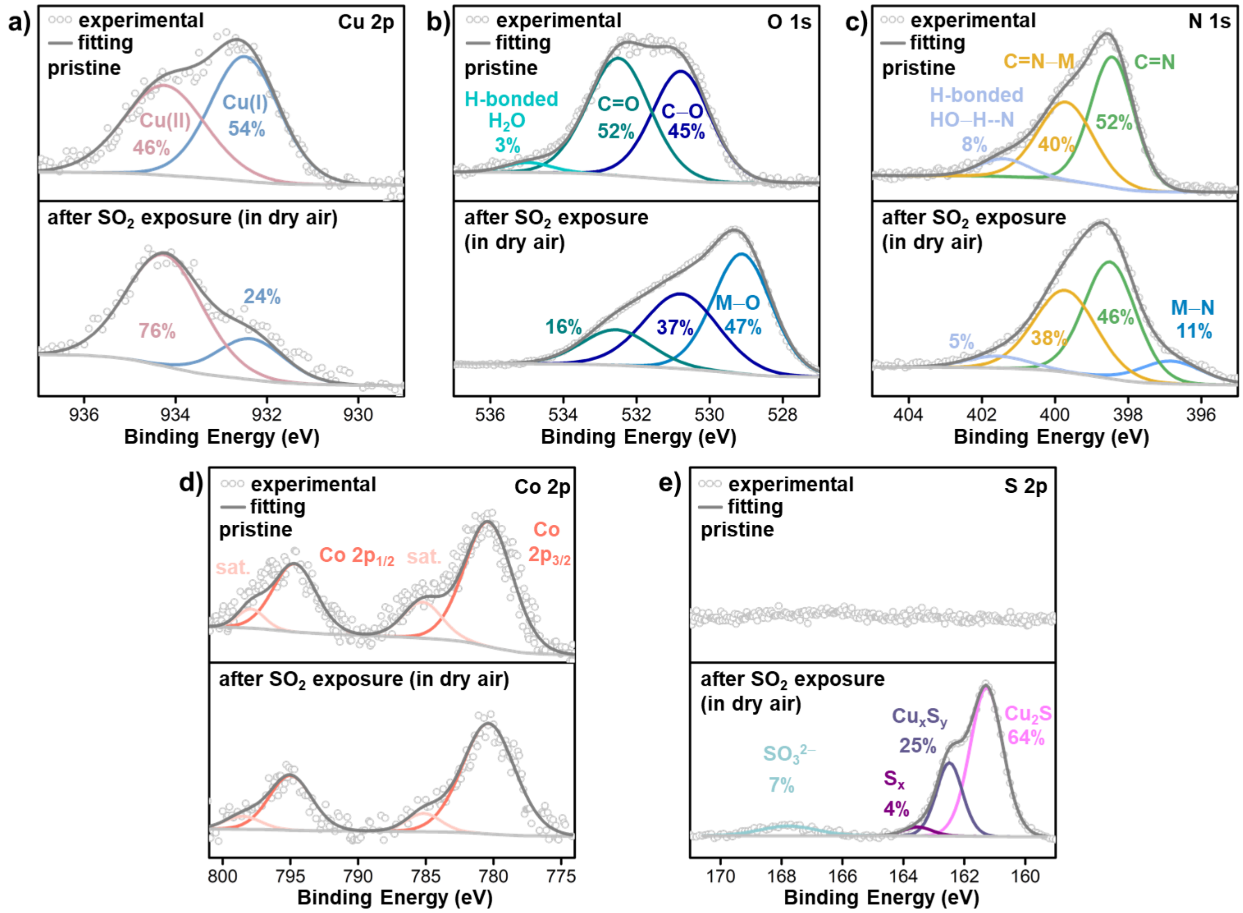

Figure S63. High-resolution XPS spectra of DC-103 after exposure to 40 ppm of SO_2 in dry air for 3 hours with the binding energies of a) Cu 2p, b) O 1s, c) N 1s, d) Co 2p, and e) S 2p.

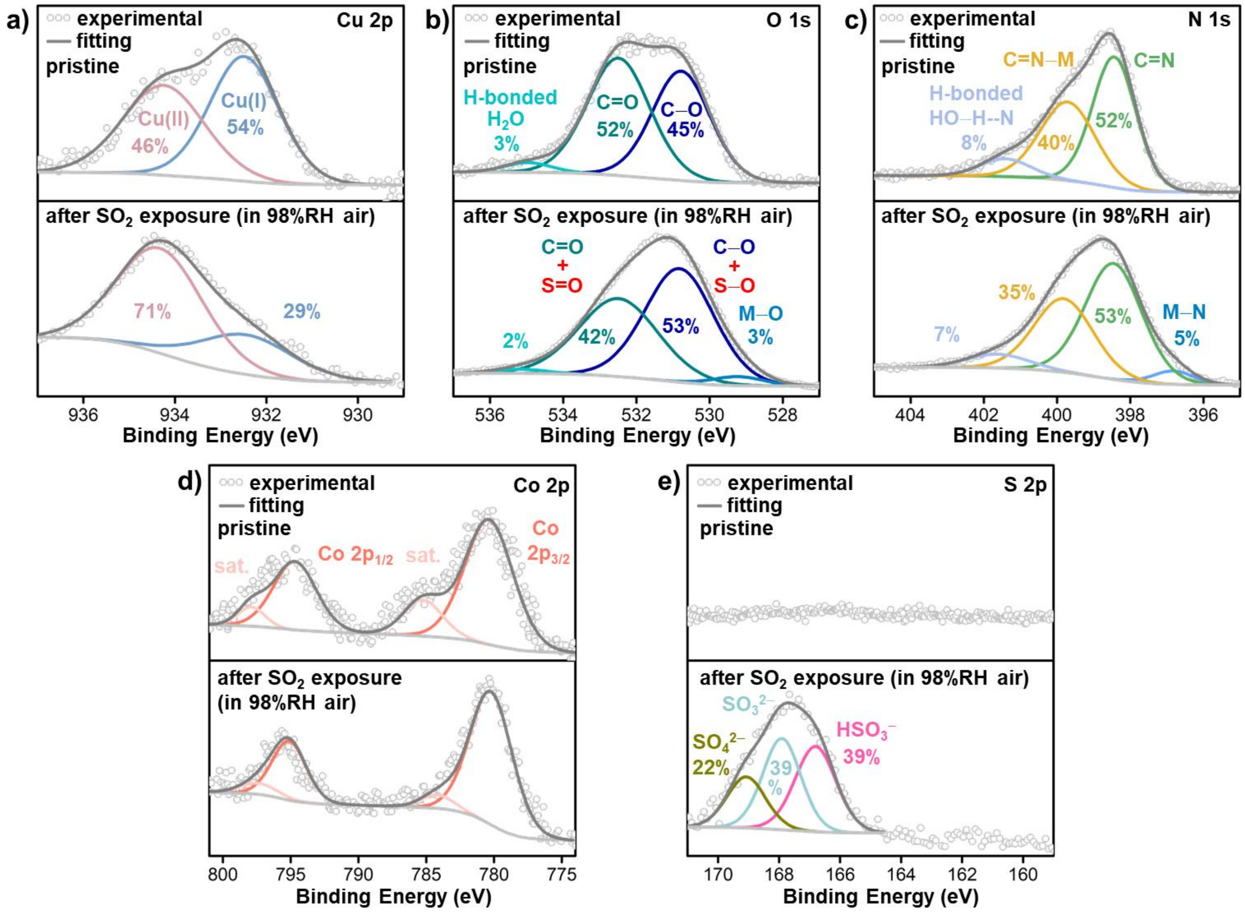

Figure S64. High-resolution XPS spectra of DC-103 after exposure to 40 ppm of SO_2 in 98% RH air for 3 hours with the binding energies of a) Cu 2p, b) O 1s, c) N 1s, d) Co 2p, and e) S 2p.

S7.3. Relating spectroscopic analysis to sensing at 40 and 400 ppm

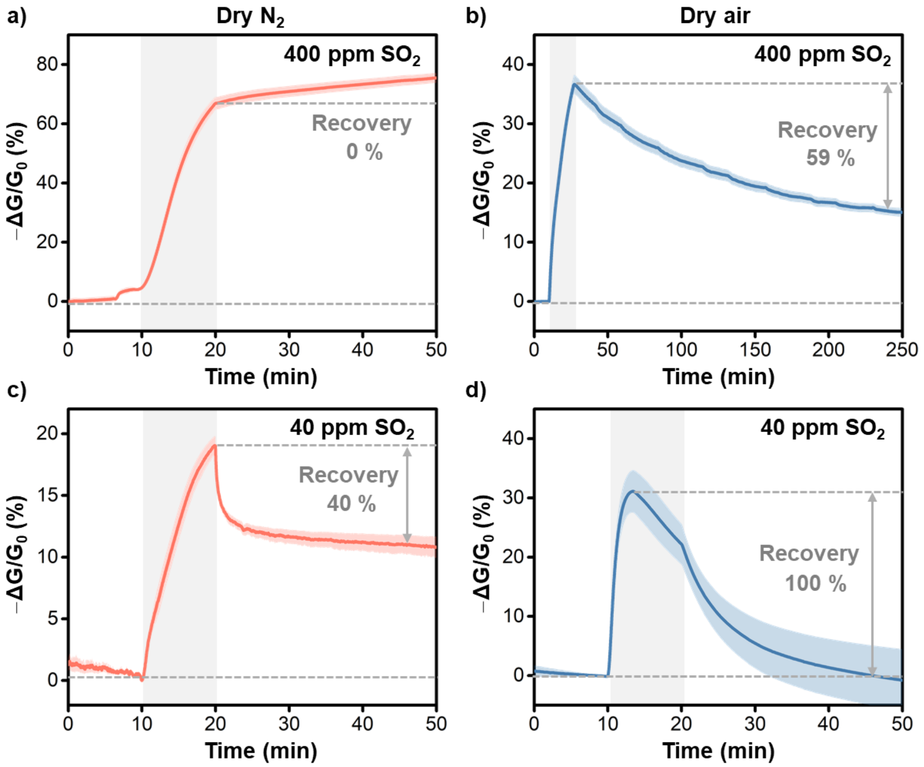

Figure S65. Averaged sensing responses of DC-103 devices towards a) 400 ppm SO_2 in dry N_2, b) 400 ppm SO_2 in dry air, c) 40 ppm SO_2 in dry N_2, and d) 40 ppm SO_2 in dry air. The shaded curves represent the standard deviation of sensing responses from 3 devices and the gray shaded area represents the time of exposure of the devices to SO_2.

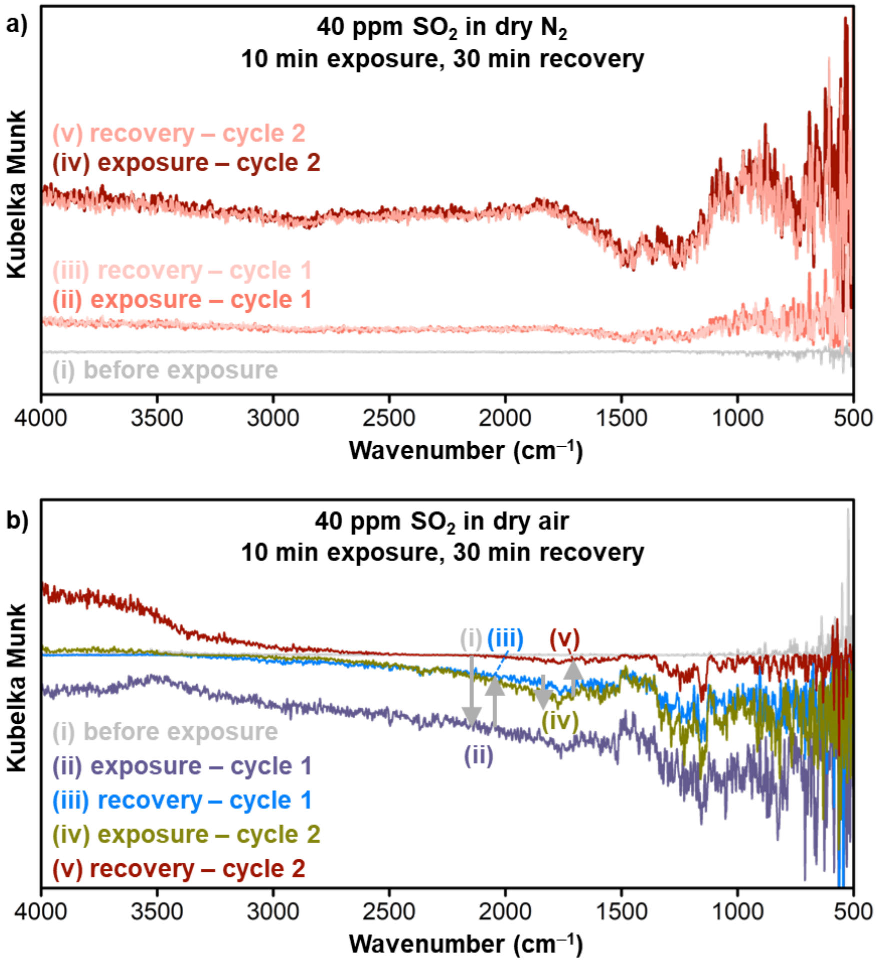

Figure S66. DRIFTS difference spectra of DC-103 upon exposure to two consecutive cycles of 40 ppm of SO_2 in a) dry N_2 and b) dry air for 10 minutes, followed by a 30 minute background gas purge period after each exposure cycle.

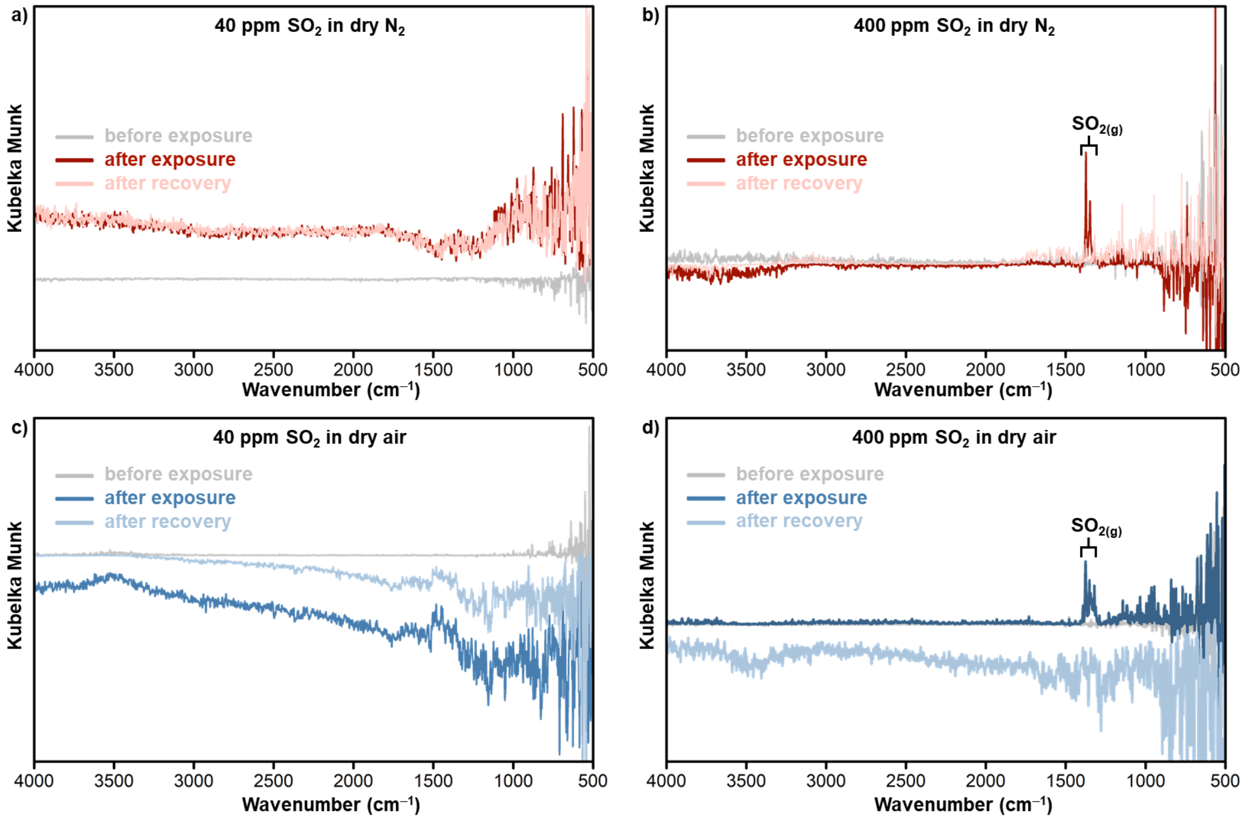

Figure S67. DRIFTS difference spectra of DC-103 upon exposure to a) 40 ppm of SO_2 in dry N_2, b) 400 ppm of SO_2 in dry N_2, c) 40 ppm of SO_2 in dry air, and d) 400 ppm of SO_2 in dry air. The "after exposure" spectrum is collected after 10 minutes of exposure to SO_2, while the "after recovery" spectrum is collected following a 30 minute purge in background gas.

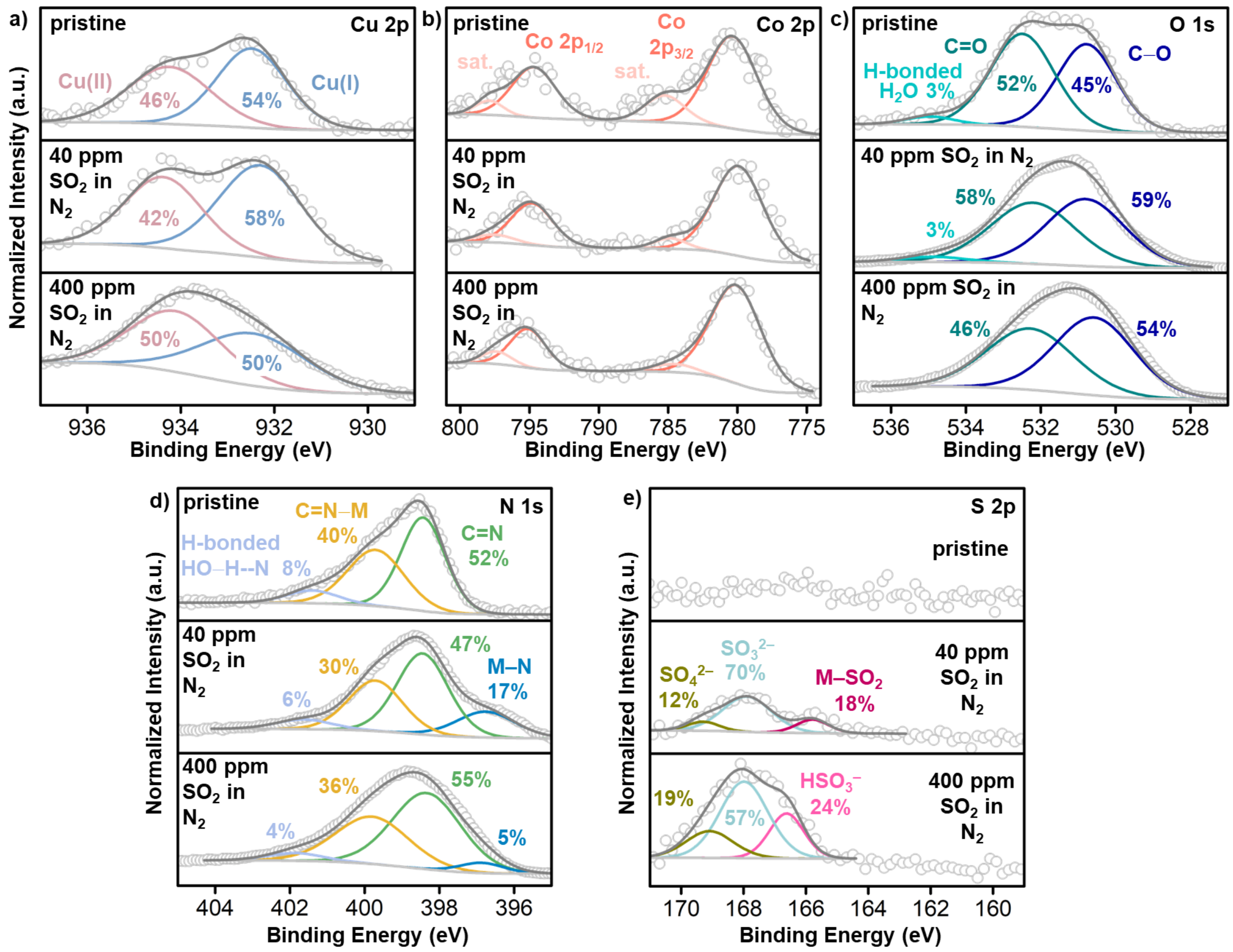

Figure S68. High-resolution XPS spectra of DC-103 after exposure to 40 ppm of SO_2 in dry N_2 for 10 minutes with the binding energies of a) Cu 2p, b) Co 2p, c) O 1s, d) N 1s, and e) S 2p.

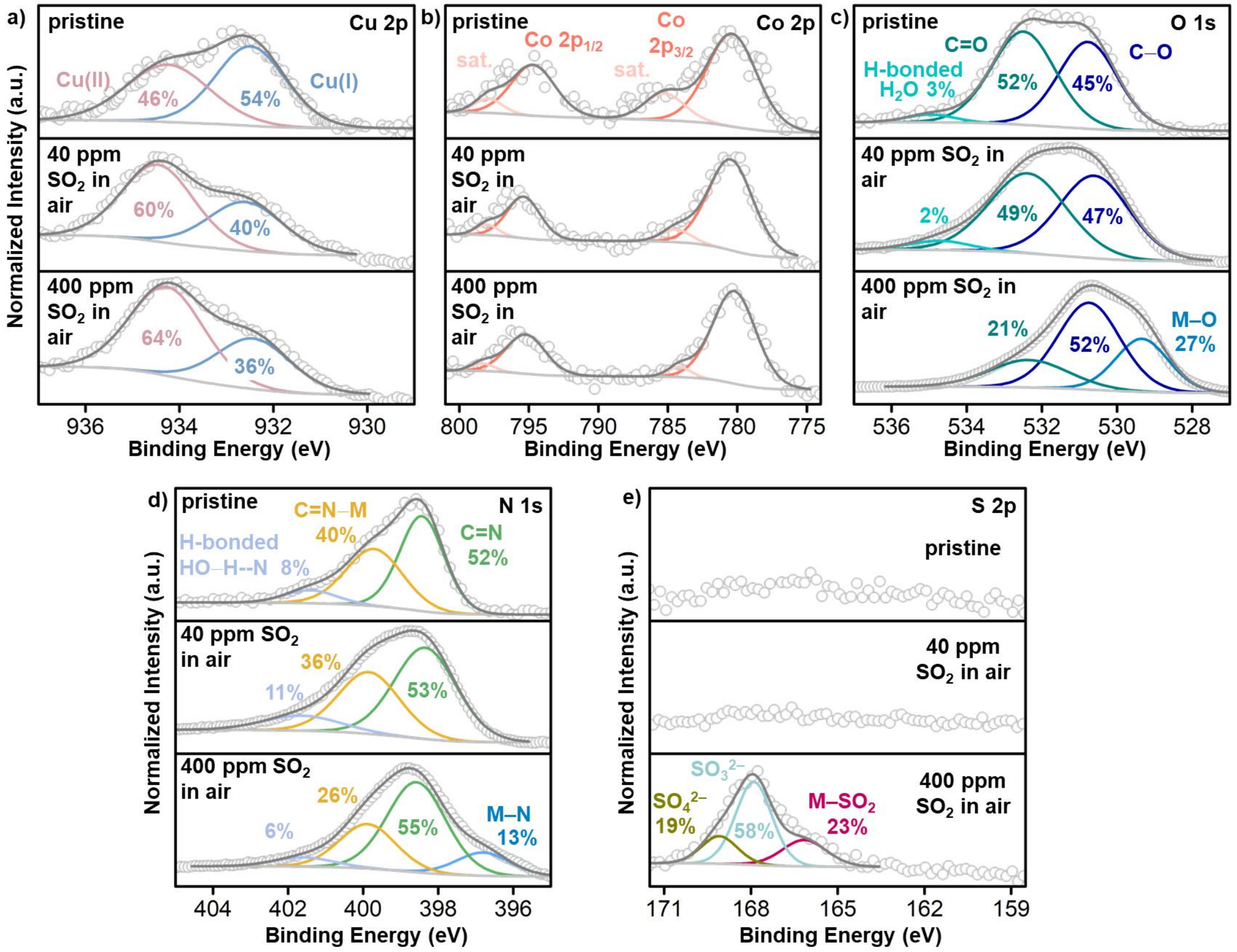

Figure S69. High-resolution XPS spectra of DC-103 after exposure to 40 ppm of SO_2 in dry air for 10 minutes with the binding energies of a) Cu 2p, b) Co 2p, c) O 1s, d) N 1s, and e) S 2p.

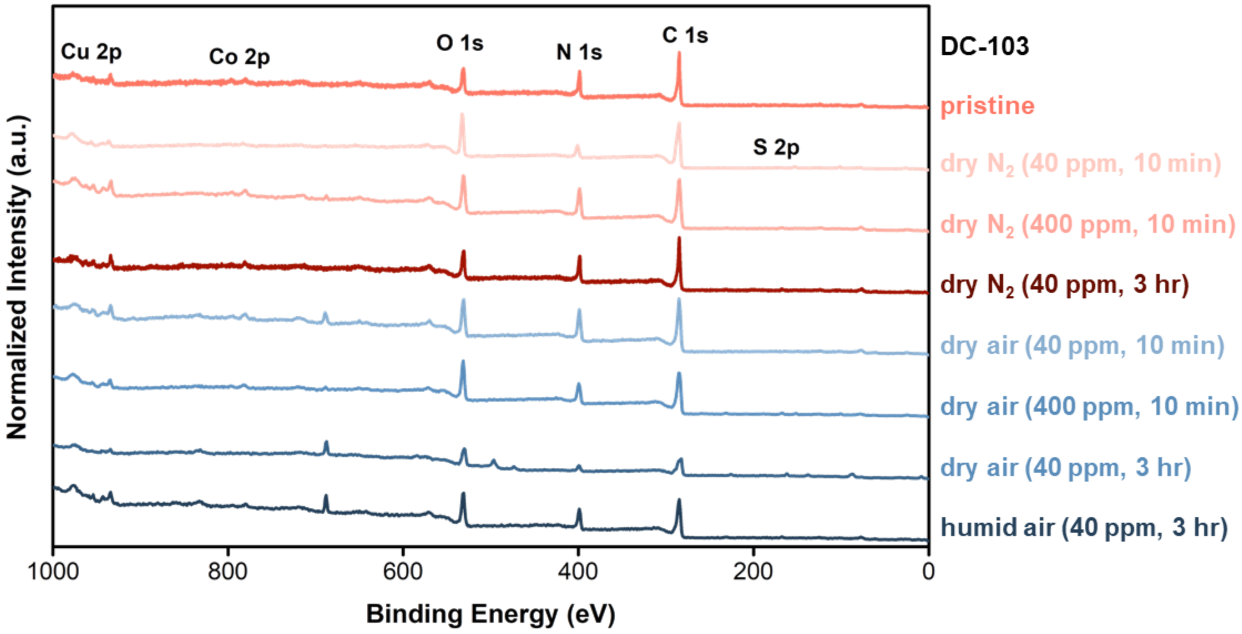

Figure S70. XPS survey spectra of pristine DC-103, and after exposure to 40 ppm of SO_2 in dry N_2, dry air, and 98% RH humid air for different time periods.

Note the peaks observed at ~687 eV in some of the spectra are attributed F 1s, likely arising from the fluorinated species in the carbon tape used as a background for sample holding during XPS data acquisition.[36]

Table S7. Table summarizing the percentage of sulfur adsorbed on DC-103 after exposure to SO_2 under various conditions. Values are determined from XPS survey spectra of **Figure S66** by Peak ID and Quantification in ESCApe.

Atmosphere of exposure	SO_2 concentration	Exposure time	Atomic concentration
Pristine DC-103	-	-	0.1 ± 0.1 %
Dry N_2	40 ppm	10 min	0.2 ± 0.1 %
	400 ppm	10 min	0.3 ± 0.1 %
	40 ppm	3 hours	1.0 ± 0.2 %
Dry air	40 ppm	10 min	0.1 ± 0.1 %
	400 ppm	10 min	0.4 ± 0.1 %
	40 ppm	3 hours	1.9 ± 0.3 %
Humid air (98% RH)	40 ppm	3 hours	0.5 ± 0.2 %

S8. Computational characterization of intermolecular interactions

S8.1. Reactive Molecular Dynamics Simulations

After optimizing the DC-103 structural configuration in the gas phase at the classical level, we included all the guest species, namely water, SO_2, O_2, or N_2 molecules (in various combinations), and simulated the system evolution in a z-size extended box to mimic the system with an interface. We identified preferred interaction sites for the species within the MOF channels (16 channels), close to the metal centers, at the interface, during reactions, and during molecular migration.

We examined the system's behavior when loaded with SO_2 under high- and low-humidity conditions, and in the presence of O_2 and N_2. In the high-humidity model, we inserted water layers within 3.5 Å of the channel's walls ≈1300 waters. In contrast, in the low-humidity model, the extension of these layers was reduced by 1.5 Å, corresponding to a 76% decrease in the water content. SO_2 molecules were added into the center of the channels (364 molecules), and O_2 or N_2 were inserted into the left space, both at the same concentration (124 molecules). The RMD simulations were performed at ambient temperature and pressure for approximately 2 ns.

The dynamics of the supercell model was simulated by the ReaxFF MD code available in the LAMMPS program,[37] and all the calculations were based on published, well-tested parameters available upon request.

The equilibration procedure was described in earlier studies. Briefly, during the RMD simulations, reactivity was always on, no constraints were applied to the systems, and all species could interact with one another and modify their conformations in response to the environment. The models were energy-minimized, gradually heated to 298 K, and equilibrated at that temperature (the equilibration time was chosen based on system size and composition; the equilibrium was checked by examining the trend in the potential energy). After equilibration, production RMDs were performed in the NVT ensemble for about 2 ns. The time step was set to 0.25 fs. The

Anderson thermostat controlled the temperature with a relaxation constant of 0.1 ps. The system configurations were collected every 0.5 ps. The analysis of the sampled data was focused on the last 500 ps of the trajectories. We examined atom-atom radial distribution functions (RDFs), the percent of species within the channels, and the shape and volume of the channels (Caver software).[38] Visual inspection of the trajectories was fundamental to understanding the adsorption scenarios of the species, reaction tendencies, and the evolution of the MOF structure.

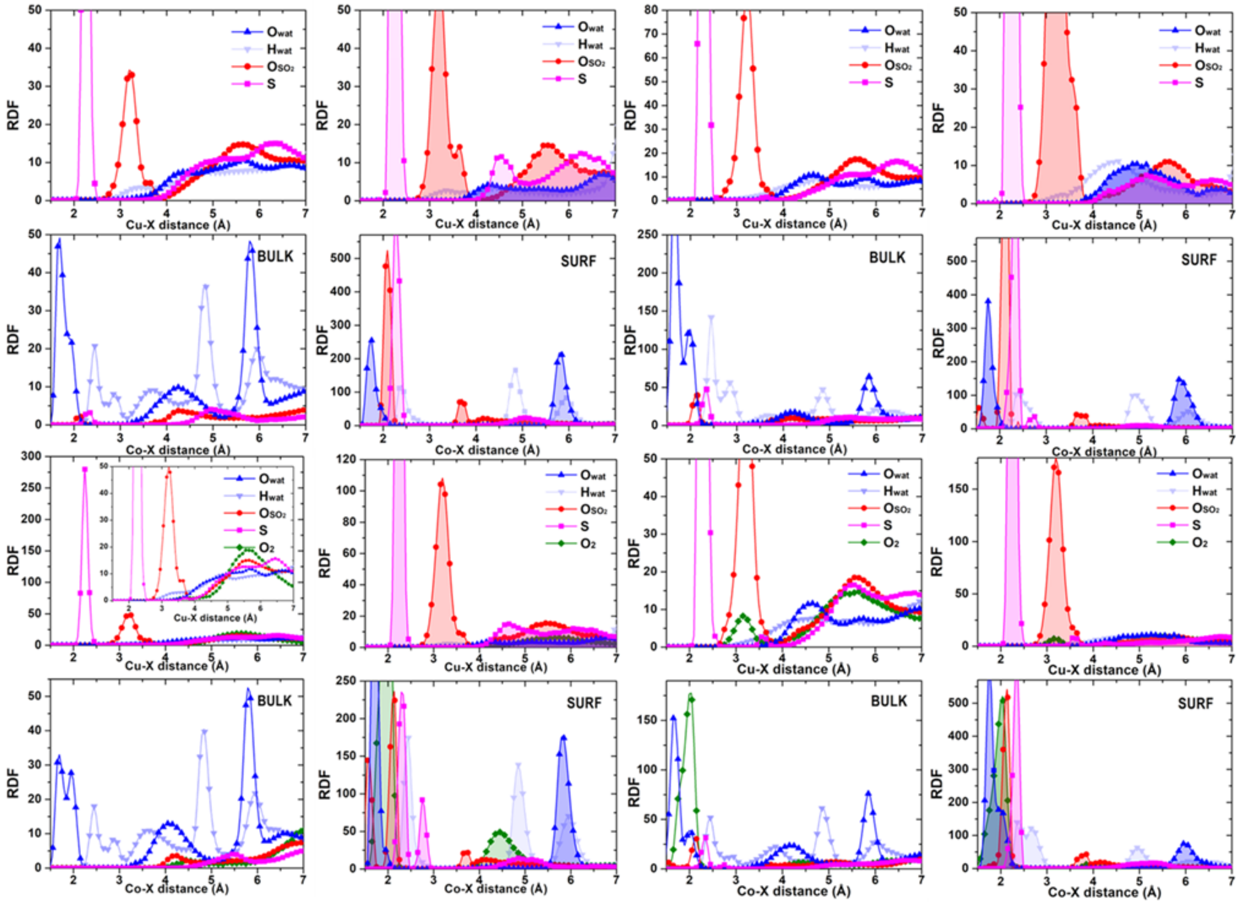

Figure S71. Top: atom-atom RDF plots of the MOF bulk and surface (filled areas under the curves) at high (four plots on the left) and low (four plots on the right) humidity without O_2**.** Bottom: atom-atom RDF plots of the MOF bulk and surface (filled areas under the curves) at high (four plots on the left) and low (four plots on the right) humidity with O_2 (green lines and squares).

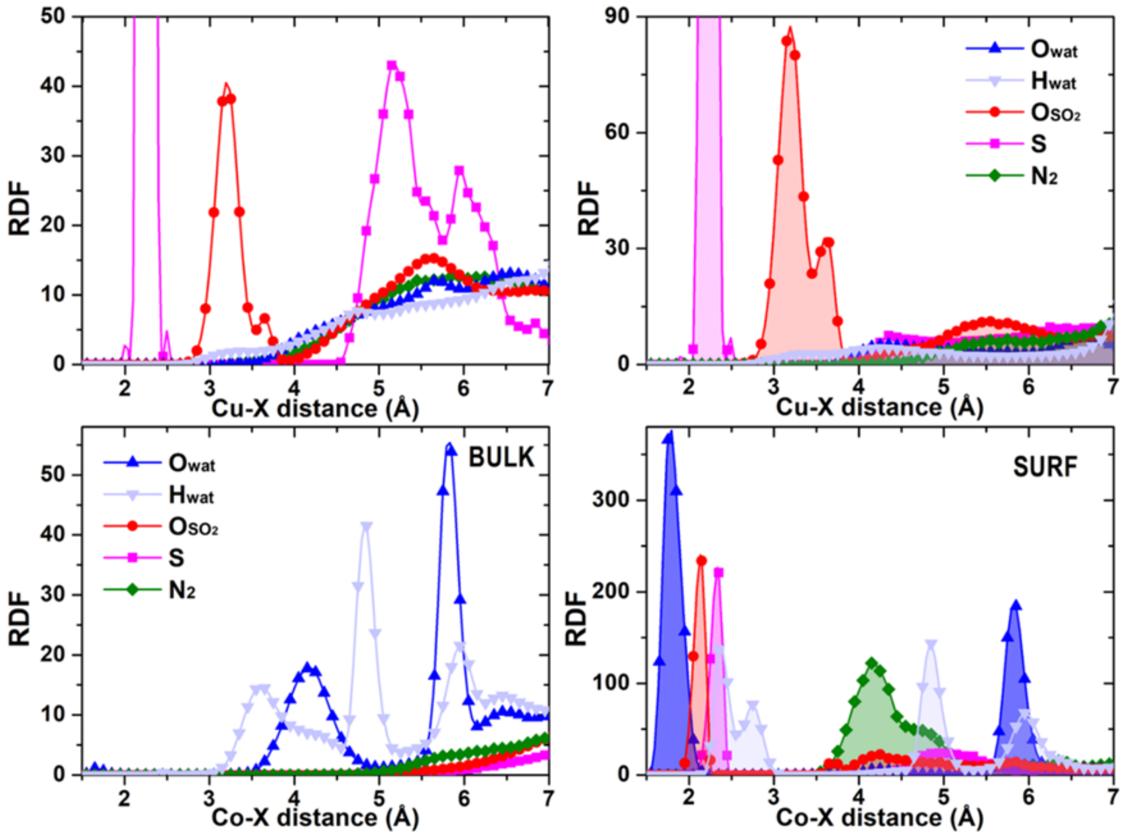

Figure S72. Atom-atom RDF plots of the MOF bulk and surface (filled areas under the curves) at high humidity with N_2 (green lines and squares).

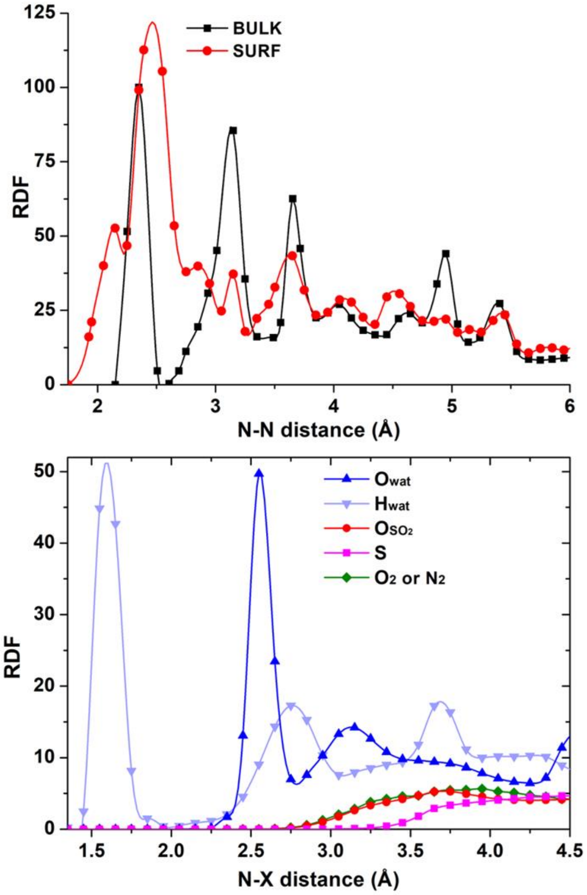

Figure S73. Atom-atom RDF of TPz N with itself (top), water (oxygen and hydrogen atoms), SO_2 (oxygen and sulfur atoms), and O_2 or N_2 (bottom). The comparison between Bulk and Surface shows a more marked tendency to rototranslation in the second case (red line).

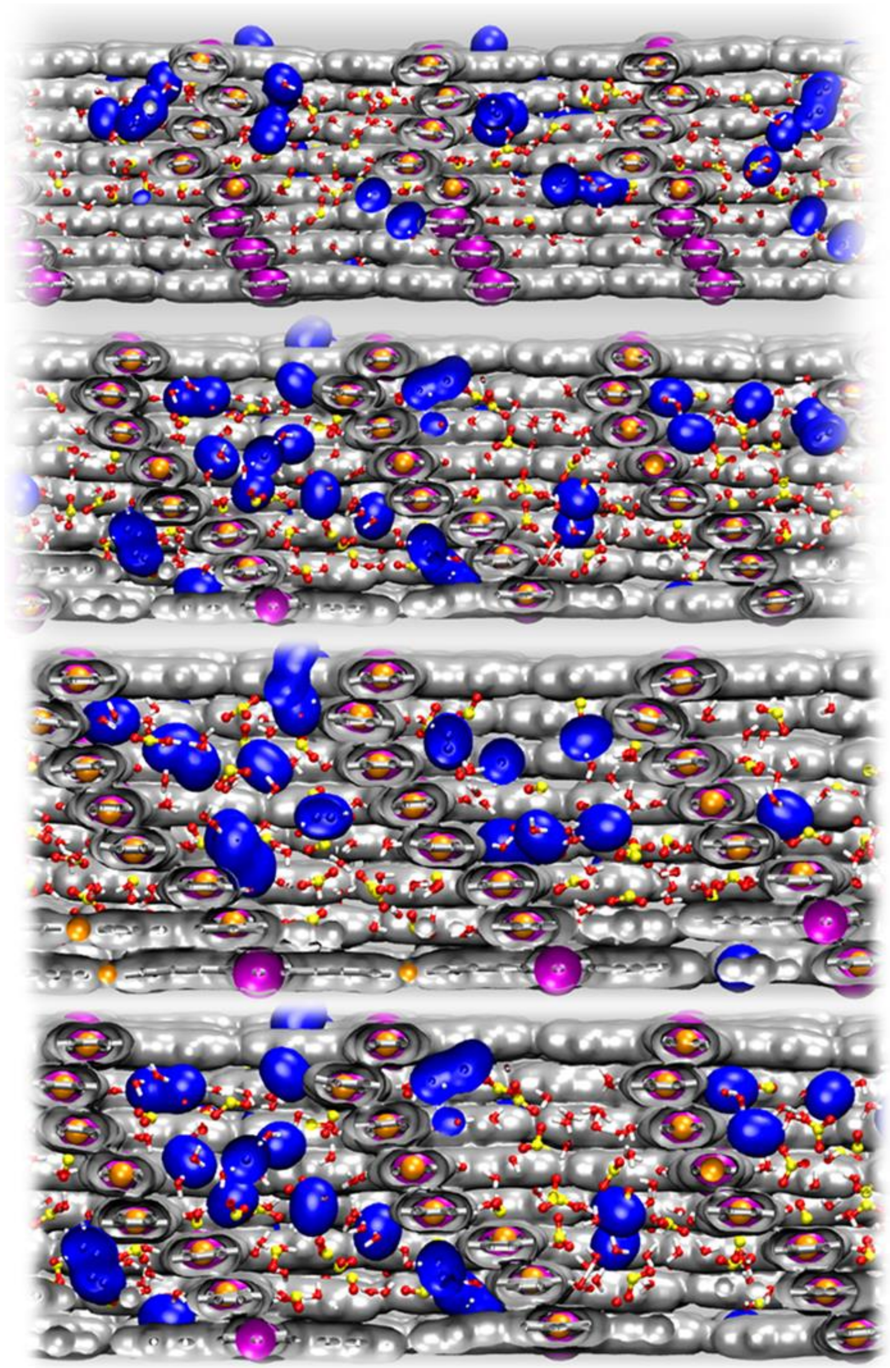

Figure S74. Blue areas represent the remaining N_2 molecules inside the tunnels. In these sections, all the species, namely water and SO_2, are present and interconnected. N_2 molecules are mainly located near the open metal (Cu) sites, with a few in the center of the channels. Their presence had a confining effect on the guests, preventing them from leaving the MOF. Even though the N_2 concentration used in the simulations was low, the tendency to separate the guests and limit their motion is apparent.

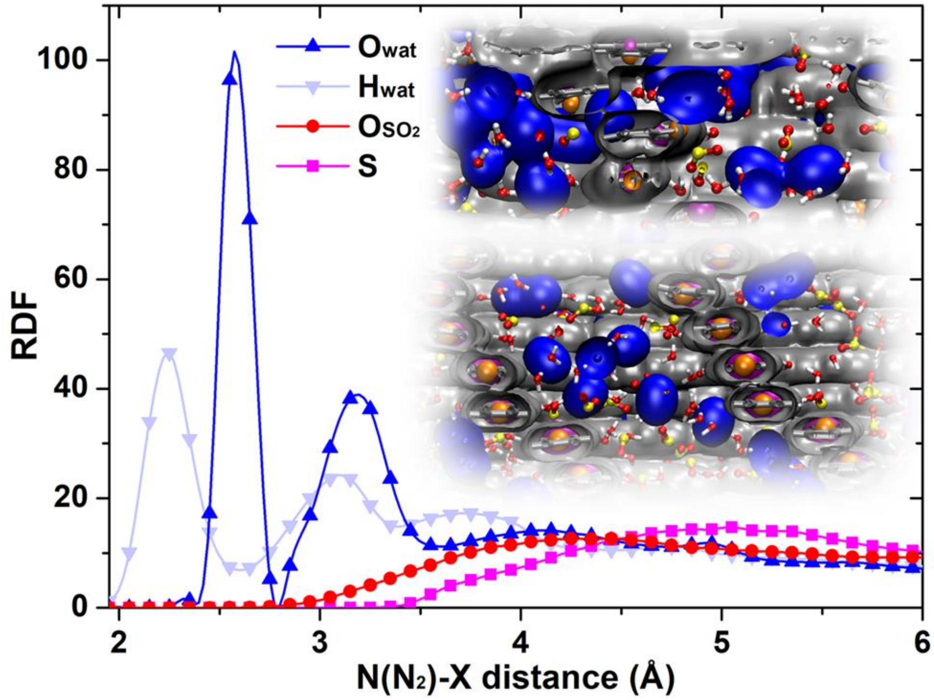

Figure S75. Atom-atom RDF of N with water (oxygen and hydrogen atoms) and SO_2 (oxygen and sulfur atoms) atoms. In the inset, two pictures of representative sections of two channels (small portions) filled with nitrogen (surface accessible blue areas), water (red (O) and white(H) ball and stick model), and SO_2 (yellow (S) and red (O) ball and stick model) molecules.

To have an idea of DRIFTS signals from the computational models, we collected additional samples to simulate the IR spectra of the MOFs with guests (H_2O and SO_2, in N_2 and air), by extending the MD trajectories by 100 ps for collecting the instantaneous net dipole moments of the system (1 million data points in all). The Python script calc-ir-spectra.py[39] was then used to calculate the dipole moment's autocorrelation functions, and to perform a Fourier transform for obtaining the IR lineshape. The spectra were then calculated by multiplying the lineshape by an electromagnetic-field-dependent prefactor, as suggested in the literature. The time step was set to 0.1 fs, and the dipole was collected at every step.

In **Figure S76**, the spectrum of the MOF loaded with N_2 (red) shows standard SO_2 stretching modes, whereas the significantly broadened new features in the 1050–1150 cm^{-1} range visible in

the air spectrum (blue) confirm the reaction of SO_2 at the Cu sites and the subsequent formation of sulfites (SO_3^{2-}) or sulfates (SO_4^{2-}). Unlike Cu, which acts as an active catalytic center for this conversion, the Co centers are, instead, less accessible to the SO_2 guests in the bulk of the framework and thus act only as structural anchors.

The characteristic SO_2 bands at about 530 cm^{-1} (bending), 1150 cm^{-1} (symmetric stretching), and 1360 cm^{-1} (asymmetric stretching) are visible in both IR spectra. Still, as shown in the bottom zoom of **Figure S76**, the SO_2 bending mode is perturbed and broadened in air (in the presence of O_2). This suggests a change in the coordination geometry, transitioning from a simple physisorbed state or O-coordinated state to a direct S–Cu bond. The direct S–Cu usually increases the frequency of the SO_2 stretching modes relative to the free gas, as evidenced in the bottom plot by the broadening and emergence of features in the 1100–1300 cm^{-1} range. In the presence of air/water, the S–Cu interaction is often a precursor to the formation of metallo-sulfites or sulfates. Water can act as a nucleophile, attacking the S atom that is already activated by its coordination to Cu.

The persistence of these peaks in the "in air" spectrum suggests that SO_2 is not displaced by water, indicating a high affinity of the MOF for SO_2 or potentially a cooperative adsorption mechanism. The blue curve in the bottom plot of **Figure S76** shows a distinct peak at 1630 cm^{-1}, which is the characteristic H-O-H bending mode of adsorbed water. The peak is less pronounced in the N_2 plot (red), suggesting water confinement. The broad envelope between 3200 and 3500 cm^{-1} in the top two plots of **Figure S76** represents the O-H stretching. The increased intensity of these peaks in the presence of O_2 (blue spectrum) confirms the permanence of water in the channels, where the hydrogen-bonding network can promote proton transfers. The peaks in the 1200–1500 cm^{-1} and 1700–1800 cm^{-1} ranges, which show minor shifts or shape changes between the red and blue lines, indicate that water slightly perturbs the environment of the organic linkers within the MOF, inducing partial rotations of the rings.

Notwithstanding the presence of water, SO_2 molecules remain largely bound inside the framework. There is no direct evidence for water Co coordination in the IR spectra. The spectra suggest that while the framework is hydrated (broad 3400 cm^{-1} and 1630 cm^{-1} peaks), Co remains "shielded" within the TPz core, while the Cu centers and the basic ligand nitrogen atoms are the primary sites for water and activity.

The comparison between the ReaxFF MD simulations (**Figure S76** top left) and the experimental spectrum (**Figure S76** bottom left) shows a strong qualitative agreement, confirming that the molecular model effectively captures the framework's characteristics under different environments. The simulation (top) reproduces the main "fingerprint" regions of the N_2 (red) and air (blue) spectra. The broad envelope between the 1100 and 1500 cm^{-1} in the MD results matches the experimental ligand perturbations and SO_2 gas-phase regions. Even though the MD-calculated spectra show a slight frequency shift relative to experiments due to force field's parameterization, the relative peak spacing remains consistent. The experimental peak at 1211 cm^{-1} (attributed to Cu–SO_2) has a corresponding broad feature in the MD spectrum. This suggests the MD potential accurately models the coordination of sulfur to the Cu centers. The simulation captures the activity around 850–900 cm^{-1}. In the bottom plot, this is marked as a Cu–SO_2. The fact that the top plot shows a distinct change in slope indicates that the simulation accounts for the restricted motion of SO_2 when bound to metal. In the experimental "air" spectrum (blue), peaks at 1044 cm^{-1} and 962 cm^{-1} are assigned to SO_4^{2-} and SO_3^{2-}, respectively. The MD of the top plot's blue curve reflects these new vibrational modes. The increased "noise" and complexity in the simulated blue curve (especially below 1100 cm^{-1}) mirror the experimental appearance of these sulfur oxoanions.

Moreover, the MD simulation captured the perturbations in the TPz ligand (**Figure S76**). The trend of the top spectrum in the 1400–1560 cm^{-1} region matches well with the experimental shifts at 1520 and 1570 cm^{-1}. This observation suggests that the MD properly accounts for the strain

exerted by guest molecules, H_2O, and SO_2 on the TPz rings. The simulation successfully replicates the “smearing” and new features in the blue spectrum. Indeed, the specific experimental assignments for SO_4^{2-} (1044 cm^{-1}) and SO_3^{2-} (962 and 646 cm^{-1}) are well reproduced by the MD blue curve, which shows a significant increase in spectral density in these regions compared to the red curve. This result is direct evidence that the MD run successfully simulated the catalytic oxidation of SO_2 mediated by the Cu centers and humidity. The ReaxFF results for the TPz ligand perturbations (1300–1570 cm^{-1}) are particularly impressive. Indeed, in the experimental plot, the 1570 cm^{-1} and 1520 cm^{-1} peaks shift or change intensity in air. The blue curve in this region is noticeably more perturbed than the red one. Because ReaxFF accounts for charge redistribution, it is likely capturing the perturbation of the TPz nitrogen atoms by the generated H_2SO_x acids, which changes the ligand’s vibrational frequencies. The broad feature in the simulation near 1200 cm^{-1} matches the experimental Cu–SO_2 assignment (1211 cm^{-1}), and its persistence and evolution in the blue MD spectrum suggest that the simulation correctly maintains the metal-guest interaction as SO_2 evolved into SO_3^{2-}/SO_4^{2-}.

In sum, the ReaxFF model provides mechanistic validation for experimental assignments. It confirms that the Cu centers are the active sites for SO_2 conversion and that the resulting acidic environment is what causes the visible perturbations in the TPz ligand.

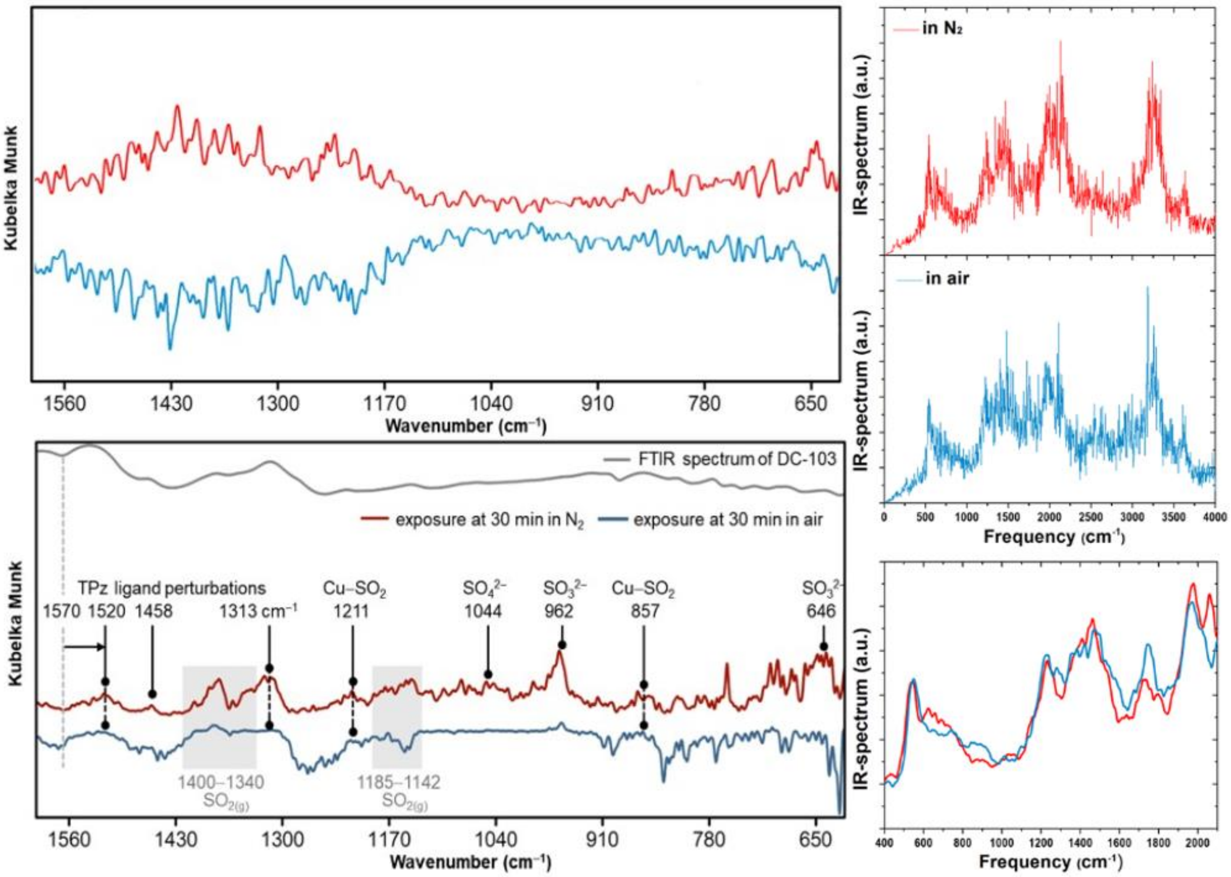

Figure S76. Left: simulated DRIFTS spectra of the MOF filled with guests (top) compared with the experimental DRIFTS difference spectra upon exposure to 400 ppm of SO_2 in dry nitrogen and dry air atmospheres (bottom). Right: simulated IR spectra from the MD.

S8.2. Quantum Chemistry calculations

Aiming to establish a rationale for the reactive dynamics-level results and to provide tentative quantitative estimates of interactions between adsorbate species and the MOF, we performed a series of DFT calculations, mainly using the bulk unit cells depicted in **Figure S28a,b**. It is worth mentioning that the additional data should be interpreted considering that the reduced models:

(i) are structurally constrained by strict periodic boundary conditions,

(ii) do not include entropic contributions because they are based on local energy minimizations (starting from snapshots extracted from portions of molecular dynamics trajectories),

(iii) were computed at very low concentrations of adsorbate species to reduce the overall computational cost.

Our analysis was further enriched by considering systems with a perpendicular space, thereby mimicking surface-like conditions. This modeling choice was necessary to properly sample interactions between adsorbate guest species and open metal sites, which may occur at the MOF surface or in bulk regions undergoing significant structural rearrangements. Indeed, the strict periodic boundary conditions imposed in bulk unit-cell models prevent the framework from undergoing the structural deformations observed upon adsorbate interaction in MD simulations. With these considerations in mind, the interaction landscape emerging from the present DFT simulations shows very good agreement with the trends observed in the radial distribution functions (RDFs) reported in **Figure S71**.

Considering the Co site, we found that, when accessible on the MOF surface, it can favorably adsorb several guest species, with adsorption energies in the range 0.3-0.8 eV (per molecule at very low concentration). Among the species analyzed in **Figure S77**(top), oxygen is the most strongly interacting, with adsorption energies around 0.8 eV. In contrast, the other molecules exhibit interaction energies in the 0.3–0.5 eV range. For SO_2, we explicitly considered both its native oxidation state (+4) and its highest oxidation state (+6) as H_2SO_4. This latter oxidized species has been identified during reactive MD simulations, and its formation has been investigated in a previous study on a related MOF system.[8] Overall, this picture is consistent with the RDF features associated with Co sites under low-humidity conditions for both bulk and surface systems, where a peak corresponding to O_2 adsorption is clearly visible. Interestingly, when explicitly considering the insertion of an O_2 molecule into the bulk MOF structure and its

adsorption atop a Co center via a nudged elastic band (NEB) simulation (**Figure S78**), we found that the associated activation energy was readily accessible under ambient conditions (barrier of about 0.2 eV). From a structural perspective, this event is accompanied by a relative lateral shift of the MOF layers, increasing layer staggering. This behavior, in agreement with the MD simulations, suggests that interactions with adsorbate guest species can induce significant structural rearrangements of the MOF, ultimately exposing the open metal sites and enhancing their ability to interact with guest molecules.

In contrast, the RDF reported in **Figure S71** indicates that the presence of SO_2 at bulk Co sites is disfavored, in agreement with the results obtained from local DFT relaxations. When attempting to adsorb an SO_2 molecule in the interlayer region, we identified a locally favorable interaction involving two vicinal Co centers (**Figure S79**); however, this configuration has a significantly higher energy than more favorable adsorption sites (discussed below). This energetic penalty can be attributed to the substantial increase in interlayer separation induced by the relatively large size of SO_2 compared with smaller guest molecules such as H_2O and O_2. Altogether, these results indicate that bulk Co sites are not readily accessible or energetically favorable for SO_2 sensing.

Compared with the Co site—rigidly located at the center of the MOF nodes and significantly shielded between the framework planes in the bulk—the Cu site is considerably more exposed to the external environment, being positioned midway along the linkers connecting the nodes. In addition, the Cu coordination environment can rotate more freely, allowing the site to reorient itself to maximize interactions with the incoming guest species. A further source of complexity arises from its intrinsic redox flexibility. As previously reported[8] and confirmed here by XPS analysis, this site can exist in both Cu(II) and Cu(I) oxidation states, the latter being associated with a reduced oxygen-coordination environment and a correspondingly stronger affinity for adsorbates.

In the configurations shown in **Figure S77** (bottom), which represent surface sites, possible reduction effects were not included, since the Cu ions are kept tetracoordinated. When located

on the MOF surface, the overall interaction scenario is characterized by a general decrease in adhesion energy relative to the Co site, with values ranging from 0.2 to 0.4 eV. As in the Co case, O_2 is the most strongly interacting species, with adhesion energies around 0.4 eV, consistent with the peaks observed in the RDF of **Figure S71** under low-humidity conditions. H_2O and SO_2 compete strongly for the Cu active site, with adsorption energies of 0.3-0.4 eV.

Regarding the bulk of the MOF system, the configuration shown in **Figure S80a** illustrates a Cu site simultaneously coordinated to both molecules in the unreacted state, in agreement with the DRIFTS fingerprints. Configurations **Figure S80b-d** depict structures associated with the oxidation pathway of SO_2 to HSO_3/SO_3 promoted by water. In these configurations, protons can either be stabilized on oxygen atoms coordinated to the Cu site, as shown in (b), or remain bound to the sulfur-containing species, as illustrated in (c) and (d). In configuration (c), HSO_3 interacts with a single Cu atom through its sulfur atom. In contrast, in configuration (d), the same species simultaneously interacts with two Cu atoms from adjacent MOF layers via its sulfur and oxygen atoms. The strong interaction of these oxidized species with the Cu center and its surrounding oxygen environment can significantly perturb the local coordination of the Cu center, potentially leading to permanent alterations in the MOF structure. This structural modification may be directly related to the experimentally observed poor recovery of the material under selected conditions.

All the collected data suggest that the pronounced RDF peaks observed for sulfur-containing species in the bulk upon interaction with Cu arise from two main effects: (i) the ability of these molecules to interact with the metal center through both sulfur and oxygen atoms, and (ii) their larger molecular size. As previously discussed, this larger size reduces their ability to interact with inner Co sites in the bulk relative to smaller molecules such as O_2 and H_2O, which can more readily penetrate and access the internal metal sites.

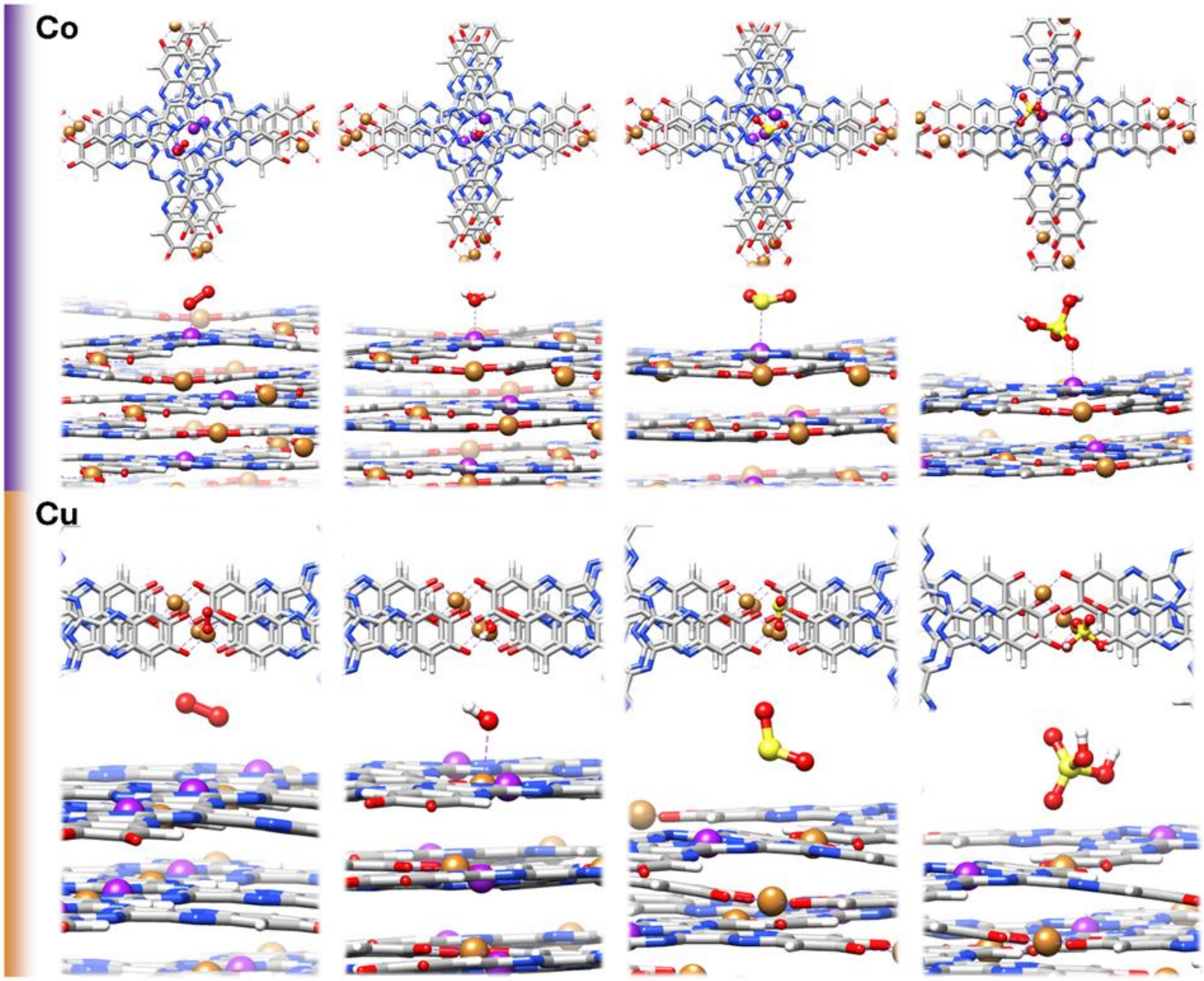

Figure S77. Top and side views of Cobalt (first row) and Copper (second row) sites of a surface MOF top-layer in the presence of adsorbed O_2, H_2O, SO_2, and H_2SO_4 molecules (from left to right). Color codes: C gray, H white, O red, N blue, Cu orange, Co purple, and S yellow.

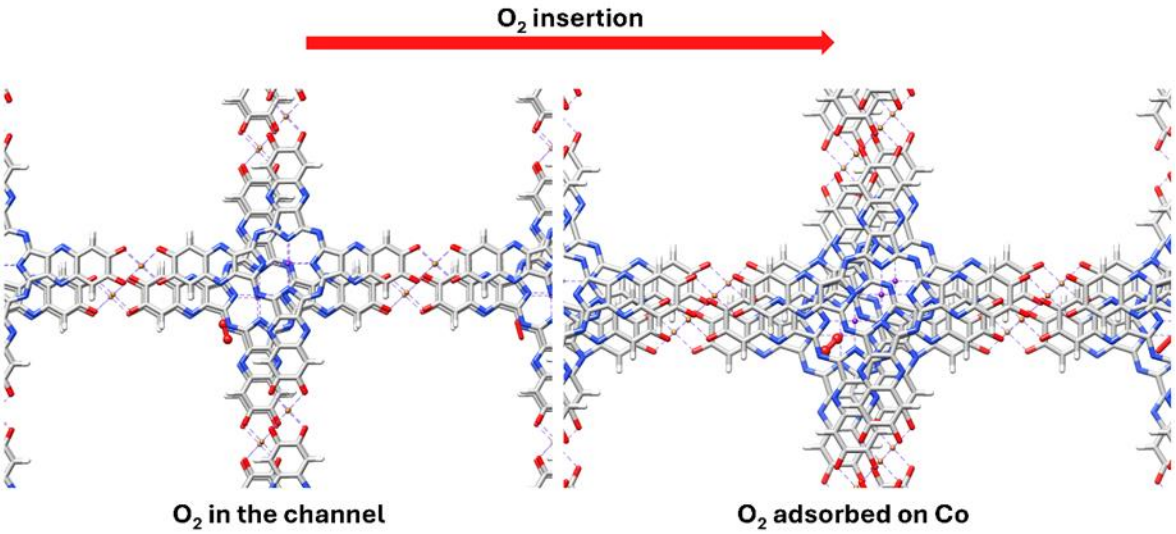

Figure S78. Progressive insertion of an O_2 molecule inside the MOF structure, being adsorbed on a Co metal center. Color code: C gray, H white, O red, N blue, Cu orange, and Co purple.

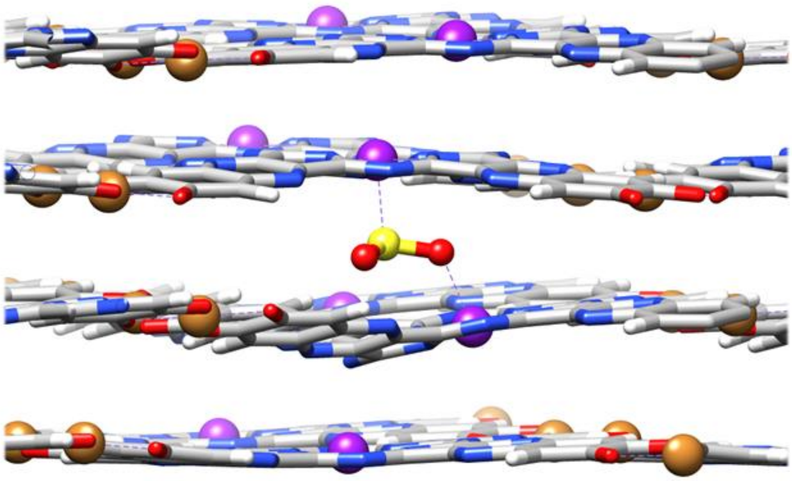

Figure S79. Insertion of a SO_2 molecule inside the MOF structure, being adsorbed via the interaction with two vicinal Co centers. Color code: C gray, H white, O red, N blue, Cu orange, Co purple, and S yellow.

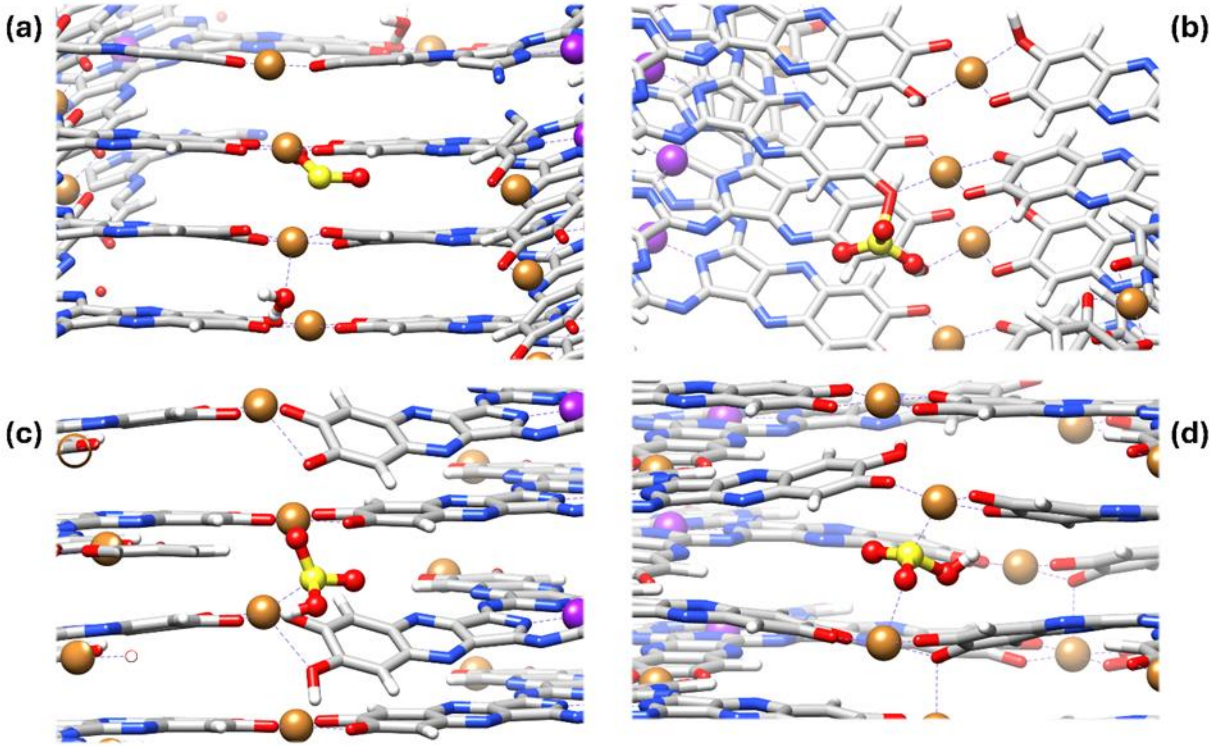

Figure S80. Selected adsorption geometries of SO_2 and H_2O molecules within the MOF structure in the vicinity of the Cu site, leading to both unreactive (a) and reactive (b–d) configurations. Color code: C gray, H white, O red, N blue, Cu orange, Co purple, and S yellow.

S9. Sensing responses of DC-103 towards other gases

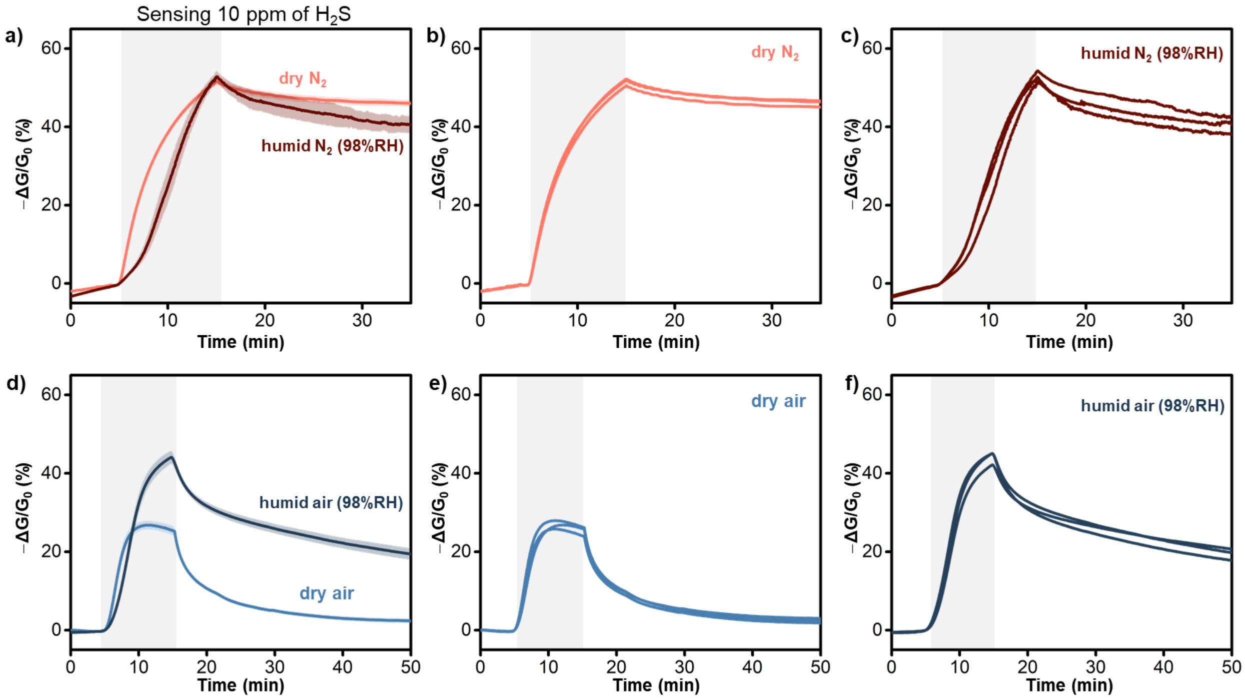

Figure S81. a) Averaged, b) and c) individual sensing responses of three devices of DC-103 towards 10 ppm of H_2S in dry and humid N_2 at 98% RH. d) Averaged, e) and f) individual sensing responses of three devices of DC-103 towards 10 ppm of H_2S in dry and humid air at 98% RH. The shaded curves represent the standard deviation of sensing responses from 3 devices and the gray shaded area represents the time of exposure of the devices to H_2S.

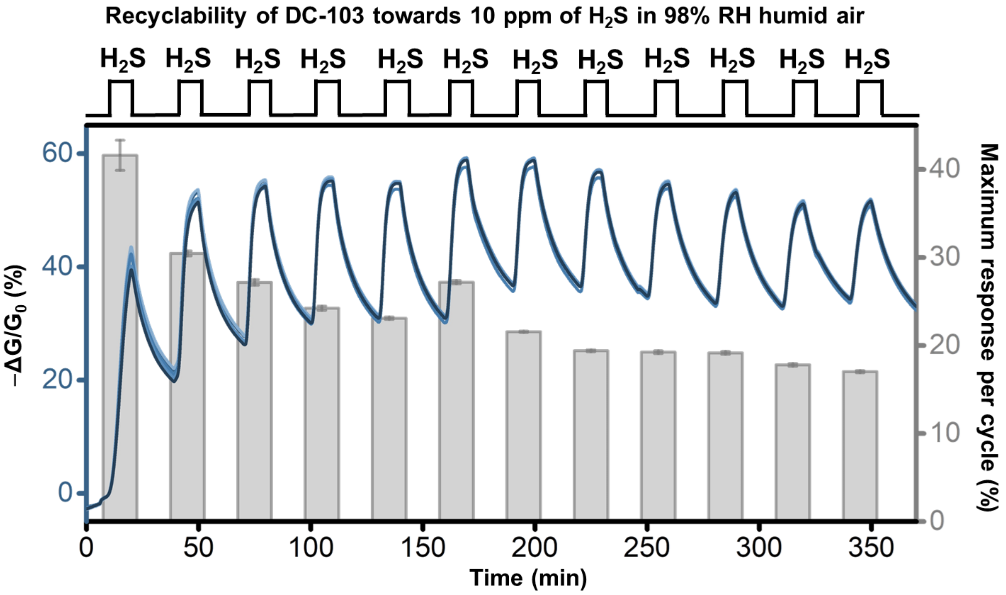

Figure S82. Curves of the averaged sensing responses of DC-103 with the standard deviation of three devices upon multiple exposure-recovery cycles towards 10 ppm of H_2S in 98% RH humid air, and bar graph showing the magnitude of the maximum response value across each cycle.

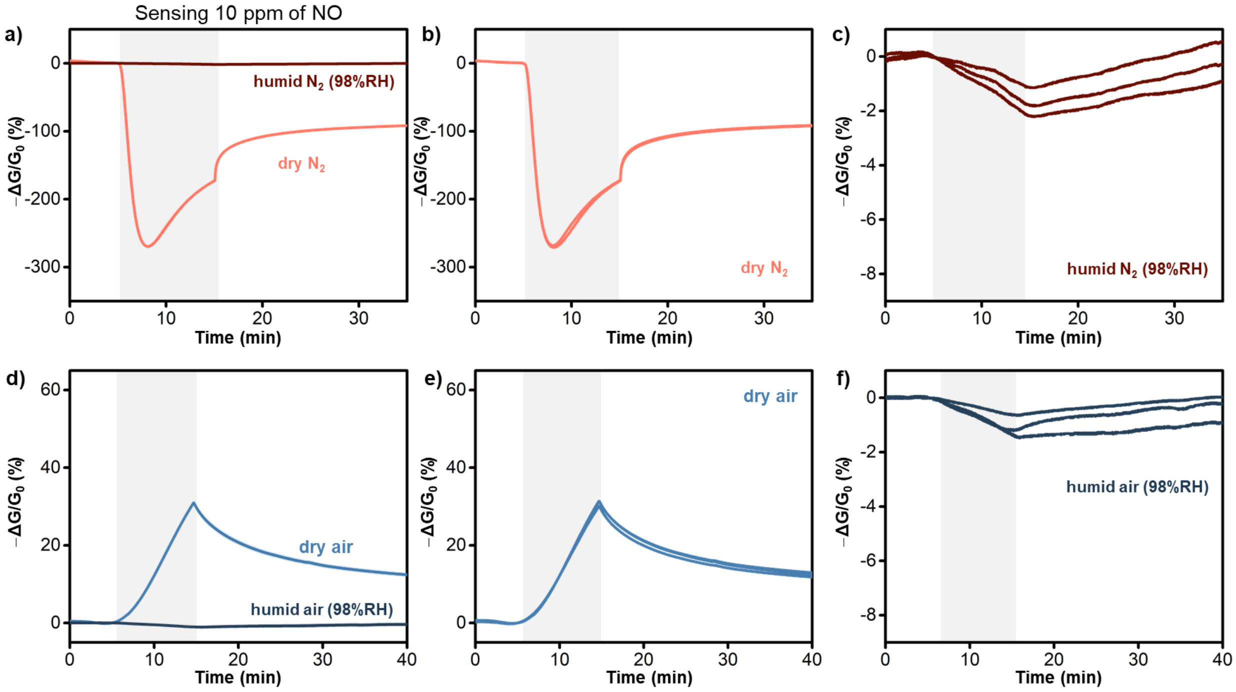

Figure S83. a) Averaged, b) and c) individual sensing responses of three devices of DC-103 towards 10 ppm of NO in dry and humid N_2 at 98% RH. d) Averaged, e) and f) individual sensing responses of three devices of DC-103 towards 10 ppm of NO in dry and humid air at 98% RH. The shaded curves represent the standard deviation of sensing responses from 3 devices and the gray shaded area represents the time of exposure of the devices to NO.

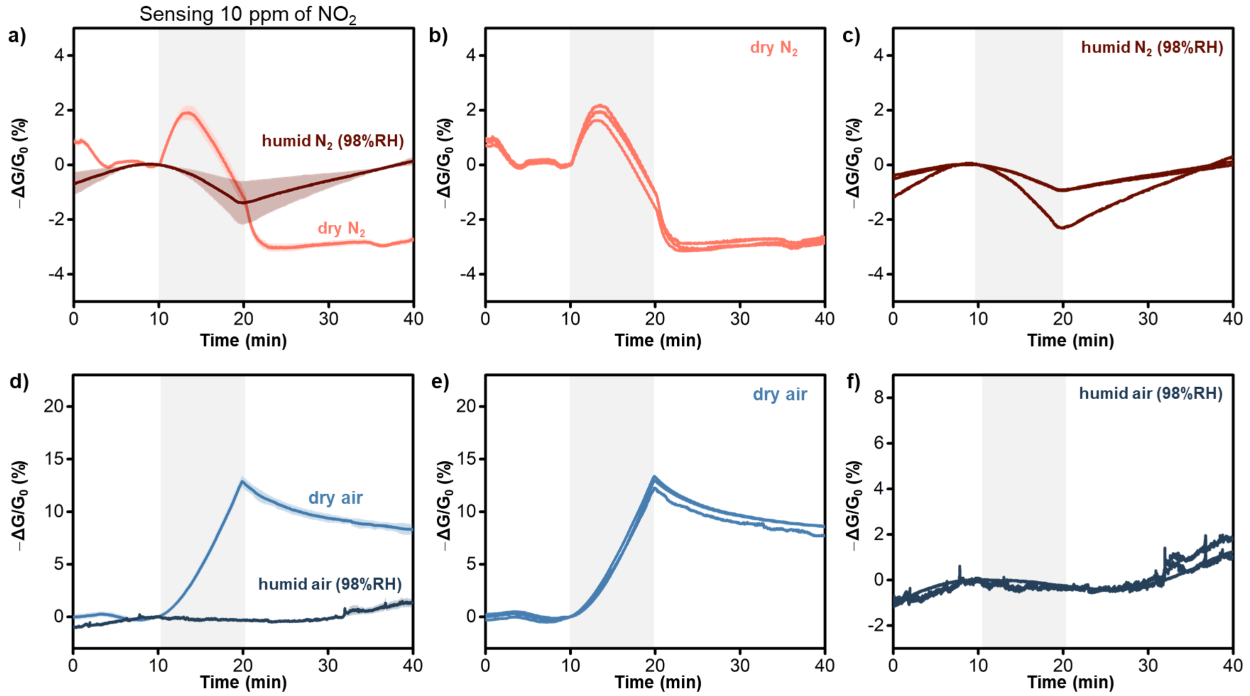

Figure S84. a) Averaged, b) and c) individual sensing responses of three devices of DC-103 towards 10 ppm of NO_2 in dry and humid N_2 at 98% RH. d) Averaged, e) and f) individual sensing responses of three devices of DC-103 towards 10 ppm of NO_2 in dry and humid air at 98% RH. The shaded curves represent the standard deviation of sensing responses from 3 devices and the gray shaded area represents the time of exposure of the devices to NO_2.

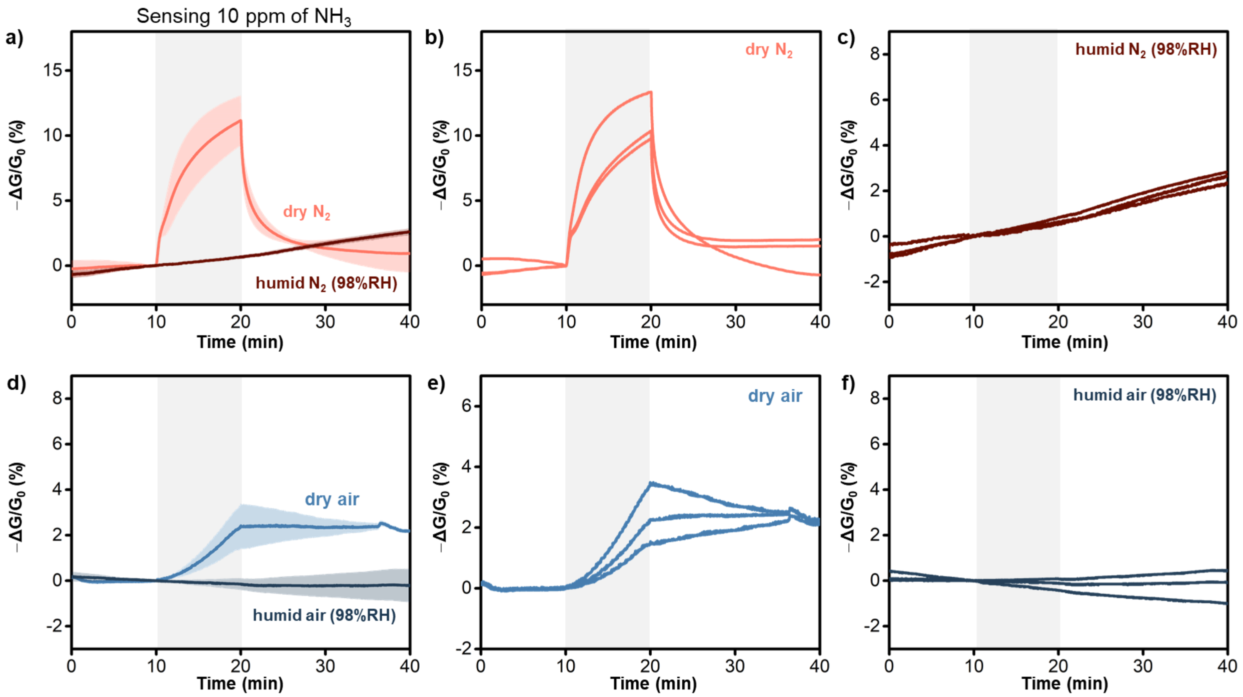

Figure S85. a) Averaged, b) and c) individual sensing responses of three devices of DC-103 towards 10 ppm of NH_3 in dry and humid N_2 at 98% RH. d) Averaged, e) and f) individual sensing responses of three devices of DC-103 towards 10 ppm of NH_3 in dry and humid air at 98% RH. The shaded curves represent the standard deviation of sensing responses from 3 devices and the gray shaded area represents the time of exposure of the devices to NH_3.

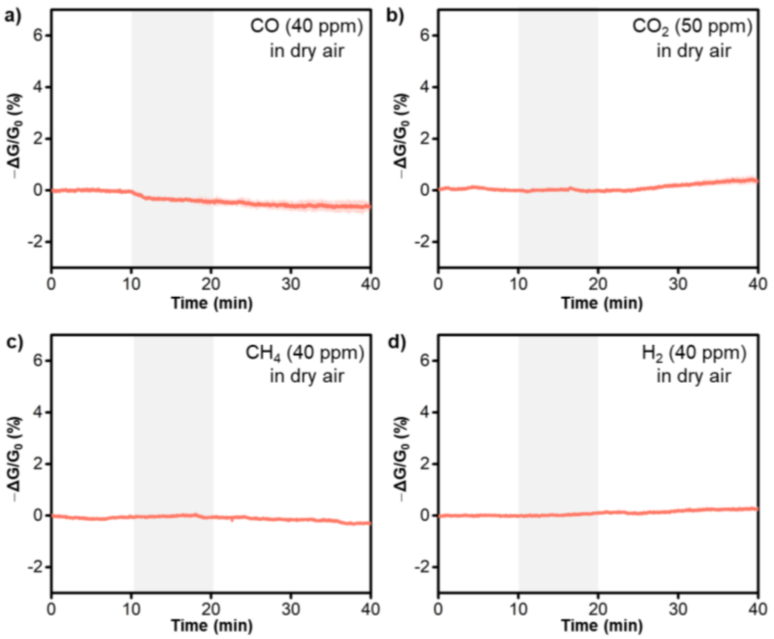

Figure S86. Averaged sensing responses of three devices of DC-103 towards a) 40 ppm of carbon monoxide (CO), b) 50 ppm of carbon dioxide (CO_2), c) 40 ppm of methane (CH_4), and d) 40 ppm of hydrogen gas (H_2) in dry air.

S10. SO_2 and H_2S Sensing of Control Materials

$CoTPz(OH)_8$ was synthesized as described in **Section S2**, CoPc-Cu-MOF was synthesized using a previously reported procedure,[40] and DC-102 and DC-100 were synthesized similar to the previously reported procedures.[2] Chemiresistive devices of each control material were prepared by sonicating a 1 mg/mL aqueous solution for around 2 hours to form homogeneous suspensions, followed by dropcasting 25 μL of the latter onto 5-μm gold interdigitated electrodes (IDE), and allowing them to dry under vacuum for 2 hours. The resistance of the devices were in the range of 0.1 MΩ, thus the following sensing experiments were collected at a driving voltage of 1V.

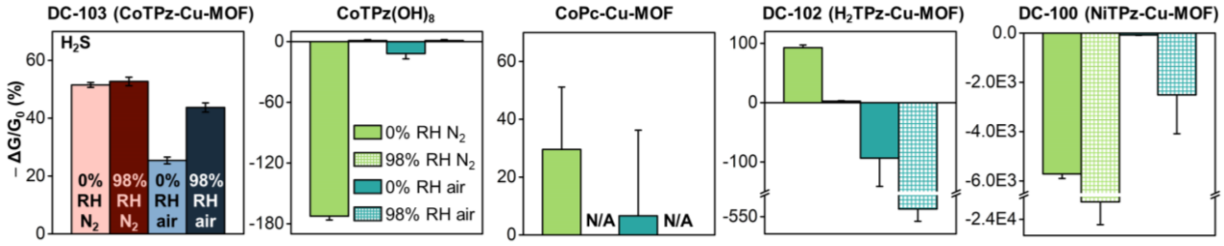

Figure S87. Bar graphs showing the magnitude of sensing response of various materials towards 10 ppm of H_2S after 10 minutes of exposure to the analyte gas in dry N_2, 98% RH humid N_2, dry air, and 98% RH humid air environments. Error bars correspond to the standard deviation from three devices.

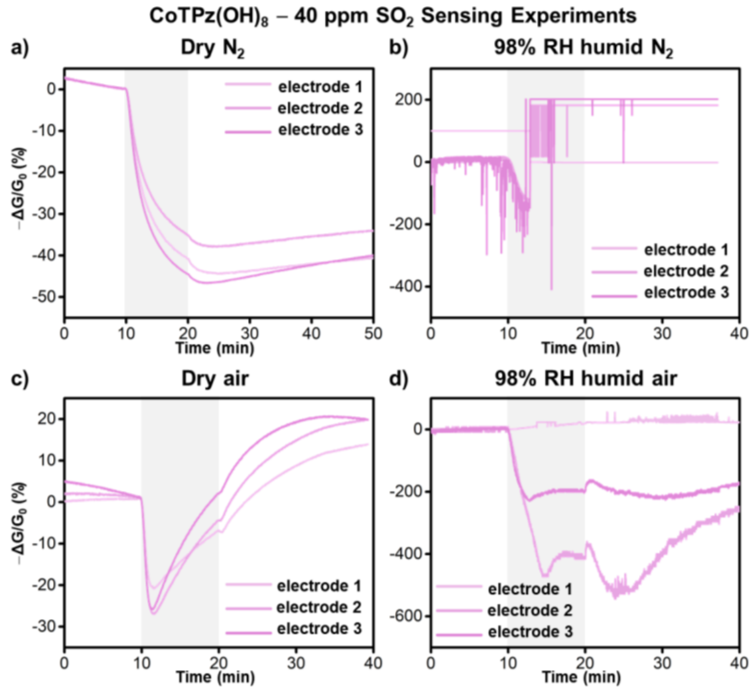

Figure S88. Individual sensing responses of three devices of CoTPz(OH)$_8$ towards 10 ppm of SO_2 in a) dry N_2, b) 98% RH humid N_2, c) dry air, and d) 98% RH humid air. The gray shaded area represents the time of exposure of the devices to SO_2.

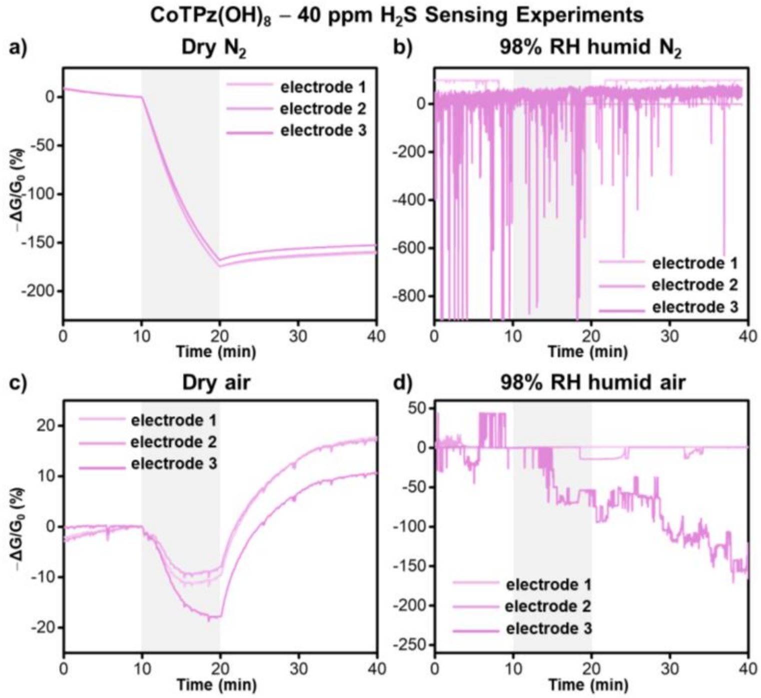

Figure S89. Individual sensing responses of three devices of CoTPz(OH)$_8$ towards 10 ppm of H_2S in a) dry N_2, b) 98% RH humid N_2, c) dry air, and d) 98% RH humid air. The gray shaded area represents the time of exposure of the devices to H_2S.

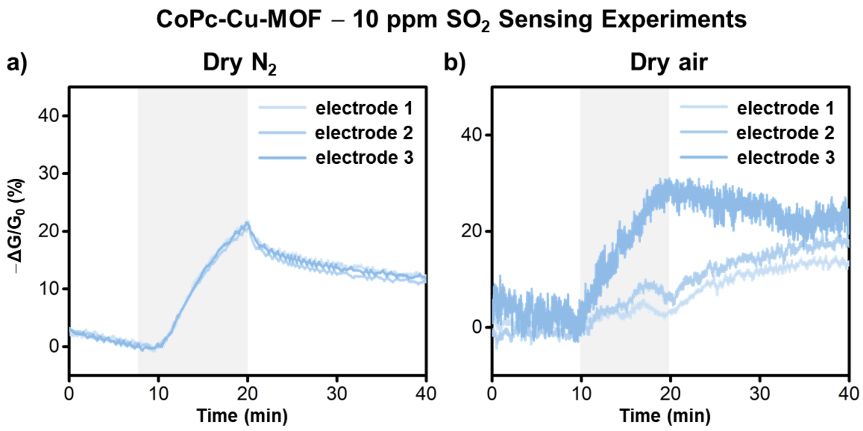

Figure S90. Individual sensing responses of three devices of CoPc-Cu-MOF towards 10 ppm of SO_2 in a) dry N_2 and b) dry air. The gray shaded area represents the time of exposure of the devices to SO_2.

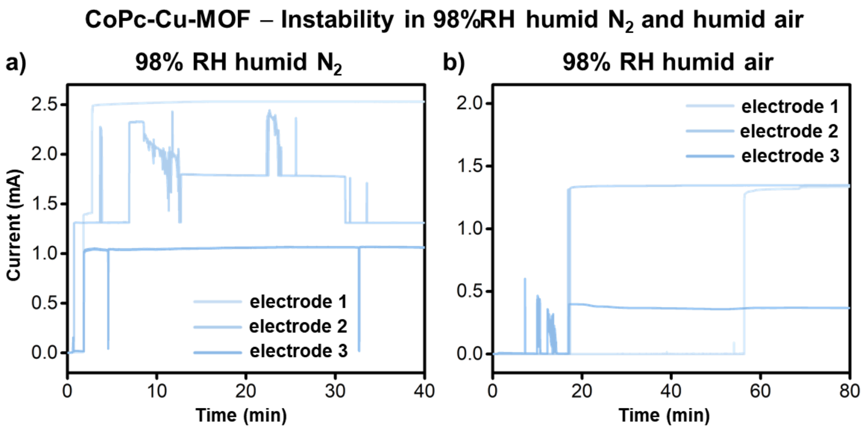

Figure S91. Recorded current measurements with time upon the exposure of three devices of CoPc-Cu-MOF towards 98% RH humid a) N_2 and b) air, showing the instability of current measurements. The step-wise rapid increase in current to mA-range values signifies the short-circuiting of the electrodes upon high humidity exposure, thus becoming no longer suitable for sensing experiments.

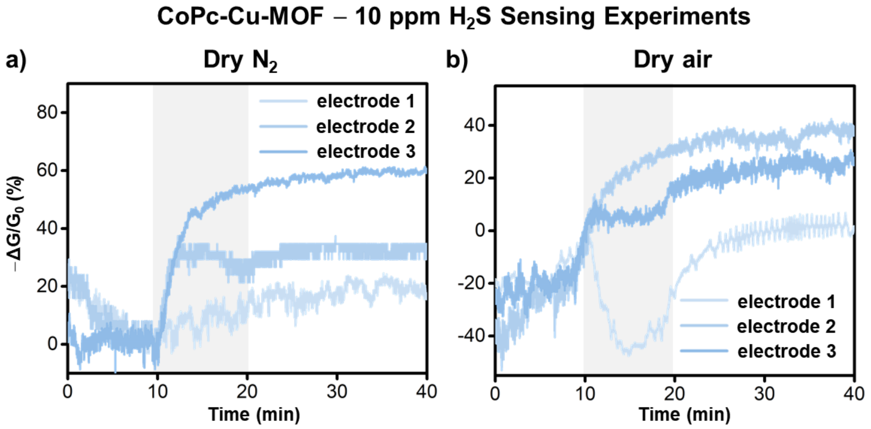

Figure S92. Individual sensing responses of three devices of CoPc-Cu-MOF towards 10 ppm of H_2S in a) dry N_2 and b) dry air. The gray shaded area represents the time of exposure of the devices to H_2S.

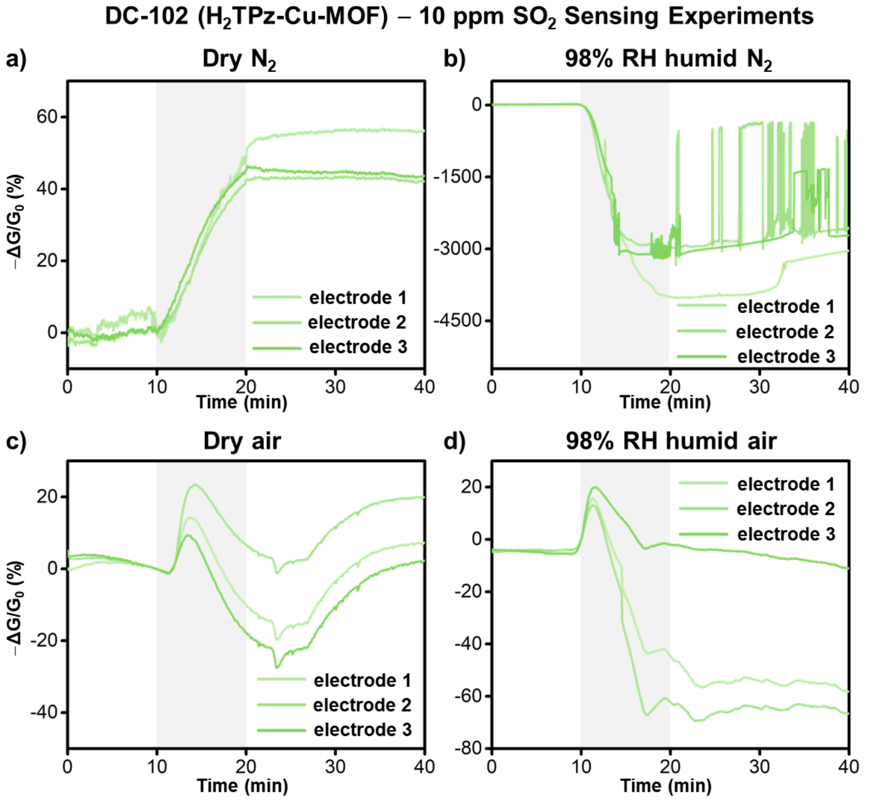

Figure S93. Individual sensing responses of three devices of DC-102 towards 10 ppm of SO_2 in a) dry N_2, b) 98% RH humid N_2, c) dry air, and d) 98% RH humid air. The gray shaded area represents the time of exposure of the devices to SO_2.

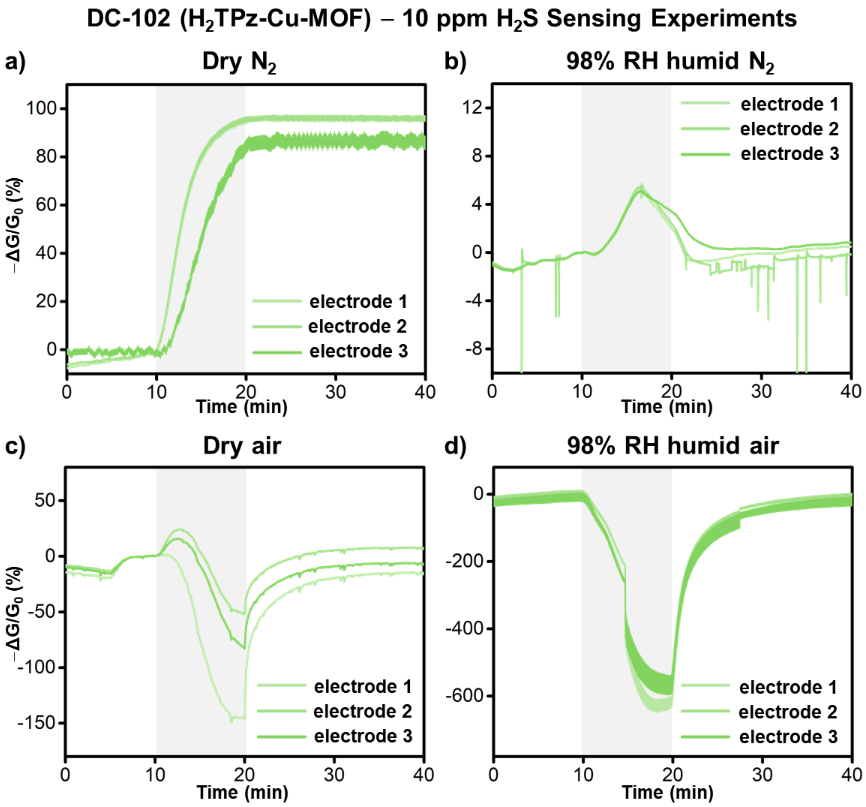

Figure S94. Individual sensing responses of three devices of DC-102 towards 10 ppm of H_2S in a) dry N_2, b) 98% RH humid N_2, c) dry air, and d) 98% RH humid air. The gray shaded area represents the time of exposure of the devices to H_2S.

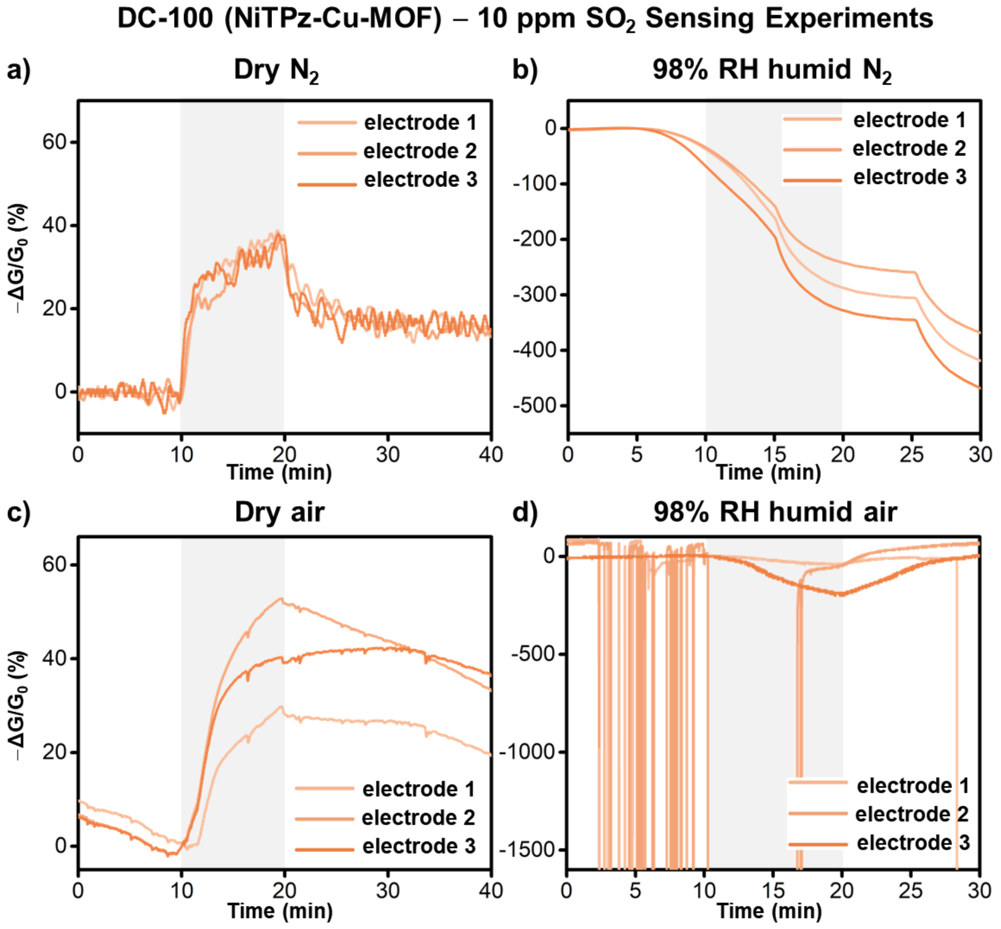

Figure S95. Individual sensing responses of three devices of DC-100 towards 10 ppm of SO_2 in a) dry N_2, b) 98% RH humid N_2, c) dry air, and d) 98% RH humid air. The gray shaded area represents the time of exposure of the devices to SO_2.

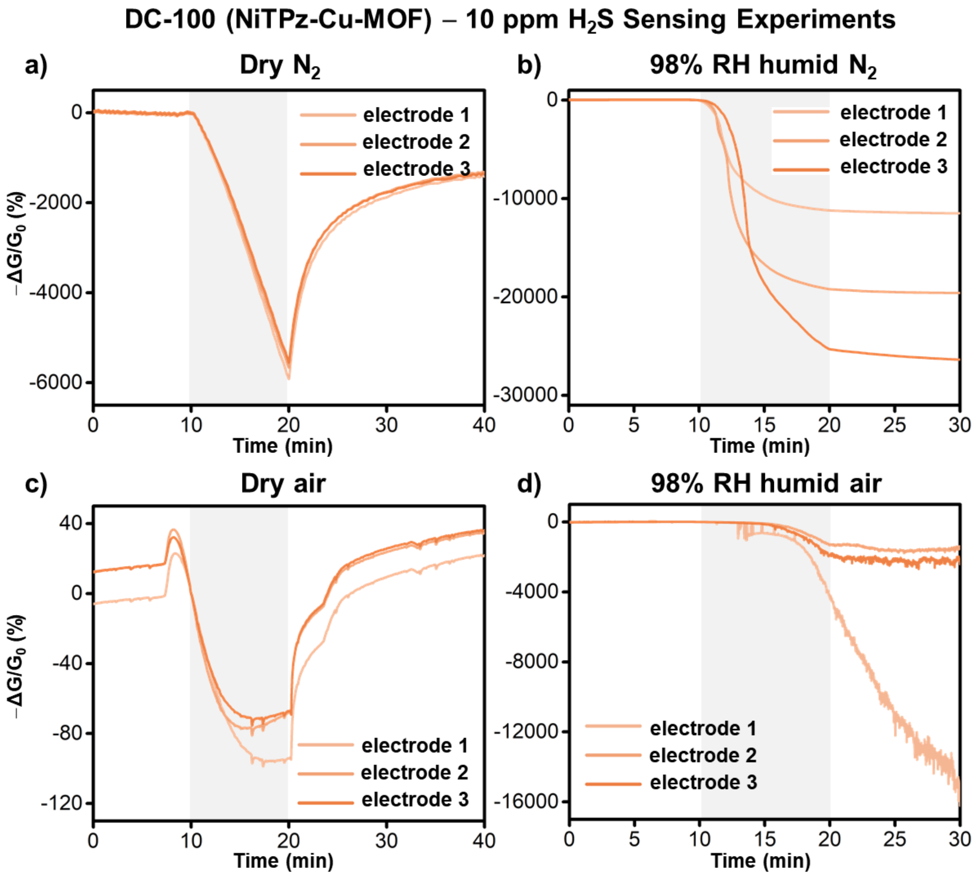

Figure S96. Individual sensing responses of three devices of DC-100 towards 10 ppm of H_2S in a) dry N_2, b) 98% RH humid N_2, c) dry air, and d) 98% RH humid air. The gray shaded area represents the time of exposure of the devices to H_2S.

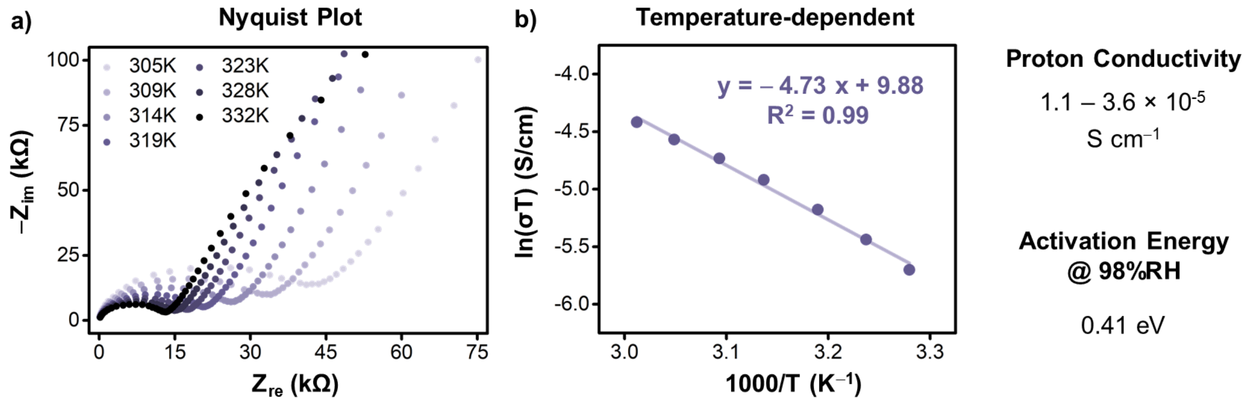

Figure S97. a) Nyquist plot of DC-100 at temperatures ranging between 305 and 332K, and b) temperature-dependent conductivity calculations, allowing for the determination of proton conductivity values ranging between 1.1 and 3.6 × 10^{-5} S cm^{-1} across the temperature ranges and an activation energy of 0.41 eV in a saturated (98% RH) humid environment.

S11. Impedance measurements

S11.1. General Experimental Notes

Electrochemical impedance spectroscopy (EIS) measurements were collected across the ends of gold interdigitated electrodes, onto which MOF suspensions were dropcast, following the procedure described in **Section S6.1.1**. Measurements were acquired over a frequency range of 0.1 Hz to 2 MHz using 3 points per decade, an AC amplitude of 100 mV rms, and 0 V DC bias. These parameters enabled a total data acquisition time of around 2 minutes per spectrum, allowing time-resolved measurements during toxic gas exposure.

For experiments under varying humidity conditions (**Figures S99-S103**), electrodes were equilibrated at the target %RH for around 15 minutes prior to data collection. For EIS sensing experiments (**Figures S104-S111**), measurements were collected after around 15 minutes of purging with background gas (equivalent to the equilibration and baseline performed in the chemiresistive sensing experiments). Five consecutive spectra were collected at 2-minute intervals for a total exposure time of 10 minutes, consistent with the experimental procedures followed in the chemiresistive sensing experiments.

EIS data were fitted using equivalent electrical circuits shown in **Figure S98**, selected based on minimizing the goodness-of-fit parameter (χ^2), incorporating resistors (R), constant phase elements (CPE), and Warburg diffusion (W) elements.[41]

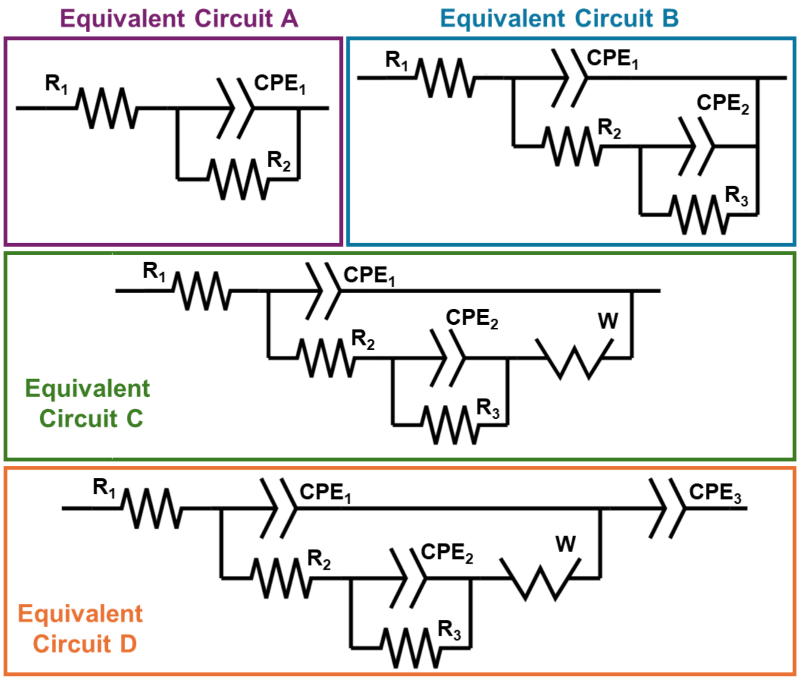

Figure S98. Equivalent electrical circuits used for fitting experimental EIS measurements of **Figures S99-S109**.

S11.2. Results and Discussion

S11.2.1. EIS measurements at different humidity levels

We observed that the shape of the Nyquist plots evolves with humidity for both DC-103 and DC-100 in N_2 and air atmospheres. For DC-103, Nyquist plots exhibit a well-defined semicircle under dry and low humidity conditions (up to around 50% RH), indicating dominance of charge transfer processes with minimal diffusion contributions (**Figure S99-S100**).[41] At 75% RH, the emergence of two partially resolved semicircles suggests the presence of multiple interfacial processes.[42] At 98% RH, the semicircular features diminish, and a pronounced 45° Warburg tail appears, consistent with increased diffusion contributions under saturated humidity.[41] Across all conditions, the real impedance (Z_{re}) and imaginary impedance ($-Z_{im}$) values remain within 0–40 kΩ, corresponding to resistance values within the same order of magnitude. These AC-derived resistance values are consistent with those extracted from DC I-V measurements (**Figure S31**).

In contrast, DC-100 exhibits a significantly stronger dependence on humidity. Diffusion contributions emerge as early as ~25% RH, as indicated by the appearance of a Warburg tail, and become dominant at ≥ 50-75% RH (**Figures S101-S102**). Notably, Z_{re} and $–Z_{im}$ values decrease substantially with increasing humidity, spanning several (3–4) orders of magnitude between dry and saturated humid conditions. This trend is consistent with DC resistance values obtained from I-V measurements (**Figure S103**).

These observations indicate that humidity-induced transport processes have minimal effect on the electronic conduction of DC-103 but strongly influence that of DC-100. We attribute this difference to the higher intrinsic electronic conductivity of DC-103, which remains dominant even under humid conditions, whereas in DC-100, comparable electronic and protonic contributions enable humidity-enhanced proton conduction to dominate at high %RH.

DC-103 under different humidity levels in N_2 (electrode 1)

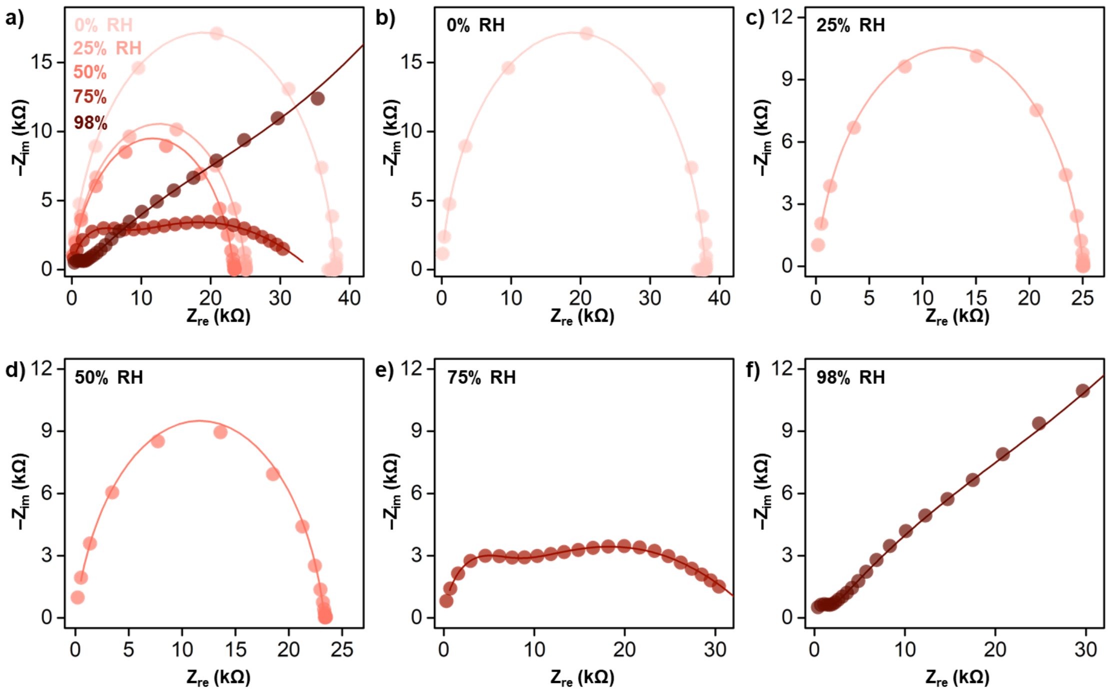

DC-103 under different humidity levels in N_2 (electrode 2)

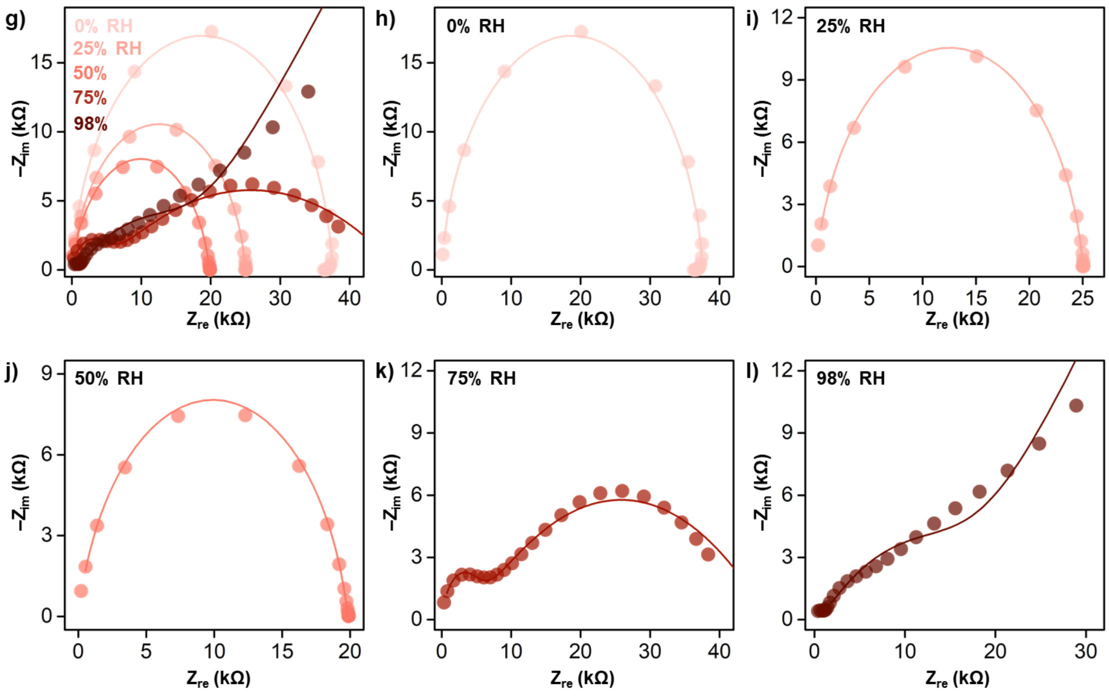

Figure S99. Nyquist plots of two independent DC-103 electrodes a,g) at different humidity levels in N_2, with separate graphs for each humidity level: b,h) 0% RH; c,i) 25% RH; d,j) 50% RH; e,k) 75% RH; and f,l) 98% RH in N_2. Dots represent experimental data, while lines represent fittings according to equivalent circuits shown in **Figure S98**.

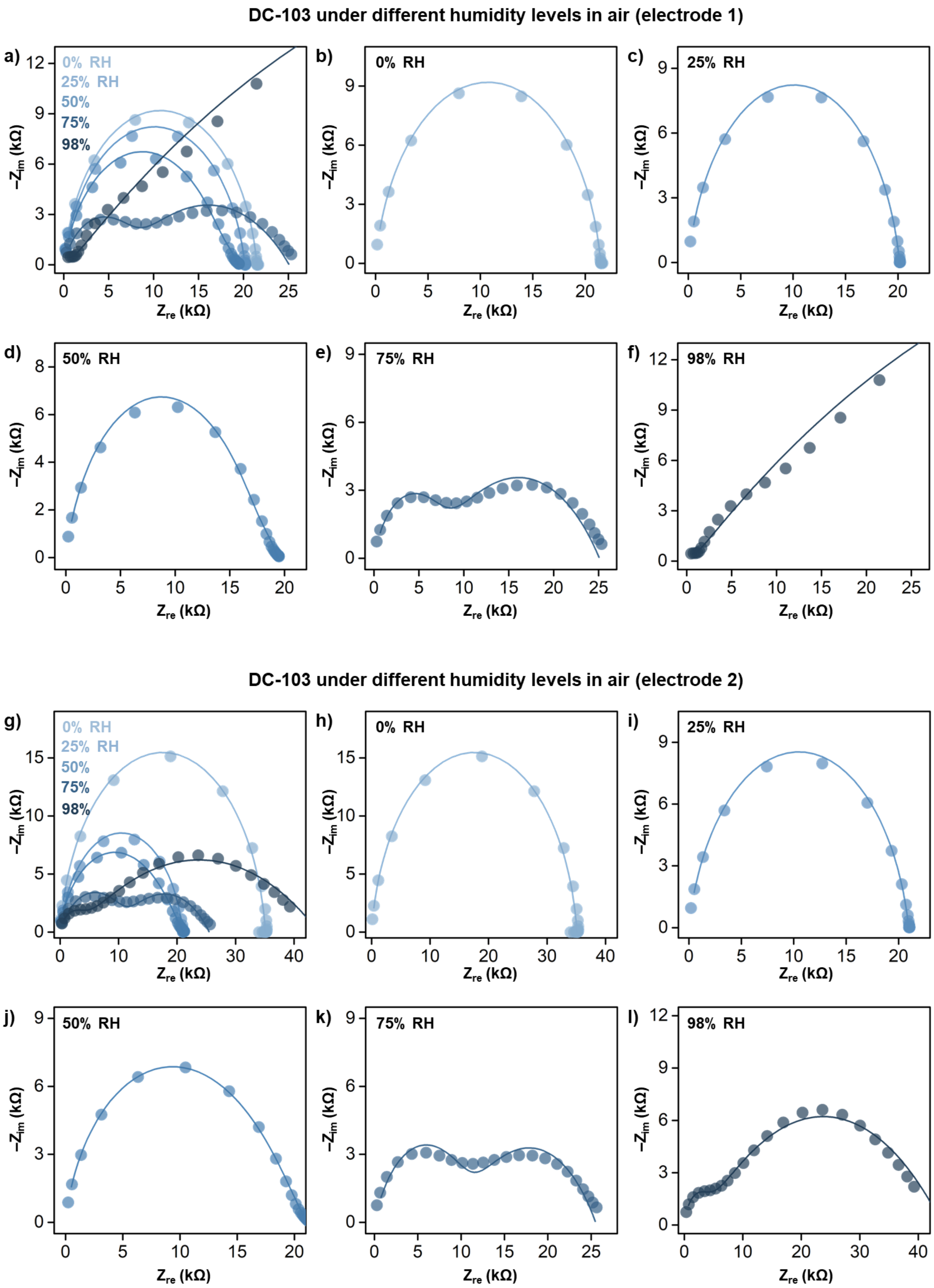

Figure S100. Nyquist plots of two independent DC-103 electrodes a,g) at different humidity levels in air, with separate graphs for each humidity level: b,h) 0% RH; c,i) 25% RH; d,j) 50% RH; e,k) 75% RH; and f,l) 98% RH in air. Dots represent experimental data, while lines represent fittings according to equivalent circuits shown in **Figure S98**.

DC-100 under different humidity levels in N_2 (electrode 1)

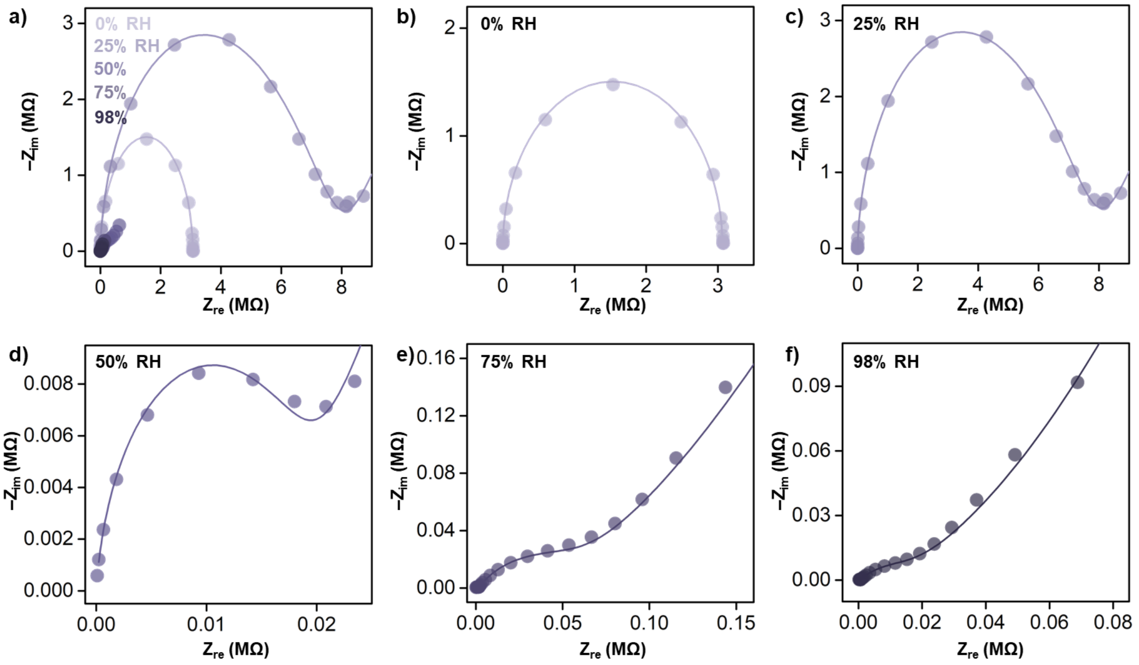

DC-100 under different humidity levels in N_2 (electrode 2)

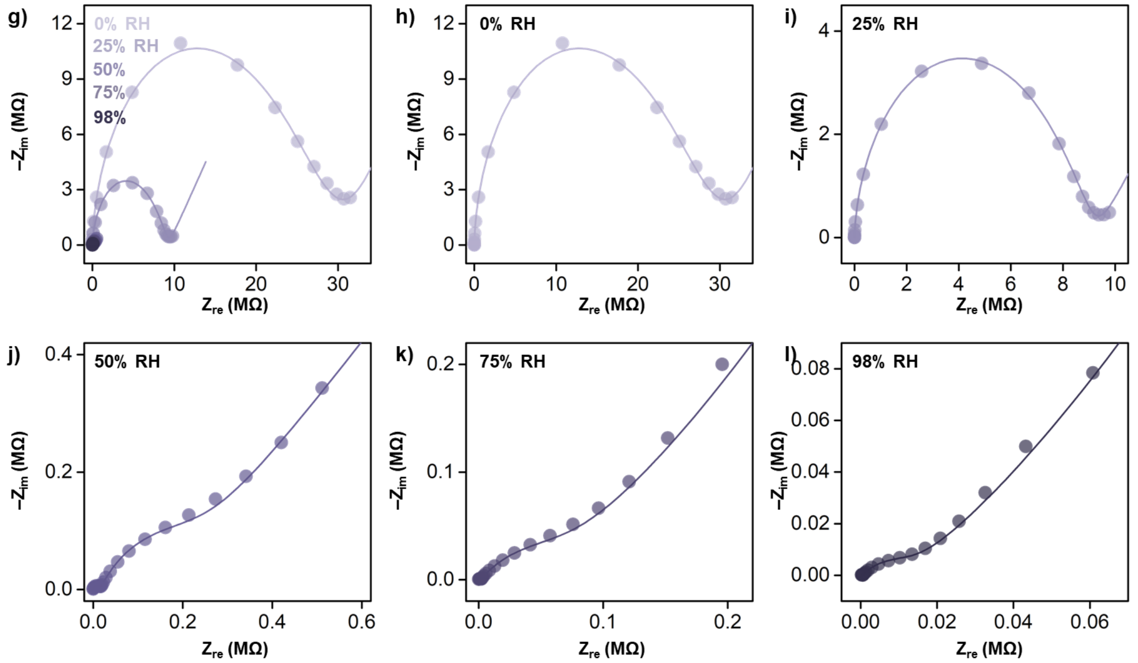

Figure S101. Nyquist plots of two independent DC-100 electrodes a,g) at different humidity levels in N_2, with separate graphs for each humidity level: b,h) 0% RH; c,i) 25% RH; d,j) 50% RH; e,k) 75% RH; and f,l) 98% RH in N_2. Dots represent experimental data, while lines represent fittings according to equivalent circuits shown in **Figure S98**.

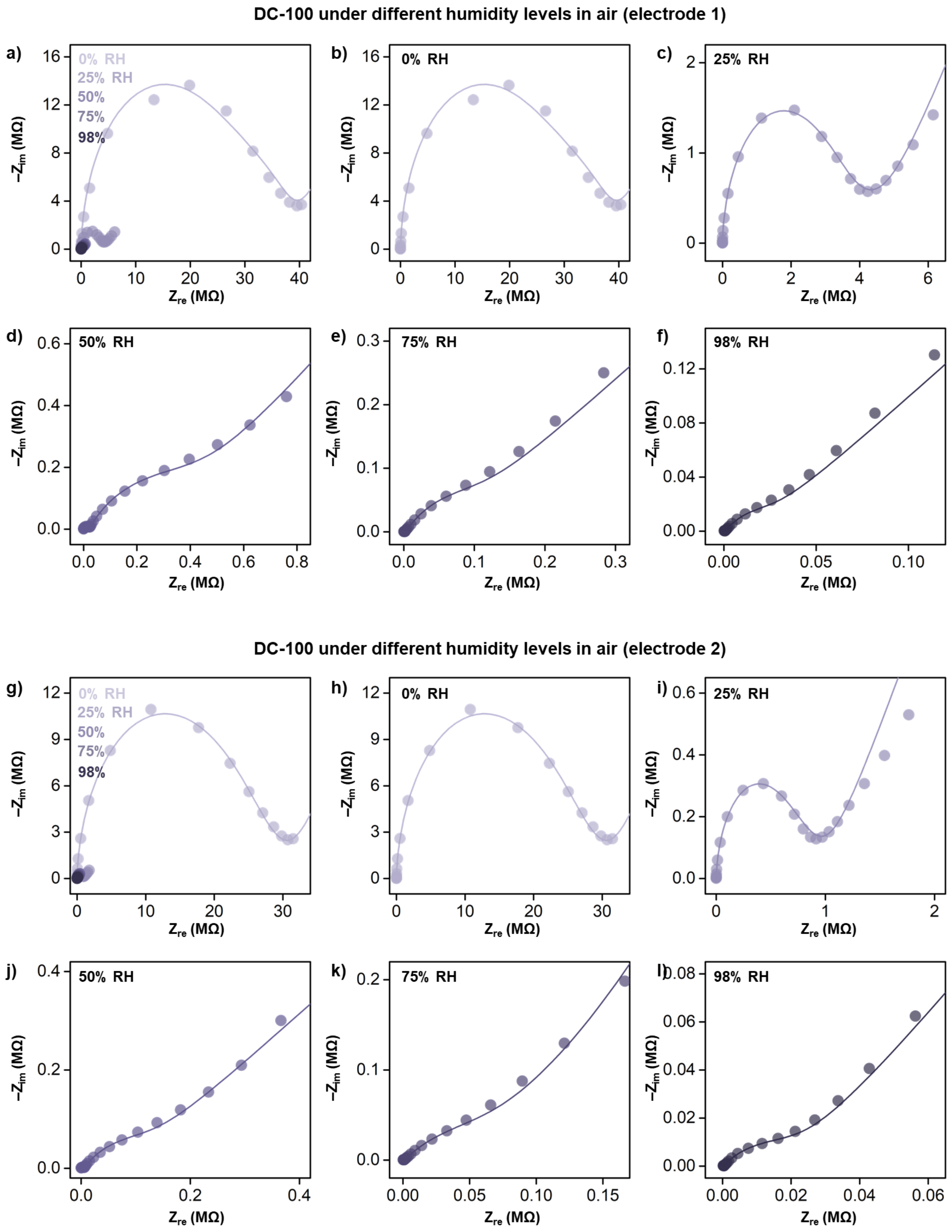

Figure S102. Nyquist plots of two independent DC-100 electrodes a,g) at different humidity levels in air, with separate graphs for each humidity level: b,h) 0% RH; c,i) 25% RH; d,j) 50% RH; e,k) 75% RH; and f,l) 98% RH in air. Dots represent experimental data, while lines represent fittings according to equivalent circuits shown in **Figure S98**.

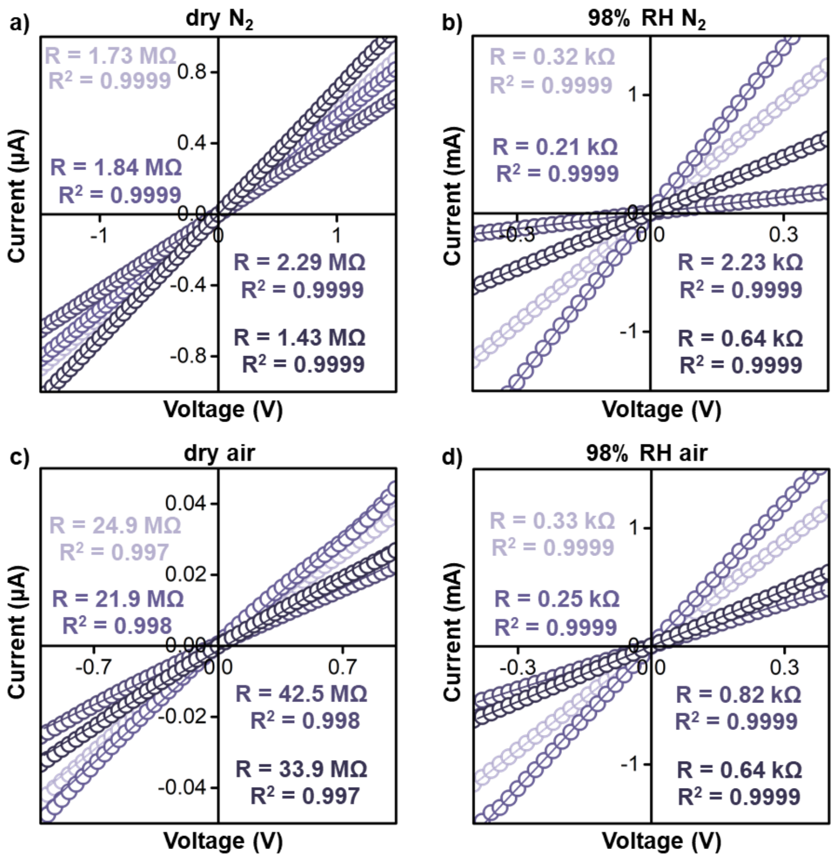

Figure S103. I-V curves of fabricated four replicate DC-100 devices in a) dry N_2, b) 98% RH N_2, c) dry air, d) 98% RH air.

S11.2.2. EIS measurements upon exposure to SO_2 in dry atmosphere

Time-resolved EIS measurements were further collected upon SO_2 exposure. For DC-103, exposure to 40 ppm of SO_2 in dry N_2 revealed minimal changes in the impedance spectra, whereas a higher concentration of 400 ppm led to a progressive increase in the Nyquist plot semicircle diameter with exposure time (**Figures S104a,c** and **S105a,c**), indicating an increase in resistance.[41] This trend is consistent with chemiresistive sensing results, where the resistance of DC-103 devices increased upon SO_2 exposure, observed as an increase in $-\Delta G/G_0$ (**Figure S65**). Analysis of frequency-dependent $-Z_{im}$ reveals a peak centered at ~10^4–10^5 Hz (log(f) = 4–5), corresponding to a relatively fast relaxation process (**Figures S104b,d** and **S105b,d**).[41]

For DC-100, exposure to 40 ppm SO_2 in dry N_2 similarly results in an increase in semicircle diameter (**Figure S106a,c**), consistent with the chemiresistive sensing responses (**Figure S95**). However, the $-Z_{im}$ peak occurs at significantly lower frequencies ~10^1–10^3 Hz (log(f) = 1–3), indicating a slower characteristic process (**Figure S106b,d**).[41] EIS measurements collected upon SO_2 exposure in dry air for both materials show trends consistent with those observed in dry N_2 (**Figures S107-S109**).

The different frequency ranges between DC-103 and DC-100 reflect variations in the characteristic time constants, with higher frequency features associated with faster interfacial charge transfer processes (in DC-103), and lower frequency features indicative of slower, mass transport-influenced processes (in DC-100).[41]

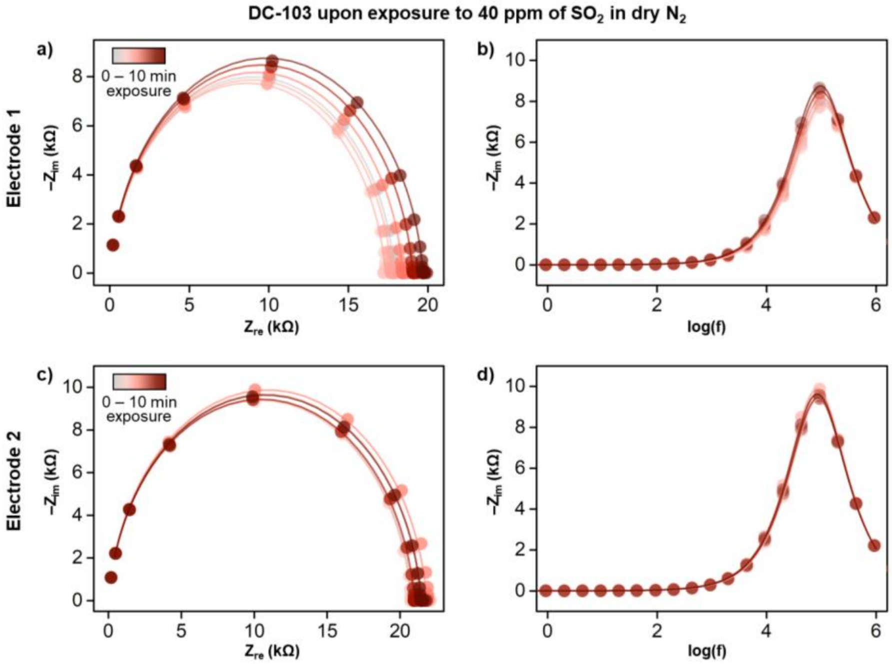

Figure S104. EIS measurements of DC-103 at 40 ppm SO_2 in dry N_2. a,c) Nyquist plots of two independent DC-103 electrodes collected at 2-minute intervals during exposure to 40 ppm of SO_2 in dry N_2. b,d) Corresponding plots of the negative imaginary impedance component as a function of logarithmic frequency for each electrode. Dots represent experimental data, while lines represent fittings according to equivalent circuits shown in **Figure S98**.

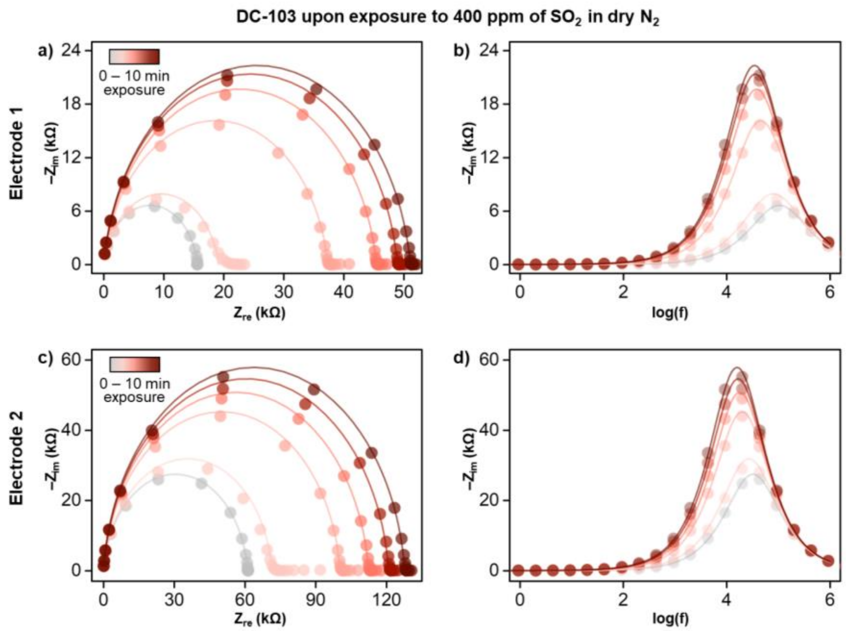

Figure S105. EIS measurements of DC-103 at 400 ppm SO_2 in dry N_2. a,c) Nyquist plots of two independent DC-103 electrodes collected at 2-minute intervals during exposure to 400 ppm of SO_2 in dry N_2. b,d) Corresponding plots of the negative imaginary impedance component as a function of logarithmic frequency for each electrode. Dots represent experimental data, while lines represent fittings according to equivalent circuits shown in **Figure S98**.

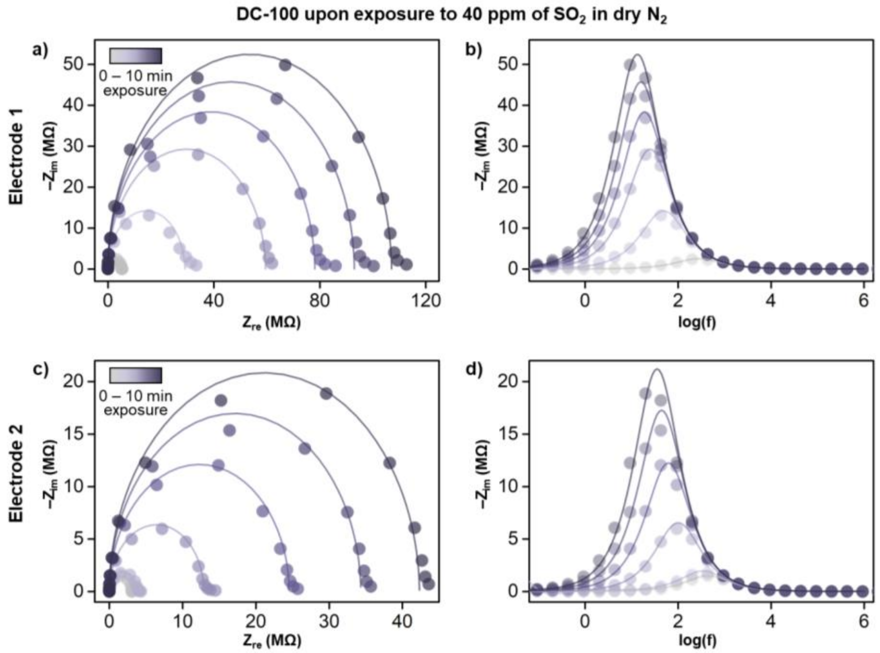

Figure S106. EIS measurements of DC-100 at 40 ppm SO_2 in dry N_2. a,c) Nyquist plots of two independent DC-100 electrodes collected at 2-minute intervals during exposure to 40 ppm of SO_2 in dry N_2. b,d) Corresponding plots of the negative imaginary impedance component as a function of logarithmic frequency for each electrode. Dots represent experimental data, while lines represent fittings according to equivalent circuits shown in **Figure S98**.

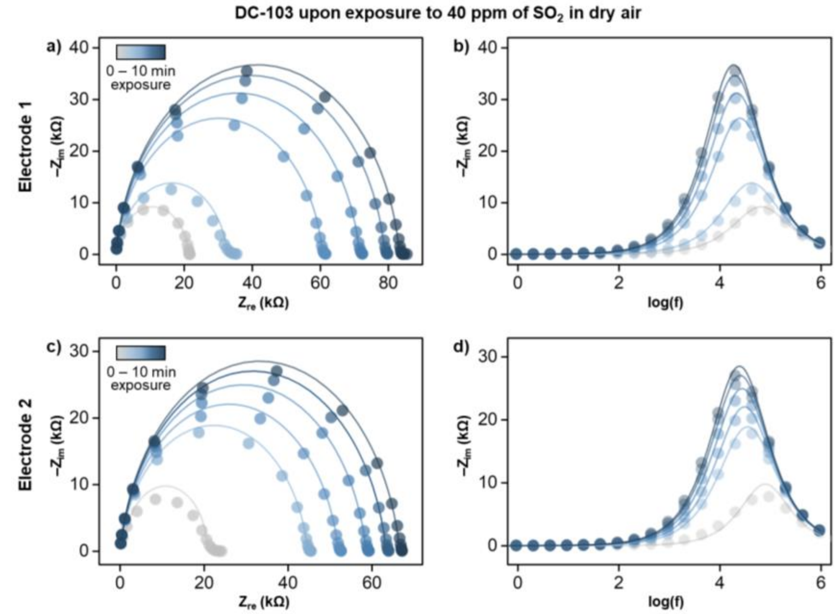

Figure S107. EIS measurements of DC-103 at 40 ppm SO_2 in dry air. a,c) Nyquist plots of two independent DC-103 electrodes collected at 2-minute intervals during exposure to 40 ppm of SO_2 in dry air. b,d) Corresponding plots of the negative imaginary impedance component as a function of logarithmic frequency for each electrode. Dots represent experimental data, while lines represent fittings according to equivalent circuits shown in **Figure S98**.

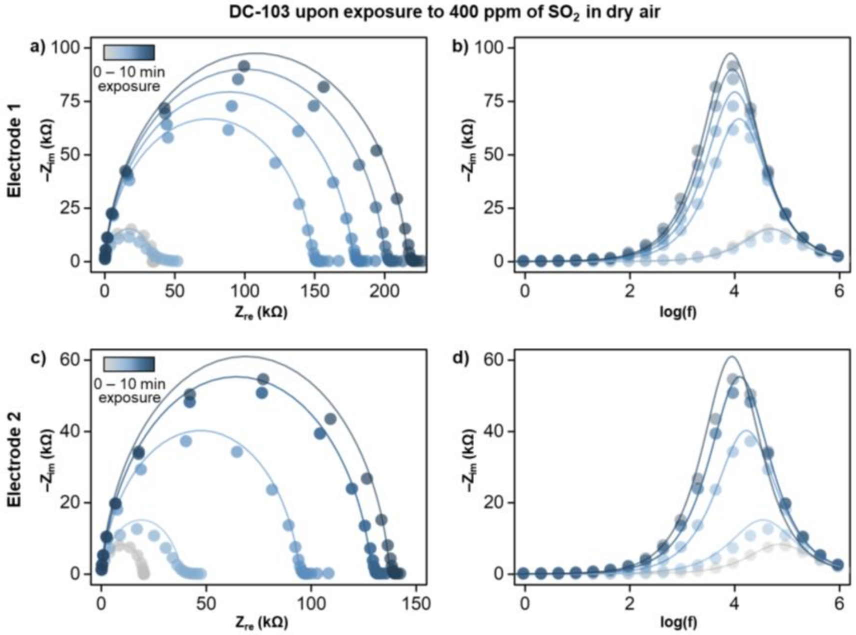

Figure S108. EIS measurements of DC-103 at 400 ppm SO_2 in dry air. a,c) Nyquist plots of two independent DC-103 electrodes collected at 2-minute intervals during exposure to 400 ppm of SO_2 in dry air. b,d) Corresponding plots of the negative imaginary impedance component as a function of logarithmic frequency for each electrode. Dots represent experimental data, while lines represent fittings according to equivalent circuits shown in **Figure S98**.

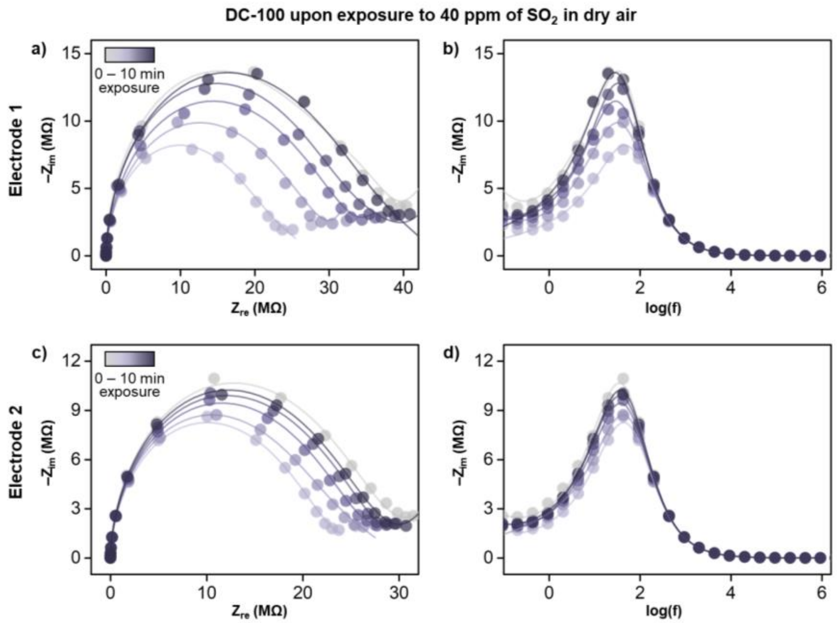

Figure S109. EIS measurements of DC-100 at 40 ppm SO_2 in dry air. a,c) Nyquist plots of two independent DC-100 electrodes collected at 2-minute intervals during exposure to 40 ppm of SO_2 in dry air. b,d) Corresponding plots of the negative imaginary impedance component as a function of logarithmic frequency for each electrode. Dots represent experimental data, while lines represent fittings according to equivalent circuits shown in **Figure S98**.

S11.2.3. EIS measurements upon exposure to SO_2 in humid atmosphere

Under humid conditions, DC-103 exhibits increasingly pronounced low-frequency tails upon exposure to 40 ppm of SO_2 at 98% RH in both N_2 and air (**Figure S110**), indicating enhanced contributions from diffusion or possible capacitive contributions.[41] In contrast, DC-100 spectra remain largely unchanged upon SO_2 exposure at high humidity levels (**Figure S111**), consistent with an already diffusion-dominated transport regime under these conditions.

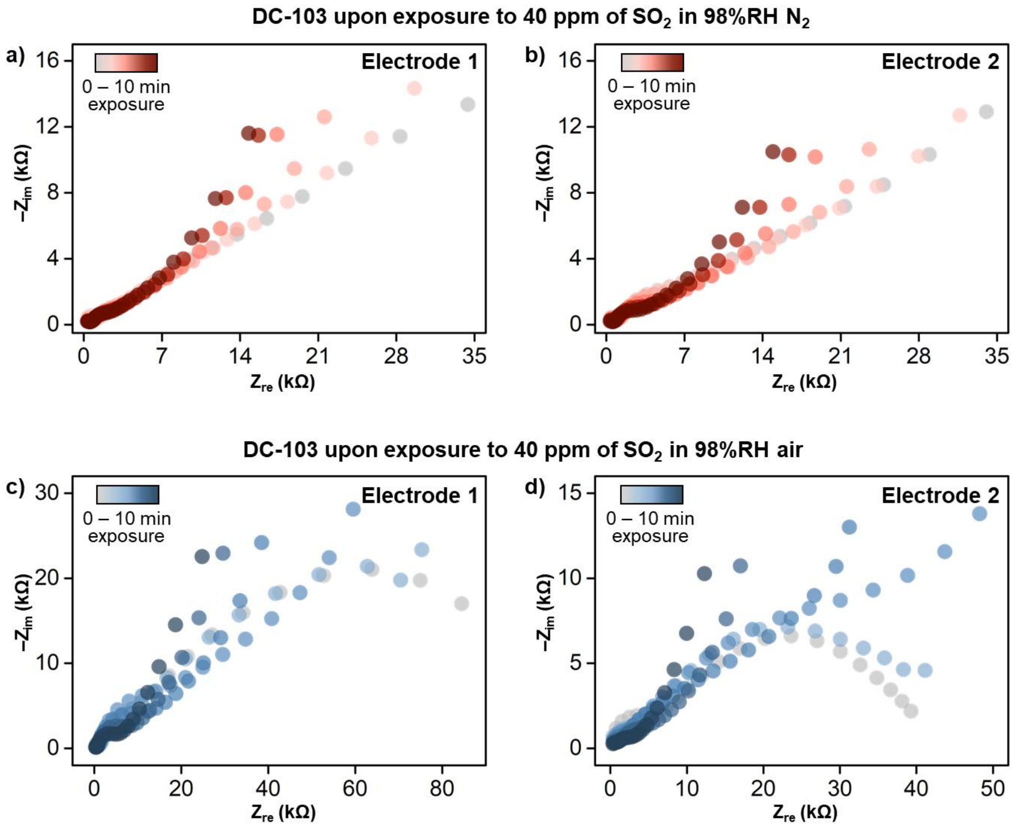

Figure S110. EIS measurements of DC-103 at 40 ppm SO_2 in humid N_2 and air. a,c) Nyquist plots of two independent DC-103 electrodes collected at 2-minute intervals during exposure to 40 ppm of SO_2 in 98% RH N_2. c,d) Nyquist plots of two independent DC-103 electrodes collected at 2-minute intervals during exposure to 40 ppm of SO_2 in 98% RH air.

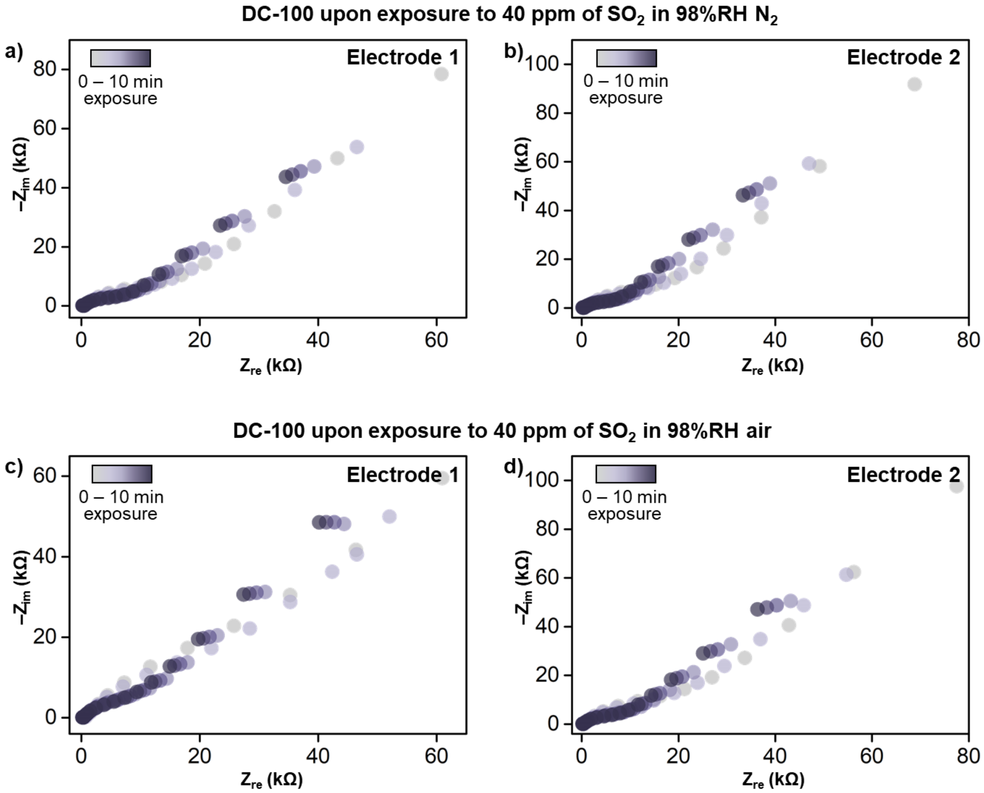

Figure S111. EIS measurements of DC-100 at 40 ppm SO_2 in humid N_2 and air. a,c) Nyquist plots of two independent DC-100 electrodes collected at 2-minute intervals during exposure to 40 ppm of SO_2 in 98% RH N_2. c,d) Nyquist plots of two independent DC-100 electrodes collected at 2-minute intervals during exposure to 40 ppm of SO_2 in 98% RH air.

S12. Suspension stability

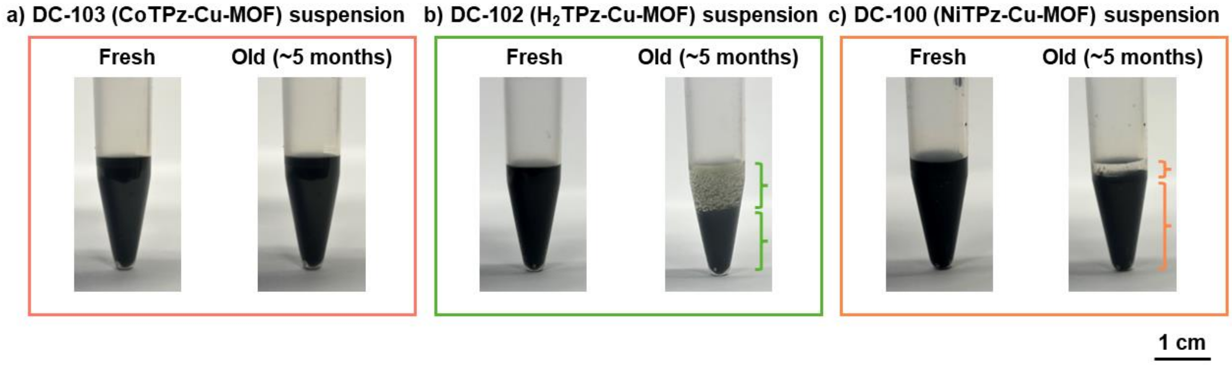

Figure S112. Images of fresh and 5 month-old suspensions of a) DC-103, b) DC-102, and c) DC-100 (with a concentration of 1 mg/1 mL of Milli-Q water). The images show the superior colloidal stability of the DC-103 suspension with respect to that of DC-102 and DC-100.

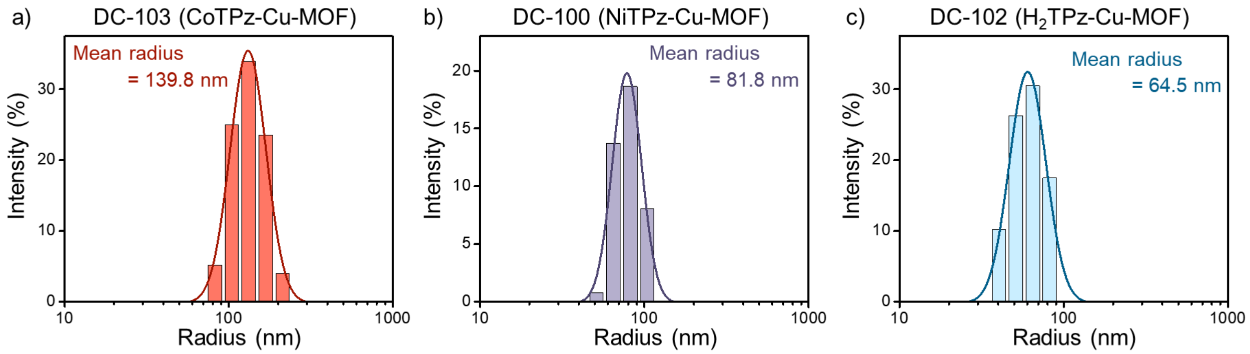

Figure S113. Dynamic light scattering (DLS) plots of a) DC-103, b) DC-100, and c) DC-102 MOF suspensions. The trend in larger crystal sizes of DC-103 compared to DC-100 and DC-102 is consistent with TEM images in **Figure S15-S16** and our previous report.[2] Slight discrepancies in mean values arise from inherent assumptions in DLS measurements that particles are spherical in shape.[43]

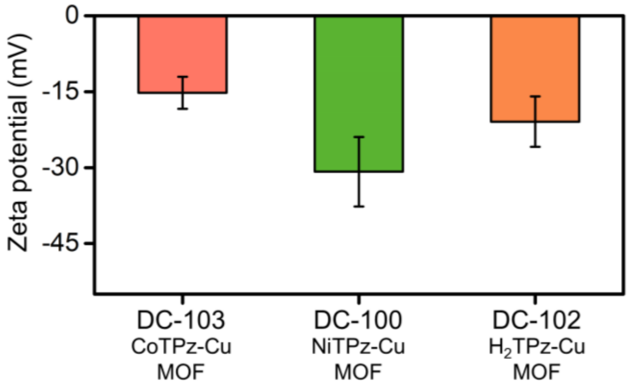

Figure S114. Bar graph showing average values of zeta potential for DC-103, DC-100, and DC-102, extracted from plots of **Figure S115**.

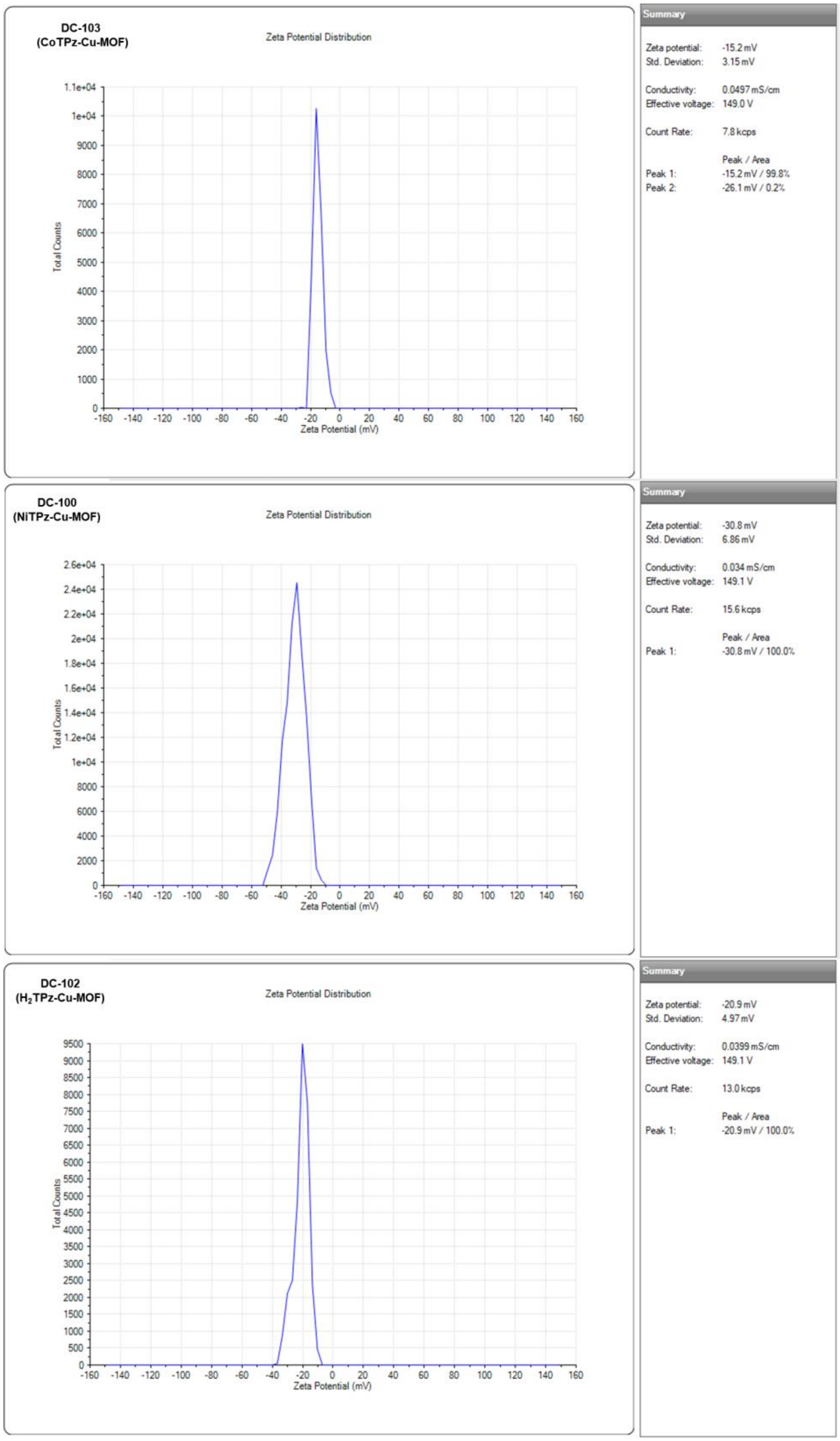

Figure S115. Raw data of zeta potential measurements of fresh DC-103, DC-100, and DC-102 MOF suspensions.

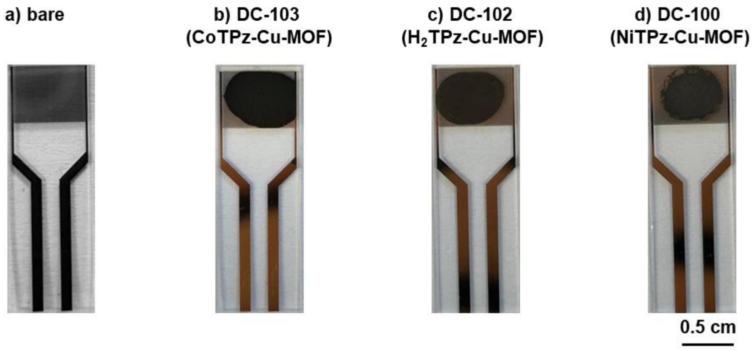

Figure S116. Images of a) bare gold IDE prior to dropcasting, as well as gold IDE with a dropcasted fresh suspension (volume = 25 μL of concentration 1 mg/ 1 mL of Milli-Q water) of a) DC-103, b) DC-102, and c) DC-100. After dropcasting, the electrodes were dried in a vacuum chamber for 2 hours.

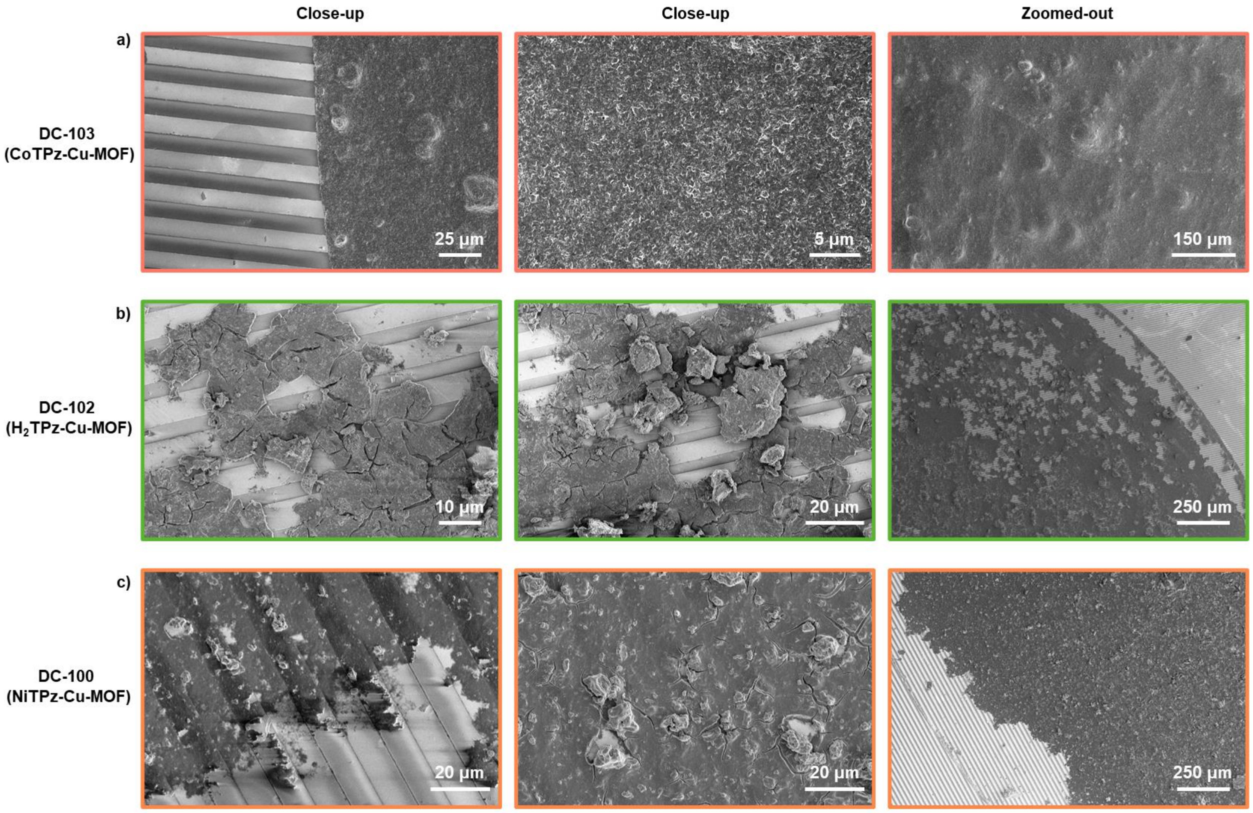

Figure S117. SEM images of devices after dropcasting suspensions of a) DC-103, b) DC-102, and DC-100 in close-up and zoomed-out views. The enhanced dispersion of the DC-103 suspension is observed as a uniform thin film upon dropcasting, unlike the other analogous MOFs where the deposition is not uniform and includes cracks.

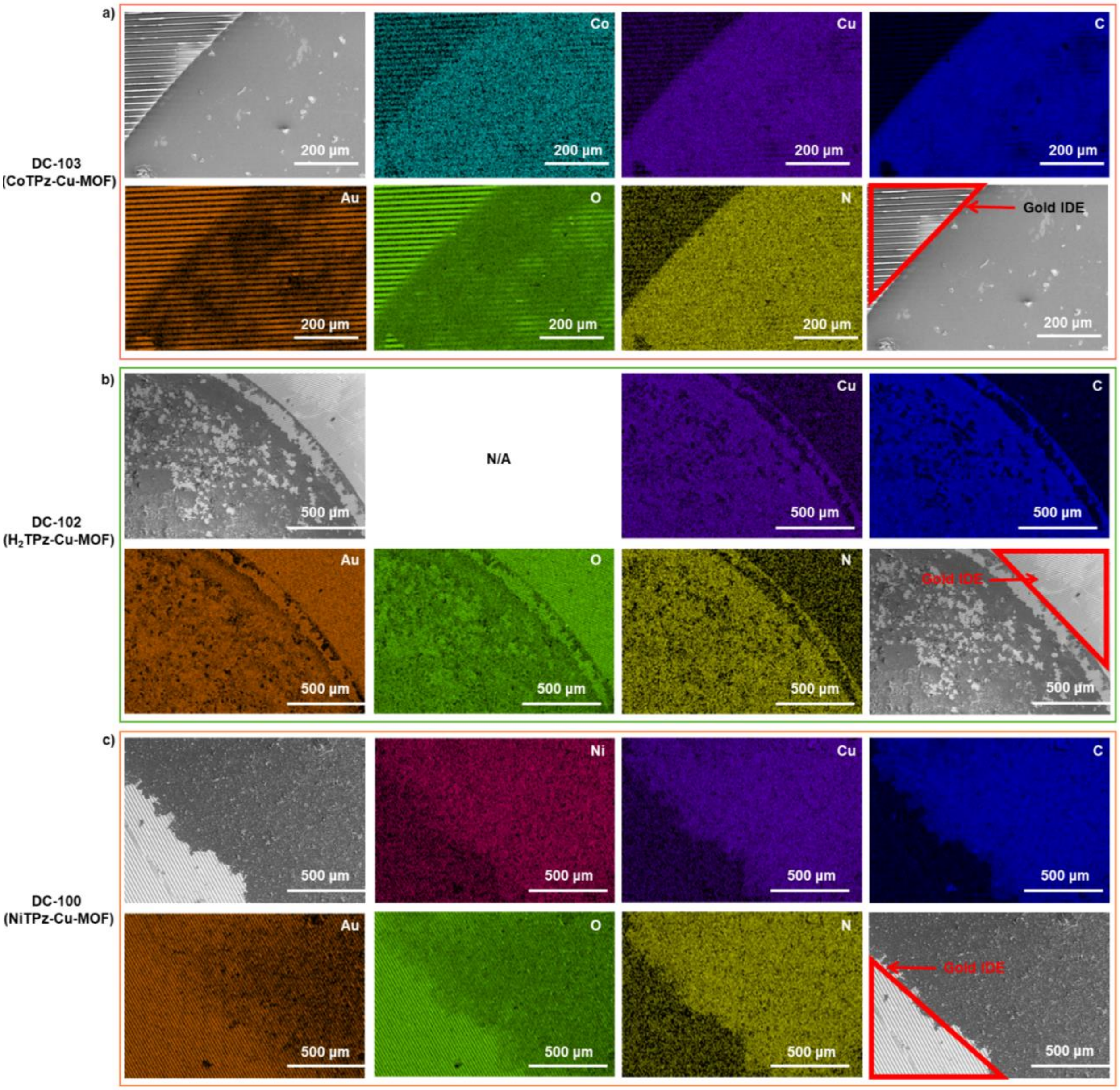

Figure S118. EDX mapping of the devices upon dropcasting of a) DC-103, b) DC-102, and DC-100 showing the elemental mapping of the MOF elements, as well as of the gold IDE. The red highlighted region in the last figure of each part represents the edge of the gold IDE, with the MOF thin film, dropcasted onto the center of the IDE, represents the rest of the area of the image.

S13. References

1. Zhou, Z.-L.; Weber, E.; Keana, J. F., Acetoxylation of 6, 7-dialkoxy-substituted 1, 4-dihydroquinoxaline-2, 3-diones (Qxs) using fuming nitric acid in acetic acid: A facile synthesis of 5-acyloxy-6, 7-dialkoxy QXs. *Tetrahedron Lett.* **1995,** *36* (42), 7583-7586.
2. Chan, J. Y. M.; Shehayeb, E. O.; Pennington, D. L.; Hendon, C. H.; Mirica, K. A., Molecular Engineering of a Conductive Metal–Organic Framework for Ultrasensitive, Rapid, Selective, and Reversible Sensing of Nitric Oxide. *J. Am. Chem. Soc.* **2025,** *147* (32), 29003-29012.
3. Xie, L. S.; Skorupskii, G.; Dincă, M., Electrically conductive metal–organic frameworks. *Chem. Rev.* **2020,** *120* (16), 8536.
4. Liu, Y.; Li, S.; Dai, L.; Li, J.; Lv, J.; Zhu, Z.; Yin, A.; Li, P.; Wang, B., The Synthesis of Hexaazatrinaphthylene-Based 2D Conjugated Copper Metal-Organic Framework for Highly Selective and Stable Electroreduction of CO_2 to Methane. *Angew. Chem. Int. Ed.* **2021,** *60* (30), 16409-16415.
5. Huang, H.; Zhao, Y.; Bai, Y.; Li, F.; Zhang, Y.; Chen, Y., Conductive Metal–Organic Frameworks with Extra Metallic Sites as an Efficient Electrocatalyst for the Hydrogen Evolution Reaction. *Adv. Sci.* **2020,** *7* (9), 2000012.
6. Ding, J.; Wei, Z.; Li, F.; Zhang, J.; Zhang, Q.; Zhou, J.; Wang, W.; Liu, Y.; Zhang, Z.; Su, X.; Yang, R.; Liu, W.; Su, C.; Yang, H. B.; Huang, Y.; Zhai, Y.; Liu, B., Atomic high-spin cobalt (II) center for highly selective electrochemical CO reduction to CH_3OH. *Nat. Commun.* **2023,** *14* (1), 6550.
7. Smits, F., Measurement of sheet resistivities with the four-point probe. *Bell Syst. Tech. J.* **1958,** *37* (3), 711-718.
8. Zhong, Z.; Damacet, P.; Sánchez-González, E.; Eagleton, A. M.; Vereshchuk, N.; Wongratanaphisan, R.; Anderson, J. T.; Goncalves, S.; Peterson, G. W.; Blount, B.; Monti, S.; Barcaro, G.; Ibarra, I. A.; Mirica, K. A., Scalable templated fabrication of Cu-based MOF on textiles for simultaneous sensing, filtration, and detoxification of SO_2. *Chem* **2025,** *11* (10), 102580.
9. Piątek, J.; Budnyak, T. M.; Monti, S.; Barcaro, G.; Gueret, R.; Grape, E. S.; Jaworski, A.; Inge, A. K.; Rodrigues, B. V. M.; Slabon, A., Toward Sustainable Li-Ion Battery Recycling: Green Metal–Organic Framework as a Molecular Sieve for the Selective Separation of Cobalt and Nickel. *ACS Sustain. Chem. Eng.* **2021,** *9* (29), 9770-9778.
10. Giannozzi, P.; Andreussi, O.; Brumme, T.; Bunau, O.; Buongiorno Nardelli, M.; Calandra, M.; Car, R.; Cavazzoni, C.; Ceresoli, D.; Cococcioni, M.; Colonna, N.; Carnimeo, I.; Dal Corso, A.; de Gironcoli, S.; Delugas, P.; DiStasio, R. A.; Ferretti, A.; Floris, A.; Fratesi, G.; Fugallo, G.; Gebauer, R.; Gerstmann, U.; Giustino, F.; Gorni, T.; Jia, J.; Kawamura, M.; Ko, H. Y.; Kokalj, A.; Küçükbenli, E.; Lazzeri, M.; Marsili, M.; Marzari, N.; Mauri, F.; Nguyen, N. L.; Nguyen, H. V.; Otero-de-la-Roza, A.; Paulatto, L.; Poncé, S.; Rocca, D.; Sabatini, R.; Santra, B.; Schlipf, M.; Seitsonen, A. P.; Smogunov, A.; Timrov, I.; Thonhauser, T.; Umari, P.; Vast, N.; Wu, X.; Baroni, S., Advanced capabilities for materials modelling with Quantum ESPRESSO. *J. Phys. Condens. Matter* **2017,** *29* (46), 465901.
11. Perdew, J. P.; Burke, K.; Ernzerhof, M., Generalized Gradient Approximation Made Simple. *Phys. Rev. Lett.* **1996,** *77* (18), 3865-3868.
12. Kresse, G.; Joubert, D., From ultrasoft pseudopotentials to the projector augmented-wave method. *Phys. Rev. B* **1999,** *59* (3), 1758-1775.
13. Grimme, S.; Antony, J.; Ehrlich, S.; Krieg, H., A consistent and accurate ab initio parametrization of density functional dispersion correction (DFT-D) for the 94 elements H-Pu. *J. Chem. Phys.* **2010,** *132* (15).

14. Damacet, P.; Shehayeb, E. O.; Monti, S.; Barcaro, G.; Mirica, K. A., Redox-active metal–organic framework nanocrystals for the simultaneous adsorption, detection, and detoxification of heavy metal cations. *ACS Appl. Mater. Interfaces* **2025**.
15. Damacet, P.; Chandra, P.; Ambrogi, E. K.; Noh, H.-J.; Asmus, E. L.; Shehayeb, E. O.; Barcaro, G.; Monti, S.; Mirica, K. A., Electronic textiles based on conductive metal–organic frameworks as scavengers and sensors of toxic oxyanions from water. *J. Am. Chem. Soc.* **2025,** *147* (31), 27561-27575.
16. Hou, L.; Duan, J.; Xiong, F.; Carraro, C.; Shi, T.; Maboudian, R.; Long, H., Low Power Gas Sensors: From Structure to Application. *ACS Sens.* **2024,** *9* (12), 6327-6357.
17. Ammu, S.; Dua, V.; Agnihotra, S. R.; Surwade, S. P.; Phulgirkar, A.; Patel, S.; Manohar, S. K., Flexible, all-organic chemiresistor for detecting chemically aggressive vapors. *J. Am. Chem. Soc.* **2012,** *134* (10), 4553-4556.
18. Benedetto, G.; Stolz, R. M.; Meng, Z.; Chan, J. Y. M.; Shehayeb, E. O.; Morrell, C. T.; Fabusola, G.; Elsaesser, N.; Simon, C. M.; Mirica, K. A., Conductive Covalent Organic Frameworks as Chemiresistive Sensor Arrays for the Detection and Differentiation of Gasotransmitters. *J. Am. Chem. Soc.* **2025,** *147* (47), 43438-43452.
19. Ponzoni, A., A statistical analysis of response and recovery times: the case of ethanol chemiresistors based on pure SnO_2. *Sensors* **2022,** *22* (17), 6346.
20. Bufalini, M., Oxidation sulfur dioxide in polluted atmospheres. Review. *Environ. Sci. Technol.* **1971,** *5* (8), 685-700.
21. Liu, T.; Chan, A. W.; Abbatt, J. P., Multiphase oxidation of sulfur dioxide in aerosol particles: Implications for sulfate formation in polluted environments. *Environ. Sci. Technol.* **2021,** *55* (8), 4227-4242.
22. Flamm, D. L.; Bacon, D.; Kinsbron, E.; English, A. T., Chemical Reaction of Sulfur Dioxide at High Humidity and Temperature: Implications for Accelerated Testing. *J. Electrochem. Soc.* **1981,** *128* (3), 679.
23. Urone, P.; Lutsep, H.; Noyes, C. M.; Parcher, J. F., Static studies of sulfur dioxide reactions in air. *Environ. Sci. Technol.* **1968,** *2* (8), 611-618.
24. Tokunaga, O.; Nishimura, K.; Washino, M., Radiation treatment of exhaust gases—II. Oxidation of sulfur dioxide in the moist mixture of oxygen and nitrogen. *Int. J. Appl. Radiat. Isot.* **1978,** *29* (2), 87-90.
25. Kellogg, W.; Cadle, R.; Allen, E.; Lazrus, A.; Martell, E., The Sulfur Cycle: Man's contributions are compared to natural sources of sulfur compounds in the atmosphere and oceans. *Science* **1972,** *175* (4022), 587-596.
26. Smith, R. A.; Alexander, R. B., Correlations between stream sulphate and regional SO_2 emissions. *Nature* **1986,** *322* (6081), 722-724.
27. Hung, H.-M.; Hoffmann, M. R., Oxidation of gas-phase SO_2 on the surfaces of acidic microdroplets: Implications for sulfate and sulfate radical anion formation in the atmospheric liquid phase. *Environ. Sci. Technol.* **2015,** *49* (23), 13768-13776.
28. Cadle, R., Formation and chemical reactions of atmospheric particles. *J. Colloid Interface Sci.* **1972,** *39* (1), 25-31.
29. Tchalala, M.; Bhatt, P.; Chappanda, K.; Tavares, S.; Adil, K.; Belmabkhout, Y.; Shkurenko, A.; Cadiau, A.; Heymans, N.; De Weireld, G., Fluorinated MOF platform for selective removal and sensing of SO_2 from flue gas and air. *Nat. Commun.* **2019,** *10* (1), 1328.
30. Chernikova, V.; Yassine, O.; Shekhah, O.; Eddaoudi, M.; Salama, K. N., Highly sensitive and selective SO_2 MOF sensor: the integration of MFM-300 MOF as a sensitive layer on a capacitive interdigitated electrode. *J. Mater. Chem. A* **2018,** *6* (14), 5550-5554.
31. Zhai, Z.; Wang, J.; Sun, Y.; Hao, X.; Niu, B.; Xie, H.; Li, C., MOFs/nanofiber-based capacitive gas sensors for the highly selective and sensitive sensing of trace SO_2. *Appl. Surf. Sci.* **2023,** *613*, 155772.

32. Zhang, J.; Xia, T.; Zhao, D.; Cui, Y.; Yang, Y.; Qian, G., In situ secondary growth of Eu (III)-organic framework film for fluorescence sensing of sulfur dioxide. *Sens. Actuators B: Chem.* **2018,** *260*, 63-69.
33. Zhao, R.; Wang, W.; Liu, Y.; Petkov, P.; Khan, A. H.; Gao, L.; Zhang, P.; Brunner, E.; Wang, H. I.; Singh, S., A Donor–Acceptor-Type Two-Dimensional Poly(Arylene Vinylene) for Efficient Electron Transport and Sensitive Chemiresistors. *Angew. Chem. Int. Ed.* **2025**, e202504302.
34. Wang, S.; Fu, Y.; Wang, F.; Wang, X.; Yang, Y.; Wang, M.; Wang, J.; Lin, E.; Ma, H.; Chen, Y., Scalable Melt Polymerization Synthesis of Covalent Organic Framework Films for Room Temperature Low-Concentration SO_2 Detection. *J. Am. Chem. Soc.* **2024,** *146* (49), 33509-33517.
35. Srinivasan, P.; Ezhilan, M.; Kulandaisamy, A. J.; Babu, K. J.; Rayappan, J. B. B., Room temperature chemiresistive gas sensors: challenges and strategies—a mini review. *J. Mater. Sci.: Mater. Electron.* **2019,** *30* (17), 15825-15847.
36. Tokranov, A. K.; Nishizawa, N.; Amadei, C. A.; Zenobio, J. E.; Pickard, H. M.; Allen, J. G.; Vecitis, C. D.; Sunderland, E. M., How Do We Measure Poly- and Perfluoroalkyl Substances (PFASs) at the Surface of Consumer Products? *Environ. Sci. Technol. Lett.* **2019,** *6* (1), 38-43.
37. Thompson, A. P.; Aktulga, H. M.; Berger, R.; Bolintineanu, D. S.; Brown, W. M.; Crozier, P. S.; in 't Veld, P. J.; Kohlmeyer, A.; Moore, S. G.; Nguyen, T. D.; Shan, R.; Stevens, M. J.; Tranchida, J.; Trott, C.; Plimpton, S. J., LAMMPS - a flexible simulation tool for particle-based materials modeling at the atomic, meso, and continuum scales. *Comput. Phys. Commun.* **2022,** *271*, 108171.
38. Jurcik, A.; Bednar, D.; Byska, J.; Marques, S. M.; Furmanova, K.; Daniel, L.; Kokkonen, P.; Brezovsky, J.; Strnad, O.; Stourac, J.; Pavelka, A.; Manak, M.; Damborsky, J.; Kozlikova, B., CAVER Analyst 2.0: analysis and visualization of channels and tunnels in protein structures and molecular dynamics trajectories. *Bioinformatics* **2018,** *34* (20), 3586-3588.
39. Braun, E., Open source code: Calculating an IR spectra from a LAMMPS simulation. *Zenodo* **2016**.
40. Aykanat, A.; Meng, Z.; Stolz, R. M.; Morrell, C. T.; Mirica, K. A., Bimetallic Two-Dimensional Metal–Organic Frameworks for the Chemiresistive Detection of Carbon Monoxide. *Angew. Chem. Int. Ed.* **2022,** *61* (6), e202113665.
41. Lazanas, A. C.; Prodromidis, M. I., Electrochemical Impedance Spectroscopy─A Tutorial. *ACS Meas. Sci. Au* **2023,** *3* (3), 162-193.
42. Balasubramani, V.; Chandraleka, S.; Rao, T. S.; Sasikumar, R.; Kuppusamy, M. R.; Sridhar, T. M., Review—Recent Advances in Electrochemical Impedance Spectroscopy Based Toxic Gas Sensors Using Semiconducting Metal Oxides. *J. Electrochem. Soc.* **2020,** *167* (3), 037572.
43. Kato, H., Size Determination of Nanoparticles by Dynamic Light Scattering. In *Nanomaterials*, 2012; pp 535-554.
